# BEXCIS: Bayesian methods for estimating the degree of the skewness of X chromosome inactivation

**DOI:** 10.1186/s12859-022-04721-y

**Published:** 2022-05-24

**Authors:** Wen-Yi Yu, Yu Zhang, Meng-Kai Li, Zi-Ying Yang, Wing Kam Fung, Pei-Zhen Zhao, Ji-Yuan Zhou

**Affiliations:** 1 Department of Biostatistics, State Key Laboratory of Organ Failure Research, Ministry of Education, and Guangdong Provincial Key Laboratory of Tropical Disease Research, School of Public Health, Southern Medical University, Guangzhou, China; 2 Guangdong-Hong Kong-Macao Joint Laboratory for Contaminants Exposure and Health, Guangzhou, China; 3 Department of Statistics and Actuarial Science, The University of Hong Kong, Hong Kong, China

**Keywords:** Skewed X chromosome inactivation, Bayesian method, Penalized Fieller’s method, Graves’ disease data, Minnesota Center for Twin and Family Research data

## Abstract

**Background:**

X chromosome inactivation (XCI) is an epigenetic phenomenon that one of two X chromosomes in females is transcriptionally silenced during early embryonic development. Skewed XCI has been reported to be associated with some X-linked diseases. There have been several methods measuring the degree of the skewness of XCI. However, these methods may still have several limitations.

**Results:**

We propose a Bayesian method to obtain the point estimate and the credible interval of the degree of XCI skewing by incorporating its prior information of being between 0 and 2. We consider a normal prior and a uniform prior for it (respectively denoted by BN and BU). We also propose a penalized point estimate based on the penalized Fieller’s method and derive the corresponding confidence interval. Simulation results demonstrate that the BN and BU methods can solve the problems of extreme point estimates, noninformative intervals, empty sets and discontinuous intervals. The BN method generally outperforms other methods with the lowest mean squared error in the point estimation, and well controls the coverage probability with the smallest median and the least variation of the interval width in the interval estimation. We apply all the methods to the Graves’ disease data and the Minnesota Center for Twin and Family Research data, and find that SNP rs3827440 in the Graves’ disease data may undergo skewed XCI towards the allele *C*.

**Conclusions:**

We recommend the BN method for measuring the degree of the skewness of XCI in practice. The R package BEXCIS is publicly available at https://github.com/Wen-YiYu/BEXCIS.

**Supplementary Information:**

The online version contains supplementary material available at 10.1186/s12859-022-04721-y.

## Background

X chromosome inactivation (XCI) [1, 2] is an epigenetic phenomenon which only occurs in female mammals. By the process of XCI, one of two X chromosomes in females will be transcriptionally silenced during the early development of embryos, to ensure that the transcriptional dosages on X chromosome are balanced between males and females [3]. There are three patterns of XCI [4], which are random XCI (XCI-R), skewed XCI (XCI-S) and escape from XCI (XCI-E). Generally, XCI-R is a random and independent selection process in each cell of females, i.e., $50\%$ cells have either the paternal or maternal allele silenced and the remaining $50\%$ keep the other allele inactivated at an X-chromosomal locus [4]. XCI-E means that both the paternal and maternal alleles at a locus will be active. In humans, 15–30% X-linked genes have been shown to undergo XCI-E [5, 6]. Besides, XCI-S is the observation that the same allele is inactivated in more than $75\%$ cells in females [7–9], and the extreme XCI-S is a phenomenon that at least $90\%$ cells in females keep the same allele inactivated [10]. Due to the analytical complications caused by XCI, association tests for detecting disease-associated single nucleotide polymorphisms (SNPs) on autosomes may not be directly applied to X chromosome.

Researchers have proposed some methods to test for the association on X chromosome for qualitative traits [11–17] and quantitative traits [18–21]. For qualitative traits, Zheng et al. [11] took account of XCI-E and put forward a series of test statistics combining the genetic effect in two sexes. Clayton [12] first incorporated XCI-R into the association analysis by regarding males as homozygous females. However, Clayton’s methods do not consider the XCI-E or all the XCI-S patterns. As such, Wang et al. [13] proposed a resampling-based maximum likelihood ratio approach for qualitative traits, which is robust to any XCI pattern. For XCI-E, Wang et al. [13] coded three genotypes in females (*dd*, *Dd* and *DD*) as 0, 1 and 2 and coded two genotypes in males (*d* and *D*) as 0 and 1, where *d* is the normal allele and *D* is the deleterious allele at the SNP under study. For XCI-R and XCI-S, three genotypes in females were coded as 0, $\gamma$ and 2 and two genotypes in males were coded as 0 and 2, respectively, where $\gamma \in [0,\ 2]$ is an unknown genotypic value for heterozygous females and can be used to measure the degree of XCI-S [13]. The value of $\gamma$ not only reveals the potential XCI pattern but also gives us a hint about the proportion of the cells in females expressing the normal allele *d* or the deleterious allele *D* at the SNP. Specifically, $\gamma \in [0,\ 1)$ means XCI-S skewed towards *D*, $\gamma =1$ represents XCI-R, and $\gamma \in (1,\ 2]$ suggests XCI-S skewed towards *d*. If the estimate of $\gamma$ is significantly different from 1, the SNP is statistically inferred to undergo XCI-S, otherwise, the SNP may undergo XCI-R or XCI-E. For example, $\gamma =0.4$ represents XCI-S skewed towards *D*, where only about $20\%\ (0.4/2)$ of the cells have *D* active and the other $80\%$ of the cells have *d* active. For quantitative traits, Zhang et al. [18] proposed an association test based on nuclear families, which requires the quantitative trait being normally distributed and assumes that the variances of the trait value for the three genotypes in females are the same. However, Ma et al. [19] reported that XCI and other factors (e.g., gene-gene interactions and gene mutation) may cause higher variance of the trait in heterozygous females compared to homozygous females. As a result, Ma et al. [19] proposed three methods for testing the association based on unrelated females, which take account of the inflated variance of the quantitative trait in heterozygous females. Gao et al. [20] further developed a software toolset, which can implement the three test statistics in Ma et al. [19].

In addition to the detection of the disease-associated SNPs on X chromosome, it is also important to measure the degree of XCI-S. It has been reported that the degree of XCI-S may increase with age [4] and is associated with many diseases such as scleroderma, rheumatoid arthritis, breast cancer, ovarian cancer, severe combined immunodeficiency and so on [22–28]. For heterozygous females, larger proportion of the cells with active deleterious allele will lead to more severe expression of the related diseases, while smaller proportion can protect the body from negative effects, which suggests that XCI-S is somehow both a confounding factor in genetic association analysis and a critical tool providing valuable information about the pathogenesis at the X-chromosomal locus [22]. Therefore, methods for measuring the skewness of XCI are necessary and researchers have provided several methods for qualitative traits [29, 30] and quantitative traits [31]. Specifically, Xu et al. [29] proposed a statistical measure for $\gamma$ based on family trios and derived the corresponding confidence interval (CI) with the likelihood ratio (LR) test. Based on case-control design, Wang et al. [30] showed that $\gamma$ can be expressed as a ratio of two logistic regression coefficients and derived three types of the CIs for $\gamma$ (the LR, Fieller’s and delta methods). The Fieller’s and LR methods outperform the delta method and the Fieller’s method is recommended because it is non-iterative and requires much less computations than the LR method. Since the approach of Xu et al. [29] and those of Wang et al. [30] are only applicable to qualitative traits, Li et al. [31] extended the methods of Wang et al. [30] to make them accommodate quantitative traits. Note that both the Fieller’s and LR methods may cause unbounded CIs if the denominator of the ratio is not significantly deviated from 0 [30]. Fortunately, Wang et al. [32] proposed a penalized Fieller’s (PF) method for the ratio estimate, which can always obtain a bounded CI with an appropriate penalty parameter. The PF method has never been used to measure the degree of XCI-S, and we will apply it to such task for the first time. However, all the existing methods for measuring $\gamma$ do not consider the constraint condition that the value of $\gamma$ should be between 0 and 2. They simply cut off the point estimates and the corresponding CIs into $[0,\ 2]$ to get the final results, which may lead to extreme point estimates (0 or 2) as well as noninformative CIs ($[0,\ 2]$) or invalid CIs (empty sets). In contrast, the Bayesian method [33, 34] can incorporate the prior information and has been widely used in statistical genetics in recent years [35]. To make an improvement, we will apply the Bayesian method to the $\gamma$ measuring problem so that we can make full use of the prior information of $\gamma$ and obtain more accurate and robust point estimate and credible interval for $\gamma$.

Therefore, in this article, borrowing the idea of Wang et al. [32], we first derive a penalized point estimate to measure the degree of XCI-S ($\gamma$) and compute the corresponding CI by the PF method. Then, we propose a Bayesian method to obtain the samples of $\gamma$ from its approximate posterior distribution and calculate the mode of the samples as its point estimate and the highest posterior density interval (HPDI) as its credible interval [36]. We conduct extensive simulation studies to compare the proposed Bayesian and penalized point estimates with the existing point estimate, as well as to compare the Bayesian and PF methods with the existing Fieller’s method in the interval estimation, respectively. Finally, we apply all the methods to the Graves’ disease data and the Minnesota Center for Twins and Family Research (MCTFR) data for their practice on the qualitative trait and the quantitative trait, respectively.

## Results

### Simulation results

To evaluate the performances of the proposed point estimation and interval estimation methods, we conduct extensive simulation studies. Assume that $\sigma _{0}^{2}$, $\sigma _{1}^{2}$ and $\sigma _{2}^{2}$ are the variances of the quantitative trait for females with genotypes *dd*, *Dd* and *DD*, respectively. We consider the qualitative trait and the quantitative trait when $(\sigma _{0}^{2},\ \sigma _{1}^{2},\ \sigma _{2}^{2})=(1,\ 1.2,\ 1)$ and $(\sigma _{0}^{2},\ \sigma _{1}^{2},\ \sigma _{2}^{2})=(4,\ 4.8,\ 4)$, and the sample size *n* is taken as 500 and 2,000, the minor allele frequency (MAF) is fixed at 0.3 and 0.1, and the inbreeding coefficient $\rho$ is set to be 0, -0.05 and 0.05, where $\rho =0$ means that the Hardy–Weinberg equilibrium (HWE) holds in females and $\rho \ne 0$ denotes the departure from HWE in females. We simulate 500 SNPs with stochastic underlying $\gamma$’s for each scenario. The penalized point estimate and the existing point estimate of $\gamma$ may obtain the point estimate less than 0 or larger than 2 while the value of $\gamma$ should be within $[0,\ 2]$, so we need to truncate the penalized point estimate and the existing point estimate into $[0,\ 2]$ to get the final results. We denote the penalized point estimate and the existing point estimate before truncation as ${\hat{\gamma }}^{*}_{origin}$ and ${\hat{\gamma }}_{origin}$, and denote those after truncation as ${\hat{\gamma }}_{PF}$ and ${\hat{\gamma }}_{F}$, respectively. We also use the Bayesian methods with the normal prior and the uniform prior for $\gamma$ (represented by BN and BU) to obtain the point estimate of $\gamma$, which are denoted as ${\hat{\gamma }}_{BN}$ and ${\hat{\gamma }}_{BU}$, respectively. To reveal the accuracy and robustness of ${\hat{\gamma }}_{BN}$, ${\hat{\gamma }}_{BU}$, ${\hat{\gamma }}_{PF}$ and ${\hat{\gamma }}_{F}$, we calculate their mean squared errors (MSEs) and summarize the proportions of the extreme values (0 or 2) they get among the 500 replicates, respectively. Here, $\text {MSE}=\frac{\sum _{k=1}^{K}({\hat{\gamma }}_{k}-\gamma _k)^{2}}{K}$, where *K* is the number of replicates, $\gamma _{k}$ is the *k*th true value of $\gamma$, and ${\hat{\gamma }}_{k}$ is the estimate of $\gamma _{k}$. We also draw scatter plots to directly display the four point estimates against the true values of $\gamma$. To investigate the performances of the BN, BU, PF and Fieller’s methods, we respectively assess the coverage probability (CP) as well as the mean, median, standard deviation and interquartile range of the widths of the $95\%$ HPDIs or CIs of $\gamma$ (denoted by $\hbox {W}_{\mathrm{mean}}$, $\hbox {W}_{\mathrm{median}}$, $\hbox {W}_{\mathrm{SD}}$ and $\hbox {W}_{\mathrm{IQR}}$) for them. We compute the proportions of the noninformative interval ($[0,\ 2]$), empty set and discontinuous interval they obtain among the 500 replicates (denoted by NP, EP and DP) to further confirm these methods’ validity. Scatter plots are drawn to show the widths of the $95\%$ HPDIs or CIs of these methods against the true values of $\gamma$.

**Fig. 1:**
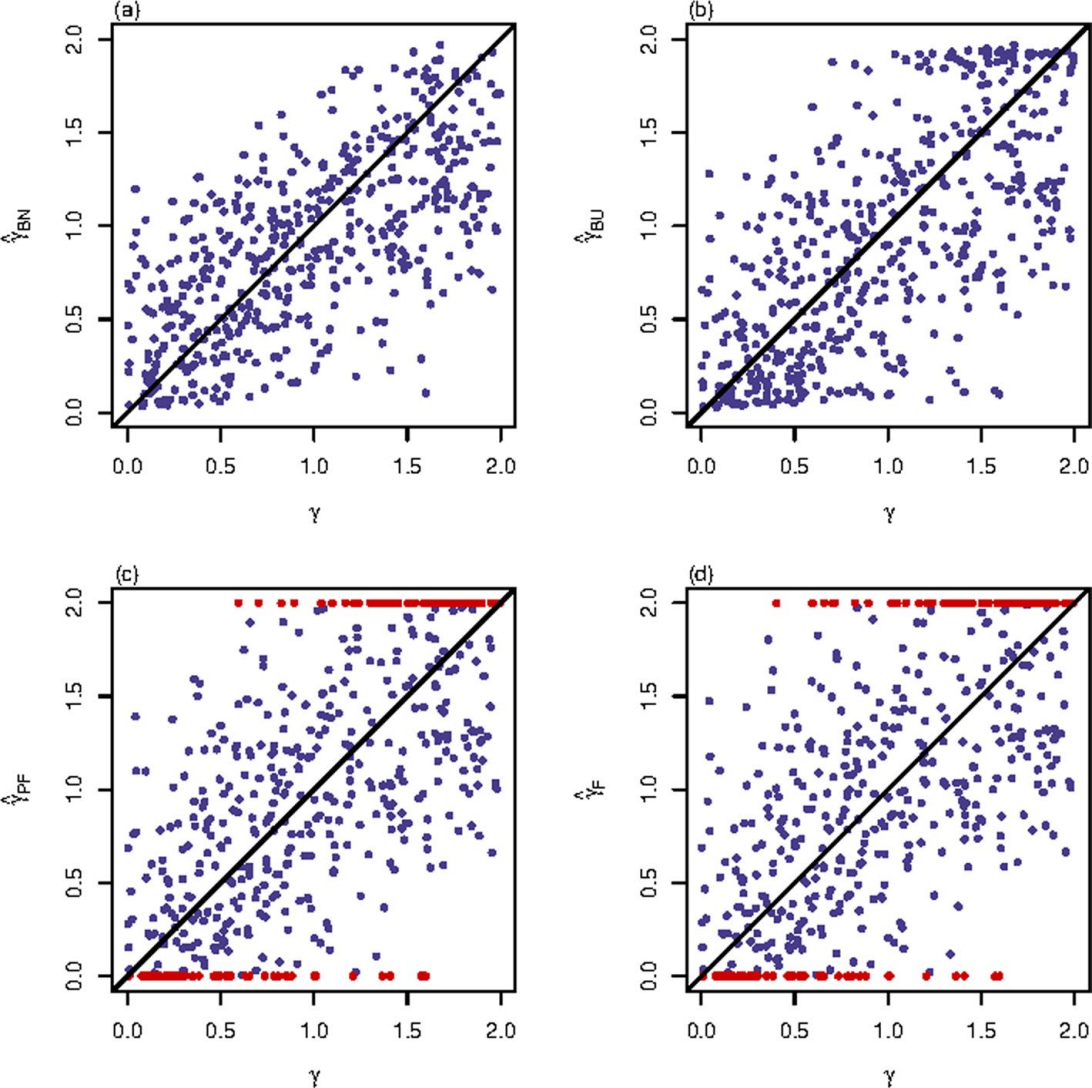
Scatter plots of point estimates of $\gamma$ for qualitative trait with $n=500$, $\text {MAF}=0.3$ and $\rho =0$. The results are against true value of $\gamma$. The red points represent the extreme values (0 or 2). **a** ${\hat{\gamma }}_{BN}$; **b** ${\hat{\gamma }}_{BU}$; **c** ${\hat{\gamma }}_{PF}$; **d** ${\hat{\gamma }}_{F}$

**Fig. 2:**
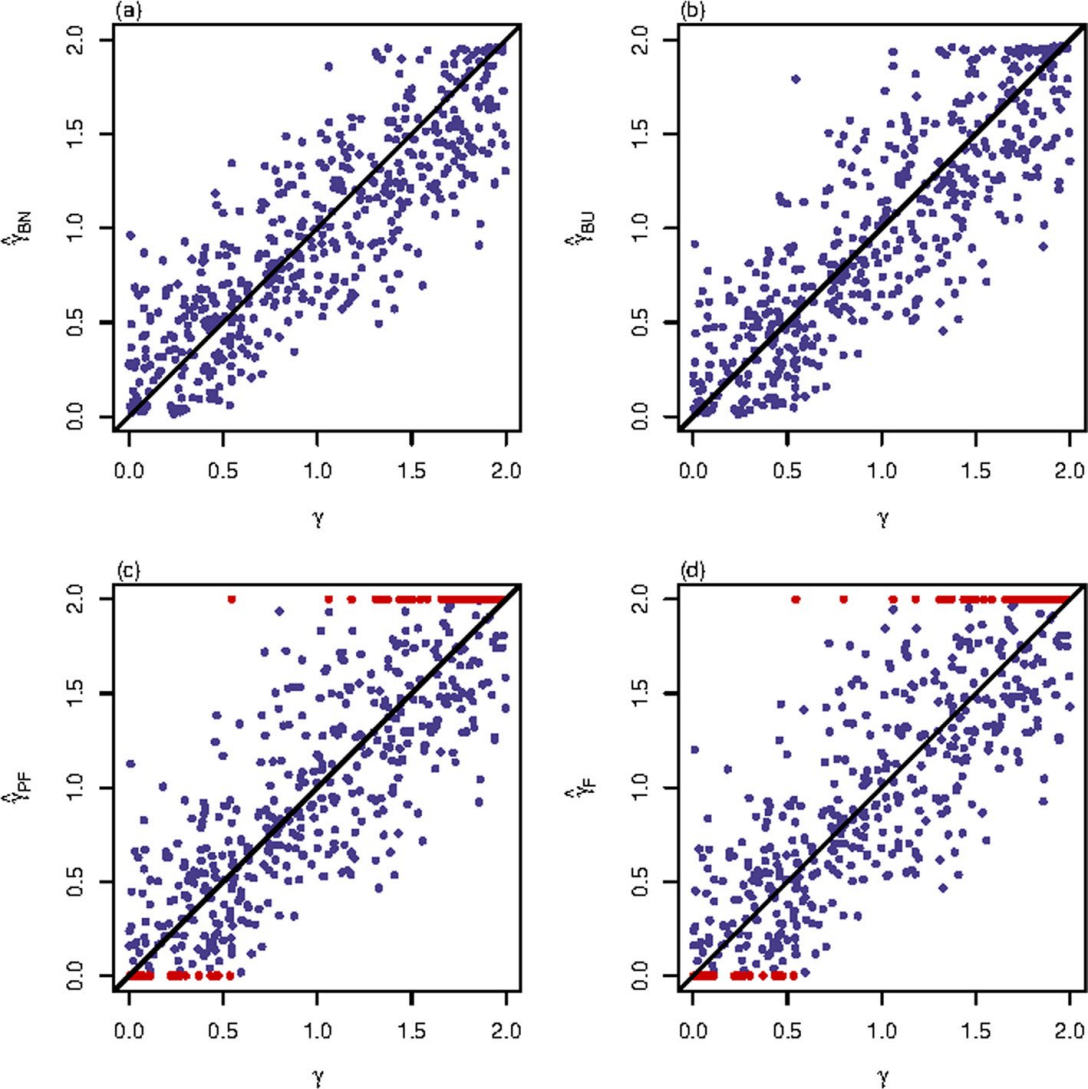
Scatter plots of point estimates of $\gamma$ for quantitative trait with $n=500$, $\text {MAF}=0.3$ and $\rho =0$. The results are against true value of $\gamma$ with $(\sigma _{0}^{2},\ \sigma _{1}^{2},\ \sigma _{2}^{2})=(1,\ 1.2,\ 1)$. The red points represent the extreme values (0 or 2). **a** ${\hat{\gamma }}_{BN}$; **b** ${\hat{\gamma }}_{BU}$; **c** ${\hat{\gamma }}_{PF}$; **d** ${\hat{\gamma }}_{F}$

The proportions of the extreme values of ${\hat{\gamma }}_{PF}$ and ${\hat{\gamma }}_{F}$ among the 500 replicates for qualitative trait and quantitative trait with $(\sigma _{0}^{2},\ \sigma _{1}^{2},\ \sigma _{2}^{2})=(1,\ 1.2,\ 1)$ are presented in Table 1, the MSEs of ${\hat{\gamma }}_{BN}$, ${\hat{\gamma }}_{BU}$, ${\hat{\gamma }}_{PF}$ and ${\hat{\gamma }}_{F}$ for qualitative trait and quantitative trait with $(\sigma _{0}^{2},\ \sigma _{1}^{2},\ \sigma _{2}^{2})=(1,\ 1.2,\ 1)$ are shown in Table 2, and the scatter plots of these four point estimates against the true values of $\gamma$ under these settings are respectively displayed in Figs. 1, 2 and Additional file 1: Figs. S1–S22. Note that ${\hat{\gamma }}_{BN}$ and ${\hat{\gamma }}_{BU}$ can solve the problem of extreme point estimates and thus are not listed in Table 1. Comparing the proportions of the extreme values of ${\hat{\gamma }}_{PF}$ with those of ${\hat{\gamma }}_{F}$ in Table 1, ${\hat{\gamma }}_{PF}$ can reduce the proportion of the extreme point estimates equal to 2. An explanation for this is that ${\hat{\gamma }}_{PF}$ is obtained by shrinking the denominator of ${\hat{\gamma }}_{F}$ away from 0 and accordingly adjusting the numerator of ${\hat{\gamma }}_{F}$, which can avoid the point estimate being positive infinity before the truncation (since ${\hat{\beta }}_{1}$ and ${\hat{\beta }}_{2}$ in ${\hat{\gamma }}_{origin}=\frac{2{\hat{\beta }}_{1}}{{\hat{\beta }}_{1}+{\hat{\beta }}_{2}}$ usually have the same sign [30]) and hence can cut down the proportion of the point estimates equal to 2 after the truncation. On the other hand, we can see from Table 1 that the proportions of the extreme point estimates equal to 0 are the same for ${\hat{\gamma }}_{PF}$ and ${\hat{\gamma }}_{F}$. Note that ${\hat{\gamma }}^{*}_{origin}$ and ${\hat{\gamma }}_{origin}$ always have the same sign if they are not zero. When ${\hat{\gamma }}^{*}_{origin}$ and ${\hat{\gamma }}_{origin}$ are negative (${\hat{\beta }}_{1}$ and ${\hat{\beta }}_{2}$ have different signs), ${\hat{\gamma }}_{PF}={\hat{\gamma }}_{F}=0$, and when they are positive, ${\hat{\gamma }}_{PF}$ and ${\hat{\gamma }}_{F}$ will both be greater than 0. That is why ${\hat{\gamma }}_{PF}$ and ${\hat{\gamma }}_{F}$ always have the same amount of the extreme point estimates equal to 0. It can also be observed from Table 1 that the total proportions of the extreme point estimates in ${\hat{\gamma }}_{PF}$ and ${\hat{\gamma }}_{F}$ both decrease when *n* becomes larger, MAF gets higher or the trait turns from qualitative into quantitative.

**Table 1:** Proportions (in $\%$) of extreme values of ${\hat{\gamma }}_{PF}$ and ${\hat{\gamma }}_{F}$ among 500 replicates

Trait	*n*	MAF	${\rho }$	${{\hat{\gamma }}_{PF}}$			${{\hat{\gamma }}_{F}}$		
				0	2	Total	0	2	Total
Qualitative	500	0.3	0	11.8	13.0	24.8	11.8	15.2	27.0
			− 0.05	12.2	15.0	27.2	12.2	18.0	30.2
			0.05	9.2	16.6	25.8	9.2	18.8	28.0
		0.1	0	23.4	6.8	30.2	23.4	19.2	42.6
			− 0.05	25.0	4.2	29.2	25.0	20.8	45.8
			0.05	24.8	9.6	34.4	24.8	17.8	42.6
	2000	0.3	0	3.6	6.8	10.4	3.6	6.8	10.4
			− 0.05	5.4	7.2	12.6	5.4	7.2	12.6
			0.05	6.2	7.2	13.4	6.2	7.6	13.8
		0.1	0	8.8	15.0	23.8	8.8	19.4	28.2
			− 0.05	13.4	12.0	25.4	13.4	20.6	34.0
			0.05	7.4	13.4	20.8	7.4	17.6	25.0
Quantitative	500	0.3	0	5.4	10.4	15.8	5.4	10.6	16.0
			− 0.05	6.4	11.6	18.0	6.4	12.6	19.0
			0.05	7.0	8.6	15.6	7.0	8.8	15.8
		0.1	0	14.2	14.0	28.2	14.2	19.8	34.0
			− 0.05	20.8	10.0	30.8	20.8	16.6	37.4
			0.05	12.6	12.8	25.4	12.6	17.4	30.0
	2000	0.3	0	2.6	4.8	7.4	2.6	4.8	7.4
			− 0.05	3.0	5.6	8.6	3.0	5.6	8.6
			0.05	3.0	6.0	9.0	3.0	6.0	9.0
		0.1	0	3.6	13.4	17.0	3.6	14.0	17.6
			− 0.05	4.0	15.6	19.6	4.0	19.8	23.8
			0.05	5.6	10.2	15.8	5.6	10.6	16.2

Proportions (in $\%$) are given under qualitative trait and quantitative trait with $(\sigma _{0}^{2},\ \sigma _{1}^{2},\ \sigma _{2}^{2})=(1,\ 1.2,\ 1)$

**Table 2:** MSEs of ${\hat{\gamma }}_{BN}$, ${\hat{\gamma }}_{BU}$, ${\hat{\gamma }}_{PF}$ and ${\hat{\gamma }}_{F}$

Trait	*n*	MAF	${\rho }$	${{\hat{\gamma }}_{BN}}$	${{\hat{\gamma }}_{BU}}$	${{\hat{\gamma }}_{PF}}$	${{\hat{\gamma }}_{F}}$
Qualitative	500	0.3	0	0.1897	0.2192	0.2686	0.2868
			− 0.05	0.1746	0.2023	0.2960	0.3234
			0.05	0.1705	0.2056	0.2511	0.2608
		0.1	0	0.3838	0.4456	0.5163	0.6584
			− 0.05	0.4707	0.5374	0.5525	0.6978
			0.05	0.3943	0.4642	0.5603	0.6660
	2000	0.3	0	0.0736	0.0768	0.0848	0.0857
			− 0.05	0.0703	0.0742	0.0808	0.0820
			0.05	0.0689	0.0709	0.0756	0.0760
		0.1	0	0.1811	0.1921	0.2725	0.3266
			− 0.05	0.2213	0.2435	0.3466	0.4329
			0.05	0.1698	0.1847	0.2265	0.2663
Quantitative	500	0.3	0	0.0972	0.1062	0.1235	0.1267
			− 0.05	0.0890	0.1009	0.1237	0.1283
			0.05	0.0894	0.0988	0.1125	0.1143
		0.1	0	0.2285	0.2596	0.4325	0.5005
			− 0.05	0.2319	0.2692	0.4416	0.4850
			0.05	0.2179	0.2436	0.3356	0.3884
	2000	0.3	0	0.0320	0.0334	0.0345	0.0345
			− 0.05	0.0332	0.0340	0.0349	0.0350
			0.05	0.0307	0.0316	0.0324	0.0324
		0.1	0	0.1065	0.1170	0.1300	0.1379
			− 0.05	0.1391	0.1521	0.2009	0.2214
			0.05	0.1001	0.1049	0.1312	0.1446

Mean squared errors (MSEs) are given under qualitative trait and quantitative trait with $(\sigma _{0}^{2},\ \sigma _{1}^{2},\ \sigma _{2}^{2})=(1,\ 1.2,\ 1)$

In addition to the advantage of avoiding the extreme point estimates, it can be seen from Table 2 that ${\hat{\gamma }}_{BN}$ and ${\hat{\gamma }}_{BU}$ always have smaller MSEs than ${\hat{\gamma }}_{PF}$ and ${\hat{\gamma }}_{F}$, and the MSEs of ${\hat{\gamma }}_{BN}$ remain the smallest across all the situations. Irrespective of other factors, we find that $\rho$ generally has a little effect on the MSEs of the four point estimates, which means that the four point estimates are robust to the deviation from HWE in general. When other parameters remain unchanged, the MSEs of the four point estimates all become smaller with larger *n* or higher MAF. Compared to the qualitative trait, all the four point estimation methods give less MSEs for the quantitative trait with $(\sigma _{0}^{2},\ \sigma _{1}^{2},\ \sigma _{2}^{2})=(1,\ 1.2,\ 1)$, regardless of the values of *n*, MAF and $\rho$.

Figures 1 and 2 and Additional file 1: Figs. S1–S22 not only support the findings of Tables 1 and 2 but also provide extra information on the performances of the four point estimation methods under different true values of $\gamma$. Specifically, Fig. 1 presents the four point estimates of $\gamma$ against the true values of $\gamma$ with $n=500$, $\text {MAF}=0.3$ and $\rho =0$ for qualitative trait. Fig. 1a shows good agreement between ${\hat{\gamma }}_{BN}$ and the true values of $\gamma$, while Fig. 1b presents larger discrepancies between ${\hat{\gamma }}_{BU}$ and the true values of $\gamma$, which means that ${\hat{\gamma }}_{BN}$ performs better than ${\hat{\gamma }}_{BU}$ under this situation. Compared to Fig. 1a–c for ${\hat{\gamma }}_{PF}$ and Fig. 1d for ${\hat{\gamma }}_{F}$ both display worse point estimates with the existence of extreme values (represented by red points). Similar results can be found in all the other cases (Fig. 2 and Additional file 1: Figs. S1–S22), which indicates that ${\hat{\gamma }}_{BN}$ and ${\hat{\gamma }}_{BU}$ have better performances than ${\hat{\gamma }}_{PF}$ and ${\hat{\gamma }}_{F}$, and ${\hat{\gamma }}_{BN}$ is generally the best one among these four point estimates across all the simulation scenarios. Figure 2 gives the four point estimates of $\gamma$ against the true values of $\gamma$ with $n=500$, $\text {MAF}=0.3$ and $\rho =0$ for the quantitative trait when $(\sigma _{0}^{2},\ \sigma _{1}^{2},\ \sigma _{2}^{2})=(1,\ 1.2,\ 1)$. In the comparison of Figs. 1 and 2, we see that the four point estimation methods provide better point estimates for the quantitative trait with $(\sigma _{0}^{2},\ \sigma _{1}^{2},\ \sigma _{2}^{2})=(1,\ 1.2,\ 1)$ than for the qualitative trait (can also be seen in Additional file 1: Figs. S1–S11 vs. S12–S22). Similarly, we have the same findings on the effects of *n*, MAF and $\rho$ on the performances of these four point estimation methods from Figs. 1, 2 and Additional file 1: Figs. S1–S22 as we did in Table 2. In addition, the four point estimates are generally scattered evenly around the true values of $\gamma$ except for those settings when $n=500$ and $\text {MAF}=0.1$ for qualitative trait, where ${\hat{\gamma }}_{BN}$ and ${\hat{\gamma }}_{BU}$ tend to underestimate the true value of $\gamma$ (Additional file 1: Figs. S3–S5). The four point estimation methods obtain their best performance at $n=2000$ and $\text {MAF}=0.3$ for quantitative trait when $(\sigma _{0}^{2},\ \sigma _{1}^{2},\ \sigma _{2}^{2})=(1,\ 1.2,\ 1)$ (Additional file 1: Figs. S17–S19), where ${\hat{\gamma }}_{PF}$ and ${\hat{\gamma }}_{F}$ still have a small amount of extreme point estimates (represented by red points) when the true values of $\gamma$ are smaller than 0.5 or larger than 1.5.

**Fig. 3:**
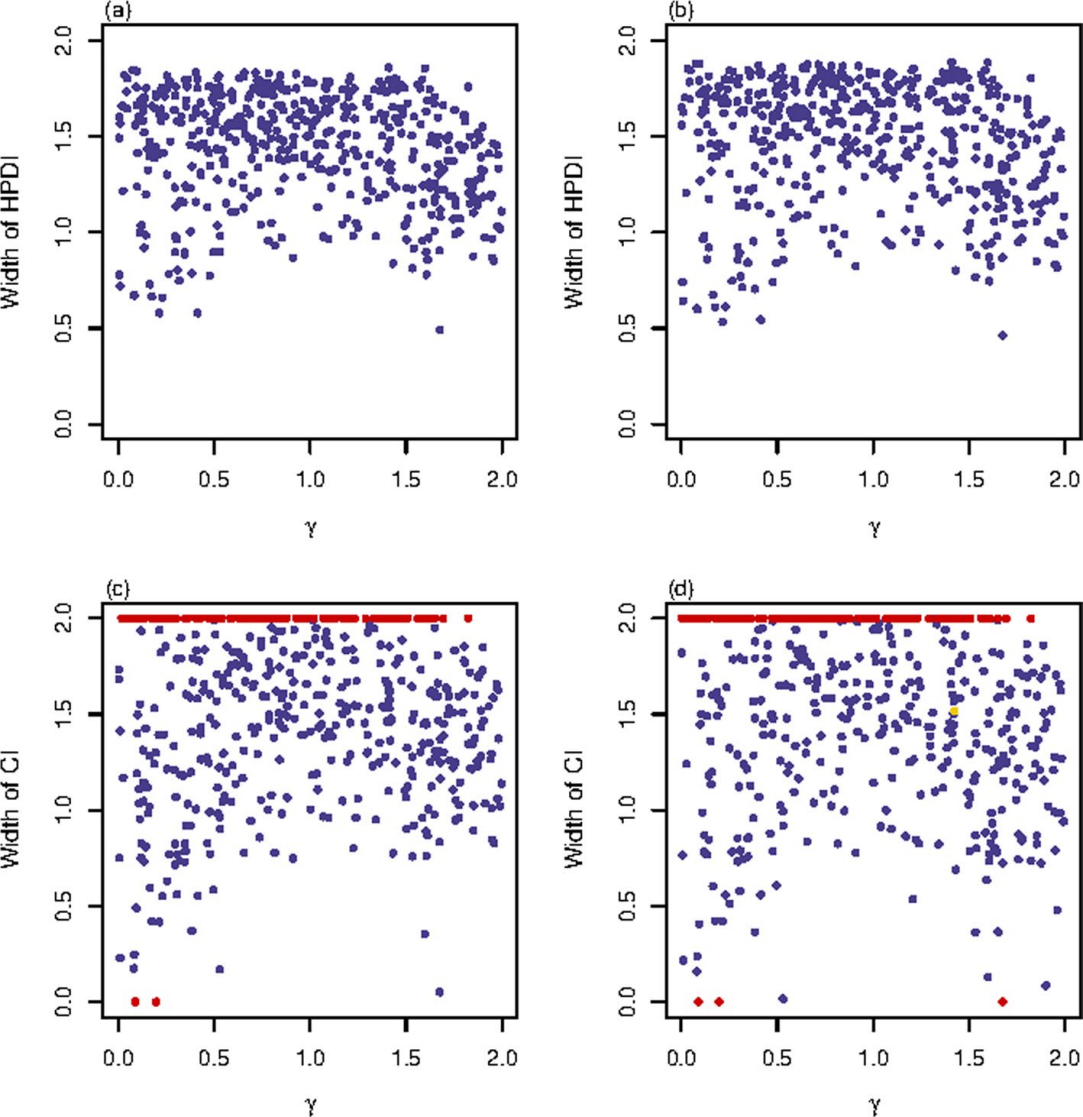
Widths of HPDIs or CIs for qualitative trait with $n=500$, $\text {MAF}=0.3$ and $\rho =0$. The results are against true value of $\gamma$. The red points represent the widths of the noninformative intervals or the empty sets, and the yellow point represents the width of the discontinuous interval. **a** BN method; **b** BU method; **c** PF method; **d** Fieller’s method

**Fig. 4:**
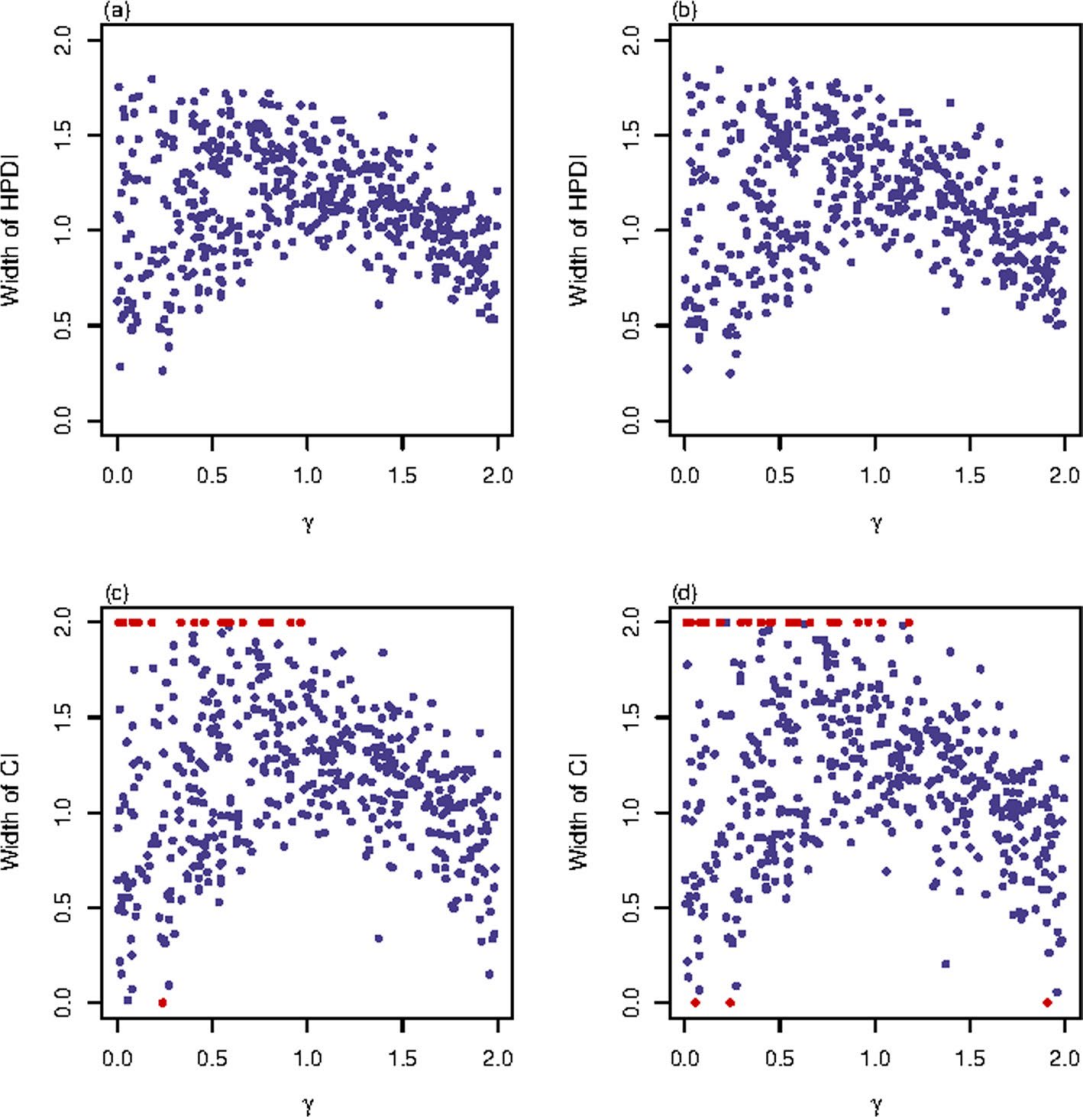
Widths of HPDIs or CIs for quantitative trait with $n=500$, $\text {MAF}=0.3$ and $\rho =0$. The results are against true value of $\gamma$ with $(\sigma _{0}^{2},\ \sigma _{1}^{2},\ \sigma _{2}^{2})=(1,\ 1.2,\ 1)$. The red points represent the widths of the noninformative intervals or the empty sets. **a** BN method; **b** BU method; **c** PF method; **d** Fieller’s method

The NP, EP and DP of the PF and Fieller’s methods among the 500 replicates for qualitative trait and quantitative trait with $(\sigma _{0}^{2},\ \sigma _{1}^{2},\ \sigma _{2}^{2})=(1,\ 1.2,\ 1)$ are displayed in Table 3, the CP, $\hbox {W}_{\mathrm{mean}}$ and $\hbox {W}_{\mathrm{median}}$ of the BN, BU, PF and Fieller’s methods for qualitative trait and quantitative trait with $(\sigma _{0}^{2},\ \sigma _{1}^{2},\ \sigma _{2}^{2})=(1,\ 1.2,\ 1)$ are listed in Table 4, and the widths of the $95\%$ HPDIs or CIs for these four interval estimation methods against the true values of $\gamma$ under these settings are respectively presented in Figs. 3, 4 and Additional file 2: Figs. S23–S44. Notice that the BN and BU methods are not listed in Table 3 because of the superiority of the BN and BU methods over the other two methods that they have no noninformative HPDI, empty set or discontinuous HPDI under all the situations. We can see from Table 3 that the DPs of the PF method are all equal to 0 because we choose a sufficiently large penalty parameter $\lambda$ ($\lambda = \frac{z^{2}_{1-\alpha /2}}{4}$) for the PF method, while the Fieller’s method may obtain nonzero DPs especially when $n=500$ and $\text {MAF}=0.1$. Moreover, the PF method always has less NP than the Fieller’s method. The reason for this result is that the PF method tends to obtain shorter CIs than the Fieller’s method before the truncation [32], which benefits for the reduction of NPs since a noninformative CI is created by the truncation when $[0,\ 2]$ is totally contained by the wide original CI. Although the zero DP and lower NPs show the advantages of the PF method over the Fieller’s method, the PF method may have greater EPs when MAF is low, which is actually caused by the shorter CIs of the PF methods as well. Specifically, an empty set is created by the truncation when the original CI is disjoint from $[0,\ 2]$, which can occur when the original point estimates locate outside $[0,\ 2]$. In these cases, the shorter the CI is, the larger the probability for the original CI to be disjoint from $[0,\ 2]$ is, which causes bigger EPs of the PF method in some scenarios. It is also shown in Table 3 that the NP of the PF method as well as the NP and DP of the Fieller’s method get smaller if *n* is larger, MAF is higher or the trait changes from qualitative to quantitative. The EP of the PF method gets lower only if MAF increases while that of the Fieller’s method fluctuates irregularly throughout the simulation studies.

**Table 3:** NPs, EPs and DPs (in $\%$) for the PF and Fieller’s methods

Trait	*n*	MAF	${\rho }$	PF			Fieller		
				NP	EP	DP	NP	EP	DP
Qualitative	500	0.3	0	23.4	0.4	0.0	31.4	0.6	0.2
			− 0.05	23.6	0.4	0.0	34.6	0.8	0.0
			0.05	22.6	0.0	0.0	29.4	0.6	0.0
		0.1	0	43.2	1.6	0.0	60.2	0.2	1.0
			− 0.05	47.6	3.6	0.0	54.8	0.0	1.0
			0.05	41.0	1.4	0.0	61.0	0.8	0.4
	2000	0.3	0	0.2	0.0	0.0	0.6	0.2	0.0
			− 0.05	0.4	0.0	0.0	0.6	0.0	0.0
			0.05	0.0	0.0	0.0	0.4	0.2	0.0
		0.1	0	15.8	0.8	0.0	23.8	0.8	0.0
			− 0.05	16.8	3.2	0.0	22.2	1.2	0.6
			0.05	12.8	0.6	0.0	17.0	0.4	0.2
Quantitative	500	0.3	0	3.6	0.2	0.0	5.6	0.6	0.0
			− 0.05	3.6	0.0	0.0	6.2	0.2	0.0
			0.05	3.0	0.0	0.0	5.2	0.2	0.0
		0.1	0	23.2	5.2	0.0	26.2	3.2	0.2
			− 0.05	20.8	5.0	0.0	25.8	2.6	0.6
			0.05	22.0	1.8	0.0	26.8	1.2	0.2
	2000	0.3	0	0.0	0.0	0.0	0.0	0.0	0.0
			− 0.05	0.0	0.4	0.0	0.0	0.4	0.0
			0.05	0.0	0.2	0.0	0.0	0.2	0.0
		0.1	0	3.0	0.2	0.0	4.4	0.6	0.0
			− 0.05	5.8	0.6	0.0	9.0	1.0	0.0
			0.05	2.8	0.4	0.0	5.6	0.6	0.0

Proportions (in $\%$) of the noninformative intervals (NP), empty sets (EP) and discontinuous intervals (DP) for the penalized Fieller’s (PF) and Fieller’s methods are given under qualitative trait and quantitative trait with $(\sigma _{0}^{2},\ \sigma _{1}^{2},\ \sigma _{2}^{2})=(1,\ 1.2,\ 1)$

**Table 4:** CPs (in $\%$), $\hbox {W}_{\mathrm{mean}}$’s and $\hbox {W}_{\mathrm{median}}$’s of BN, BU, PF and Fieller’s methods

Trait	*n*	MAF	${\rho }$	CP				$\hbox {W}_{\mathrm{mean}}$				$\hbox {W}_{\mathrm{median}}$			
				BN	BU	PF	Fieller	BN	BU	PF	Fieller	BN	BU	PF	Fieller
Qualitative	500	0.3	0	93.8	95.2	95.2	95.0	1.4390	1.4643	1.5113	1.5358	1.4936	1.5353	1.5846	1.6362
			− 0.05	95.6	95.8	94.2	93.6	1.4303	1.4521	1.5007	1.5045	1.4857	1.5273	1.5724	1.5897
			0.05	94.2	95.0	95.4	95.4	1.4116	1.4336	1.4919	1.4895	1.4634	1.5004	1.5497	1.5643
		0.1	0	94.0	96.2	89.0	98.6	1.6371	1.6737	1.5841	1.8346	1.6772	1.7290	1.9183	2.0000
			− 0.05	93.2	96.2	84.6	98.8	1.6705	1.7130	1.5560	1.8369	1.6951	1.7445	1.9838	2.0000
			0.05	92.4	93.4	86.2	95.6	1.6060	1.6382	1.5602	1.7794	1.6638	1.7109	1.8887	2.0000
	2000	0.3	0	95.2	95.6	95.6	95.8	0.9647	0.9714	1.0063	1.0133	0.9654	0.9696	0.9956	1.0017
			− 0.05	94.6	95.0	95.6	95.4	0.9527	0.9571	0.9879	0.9958	0.9508	0.9492	0.9829	0.9854
			0.05	95.8	95.0	95.2	94.8	0.9451	0.9486	0.9808	0.9864	0.9599	0.9619	1.0080	1.0105
		0.1	0	93.8	95.0	94.8	95.4	1.3822	1.4041	1.4935	1.5069	1.3978	1.4251	1.5794	1.5657
			− 0.05	94.4	95.2	90.8	96.6	1.4304	1.4551	1.4554	1.5630	1.4556	1.4982	1.6570	1.6652
			0.05	95.8	96.4	96.6	97.2	1.3416	1.3602	1.4218	1.4338	1.3451	1.3737	1.4454	1.4678
Quantitative	500	0.3	0	96.0	96.4	96.2	95.8	1.1254	1.1333	1.1642	1.1741	1.1202	1.1270	1.1655	1.1715
			− 0.05	95.2	95.0	96.6	95.4	1.1289	1.1343	1.1528	1.1661	1.1248	1.1248	1.1388	1.1290
			0.05	95.6	95.6	95.8	95.4	1.1014	1.1061	1.1334	1.1455	1.0910	1.0907	1.1143	1.1135
		0.1	0	93.2	94.8	85.6	87.6	1.4866	1.5134	1.4382	1.4685	1.5045	1.5415	1.6191	1.5739
			− 0.05	94.4	95.6	81.2	88.6	1.5206	1.5505	1.3497	1.4928	1.5501	1.5844	1.5969	1.6238
			0.05	94.2	95.4	90.2	92.8	1.4616	1.4865	1.4489	1.4995	1.4926	1.5261	1.5868	1.6013
	2000	0.3	0	94.8	95.4	93.6	93.6	0.6563	0.6592	0.6694	0.6704	0.6684	0.6673	0.6792	0.6803
			− 0.05	94.6	95.8	96.2	96.2	0.6605	0.6632	0.6747	0.6760	0.6798	0.6793	0.6855	0.6877
			0.05	94.8	94.8	94.2	94.2	0.6356	0.6374	0.6508	0.6514	0.6464	0.6493	0.6613	0.6609
		0.1	0	96.8	96.6	95.8	93.4	1.1567	1.1687	1.2006	1.2012	1.1513	1.1698	1.2144	1.1945
			− 0.05	93.6	95.4	91.8	90.0	1.2586	1.2726	1.2996	1.2817	1.2726	1.2979	1.3801	1.3191
			0.05	93.8	95.4	95.0	93.6	1.1202	1.1319	1.1419	1.1616	1.1257	1.1353	1.1558	1.1859

Coverage probability (CP, in $\%$) and the mean and median of the widths of the highest posterior density intervals or confidence intervals (respectively denoted as $\hbox {W}_{\mathrm{mean}}$ and $\hbox {W}_{\mathrm{median}}$) of Bayesian method with normal prior (BN), Bayesian method with uniform prior (BU), penalized Fieller’s (PF) and Fieller’s methods among 500 replicates are given under qualitative trait and quantitative trait when $(\sigma _{0}^{2},\ \sigma _{1}^{2},\ \sigma _{2}^{2})=(1,\ 1.2,\ 1)$. The empirical CP should be between $93.05\%$ and $96.95\%$ ($0.95 \pm 2 \times \sqrt{\frac{0.95\times 0.05}{500}}$) with $95\%$ probability

From Table 4, we find that the CPs of the BN and BU methods are controlled around $95\%$ in all the simulated situations, while the CPs of the PF and Fieller’s methods are usually underestimated or overestimated when MAF is low. Moreover, we observe from Table 4 that the $\hbox {W}_{\mathrm{mean}}$’s and $\hbox {W}_{\mathrm{median}}$’s of the BN and BU methods are smaller than those of the PF and Fieller’s methods under all the scenarios. Specifically, among these four interval estimation methods, the BN method has the smallest $\hbox {W}_{\mathrm{mean}}$ in most cases and owns the least $\hbox {W}_{\mathrm{median}}$ under all the circumstances. The $\hbox {W}_{\mathrm{median}}$’s of the Fieller’s method are all 2 when $n=500$ and $\text {MAF}=0.1$ for qualitative trait, which means that more than half of the CIs obtained by the Fieller’s method are noninformative in this case. Irrespective of other factors, $\rho$ has a little effect on the $\hbox {W}_{\mathrm{mean}}$’s and $\hbox {W}_{\mathrm{median}}$’s of the four methods, which indicates that all the four methods are robust to the departure from HWE. The $\hbox {W}_{\mathrm{mean}}$’s and $\hbox {W}_{\mathrm{median}}$’s of these four methods decrease when *n* gets larger, MAF becomes higher or the trait changes from qualitative to quantitative.

In addition to the support of the findings from Table 4, Figs. 3, 4 and Additional file 2: Figs. S23–S44 present the distributions of the widths of the $95\%$ HPDIs or CIs for these four interval estimation methods against the true values of $\gamma$. Specifically, Fig. 3 gives the results with $n=500$, $\text {MAF}=0.3$ and $\rho =0$ for the qualitative trait. It is shown in Fig. 3a, b that the BN and BU methods obtain similar widths of the $95\%$ HPDIs, which are both close to 1.5. The widths of the $95\%$ CIs for the PF and Fieller’s methods shown in Fig. 3c, d are quite dispersive and a great amount of noninformative CIs (represented by red points) can be seen in these two subplots. Comparing Fig. 4 with Fig. 3, we notice that the four methods obtain shorter intervals with less variation, and the PF and Fieller’s methods have less noninformative CIs for the quantitative trait with $(\sigma _{0}^{2},\ \sigma _{1}^{2},\ \sigma _{2}^{2})=(1,\ 1.2,\ 1)$ than for the qualitative trait when $n=500$, $\text {MAF}=0.3$ and $\rho =0$. This result is true for all other simulation settings (Additional file 2: Figs.  S23–S33 vs. S34–S44). Similarly, the findings from Table 4 on the influence of *n*, MAF and $\rho$ on the widths of the $95\%$ HPDIs or CIs for the four methods are also supported by Figs. 3 and 4 and Additional file 2: Figs. S23–S44. Note that although there are plenty of noninformative CIs in the PF and Fieller’s methods when $n=500$ and $\text {MAF}=0.1$, the informative CIs of these two methods have chance to be narrower than the HPDIs of the BN and BU methods (Additional file 2: Figs. S25–S27 and S36–S38). The Fieller’s method may obtain discontinuous CIs (represented by yellow points in Fig. 3 and Additional file 2: Figs. S25–S27, S32–S33 and S36–S38), especially when $n=500$ or $\text {MAF}=0.1$ for qualitative trait. All these four interval estimation methods have their best performances with $n=2000$ and $\text {MAF}=0.3$ for quantitative trait when $(\sigma _{0}^{2},\ \sigma _{1}^{2},\ \sigma _{2}^{2})=(1,\ 1.2,\ 1)$, where the widths of the intervals of all the four methods are mostly less than 1 and tend to be smaller when the true values of $\gamma$ are close to 0 or 2 (Additional file 2: Figs. S39–S41).

The $\hbox {W}_{\mathrm{SD}}$’s and $\hbox {W}_{\mathrm{IQR}}$’s of the four methods for qualitative trait and quantitative trait with $(\sigma _{0}^{2},\ \sigma _{1}^{2},\ \sigma _{2}^{2})=(1,\ 1.2,\ 1)$ are listed in Additional file 3: Table S1 and described in Additional file 4: Text. Note that the BN method has the lowest $\hbox {W}_{\mathrm{SD}}$ and $\hbox {W}_{\mathrm{IQR}}$ among the four methods. When the variances of the quantitative trait become larger, i.e., $(\sigma _{0}^{2},\ \sigma _{1}^{2},\ \sigma _{2}^{2})=(4,\ 4.8,\ 4)$, the results are given in Additional file 3: Tables S2–S6 and Additional file 5: Figs. S45-S68. By comparing these results with those under $(\sigma _{0}^{2},\ \sigma _{1}^{2},\ \sigma _{2}^{2})=(1,\ 1.2,\ 1)$, the four point estimation methods and the four interval estimation methods generally perform worse, and even worse than those for qualitative trait. However, the Bayesian methods still have their advantages over the PF and Fieller’s methods in both the point estimation and the interval estimation.

### Application to the Graves’ disease data

According to Chu et al. [37], SNP rs3827440 within the *GPR*174 gene on X chromosome was detected to be associated with the Graves’ disease. In fact, in addition to the Graves’ disease, SNP rs3827440 was also reported to be significantly associated with the autoimmune Addison’s disease [38]. There were two stages of the association analysis in Chu et al. [37], i.e., the genome-wide association study (GWAS) stage and the replication stage. The association between SNP rs3827440 and the Graves’ disease was identified in both of two stages and the pooled data of these two stages. There are 2941 subjects (699 males and 2242 females) in the GWAS stage and 8074 subjects (1814 males and 6260 females) in the replication stage. We exclude the males and get 1115 (1127) females in the case (control) group in the GWAS stage, and 3375 (2885) females in the case (control) group in the replication stage. Note that there are two alleles *T* and *C* at rs3827440, where *T* is the deleterious allele leading to higher expression of the *GPR*174 gene. In the GWAS stage, there are respectively 163, 508 and 444 (219, 541 and 367) females with genotypes *CC*, *TC* and *TT* in the case (control) group. In the replication stage, the sample sizes of the females with genotypes *CC*, *TC* and *TT* are 471, 1606 and 1298 (584, 1344 and 957) in the case (control) group, respectively. The allele frequency of *T* in females is 0.57 in the GWAS stage and 0.56 in the replication stage.

We respectively obtain ${\hat{\gamma }}_{BN}$, ${\hat{\gamma }}_{BU}$, ${\hat{\gamma }}_{PF}$ and ${\hat{\gamma }}_{F}$, and derive the corresponding intervals with the BN, BU, PF and Fieller’s methods based on the data in the GWAS stage and replication stage without considering any covariate, and apply these methods to the pooled data by regarding stage as a covariate [30]. The hyperparameters in the Bayesian methods are set to be the same as those in the Methods section and we choose $N(0, 10^{2})$ as the prior distribution of the effect size of the stage. The point estimates and the corresponding $95\%$ HPDIs or CIs of $\gamma$ at SNP rs3827440 are given in Table 5. From Table 5, we find that the results of the Fieller’s method we get are consistent with those in Wang et al. [30].The HPDIs or CIs obtained by these four interval estimation methods do not contain 1 in the replication stage and the pooled data, which suggests XCI-S at rs3827440. In the replication stage, the four point estimates are all close to 1.5, which indicates XCI-S towards allele *C*, and about $75\%$ (1.5/2) cells in a heterozygous female have allele *T* active at this locus. The four point estimates are all close to 1.37 in the pooled data, which suggests XCI-S towards allele *C*, with allele *T* active in about $68.50\%$ (1.37/2) cells in a heterozygous female at rs3827440. Note that all the HPDIs or CIs in the GWAS stage contain 1, which indicates XCI-R or XCI-E. This difference may be caused by the heterogeneity of the data in these two stages. Furthermore, the BN method always has the shortest interval among the four methods, which highlights its advantage.

**Table 5:** Application to the Graves’ disease data at SNP rs3827440

Stage	Point estimate				${95\%}$ HPDI or CI			
	${{\hat{\gamma }}_{BN}}$	${{\hat{\gamma }}_{BU}}$	${{\hat{\gamma }}_{PF}}$	${{\hat{\gamma }}_{F}}$	BN	BU	PF	Fieller
GWAS	0.9835	0.9890	0.9537	0.9567	(0.2092, 1.6248)	(0.1524, 1.6359)	(0.0241, 1.6441)	[0, 1.6579)
Replication	1.4804	1.5193	1.5120	1.5126	(1.1144, 1.8855)	(1.1274, 1.9064)	(1.1226, 1.9270)	(1.1224, 1.9299)
Pooled	1.3693	1.3770	1.3724	1.3727	(1.0206, 1.7134)	(1.0272, 1.7368)	(1.0280, 1.7184)	(1.0277, 1.7195)

BN, Bayesian method with normal prior; BU, Bayesian method with uniform prior; PF, penalized Fieller’s method

### Application to the MCTFR data

The Minnesota Center for Twin and Family Research Genome-Wide Association Study of Behavioral Disinhibition from the database of Genotypes and Phenotypes is a large, ongoing and family-based epidemiological study of substance abuse and related psychopathology with 2183 families, including 7377 participants (3546 males and 3831 females). Among them, 5960 participants have both phenotypic data and genotypic data while the others only have phenotypic data. There are five quantitative traits: the nicotine composite score, the alcohol consumption composite score (CON), the alcohol dependence composite score (DEP), the illicit drug composite score and the behavioral disinhibition composite score (BD) in the dataset. To avoid family structure and population structure, we exclude all the offspring in the dataset. Because we only need the information of females, we also exclude males in the dataset. Eventually, we get 1998 female individuals. There are 12,354 SNPs genotyped on X chromosome in the dataset. We use the standard quality control procedures [39] as follows. Firstly, we exclude those female individuals with missing genotype rate over $10\%$. Secondly, we delete those SNPs with missing rate over $10\%$. Thirdly, we exclude those SNPs whose MAF is less than $5\%$. Finally, we conduct the HWE tests for the remaining SNPs with the PLINK software (version 1.90) [39] and set the significance level to be $1 \times 10^{-4}$ [40], and those SNPs out of HWE are also excluded. After the quality control procedures, we include 1996 female individuals with 11,344 SNPs on X chromosome in this application.

Note that all the point estimation methods (${\hat{\gamma }}_{BN}$, ${\hat{\gamma }}_{BU}$, ${\hat{\gamma }}_{PF}$ and ${\hat{\gamma }}_{F}$) and the interval estimation methods (BN, BU, PF and Fieller) mentioned above require the presence of association between the X-chromosomal SNP and the trait under study. So, the association analysis for each SNP and each trait in the MCTFR dataset is required before we apply these methods to measure the degree of XCI-S. We use linear regression to test for the association by including the age as a covariate. However, we notice that all the residuals derived from the regressions of the five quantitative traits do not satisfy the normality assumption. So, we use the association tests based on the direct inverse normal transformation (D-INT), the indirect inverse normal transformation (I-INT) and the adaptive omnibus test (O-INT) proposed by McCaw et al. [41]. The significance level of the association tests is set to be $4.408 \times 10^{-6}\ (0.05/11344)$ after the Bonferroni correction. We then select the SNPs with at least one of the three *P* values of D-INT, I-INT and O-INT is less than $4.408 \times 10^{-6}$. After obtaining the associated SNPs, we calculate the point estimates (${\hat{\gamma }}_{BN}$, ${\hat{\gamma }}_{BU}$, ${\hat{\gamma }}_{PF}$ and ${\hat{\gamma }}_{F}$) of $\gamma$, and use the BN, BU, PF and Fieller’s methods to derive the corresponding intervals of $\gamma$ for these SNPs, respectively. Since the methods proposed in this article require the normality of the trait, each trait is first regressed on the age to obtain the residuals, and the inverse normal transformation is respectively applied to the residuals of the five quantitative traits which can be treated as new outcomes to measure the degree of XCI-S [41]. The hyperparameters in the Bayesian methods are set to be the same as those in the Methods section.

There are four SNPs (rs331318, rs5928558, rs10522027 and rs12849233) associated with the DEP trait, six SNPs (rs12557060, rs3008896, rs5961051, rs4489437, rs2097322 and rs463233) associated with the BD trait and one SNP (rs4240042) associated with the CON trait. The positions, the alleles, the MAFs, the *P* values of the HWE tests and three association tests (D-INT, I-INT and O-INT) together with the related traits and the genes of these associated SNPs are presented in Table 6.

**Table 6:** SNPs detected in association analysis for the MCTFR data

SNP	Position	Allele		MAF	*P* value				Trait	Gene
		Minor	Major		HWE test	D-INT	I-INT	O-INT		
rs12557060	28772570	G	A	0.4737	0.1781	$3.883\times 10^{-6}$	$4.087\times 10^{-3}$	$7.759\times 10^{-6}$	BD	$IL1RAPL1^a$
rs331318	32234387	G	A	0.4847	0.6868	$5.163\times 10^{-7}$	$7.768\times 10^{-4}$	$1.032\times 10^{-6}$	DEP	$DMD^b$
rs5928558	34363004	G	A	0.1844	0.0529	$4.225\times 10^{-6}$	$8.921\times 10^{-5}$	$8.069\times 10^{-6}$	DEP	
rs10522027	34558201	A	G	0.1405	0.1642	$1.490\times 10^{-8}$	$3.094\times 10^{-6}$	$2.967\times 10^{-8}$	DEP	$TMEM47^c$
rs3008896	39632138	A	G	0.4654	0.7873	$3.474\times 10^{-6}$	$4.060\times 10^{-3}$	$6.942\times 10^{-6}$	BD	
rs4240042	39748679	G	A	0.3953	0.9627	$1.208\times 10^{-6}$	$1.352\times 10^{-3}$	$2.414\times 10^{-6}$	CON	
rs5961051	53906442	C	A	0.4216	0.0810	$3.541\times 10^{-6}$	$4.563\times 10^{-3}$	$7.077\times 10^{-6}$	BD	
rs4489437	124515139	G	A	0.4481	0.9639	$2.140\times 10^{-7}$	$5.599\times 10^{-4}$	$4.277\times 10^{-7}$	BD	
rs2097322	124523716	A	C	0.4020	0.6416	$2.369\times 10^{-6}$	$2.042\times 10^{-3}$	$4.732\times 10^{-6}$	BD	
rs463233	149839444	G	A	0.4800	0.5601	$4.164\times 10^{-6}$	$5.817\times 10^{-3}$	$8.321\times 10^{-6}$	BD	
rs12849233	150564832	A	C	0.3296	0.7611	$1.360\times 10^{-7}$	$2.196\times 10^{-4}$	$2.718\times 10^{-7}$	DEP	$PASD1^d$

The significance level of the three association tests (D-INT, I-INT and O-INT) is set to be $4.408\times 10^{-6}$;

$^{\rm a}$This gene is cited by Walker et al. [42];

$^{\rm b}$This gene is cited by Miyagoe-Suzuki et al. [43];

$^{\rm c}$This gene is cited by Ng et al. [44];

$^{\rm d}$This gene is cited by Li et al. [45]

Among these SNPs, rs12557060 is within the gene interleukin-1 receptor accessory protein-like 1 (*IL*1*RAPL*1) [42] and SNP rs331318 is located in the gene Duchenne muscular dystrophy (*DMD*) [43]. It was reported that *IL*1*RAPL*1 and *DMD* are two large genes located immediately adjacent to each other within the common fragile site region of instability, which are active in normal brain tissue but are under-expressed in every brain tumor cell line and xenograft [46]. The disruption or deletion of the *IL*1*RAPL*1 gene is found to be associated with the BD trait in our association analysis whose disruption or deletion was previously detected in individuals with mental retardation and/or autism spectrum disorder [42]. According to Miyagoe-Suzuki et al. [43], the *DMD* gene encodes the dystrophin protein required for the stability of the sarcolemma and the mutations of *DMD* may cause X-linked Duchenne muscular dystrophy. Miyagoe-Suzuki et al. [43] also found that many induce pluripotent stem clones derived from a manifesting female carrier of *DMD* had two active X chromosomes or mixed XCI patterns, which means that the *DMD* gene may escape from XCI or undergo different XCI patterns within different female subgroups. SNP rs10522027 is within the gene transmembrane protein 47 (*TMEM*47), which may be a useful biomarker for predicting the response to chemotherapy and a potential therapeutic target for overcoming hepatocellular carcinoma cell chemoresistance [44]. SNP rs12849233 is in PAS domain containing repressor 1 (*PASD*1), which might possibly serve as a new target for the prognosis and the future treatment of glioma [45].

**Table 7:** Application of BN, BU, PF and Fieller’s methods for SNPs detected in association analysis

SNP	Point estimate				${95\%}$ HPDI or CI			
	${{\hat{\gamma }}_{BN}}$	${{\hat{\gamma }}_{BU}}$	${{\hat{\gamma }}_{PF}}$	${{\hat{\gamma }}_{F}}$	BN	BU	PF	Fieller
rs12557060	0.9234	0.9227	0.9303	0.9386	(0.1500, 1.8080)	(0.0872, 1.8216)	[0, 2]	[0, 2]
rs331318	1.2233	1.2198	1.2586	1.2651	(0.5205, 1.9704)	(0.5313, 1.9988)	(0.4235, 2]	(0.4088, 2]
rs5928558	0.9251	0.9119	0.9744	0.9837	(0.3847, 1.8414)	(0.4016, 1.9173)	(0.3452, 2]	(0.3629, 2]
rs10522027	0.7243	0.6869	0.7595	0.7651	(0.2828, 1.6562)	(0.2689, 1.7268)	(0.2937, 1.8982)	(0.3067, 2]
rs3008896	0.5524	0.4300	0.3920	0.3950	(0.0006, 1.3040)	(0, 1.3026)	[0, 1.3264)	[0, 1.3655)
rs4240042	1.0378	1.0244	1.0648	1.0698	(0.3815, 1.8557)	(0.3893, 1.9351)	(0.3326, 2]	(0.3277, 2]
rs5961051	1.1732	1.2490	1.2863	1.3050	(0.4478, 1.9992)	(0.4118, 2)	(0.2700, 2]	(0.2401, 2]
rs4489437	1.5543	1.6712	1.7697	1.7802	(0.9453, 1.9999)	(0.9708, 2)	(0.9175, 2]	(0.9296, 2]
rs2097322	1.6407	1.7820	1.9987	2.0000	(0.8910, 2)	(0.9109, 2)	(0.8958, 2]	(0.9353, 2]
rs463233	0.3859	0.1586	0.1715	0.1728	(0, 1.1054)	(0, 1.0898)	[0, 1.0713)	[0, 1.0847)
rs12849233	0.6330	0.6580	0.6500	0.6525	(0.0551, 1.4351)	(0.0187, 1.4338)	(0.0201, 1.4681)	(0.0099, 1.5358)

BN, Bayesian method with normal prior; BU, Bayesian method with uniform prior; PF, penalized Fieller’s method

The point estimates and the corresponding $95\%$ HPDIs or CIs of $\gamma$ for these SNPs are given in Table 7. Note that the CIs of the PF and Fieller’s methods are obtained by truncating the original CI into $[0,\ 2]$. As a result, some CIs of these two methods have the left endpoints equal to 0 or the right endpoints equal to 2, while the HPDIs of the BN and BU methods will generally be an open interval, and the left (right) endpoints of the HPDIs are generally larger (less) than 0 (2). Although the $95\%$ HPDIs or CIs of the SNPs all contain 1, which is indicative of the XCI-R or XCI-E pattern, we can still observe the advantage of the BN and BU methods that they generally get shorter intervals than the PF and Fieller’s methods. On the other hand, notice that the HPDIs and CIs for SNPs rs4489437, rs2097322 and rs463233 are strongly asymmetrical and the corresponding point estimates (1.5543, 1.6712, 1.7697 and 1.7802 for SNP rs4489437; 1.6407, 1.7820, 1.9987 and 2.0000 for SNP rs2097322, and 0.3859, 0.1586, 0.1715 and 0.1728 for SNP rs463233) are either all greater than 1.5 or all smaller than 0.5. So, this may give a clue that these three SNPs are possible to undergo XCI-S, which needs to be further confirmed by, for example, larger sample sizes or molecular genetics.

## Discussion

**Fig. 5:**
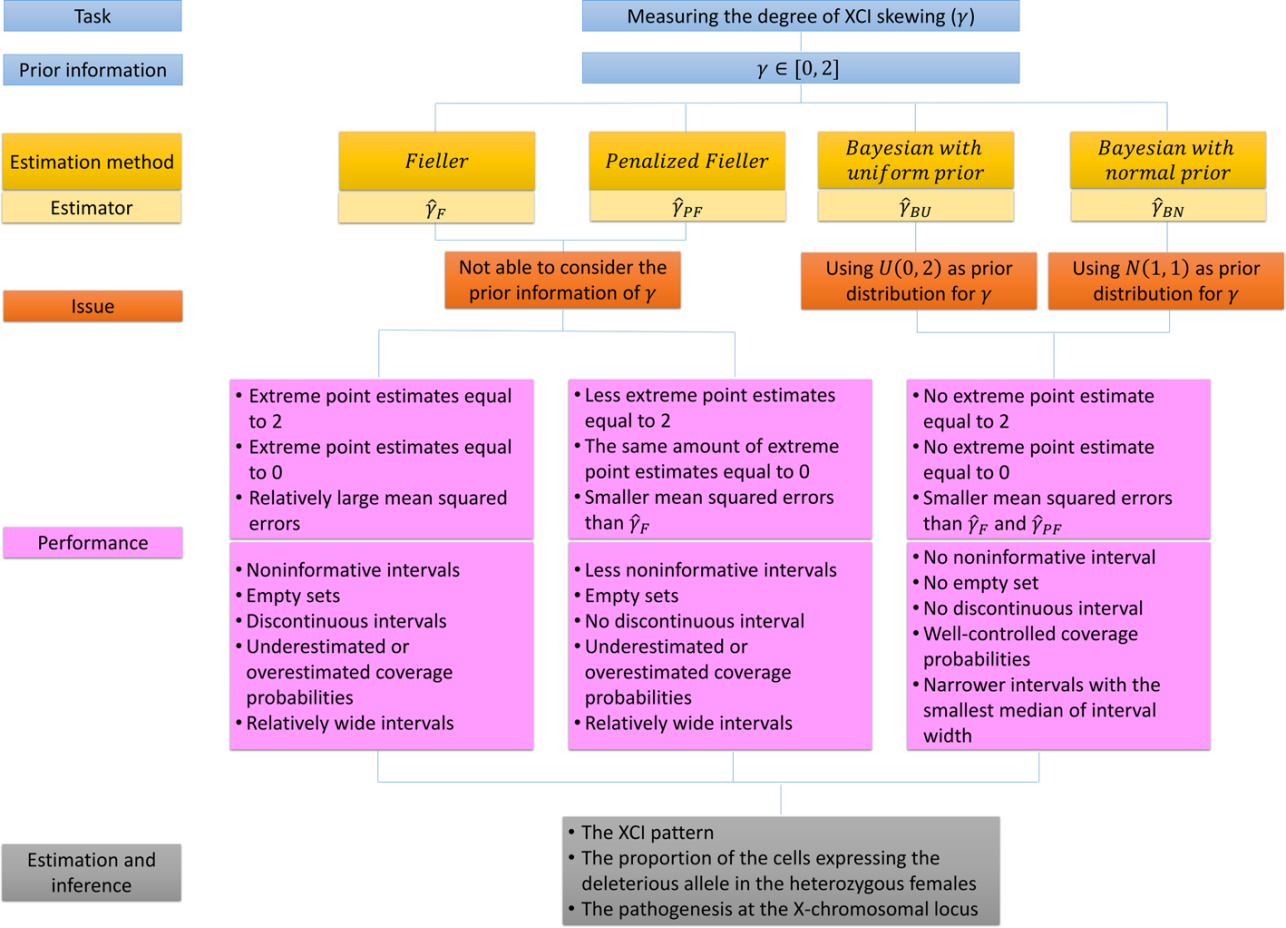
Framework of the estimation methods for the degree of XCI-S. (1) Task: the four methods aim at measuring the degree of XCI-S; (2) prior information: the constraint condition of the degree of XCI-S; (3) estimation method and estimator: the four estimation methods and the notations of their corresponding point estimates; (4) issue: the main issue of the method; (5) performance: the performances of the four estimation methods in point estimation and interval estimation; (6) estimation and inference: the estimation results and inferences obtained by the methods

In this article, we proposed a Bayesian method to obtain the point estimate and the credible interval of the degree of XCI-S ($\gamma$) by incorporating its prior information. We calculated the mode and the HPDI of the samples of $\gamma$ as the point estimate and the credible interval for $\gamma$, respectively. In fact, we also used the median and the percentile interval (the 2.5th and 97.5th percentiles of the width of interval) of the samples as the point estimate and the credible interval of $\gamma$. However, their performances are worse than the mode and the HPDI (data not shown) and hence we chose the latter instead. We considered a normal prior and a uniform prior for the degree of XCI-S in the Bayesian method, which are respectively denoted as the BN and BU methods. We also derived a penalized point estimate ${\hat{\gamma }}_{PF}$ based on the idea of the PF method and obtained its corresponding CI [32]. We compared the proposed ${\hat{\gamma }}_{BN}$, ${\hat{\gamma }}_{BU}$ and ${\hat{\gamma }}_{PF}$ with the existing point estimate ${\hat{\gamma }}_{F}$, and investigated the performances of the BN, BU, PF and Fieller’s methods in the interval estimation for both the qualitative and quantitative traits via extensive simulation studies. The framework of these four estimation methods is illustrated in Fig. 5. As summarized in Fig. 5, there is no extreme value (0 or 2) to occur for ${\hat{\gamma }}_{BN}$ and ${\hat{\gamma }}_{BU}$ while the extreme point estimates can be found in both ${\hat{\gamma }}_{PF}$ and ${\hat{\gamma }}_{F}$ under all the scenarios. Besides, the BN and BU methods can solve the problems of noninformative intervals, empty sets and discontinuous intervals which can be found in the Fieller’s method, while the PF method can only avoid the discontinuous CIs to occur. Note that the extreme point estimate 0 (2) means that $100\%$ of the cells have the deleterious (normal) allele inactivated at a SNP, which is not a common case in reality. On the other hand, it is hard for us to identify the XCI pattern with the noninformative CIs and the discontinuous CIs, and the empty sets even provide no information on the XCI pattern. These facts highlight the advantages of the BN and BU methods that they can avoid the occurrence of the extreme point estimates and guarantee continuous HPDIs to provide useful information on the XCI pattern all the time. Further, among these four point estimation methods, ${\hat{\gamma }}_{BN}$ has the smallest MSE under all the simulated situations. In interval estimation, the CPs of the BN and BU methods are generally controlled around $95\%$ while the CPs of the PF and Fieller’s methods are usually underestimated or overestimated when MAF is low. The BN method has the smallest $\hbox {W}_{\mathrm{mean}}$ in most of the cases and the lowest $\hbox {W}_{\mathrm{median}}$ and the least $\hbox {W}_{\mathrm{SD}}$ and $\hbox {W}_{\mathrm{IQR}}$ under all the circumstances. Hence, we recommend the BN method in practice for its robustness and accuracy in both point estimation and interval estimation.

We applied the four point estimation methods and the four interval estimation methods to the Graves’ disease data and the MCTFR data for their practical use on the qualitative trait and the quantitative trait, respectively. In the Graves’ disease data application, we found that SNP rs3827440 may undergo the XCI-S pattern towards the allele *C* in the replication stage and the pooled data. Although we did not detect the XCI-S pattern in the GWAS stage, the BN and BU methods still show their superiority by providing shorter HPDIs, compared to the PF and Fieller’s methods. In the MCTFR data application, the $95\%$ HPDIs and CIs of the SNPs all contain 1, which indicates the XCI-R or XCI-E pattern. However, we also found three suspectable SNPs rs4489437, rs2097322 and rs463233 which may undergo the XCI-S pattern based on their extremely asymmetrical HPDIs and CIs. Since the inverse normal transformation applied to the original traits may lead to the loss of the information in the four interval estimation methods, we expect shorter intervals of these three SNPs that do not contain 1 if we have larger samples or a normally distributed trait. However, these conclusions need to be further confirmed by molecular genetics.

On the other hand, in our simulation study, we did not incorporate any covariate. To further investigate the performances of the four point estimates and the four interval estimation methods with a covariate, here we conducted additional simulation studies by considering a covariate under HWE (i.e., $\rho =0$). The simulation settings can be found in Additional file 4: Text, and the simulation results are listed in Additional file 3: Tables S7–S11 and Additional file 5: Figs. S69–S84 and described in Additional file 4: Text, respectively. From these results, we observed that although the performances of all the proposed methods under the scenarios with a covariate are worse than those without any covariate, the trends are similar to those in the Results section, and the Bayesian methods also show their own advantages over the PF and Fieller’s methods.

The last but not least, the proposed methods have the following issues to discuss. Firstly, the prior distributions of the unknown parameters are required in the Bayesian methods and the choice of them may have influence on the results. We considered two prior distributions for $\gamma$, $U(0,\ 2)$ is a noninformative prior that should have little impact on the posterior distribution, and $N(1,\ 1) \in [0,\ 2]$ is chosen based on its own genetic background. We also considered weakly informative priors for each of the unknown parameters other than $\gamma$, which should be robust to different kinds of parameters. The researchers can choose the priors based on their own study background or refer to the priors used in this article if they have limited knowledge of the distributions of the parameters. Secondly, although we assume that all the unknown parameters are independent of each other in the Bayesian method because the Hamiltonian Monte Carlo (HMC) algorithm used for sampling in the Bayesian method does not greatly suffer from the correlated parameters, we expect better performance of the Bayesian method by considering the correlations between the unknown parameters and regard it as our future work. Thirdly, the HPDI or CI containing 1 indicates the XCI-R or XCI-E pattern at the SNP. How to further distinguish between the XCI-R and XCI-E patterns is our future work. On the other hand, note that there is an assumption that the underlying genetic model is additive to guarantee that the estimated $\gamma$ value departing from 1 indicates the XCI-S rather than the non-additive models, such as the genotypic values $X_i=\{0, 2, 2\}$ for the dominant model and $X_i=\{0, 0, 2\}$ for the recessive model. It is also true that $\gamma$ can be greater than 2 or less than 0 in the situations of the overdominance and the underdominance, respectively. It may not be possible to distinguish a non-additive model from the XCI-S by considering the estimation of $\gamma$ simply based on the mean effects of a generalized linear regression model. However, the variance-based tests may be alternative [19, 21], which is our future work. However, it should be noted that Dobyns et al. recommended discontinuing the use of the terms “X-linked dominant inheritance” and “X-linked recessive inheritance” because both are incomplete and fail to explain some aspects of the X-linked inheritance due to some biological mechanisms including cell autonomy or non-autonomy of the gene product, XCI status and mosaicism [47, 48]. Fourthly, the normality assumption of quantitative traits is required for all the methods we discussed in this article. In future, we will extend the methods to accommodate the traits which do not follow a normal distribution. Finally, all the methods are only applicable to unrelated female subjects. Thus, we will extend the methods and make them suitable for data with family structure in future studies.

## Conclusion

In summary, the existing point estimate and the existing Fieller’s method cannot consider the prior information of the degree of XCI-S, and respectively have the problems of the extreme point estimates (0 or 2) and the noninformative CIs, empty sets as well as discontinuous CIs. To solve these problems, we proposed a penalized point estimate and obtained its CI with the PF method to make an improvement, and proposed two Bayesian methods (BN and BU) to incorporate the prior information of the degree of XCI-S by using a normal prior or a uniform prior for the degree of XCI-S in the model. We recommend the Bayesian methods in practice because it can avoid obtaining the extreme point estimates and guarantee continuous HPDIs to provide useful information on the XCI pattern all the time. The BN method can also provide point estimates with the smallest MSE and HPDIs with well controlled CP, the shortest width and the lowest variation across all the simulation settings. In the real data application, we found that SNP rs3827440 in the Graves’ disease data may undergo XCI-S towards the allele *C*, which need to be confirmed by molecular genetics.

## Methods

### Notations

To detect the SNPs undergoing XCI-S and measure their degree of XCI-S, we focus on females because only females can provide the information on XCI-S. Assume that *n* females are sequenced at a candidate diallelic SNP on X chromosome, where *d* (*D*) is the normal (deleterious) allele. Then, for female *i*, the genotypes $G_{i}=\left\{ dd,\ Dd,\ DD\right\}$ and the corresponding genotypic values $X_{i}=\left\{ 0,\ \gamma ,\ 2\right\}$, $i=1,2,\cdots ,n$, where $\gamma \in [0,\ 2]$ represents the degree of XCI-S. Let ${\varvec{Z}}_{i}$ be a $M\times 1$ covariates vector and $Y_{i}$ be the trait, which can be either qualitative or quantitative. As such, the following generalized linear regression model is used to describe the association between $G_{i}$ and $Y_{i}$,

1 $$\begin{aligned} h\left( E \left( Y_{i}|X_{i},{\varvec{Z}}_{i}\right) \right) =\beta _{0}+\beta X_{i}+{\varvec{b}}^{T}{\varvec{Z}}_{i} \end{aligned}$$

where $\beta _{0}$ is the intercept and $\beta$ is the regression coefficient for $X_{i}$. ${\varvec{b}}$ is a $M\times 1$ vector of the regression coefficients for ${\varvec{Z}}_{i}$. $E(Y_{i}|X_{i},{\varvec{Z}}_{i})$ is the conditional expected value of $Y_{i}$ given $X_{i}$ and ${\varvec{Z}}_{i}$, and $h(\bullet )$ is a link function. When $Y_{i}$ is a qualitative trait, $h(\bullet )$ is the logit function. Then, Eq. (1) can be written as

$$\begin{aligned} \mathrm{Logit}\left( P\left( Y_{i}=1\right) \right) =\beta _{0} + \beta X_{i}+{\varvec{b}}^{T}{\varvec{Z}}_{i} \end{aligned}$$

where $Y_{i}$ is the disease status of female *i*, and $Y_{i}=1\ (0)$ denotes that female *i* is affected (unaffected). When $Y_{i}$ is a quantitative trait, $h(\bullet )$ is the identity function, and $Y_{i}$ has a random error $\varepsilon _{i}$. In this case, Equation (1) becomes

2 $$\begin{aligned} Y_{i}=\beta _{0}+\beta X_{i}+{\varvec{b}}^{T}{\varvec{Z}}_{i}+\varepsilon _{i} \end{aligned}$$

where $\varepsilon _{i}\sim N(0,\sigma _{0}^{2}I_{\{G_{i}=dd\}}+\sigma _{1}^{2}I_{\{G_{i}=Dd\}}+\sigma _{2}^{2}I_{\{G_{i}=DD\}})$ and $I(\bullet )$ is the indicator function. According to Ma et al. [19], the variance of the quantitative trait for heterozygous females may be higher than those for homozygous females, i.e., $\sigma _{1}^{2}$ may be greater than $\sigma _{0}^{2}$ and $\sigma _{2}^{2}$.

The genotypic value $X_{i}$ can be decomposed into $X_{1i}$ and $X_{2i}$ according to Wang et al. [30], i.e., $X_{i}=\gamma X_{1i}+(2-\gamma )X_{2i}$, where $X_{1i}$ and $X_{2i}$ are two indicator variables. $X_{1i}=I_{\{G_{i}=Dd\ \text {or}\ DD\}}$ indicates if female *i* has at least one deleterious allele *D* and $X_{2i}=I_{\{G_{i}=DD\}}$ denotes if female *i* has two deleterious alleles *D*. So, Eq. (1) can be re-expressed as

3 $$\begin{aligned} h\left( E \left( Y_{i}|X_{i},{\varvec{Z}}_{i} \right) \right) = \beta _{0} + \beta \gamma X_{1i} + \beta (2-\gamma )X_{2i}+{\varvec{b}}^{T}{\varvec{Z}}_{i} \end{aligned}$$

Let $\beta _{1} =\beta \gamma \ \mathop {\mathrm{and}} \ \beta _{2} =\beta (2-\gamma )$. So, $\gamma =\frac{\beta _{1}}{\beta }$ and $\beta =\frac{\beta _{1}+\beta _{2}}{2}$. Equation (3) turns to be

$$\begin{aligned} h\left( E \left( Y_{i}|X_{i},{\varvec{Z}}_{i} \right) \right) = \beta _{0} + \beta _{1} X_{1i} + \beta _{2} X_{2i}+{\varvec{b}}^{T}{\varvec{Z}}_{i} \end{aligned}$$

After respectively obtaining the maximum likelihood estimates ${\hat{\beta }}_{0}, {\hat{\beta }}_{1}, {\hat{\beta }}_{2}$ and $\varvec{\hat{b}}$ of $\beta _{0}, \beta _{1}, \beta _{2}$ and ${\varvec{b}}$, we have ${\hat{\beta }}=\frac{{\hat{\beta }}_{1}+{\hat{\beta }}_{2}}{2}$. Assume that $v_{1}$, $v_{2}$ and $v_{12}$ are respectively the variance of ${\hat{\beta }}_{1}$, the variance of ${\hat{\beta }}$ and the covariance of ${\hat{\beta }}_{1}$ and ${\hat{\beta }}$. To derive $\hat{v}_{1}$, $\hat{v}_{2}$ and $\hat{v}_{12}$, the empirical Fisher information matrix is used for qualitative traits [30] and the *glm* function in R software is applied for quantitative traits [31].

### Existing point estimate and CI of $\varvec{\gamma }$ by Fieller’s method

Here, we recall the existing point estimate and the corresponding CI obtained by the Fieller’s method [30, 31]. The existing point estimate of $\gamma$ can be given as a ratio of two regression coefficients

4 $$\begin{aligned} {\hat{\gamma }}_{origin}=\frac{{\hat{\beta }}_{1}}{{\hat{\beta }}}=\frac{2{\hat{\beta }}_{1}}{{\hat{\beta }}_{1}+{\hat{\beta }}_{2}} \end{aligned}$$

Since $\gamma$ represents the degree of XCI-S, which should be within $[0,\ 2]$, the final point estimate can be derived by cutting the ${{\hat{\gamma }}}_{origin}$ in Eq. (4) into $[0,\ 2]$. So, we have ${\hat{\gamma }}_{F}=\frac{2{\hat{\beta }}_{1}}{{\hat{\beta }}_{1}+{\hat{\beta }}_{2}} \cap [0,\ 2].$

To obtain the corresponding CI of $\gamma$ by the Fieller’s method, a Wald test can be built to test for $H_{0}:\gamma =\gamma _{0}$. Since $\gamma$ can be expressed as $\gamma =\frac{\beta _{1}}{\beta }$, we have

5 $$\begin{aligned} \begin{aligned} \frac{{\hat{\beta }}_{1}-\gamma _{0}{\hat{\beta }}}{\sqrt{\hat{v}_{1}+\gamma _{0}^{2}\hat{v}_{2}-2\gamma _{0}\hat{v}_{12}}}=z_{1-\alpha /2} \end{aligned} \end{aligned}$$

where $z_{1-\alpha /2}$ is the $(1-\alpha /2)$ upper quantile of a standard normal distribution when the sample size is large enough. Rearranging Equation (5), we get a quadratic equation with respect to $\gamma _{0}$ as follows

6 $$\begin{aligned} A\gamma _{0}^{2}+B\gamma _{0}+C=0 \end{aligned}$$

where $A={\hat{\beta }}^{2} -z_{1-\alpha /2}^{2} \hat{v}_{2}$, $B=2(z_{1-\alpha /2}^{2} \hat{v}_{12}-{\hat{\beta }}_{1} {\hat{\beta }})$ and $C={\hat{\beta }}_{1}^{2}-z_{1-\alpha /2}^{2} \hat{v}_1$. From Eq. (6), we have $\Delta =B^{2}-4AC$, and $A>0$ implies $\Delta >0$. If $\Delta >0$, we can obtain two roots $\gamma _{F}^{L}=\frac{-B- \sqrt{\Delta }}{2A}$ and $\gamma _{F}^{U}=\frac{-B+ \sqrt{\Delta }}{2A}$ of Equation (6) as the confidence limits with $\gamma _{F}^{L} < \gamma _{F}^{U}$. As mentioned above, the original CI should be truncated into $[0,\ 2]$ because $\gamma \in [0,\ 2]$. Then, the CI of the Fieller’s method can be summarized as follows

$$\begin{aligned} \left\{ \begin{matrix} (\gamma _{F}^{L}, \gamma _{F}^{U})\cap [0,\ 2], &{} \ A>0 &{}\ \\ \left( (-\infty ,\gamma _{F}^{L})\cup ( \gamma _{F}^{U}, \infty )\right) \cap [0,\ 2],&{} \ A<0 \ \text {and}\ \Delta >0\\ \left[ 0,\ 2\right] , &{}{\ A<0 \ \text {and}\ \Delta <0}\\ \varnothing ,&{} \ A=0 \ \text {or} \ \Delta =0 &{}\ \end{matrix} \right. \end{aligned}$$

where Ø is the empty set. We call $[0,\ 2]$ the noninformative interval and $\left( (-\infty ,\gamma _{F}^{L})\cup ( \gamma _{F}^{U}, \infty )\right) \cap [0,\ 2]$ may be discontinuous.

### Penalized point estimate and CI of $\varvec{\gamma }$ by PF method

Here, we propose a penalized point estimate and obtain its corresponding CI by the PF method [32]. Notice that if the denominator ${\hat{\beta }}=\frac{{\hat{\beta }}_{1}+{{\hat{\beta }}}_{2}}{2}$ of ${\hat{\gamma }}_{origin}$ is not statistically significantly different from 0, then ${\hat{\gamma }}_{origin}$ will tend to be infinite (mainly positive infinite because ${\hat{\beta }}_{1}$ and ${\hat{\beta }}_{2}$ usually have the same sign according to Wang et al. [30]) and the corresponding CI of the Fieller’s method before the truncation will tend to be unbounded, which is the common case if the denominator ${\hat{\beta }}$ has a large variance. To solve this problem, Wang et al. [32] proposed the PF method to reduce the variance of the denominator of a ratio estimate by imposing a penalty on it and adjusting the numerator accordingly. Borrowing this idea, we define a penalized log-likelihood function of $\beta$ as

7 $$\begin{aligned} pl=-\frac{({\hat{\beta }}-\beta )^{2}}{2{\hat{v}}_{2}}+\lambda \log \left| \beta \right| \end{aligned}$$

where $\lambda >0$ is the penalty parameter. Maximizing the log-likelihood function (7), we obtain the penalized denominator $\beta ^{*}={\hat{\beta }}/2+\mathrm{sign}({\hat{\beta }})\sqrt{{\hat{\beta }}^{2}/4+\lambda \hat{v}_{2}}$, where $\mathrm{sign}(\bullet )$ is the signum function [32]. Making a Taylor expansion for $\beta ^{*}$ around ${\hat{\beta }}$ and $\hat{v}_{2}$, we get $Var(\beta ^{*})=v^{*}_{2}=\omega ^{2} \hat{v}_{2}+O(n^{-3})$ and $Cov({\hat{\beta }}_{1},\beta ^{*})=\omega \hat{v}_{12}+O(n^{-3})$, where $\omega =\frac{\beta ^{*}}{2\beta ^{*}-{\hat{\beta }}}$.

According to Wang et al. [32], if we simply replace ${\hat{\beta }}$ by $\beta ^{*}$ in ${\hat{\gamma }}_{origin}=\frac{{\hat{\beta }}_{1}}{{\hat{\beta }}}$, we will get a biased estimate of $\gamma$. To reduce the bias caused by the penalized denominator, we need to further adjust the numerator ${\hat{\beta }}_{1}$ by $\beta ^{*}_{1}={\hat{\beta }}_{1}+{\tilde{\gamma }}(\beta ^{*}-{\hat{\beta }})$, where ${\tilde{\gamma }}=\frac{{\hat{\beta }}_{1}}{\beta ^{*}}$. Making a Taylor expansion for $\beta ^{*}_{1}$ around ${\hat{\beta }}_{1}$ and ${\hat{\beta }}$, we have $Var(\beta ^{*}_1)=v^{*}_{1}=\omega ^{-2}\hat{v}_{1}-4(\omega ^{-1}-1){\tilde{\gamma }}\hat{v}_{12}+4(1-\omega )^{2} {\tilde{\gamma }}^{2} \hat{v}_{2}$ and $Cov(\beta ^{*}_{1},\beta ^{*})=v^{*}_{12}=\hat{v}_{12}-2\omega (1-\omega ){\tilde{\gamma }}\hat{v}_{2}$. As such, the original penalized point estimate is ${\hat{\gamma }}^{*}_{origin}=\frac{{\hat{\beta }}^{*}_{1}}{{\hat{\beta }}^{*}}$. Although we may avoid the situation of the denominator approaching 0, ${\hat{\gamma }}^{*}_{origin}$ may still be out of $[0,\ 2]$. Therefore, we need to cut ${\hat{\gamma }}^{*}_{origin}$ into $[0,\ 2]$ and get the final penalized point estimate as follows,

$$\begin{aligned} {\hat{\gamma }}_{PF}=\frac{{\hat{\beta }}^{*}_{1}}{{\hat{\beta }}^{*}} \cap [0,\ 2] \end{aligned}$$

For the construction of the corresponding CI of ${\hat{\gamma }}_{PF}$, the PF method uses the same theory as the Fieller’s method. So, we only need to respectively replace ${\hat{\beta }}, {\hat{\beta }}_{1}, \hat{v}_{1}, \hat{v}_{2}$ and $\hat{v}_{12}$ by $\beta ^{*}, \beta ^{*}_1, v^{*}_{1}, v^{*}_{2}$ and $v^{*}_{12}$ in Eqs. (5) and (6) and choose an appropriate penalty parameter $\lambda$ for the PF method to get the penalized CI. From Wang et al. [32], we know that when $\lambda \ge \frac{z_{1-\alpha /2}^{2}}{4}$, the PF method can always produce a bounded CI. But when $\lambda \rightarrow \infty$, the width of the CI will tend to be 0 and the CP will also tend to be 0. So, we select $\lambda = \frac{z_{1-\alpha /2}^{2}}{4}$, which enables the PF method to produce a bounded CI and control the CP at the same time. However, although the PF method can always get a bounded CI when $\lambda =\frac{z_{1-\alpha /2}^{2}}{4}$, the CI may still be out of $[0,\ 2]$ and needs to be cut off in $[0,\ 2]$.

The point estimates and CIs of the Fieller’s and PF methods we discussed above are not able to include the prior information that $\gamma \in [0,\ 2]$ in the model. By contrast, the Bayesian approach can flexibly incorporate this prior information into the analysis.

### Point estimate and credible interval of $\varvec{\gamma }$ by Bayesian method

Bayesian method has been widely used in genetic analysis in recent years [35] and various algorithms such as HMC [36] make sampling from the parameters’ approximate posterior distributions possible even if the analytical solutions of those posterior distributions are not available. Assume that $\varvec{\theta _{\cdot }}$ represents $\varvec{\theta _{d}}$ (the unknown parameters for qualitative trait) or $\varvec{\theta _{c}}$ (the unknown parameters for quantitative trait). For the qualitative trait, we suppose that $Y_{i}$ follows a Bernoulli distribution, i.e.,

$$\begin{aligned} Y_{i}\sim B(p_{i}) \end{aligned}$$

where $p_{i}=\frac{1}{1+\mathrm{exp}\left( -(\beta _{0}+\beta \gamma X_{1i} + \beta (2-\gamma )X_{2i}+{\varvec{b}}^{T}{\varvec{Z}}_{i})\right) }$. In this case, the unknown parameters $\varvec{\theta _{d}}=(\beta _{0},\beta ,\gamma ,{\varvec{b}}^{T})^{T}$. For the quantitative trait, we assume that $Y_{i}$ is normally distributed, i.e.,

$$\begin{aligned} Y_{i} \sim N(\mu _{i},\sigma _{0}^{2} I_{\{G_{i}=dd\}}+\sigma _{1}^{2} I_{\{G_{i}=Dd\}}+\sigma _{2}^{2} I_{\{G_{i}=DD\}}) \end{aligned}$$

where $\mu _{i}= \beta _{0} + \beta \gamma X_{1i} + \beta (2-\gamma ) X_{2i}+{\varvec{b}}^{T} {\varvec{Z}}_{i}$. In this case, the unknown parameters $\varvec{\theta _{c}}$=($\beta _{0},\beta ,\gamma$, ${\varvec{b}}^{T},\sigma _{0},\sigma _{1},\sigma _{2})^{T}$. Let ${\varvec{Y}}=(Y_{1},Y_{2},\cdots ,Y_{n})^{T}$ and ${\varvec{D}}=({\varvec{X}}_{1},{\varvec{X}}_{2},{\varvec{Z}})$, where ${\varvec{X}}_{1}=(X_{11}, X_{12},\cdots , X_{1n})^{T}$, ${\varvec{X}}_{2}=(X_{21}, X_{22}$, $\cdots , X_{2n})^{T}$ and ${\varvec{Z}}=({\varvec{Z}}_{1},{\varvec{Z}}_{2}$ $,\cdots ,{\varvec{Z}}_{n})^{T}$. Then, the posterior distribution of $\varvec{\theta _{d}}$ or $\varvec{\theta _{c}}$ is

$$\begin{aligned} f(\varvec{\theta _{\cdot }}|{\varvec{Y}},{\varvec{D}}) = \frac{f(\varvec{\theta _{\cdot }})f({\varvec{Y}}|{\varvec{D}}, \varvec{\theta _{\cdot }})}{f({\varvec{Y}}|{\varvec{D}})} \end{aligned}$$

where $f(\varvec{\theta _{\cdot }})$ is the joint prior distribution of $\varvec{\theta _{\cdot }}$. $f({\varvec{Y}}|{\varvec{D}}, \varvec{\theta _{\cdot }})$ is the likelihood function of ${\varvec{Y}}$. $f({\varvec{Y}}|{\varvec{D}})$ is the conditional probability density function of ${\varvec{Y}}$ given ${\varvec{D}}$, i.e., $f({\varvec{Y}}|{\varvec{D}})=\int f({\varvec{Y}}|{\varvec{D}},\varvec{\theta _{\cdot }})f(\varvec{\theta _{\cdot }})d\varvec{\theta _{\cdot }}$. We find that $f(\varvec{\theta _{\cdot }}|{\varvec{Y}},{\varvec{D}})$ is hard to calculate, which means that the closed form of the posterior distribution of $\varvec{\theta _{\cdot }}$ is difficult for us to obtain. So, instead of directly computing their posterior distributions of $\varvec{\theta _{\cdot }}$, we use the HMC algorithm (e.g., the *rstan* package in R) to sample the parameters from the approximate posterior distribution. We choose the HMC algorithm because it can improve the independence of the samples and has higher efficiency than the other Markov-Chain Monte Carlo methods.

The HMC algorithm requires the prior distributions of $\gamma$ and the other parameters in $\varvec{\theta _{\cdot }}$. Since HMC does not dramatically suffer from the correlated parameters in model, we assume that the unknown parameters are independent of each other for simplicity [36]. Then, $f(\varvec{\theta _{\cdot }})$ can be given as $f(\varvec{\theta _{\cdot }})=\prod _{g=1}^{\theta _{\cdot }^{\#}} f(\theta _{\cdot g})$, where $\theta _{\cdot }^{\#}$ is the number of the parameters in $\varvec{\theta _{\cdot }}$, and $f(\theta _{\cdot g})$ is the prior distribution of the *g*th parameter in $\varvec{\theta _{\cdot }}$.

Since the value of $\gamma$ should be between 0 and 2, we consider a uniform distribution on $[0,\ 2]$ as the prior distribution for $\gamma$, i.e., $\gamma \sim U(0,\ 2)$, which is a noninformative prior. In addition, we also consider a normal prior distribution for $\gamma$ which is truncated into $[0,\ 2]$, i.e., $\gamma \sim N(1,\ 1) \in [0,\ 2]$. As such, not only $\gamma$ satisfies the constraint condition of $\gamma \in [0,\ 2]$, but also the probability of $\gamma$ being close to 1 is the highest, which is consistent with the literature [4], i.e., most of the SNPs on X chromosome undergo the XCI-R. Besides, the truncated normal distribution of $\gamma$ keep the probability of $\gamma$ taking the extreme value (0 or 2) not too low, which may be more suitable for practical applications. Further, the 1-sigma criterion of $N(1,\ 1)$ is $(1-1,\ 1+1)$, i.e., $(0,\ 2)$. As for $\beta _{0}$, $\beta$ and ${\varvec{b}}$ in both $\varvec{\theta _{d}}$ and $\varvec{\theta _{c}}$, we consider weak priors that enable us to obtain negative and positive effects as well as strong and weak effects [49]. Specifically, $\beta _{0}\sim N(\mu _{\beta _{0}}, \sigma ^{2}_{\beta _{0}})$, $\beta \sim N(\mu _{\beta },\sigma ^{2}_{\beta })$ and ${\varvec{b}}\sim \textit{MVN} \left( \varvec{\mu _{b}}, \sum \ \right)$, where $\varvec{\mu _{b}}=(\mu _{b_{1}}, \mu _{b_{2}},\cdots ,\mu _{b_{M}})^{T}$ is a $M \times 1$ mean vector and $\sum$ is a $M \times M$ variance-covariance matrix of ${\varvec{b}}$. In this article, we set $\mu _{\beta _{0}}=0$, $\mu _{\beta }=0$, $\varvec{\mu _{b}}=(0,0,...,0)^{T}_{M \times 1}$, $\sigma _{\beta _{0}}^{2}=10^2$ and $\sigma _{\beta }^2=10^2$, and let $\sum$ be a symmetric matrix with diagonal elements being $10^2$ and non-diagonal elements being 0.

When it comes to quantitative traits, we need to provide the prior distributions for $\sigma _{0}$, $\sigma _{1}$ and $\sigma _{2}$ additionally and sample them respectively because the variances of the quantitative trait may be different across different genotypes in females according to Ma et al. [19]. We also choose a weakly informative prior for $\sigma _{j}$ (*j*=0, 1, 2), which is an exponential distribution with the mean being 1 [36], i.e., $\sigma _{j} \sim \mathrm{exp}(a_{j})$ (*j*=0, 1, 2), where $a_{0}$, $a_{1}$ and $a_{2}$ are the hyperparameters needed to be pre-defined and are all set to be 1 in this article. The hyperparameters $\mu _{\beta _{0}}$, $\mu _{\beta }$, $\varvec{\mu _{b}}$, $\sigma _{\beta _{0}}^{2}$, $\sigma _{\beta }^2$, $\sum$, $a_{0}$, $a_{1}$ and $a_{2}$ can also be selected based on the research background or experience.

Once the likelihood function of ${\varvec{Y}}$ and the prior distributions of the parameters in $\varvec{\theta _{\cdot }}$ are provided, we can obtain as many samples of $\varvec{\theta _{\cdot }}$ as we want by the HMC algorithm. After getting enough samples of $\varvec{\theta _{\cdot }}$, we calculate the mode and the HPDI of the samples of $\gamma$ as the point estimate and the credible interval for $\gamma$, respectively.

### Simulation settings

Since males provide no information on XCI-S, we only include females in simulation studies. We consider the qualitative trait and the quantitative trait, respectively. For simplicity, we do not include any covariate in the simulation.

For the qualitative trait, according to Wang et al. [30], we set the frequencies of genotypes *dd*, *Dd* and *DD* in the control (case) group to be $g_{0}$, $g_{1}$ and $g_{2}$ ($c_{0}$, $c_{1}$ and $c_{2}$), respectively. Assume that the frequency of the deleterious allele *D* is *p* in the control group, which is usually the MAF at the SNP considered. Assume that the frequency of the normal allele *d* in the control group is *q* $(p+q=1)$. As such, we have $(g_{0},\, g_{1},\, g_{2})=(q^2+\rho pq ,\, 2(1-\rho )pq,\, p^2+\rho pq)$, where $\rho$ is the inbreeding coefficient. In our simulation, MAF is fixed at 0.3 and 0.1, and $\rho$ is set to be 0, -0.05 and 0.05. We define $\lambda _{1}$ and $\lambda _{2}$ as the odds ratios for genotypes *Dd* and *DD* compared to genotype *dd* in females, respectively. Then, we have $\lambda _{1}=\mathrm{exp}(\beta \gamma )$ and $\lambda _{2}=\mathrm{exp}(2\beta )$. Notice that $\lambda _1=\lambda _2^{\gamma /2}$ and $\gamma =2\mathrm{ln}(\lambda _1) / \mathrm{ln}(\lambda _2)$. Fixing $\lambda _{2}=2$ and randomly sampling $\gamma$ from *U*(0, 2), we can calculate $\beta$ and $\lambda _{1}$. So, we have $\frac{c_{0}}{g_{0}}=\mathrm{exp}(\beta _{0}),\ \frac{c_{1}}{g_{1}}=\lambda _1 \mathrm{exp}(\beta _{0}),$ and $\frac{c_{2}}{g_{2}}=\lambda _{2} \mathrm{exp}(\beta _{0}).$ With $c_{0}+ c_{1}+c_{2}=1$, we can calculate ($c_{0}$, $c_{1}$, $c_{2}$) and $\beta _{0}$ from the values of ($g_{0}$, $g_{1}$, $g_{2}$), $\lambda _{1}$ and $\lambda _{2}$. Then, we generate the samples of three genotypes for the control group and the case group by the trinomial distributions with probabilities ($g_{0}$, $g_{1}$, $g_{2}$) and ($c_{0}$, $c_{1}$, $c_{2}$), respectively. Finally, we can accordingly get $X_{1i}$ and $X_{2i}$ for all the females. Further, we assume that the case-control ratio is 1 : 1, with the sample size $n=500$ and 2000.

For the quantitative trait, let $g_{0}$, $g_{1}$ and $g_{2}$ respectively represent the frequencies of genotypes *dd*, *Dd* and *DD*. Then, we simulate the sample size $n_{0}$, $n_{1}$ and $n_2$ ($n_{0}+n_{1}+n_{2}=n$) for genotypes *dd*, *Dd* and *DD* from a trinomial distribution with probabilities ($g_{0}$, $g_{1}$, $g_{2}$) by fixing *n* at 500 and 2,000. As such, we can get $X_{1i}$ and $X_{2i}$ accordingly for female *i*, $i=1,2,\cdots ,n$. $Y_{i}$ is generated by $Y_{i} \sim N(\mu _{i}, \sigma _{0}^{2}I_{\{G_{i}=dd\}}+\sigma _{1}^{2}I_{\{G_{i}=Dd\}}+\sigma _{2}^{2}I_{\{G_{i}=DD\}})$ with $\mu _{i}=\beta _{0} + \beta \gamma X_{1i} + \beta (2-\gamma )X_{2i}$, where $\beta _{0}$ is set to be 0, $\beta$ is set to be 0.3 and the underlying $\gamma$ value is randomly sampled from $U(0,\ 2)$. As mentioned above, the variance of the quantitative trait for heterozygous females $(\sigma _{1}^{2})$ may be generally larger than those for homozygous females ($\sigma _{0}^{2}$ and $\sigma _{2}^{2}$) [19]. So, we consider two scenarios and set $(\sigma _{0}^{2},\ \sigma _{1}^{2},\ \sigma _{2}^{2})=(1,\ 1.2,\ 1)$ and $(\sigma _{0}^{2},\ \sigma _{1}^{2},\ \sigma _{2}^{2})=(4,\ 4.8,\ 4)$. For each simulation setting, we conduct 500 replicates (i.e., 500 SNPs) and the confidence level $(1-\alpha )$ is fixed at $95\%$ for the frequentist methods. To make the HPDIs comparable to the CIs, we calculate $95\%$ HPDIs for the Bayesian methods.

In the Bayesian methods, the prior distributions of $\gamma$, $\beta _{0}$, $\beta$ and $\sigma _{j}$ (*j*=0, 1, 2) are set as we mentioned in the Methods section, i.e., $\gamma \sim U(0,\ 2)$ and $\gamma \sim N(1,\ 1) \in [0,\ 2]$, $\beta _{0} \sim N(0,\ 10^{2})$, $\beta \sim N(0,\ 10^{2})$ and $\sigma _{j} \sim \mathrm{exp}(1)$. We set 8 chains to extract the samples parallelly and simultaneously. We extract 20,000 samples in each chain, among which the first 10,000 samples are only used for warming up and are discarded when the sampling is finished. So eventually, we get 80,000 samples in total. The target acceptance rate is set to be 0.99 to ensure the convergence. The convergence diagnostic $\hat{R}$ for Markov chains in the Bayesian method is done, which compares the between-chain and within-chain estimates for the model parameters. If the chains have not mixed well (i.e., the between-chain and within-chain estimates do not agree with each other), the $\hat{R}$ of the convergence diagnostic will be larger than 1. Note that the calculated $\hat{R}$’s in our Bayesian models are all less than 1.05 which indicates good convergence (data not shown). The simulation study is implemented by the R software (version 4.0.0).

## Supplementary information

**Additional file 1. Figs. S1-S22.** Scatter plots of point estimates of γ against true value of γ for qualitative trait and quantitative trait when (σ_0_^2^, σ_1_^2^, σ_2_^2^)=(1, 1.2, 1) with n=500 and 2000, MAF=0.3 and 0.1, and ρ=0, -0.05 and 0.05, respectively.

**Additional file 2. Figs. S23-S44.** Widths of HPDIs or CIs against true value of γ for qualitative trait and quantitative trait when (σ_0_^2^, σ_1_^2^, σ_2_^2^)=(1, 1.2, 1) with n=500 and 2000, MAF=0.3 and 0.1, and ρ=0, -0.05 and 0.05, respectively.

**Additional file 3. Table S1.** W_SD_'s and W_IQR_'s of BN, BU, PF and Fieller's methods for qualitative trait and quantitative trait with (σ_0_^2^, σ_1_^2^, σ_2_^2^)=(1, 1.2, 1). **Table S2**. Proportions of extreme values of ${\hat{\gamma }}_{PF}$ and ${\hat{\gamma }}_{F}$ for quantitative trait when (σ_0_^2^, σ_1_^2^, σ_2_^2^)=(4, 4.8, 4). **Table S3.** MSEs of ${\hat{\gamma }}_{BN}$, ${\hat{\gamma }}_{BU}$, ${\hat{\gamma }}_{PF}$ and ${\hat{\gamma }}_{F}$ for quantitative trait when (σ_0_^2^, σ_1_^2^, σ_2_^2^)=(4, 4.8, 4). **Table S4**. NPs, EPs and DPs for PF and Fieller's methods for quantitative trait when (σ_0_^2^, σ_1_^2^, σ_2_^2^)=(4, 4.8, 4). **Table S5**. CPs of BN, BU, PF and Fieller's methods for quantitative trait when (σ_0_^2^, σ_1_^2^, σ_2_^2^)=(4, 4.8, 4). **Table S6**. W_mean_'s, W_median_'s, W_SD_'s and W_IQR_'s of BN, BU, PF and Fieller's methods for quantitative trait when (σ_0_^2^, σ_1_^2^, σ_2_^2^)=(4, 4.8, 4). **Table S7**. Proportions of extreme values of ${\hat{\gamma }}_{PF}$ and ${\hat{\gamma }}_{F}$ for qualitative trait and quantitative trait when (σ_0_^2^, σ_1_^2^, σ_2_^2^)=(1, 1.2, 1) with a covariate and ρ=0. **Table S8**. MSEs of ${\hat{\gamma }}_{BN}$, ${\hat{\gamma }}_{BU}$, ${\hat{\gamma }}_{PF}$ and ${\hat{\gamma }}_{F}$ for qualitative trait and quantitative trait when (σ_0_^2^, σ_1_^2^, σ_2_^2^)=(1, 1.2, 1) with a covariate and ρ=0. **Table S9**. NPs, EPs and DPs for PF and Fieller's methods for qualitative trait and quantitative trait when (σ_0_^2^, σ_1_^2^, σ_2_^2^)=(1, 1.2, 1) with a covariate and ρ=0. **Table S10**. CPs, Wmean's and Wmedian's of BN, BU, PF and Fieller's methods for qualitative trait and quantitative trait when (σ_0_^2^, σ_1_^2^, σ_2_^2^)=(1, 1.2, 1) with a covariate and ρ=0. **Table S11**. W_SD_'s and W_IQR_'s of BN, BU, PF and Fieller's methods for qualitative trait and quantitative trait when (σ_0_^2^, σ_1_^2^, σ_2_^2^)=(1, 1.2, 1) with a covariate and ρ=0.

**Additional file 4.** Text--Simulation results of W_SD_ and W_IQR_, simulation settings with a covariate, and simulation results with a covariate.

**Additional file 5. Figs. S45-S56.** Scatter plots of point estimates of γ against true value of γ for quantitative trait when (σ_0_^2^, σ_1_^2^, σ_2_^2^)=(4, 4.8, 4) with n=500 and 2000, MAF=0.3 and 0.1, and ρ=0, -0.05 and 0.05, respectively. **Figs. S57-S68.** Widths of HPDIs or CIs against true value of γ for quantitative trait when (σ_0_^2^, σ_1_^2^, σ_2_^2^)=(4, 4.8, 4) with n=500 and 2000, MAF=0.3 and 0.1, and ρ=0, -0.05 and 0.05, respectively. **Figs. S69-S76**. Scatter plots of point estimates of γ against true value of γ for qualitative trait and quantitative trait when (σ_0_^2^, σ_1_^2^, σ_2_^2^)=(1, 1.2, 1) with a covariate when n=500 and 2000, MAF=0.3 and 0.1, and ρ=0, respectively. **Figs. S77-S84**. Widths of HPDIs or CIs against true value of γ for qualitative trait and quantitative trait when (σ_0_^2^, σ_1_^2^, σ_2_^2^)=(1, 1.2, 1) with a covariate when n=500 and 2000, MAF=0.3 and 0.1, and ρ=0, respectively.

## Data Availability

The R package for the BN, BU, PF and Fieller’s methods is freely available on GitHub (https://github.com/Wen-YiYu/BEXCIS). The Graves’ disease data can be found in Chu et al. [37]. The Minnesota Center for Twin and Family Research data supporting the conclusions of this article is available in the database of Genotypes and Phenotypes repository (accession numbers 86747-6 and 95621-5), https://www.ncbi.nlm.nih.gov/projects/gap/cgi-bin/study.cgi?study_id=phs000620.v1.p1.

## Abbreviations

BD: Behavioral disinhibition composite score
BEXCIS: Bayesian methods for estimating the degree of the skewness of X chromosome inactivation
BN: Bayesian method with the normal prior for the degree of the skewness of X chromosome inactivation
BU: Bayesian method with the uniform prior for the degree of the skewness of X chromosome inactivation
CI: Confidence interval
CON: Alcohol consumption composite score
CP: Coverage probability
DEP: Alcohol dependence composite score
D-INT: Direct inverse normal transformation
DP: Proportion of the discontinuous confidence interval
EP: Proportion of the empty set
GWAS: Genome-wide association study
HMC: Hamiltonian Monte Carlo
HPDI: Highest posterior density interval
HWE: Hardy–Weinberg equilibrium
I-INT: Indirect inverse normal transformation
LR: Likelihood ratio
MAF: Minor allele frequency
MCTFR: Minnesota Center for Twin and Family Research
MSE: Mean squared error
NP: Proportion of the noninformative confidence interval
O-INT: Adaptive omnibus test
PF: Penalized Fieller’s method
SNP: Single nucleotide polymorphism
XCI: X chromosome inactivation
XCI-E: Escape from X chromosome inactivation
XCI-R: Random X chromosome inactivation
XCI-S: Skewed X chromosome inactivation

## References

[1] Lyon M (1961). Gene action in the X-chromosome of the mouse (*Mus musculus* L.). Nature.

[2] Lyon M (1962). Sex chromatin and gene action in the mammalian X-chromosome. Am J Hum Genet.

[3] Zito A, Davies MN, Tsai PC, Roberts S, Andres-Ejarque R, Nardone S (2019). Heritability of skewed X-inactivation in female twins is tissue-specific and associated with age. Nat Commun.

[4] Amos-Landgraf JM, Cottle A, Plenge RM, Friez M, Schwartz CE, Longshore J (2006). X chromosome-inactivation patterns of 1,005 phenotypically unaffected females. Am J Hum Genet.

[5] Peeters SB, Cotton AM, Brown CJ (2014). Variable escape from X-chromosome inactivation: identifying factors that tip the scales towards expression. BioEssays.

[6] Posynick BJ, Brown CJ (2019). Escape from X-chromosome inactivation: an evolutionary perspective. Front Cell Dev Biol.

[7] Deng XX, Berletch JB, Nguyen DK, Disteche CM (2014). X chromosome regulation: diverse patterns in development, tissues and disease. Nat Rev Genet.

[8] Medema RH, Burgering BMT (2007). The X factor: skewing X inactivation towards cancer. Cell.

[9] Shvetsova E, Sofronova A, Monajemi R, Gagalova K, Draisma HHM, White SJ (2019). Skewed X-inactivation is common in the general female population. Eur J Hum Genet.

[10] Vladan B, Vesna M, Elka S, Ana B, Radoslav D, Lada Z (2014). Skewed X-chromosome inactivation in women affected by Alzheimer’s disease. J Alzheimers Dis.

[11] Zheng G, Joo JN, Zhang C, Geller NL (2007). Testing association for markers on the X chromosome. Genet Epidemiol.

[12] Clayton D (2008). Testing for association on the X chromosome. Biostatistics.

[13] Wang J, Yu R, Shete S (2014). X-chromosome genetic association test accounting for X-inactivation, skewed X-inactivation, and escape from X-inactivation. Genet Epidemiol.

[14] Chen Z, Ng HKT, Li J, Liu Q, Huang H (2017). Detecting associated single-nucleotide polymorphisms on the X chromosome in case control genome-wide association studies. Stat Methods Med Res.

[15] Wang P, Xu SQ, Wang BQ, Fung WK, Zhou JY (2019). A robust and powerful test for case–control genetic association study on X chromosome. Stat Methods Med Res.

[16] Liu W, Wang BQ, Liu-Fu GJ, Fung WK, Zhou JY (2019). X-chromosome genetic association test incorporating X-chromosome inactivation and imprinting effects. J Genet.

[17] Zhang Y, Xu SQ, Liu W, Fung WK, Zhou JY (2020). A robust test for X-chromosome genetic association accounting for X-chromosome inactivation and imprinting. Genet Res.

[18] Zhang L, Martin ER, Morris RW, Li YJ (2009). Association test for X-linked QTL in family-based designs. Am J Hum Genet.

[19] Ma L, Hoffman G, Keinan A (2015). X-inactivation informs variance-based testing for X-linked association of a quantitative trait. BMC Genom.

[20] Gao F, Chang D, Biddanda A, Ma L, Guo YJ, Zhou ZL (2015). XWAS: a software toolset for genetic data analysis and association studies of the X chromosome. J Hered.

[21] Deng WQ, Mao S, Kalnapenkis A, Esko T, Sun L (2019). Analytical strategies to include the X-chromosome in variance heterogeneity analyses: evidence for trait-specific polygenic variance structure. Genet Epidemiol.

[22] Özbalkan Z, Baǧışlar S, Kiraz S, Akyerli CB, Özer HTE, Yavuz Ş (2005). Skewed X chromosome inactivation in blood cells of women with scleroderma. Arthritis Rheum.

[23] Chabchoub G, Uz E, Maalej A, Mustafa CA, Rebai A, Mnif M (2009). Analysis of skewed X-chromosome inactivation in females with rheumatoid arthritis and autoimmune thyroid diseases. Arthritis Res Ther.

[24] Kristiansen M, Langerød A, Knudsen GP, Weber BL, Børresen-Dale AL, Ørstavik KH (2002). High frequency of skewed X inactivation in young breast cancer patients. J Med Genet.

[25] Buller RE, Sood AK, Lallas T, Buekers T, Skilling JS (1999). Association between nonrandom X-chromosome inactivation and BRCA1 mutation in germline DNA of patients with ovarian cancer. J Natl Cancer I.

[26] Puck JM, Nussbaum RL, Conley ME (1987). Carrier detection in X-linked severe combined immunodeficiency based on patterns of X chromosome inactivation. J Clin Invest.

[27] Migeon BM, Moser HW, Moser AB, Axelman J, Sillence D, Norum RA (1981). Adrenoleukodystrophy: evidence for X linkage, inactivation, and selection favoring the mutant allele in heterozygous cells. Proc Natl Acad Sci USA.

[28] Plenge R, Stevenson R, Lubs H, Schwartz C, Willard H (2002). Skewed X-chromosome inactivation is a common feature of X-linked mental retardation disorders. Am J Hum Genet.

[29] Xu SQ, Zhang Y, Wang P, Liu W, Wu XB, Zhou JY (2018). A statistical measure for the skewness of X chromosome inactivation based on family trios. BMC Genet.

[30] Wang P, Zhang Y, Wang BQ, Li JL, Wang YX, Pan DD (2019). A statistical measure for the skewness of X chromosome inactivation based on case–control design. BMC Bioinform.

[31] Li BH, Yu WY, Zhou JY (2021). A statistical measure for the skewness of X chromosome inactivation for quantitative traits and its application to the MCTFR data. BMC Genom Data.

[32] Wang P, Xu SQ, Wang YX, Wu BL, Fung WK, Gao GM (2021). Penalized Fieller’s confidence interval for the ratio of bivariate normal means. Biometrics..

[33] Hoff PD, Casella G (2009). A first course in Bayesian statistical methods. Springer texts in statistics.

[34] Spiegelhalter DJ, Abrams KR, Myles JP. Bayesian Approaches to Clinical Trials and Health-Care Evaluation. New Jersey, USA: John Wiley & Sons, Inc; 2004.

[35] Stephens M, Balding DJ (2009). Bayesian statistical methods for genetic association studies. Nat Rev Genet.

[36] Annis J, Miller BJ, Palmeri TJ (2017). Bayesian inference with Stan: a tutorial on adding custom distributions. Behav Res Methods.

[37] Chu X, Shen M, Xie F, Miao XJ, Shou WH, Liu L (2013). An X chromosome-wide association analysis identifies variants in GPR174 as a risk factor for Graves’ disease. J Med Genet.

[38] Napier C, Mitchell AL, Gan E, Wilson I, Pearce SHS (2015). Role of the X-linked gene GPR174 in autoimmune Addison’s disease. J Clin Endocrinol Metab.

[39] Purcell S, Neale B, Todd-Brown K, Thomas L, Ferreira MAR, Bender D (2007). PLINK: a tool set for whole-genome association and population-based linkage analyses. Am J Hum Genet.

[40] Chung RH, Ma D, Wang K, Hedges DJ, Jaworski JM, Gilbert JR (2011). An X chromosome-wide association study in autism families identifies TBL1X as a novel autism spectrum disorder candidate gene in males. Mol Autism.

[41] McCaw ZR, Lane JM, Saxena R, Redline S, Lin XH (2020). Operating characteristics of the rank-based inverse normal transformation for quantitative trait analysis in genome-wide association studies. Biometrics.

[42] Walker RM, Sussmann JE, Whalley HC, Ryan NM, Porteous DJ, McIntosh AM (2016). Preliminary assessment of pre-morbid DNA methylation in individuals at high genetic risk of mood disorders. Bipolar Disord.

[43] Miyagoe-Suzuki Y, Nishiyama T, Nakamura M, Narita A, Takemura F, Masuda S (2017). Induction of pluripotent stem cells from a manifesting carrier of Duchenne muscular dystrophy and characterization of their X-inactivation status. Stem Cells Int..

[44] Ng KTP, Yeung OWH, Liu J, Li CX, Liu H, Liu XB (2020). Clinical significance and functional role of transmembrane protein 47 (TMEM47) in chemoresistance of hepatocellular carcinoma. Int J Oncol.

[45] Li RY, Guo MG, Song LJ (2019). PAS Domain Containing Repressor 1 (PASD1) promotes glioma cell proliferation through inhibiting apoptosis in vitro. Med Sci Monit.

[46] McAvoy S, Ganapathiraju S, Perez DS, James CD, Smith DI (2007). DMD and IL1RAPL1: two large adjacent genes localized within a common fragile site (FRAXC) have reduced expression in cultured brain tumors. Cytogenet Genome Res.

[47] Dobyns WB, Filauro A, Tomson BN, Chan AS, Ho AW, Ting NT (2004). Inheritance of most X-linked traits is not dominant or recessive, just X-linked. Am J Med Genet A.

[48] Dobyns WB (2006). The pattern of inheritance of X-linked traits is not dominant or recessive, just X-linked. Acta Paediatr.

[49] Agresti A. An Introduction to Categorical Data Analysis. New Jersey, USA: John Wiley & Sons, Inc; 2019.

