# Supplementary material for: BEXCIS: Bayesian methods for estimating the degree of the skewness of X chromosome inactivation
Source: BMC Bioinformatics. 2022 May 24;23:193. doi: 10.1186/s12859-022-04721-y (PMC9128296; doi:10.1186/s12859-022-04721-y)
Supplement: Supplementary file 1 — Additional file 1. Figs. S1-S22. Scatter plots of point estimates of γ against true value of γ for qualitative trait and quantitative trait when (σ02, σ12, σ22)=(1, 1.2, 1) with n=500 and 2000, MAF=0.3 and 0.1, and ρ=0, -0.05 and 0.05, respectively. [file 12859_2022_4721_MOESM1_ESM.pdf]

Additional file 1: Supplementary Figures S1–S22

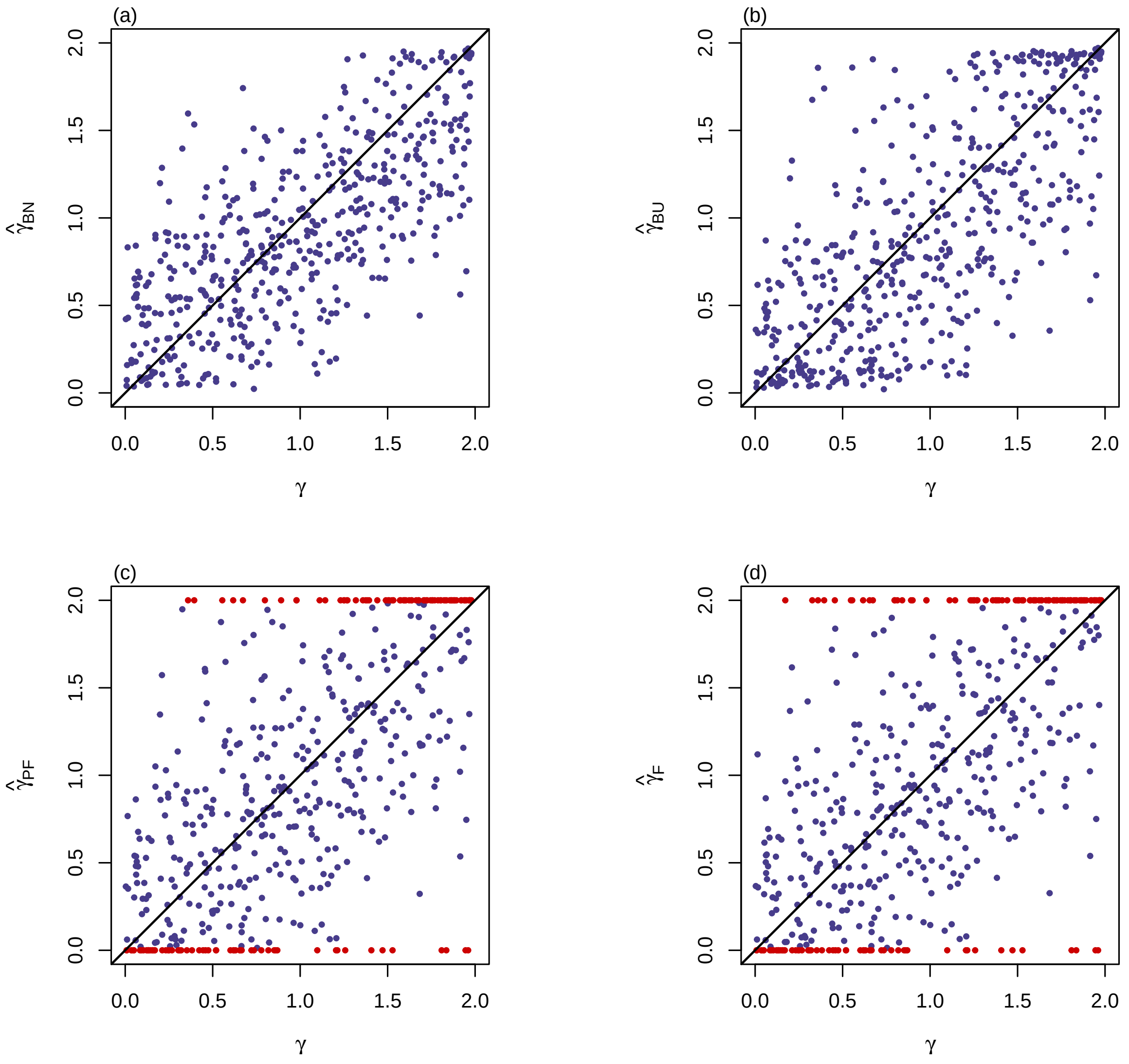

Supplementary Figure S1 Scatter plots of point estimates of γ against true value of γ for qualitative trait with $n = 500$, MAF $= 0.3$ and $\rho = -0.05$. The red points represent the extreme values (0 or 2). **(a)** $\hat{\gamma}_{BN}$; **(b)** $\hat{\gamma}_{BU}$; **(c)** $\hat{\gamma}_{PF}$; **(d)** $\hat{\gamma}_F$

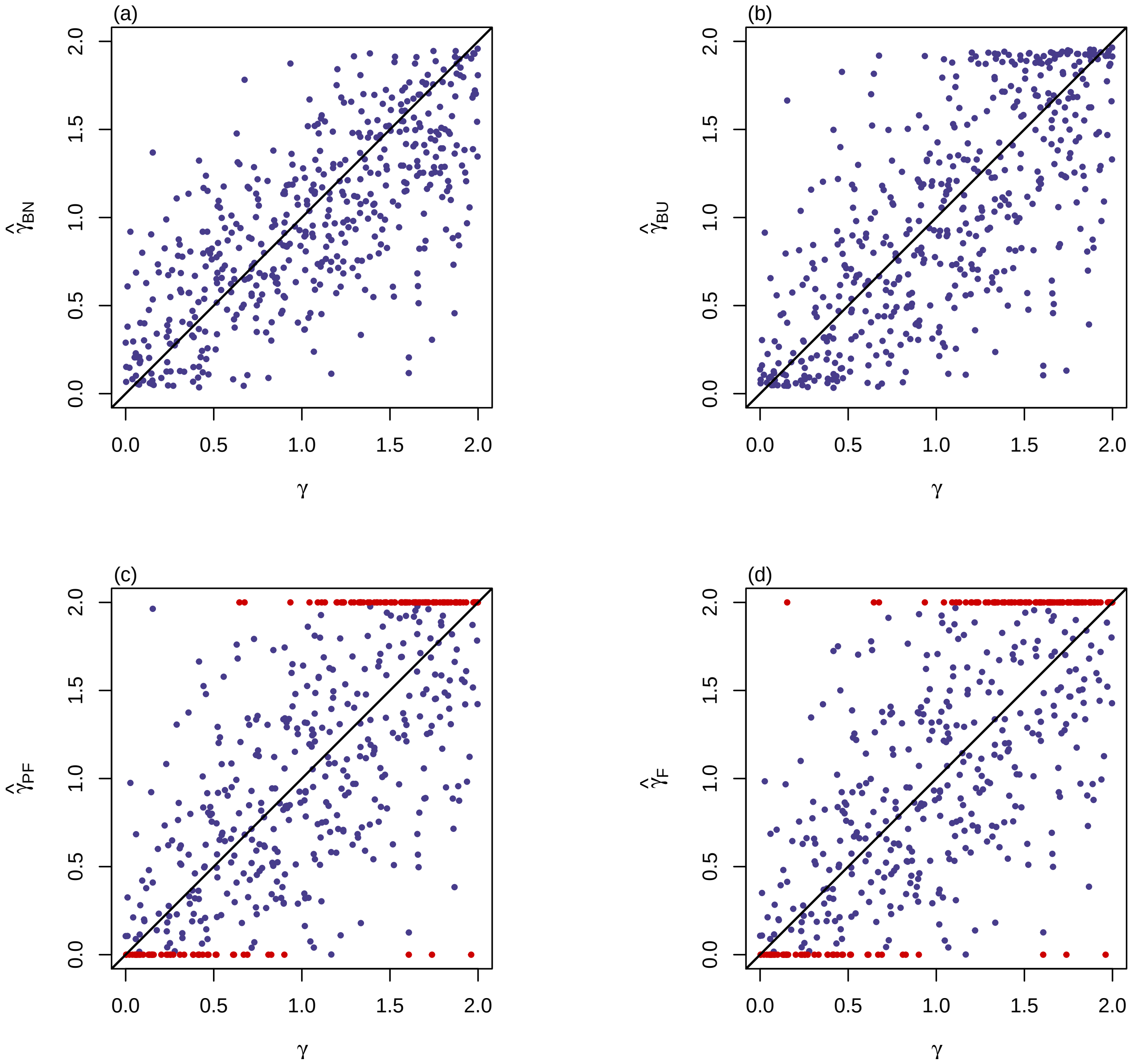

Supplementary Figure S2 Scatter plots of point estimates of γ are against true value of γ for qualitative trait with $n = 500$, MAF $= 0.3$ and $\rho = 0.05$. The red points represent the extreme values (0 or 2). **(a)** $\hat{\gamma}_{BN}$; **(b)** $\hat{\gamma}_{BU}$; **(c)** $\hat{\gamma}_{PF}$; **(d)** $\hat{\gamma}_{F}$

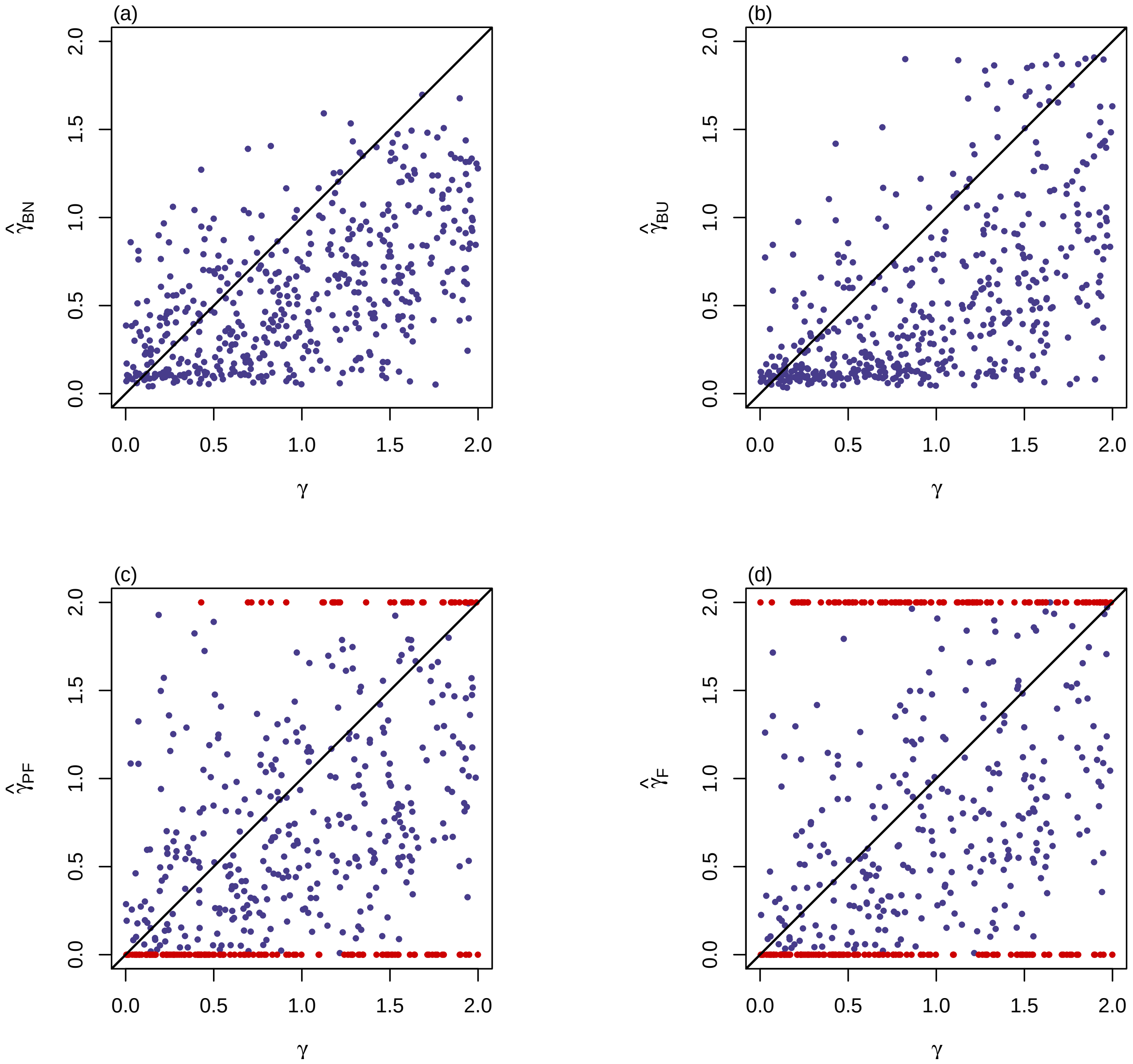

Supplementary Figure S3 Scatter plots of point estimates of γ against true value of γ for qualitative trait with $n = 500$, MAF $= 0.1$ and $\rho = 0$. The red points represent the extreme values (0 or 2). **(a)** $\hat{\gamma}_{BN}$; **(b)** $\hat{\gamma}_{BU}$; **(c)** $\hat{\gamma}_{PF}$; **(d)** $\hat{\gamma}_{F}$

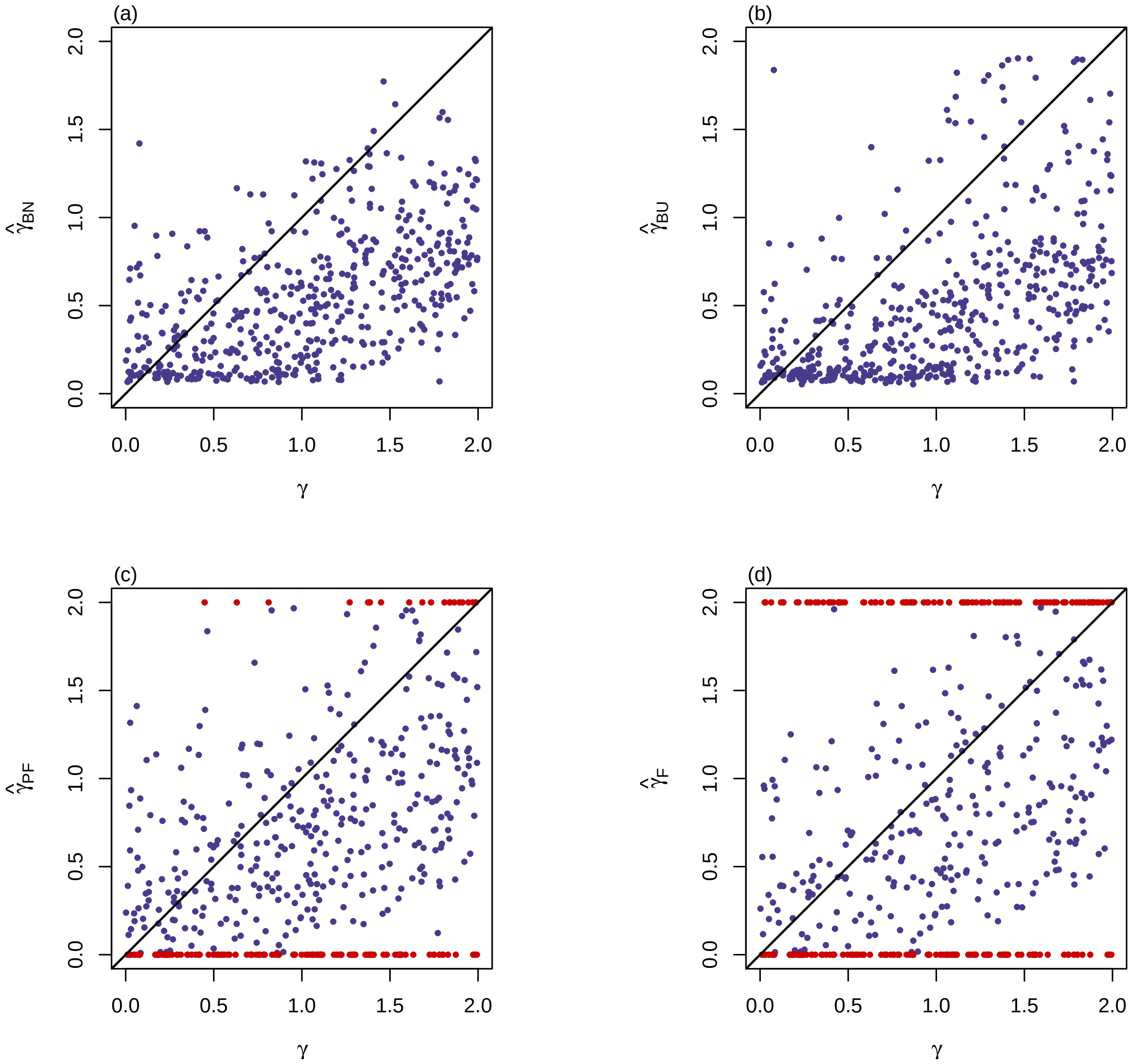

Supplementary Figure S4 Scatter plots of point estimates of γ against true value of γ for qualitative trait with $n = 500$, MAF $= 0.1$ and $\rho = -0.05$. The red points represent the extreme values (0 or 2). **(a)** $\hat{\gamma}_{BN}$; **(b)** $\hat{\gamma}_{BU}$; **(c)** $\hat{\gamma}_{PF}$; **(d)** $\hat{\gamma}_{F}$

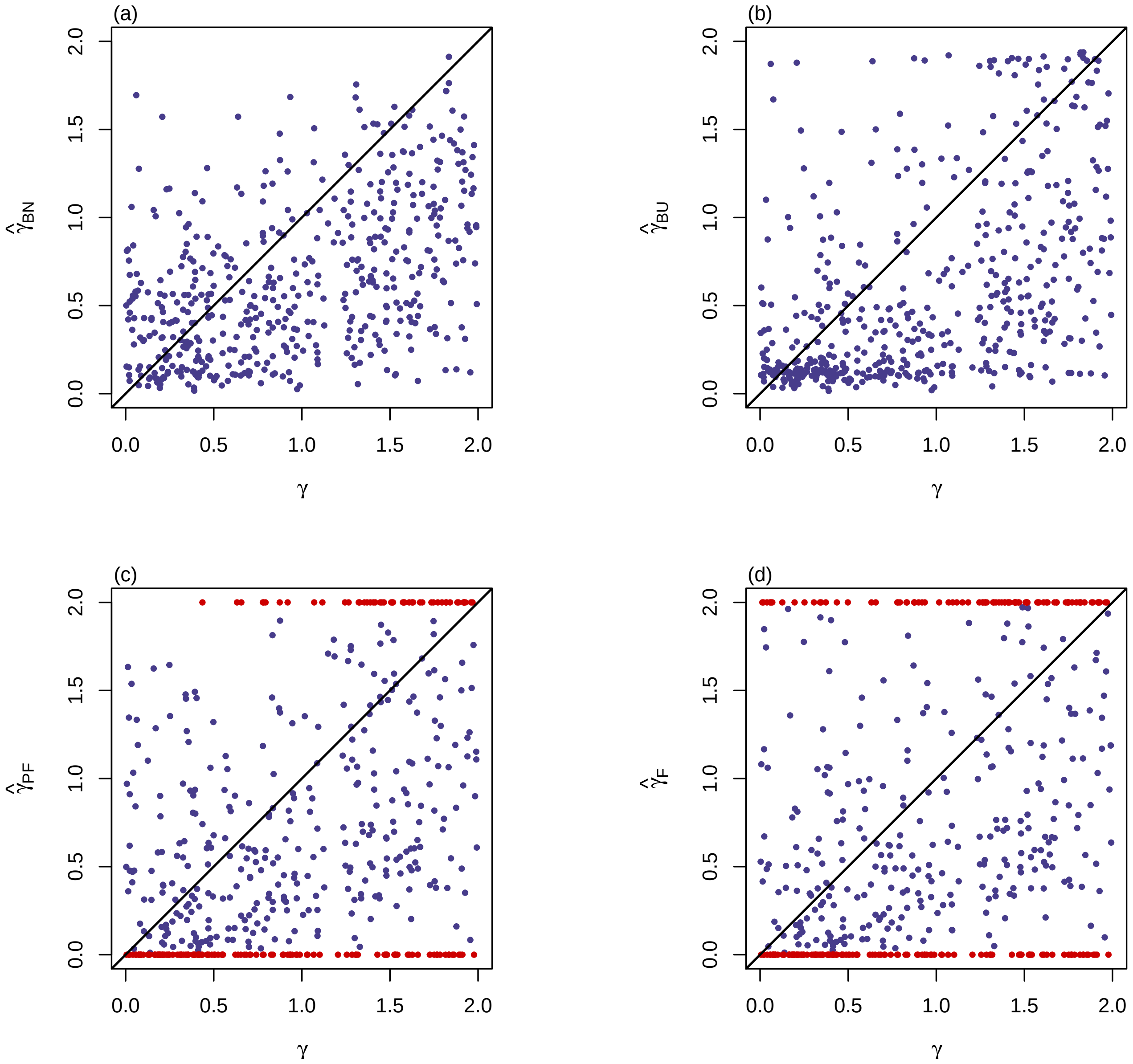

Supplementary Figure S5 Scatter plots of point estimates of γ against true value of γ for qualitative trait with $n = 500$, MAF $= 0.1$ and $\rho = 0.05$. The red points represent the extreme values (0 or 2). **(a)** $\hat{\gamma}_{BN}$; **(b)** $\hat{\gamma}_{BU}$; **(c)** $\hat{\gamma}_{PF}$; **(d)** $\hat{\gamma}_{F}$

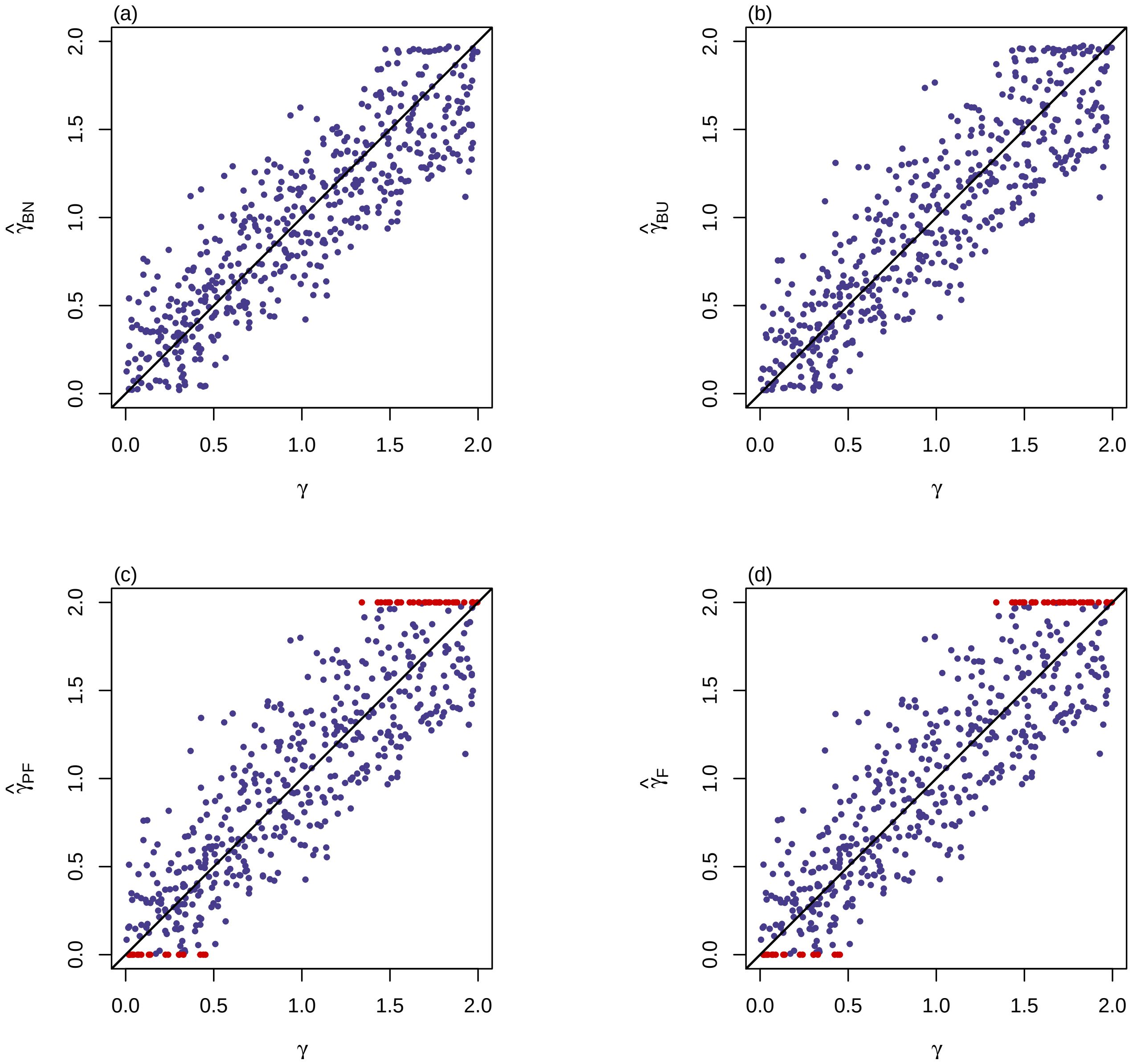

Supplementary Figure S6 Scatter plots of point estimates of γ against true value of γ for qualitative trait with $n = 2000$, MAF $= 0.3$ and $\rho = 0$. The red points represent the extreme values (0 or 2). **(a)** $\hat{\gamma}_{BN}$; **(b)** $\hat{\gamma}_{BU}$; **(c)** $\hat{\gamma}_{PF}$; **(d)** $\hat{\gamma}_{F}$

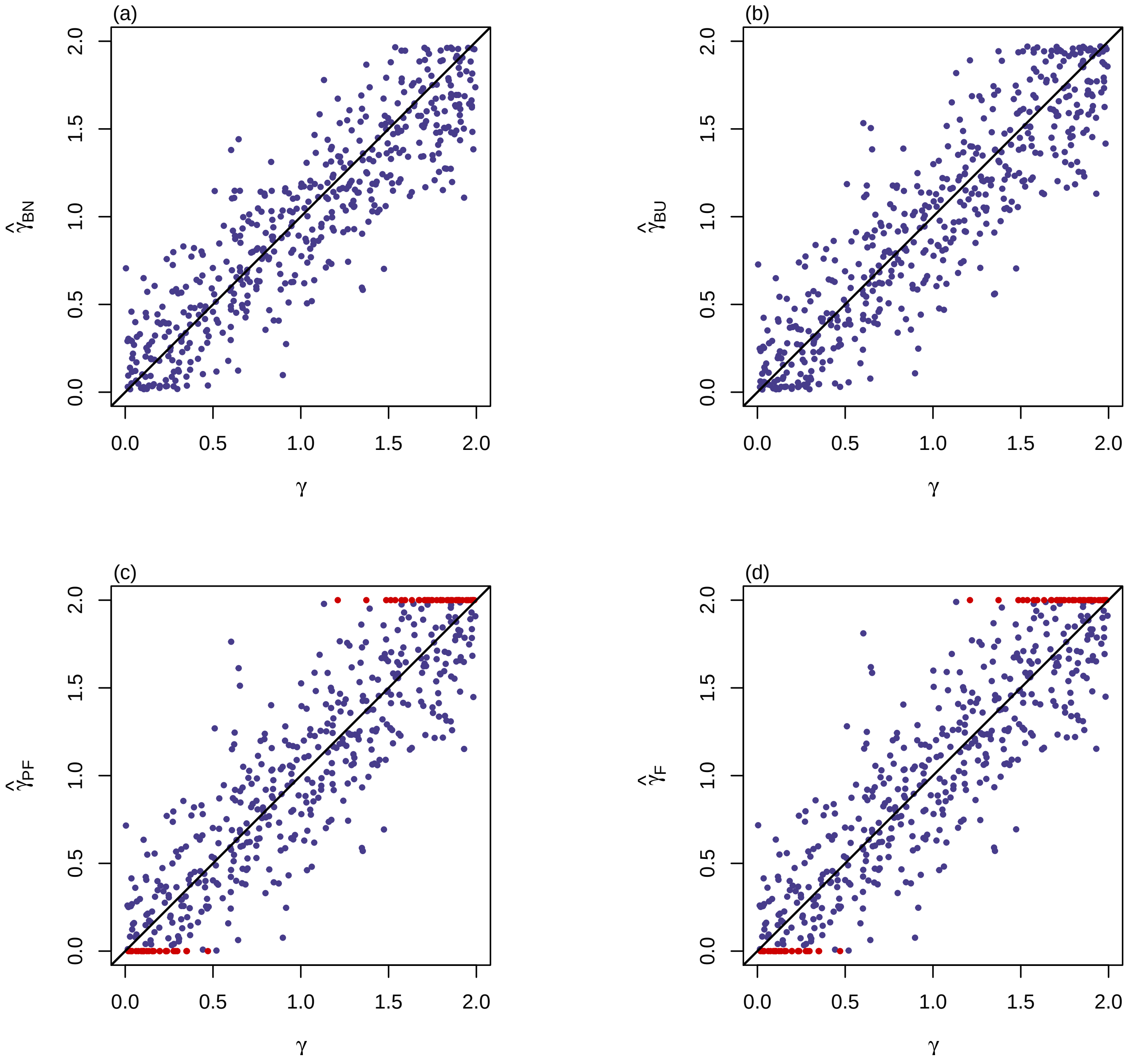

Supplementary Figure S7 Scatter plots of point estimates of γ against true value of γ for qualitative trait with $n = 2000$, MAF $= 0.3$ and $\rho = -0.05$. The red points represent the extreme values (0 or 2). **(a)** $\hat{\gamma}_{BN}$; **(b)** $\hat{\gamma}_{BU}$; **(c)** $\hat{\gamma}_{PF}$; **(d)** $\hat{\gamma}_{F}$

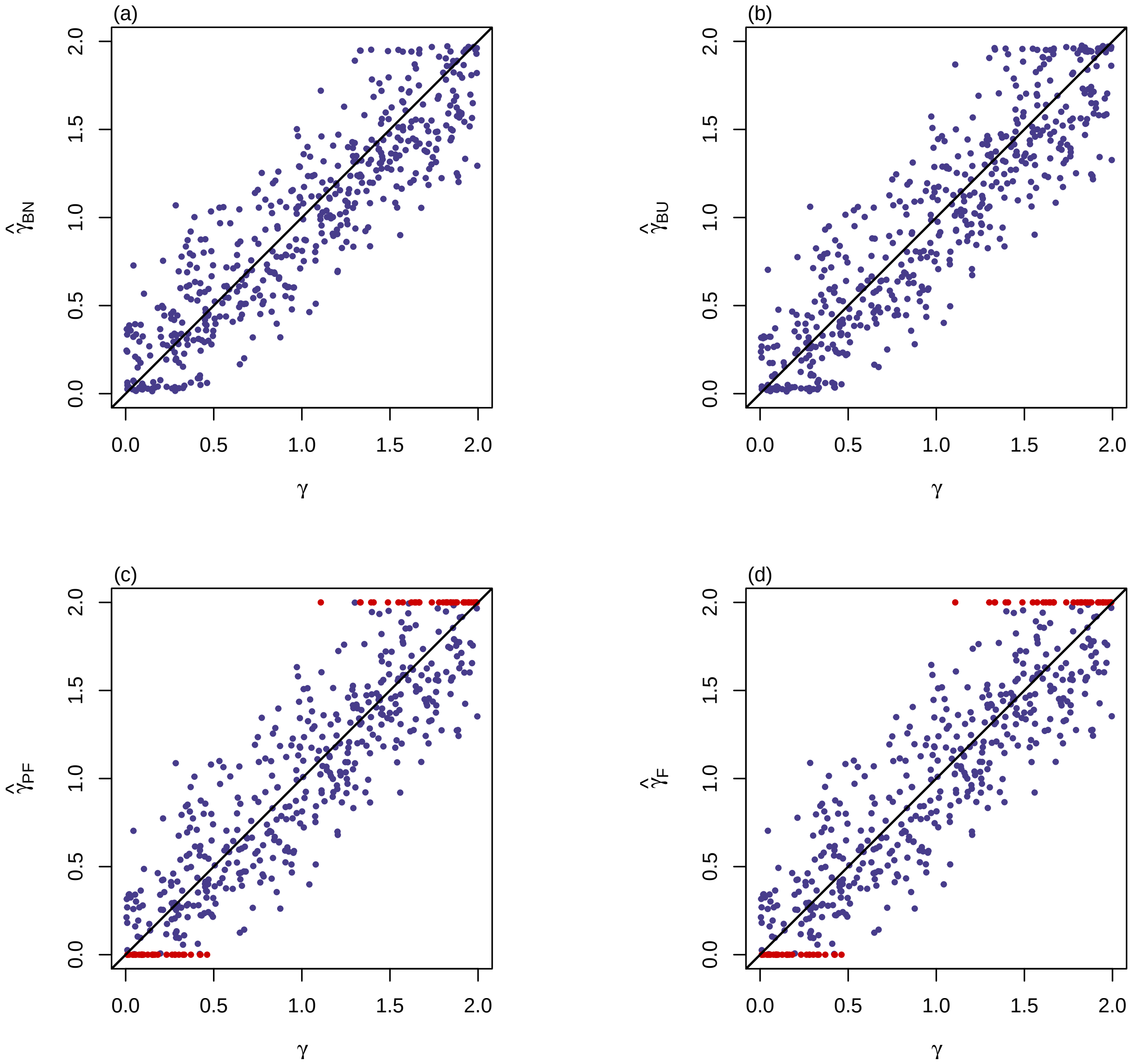

Supplementary Figure S8 Scatter plots of point estimates of γ against true value of γ for qualitative trait with $n = 2000$, MAF $= 0.3$ and $\rho = 0.05$. The red points represent the extreme values (0 or 2). **(a)** $\hat{\gamma}_{BN}$; **(b)** $\hat{\gamma}_{BU}$; **(c)** $\hat{\gamma}_{PF}$; **(d)** $\hat{\gamma}_{F}$

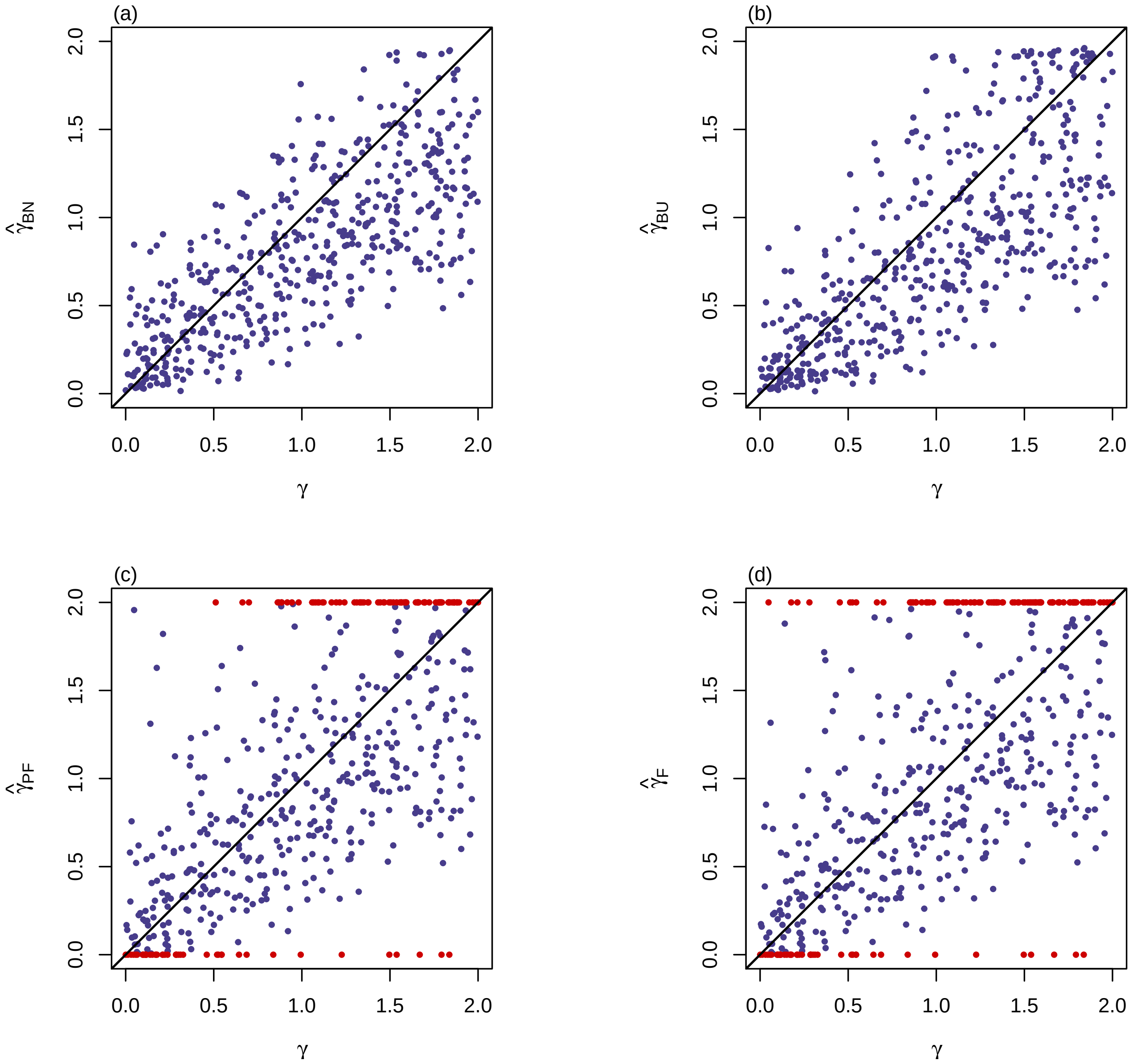

Supplementary Figure S9 Scatter plots of point estimates of γ against true value of γ for qualitative trait with $n = 2000$, MAF $= 0.1$ and $\rho = 0$. The red points represent the extreme values (0 or 2). **(a)** $\hat{\gamma}_{BN}$; **(b)** $\hat{\gamma}_{BU}$; **(c)** $\hat{\gamma}_{PF}$; **(d)** $\hat{\gamma}_F$

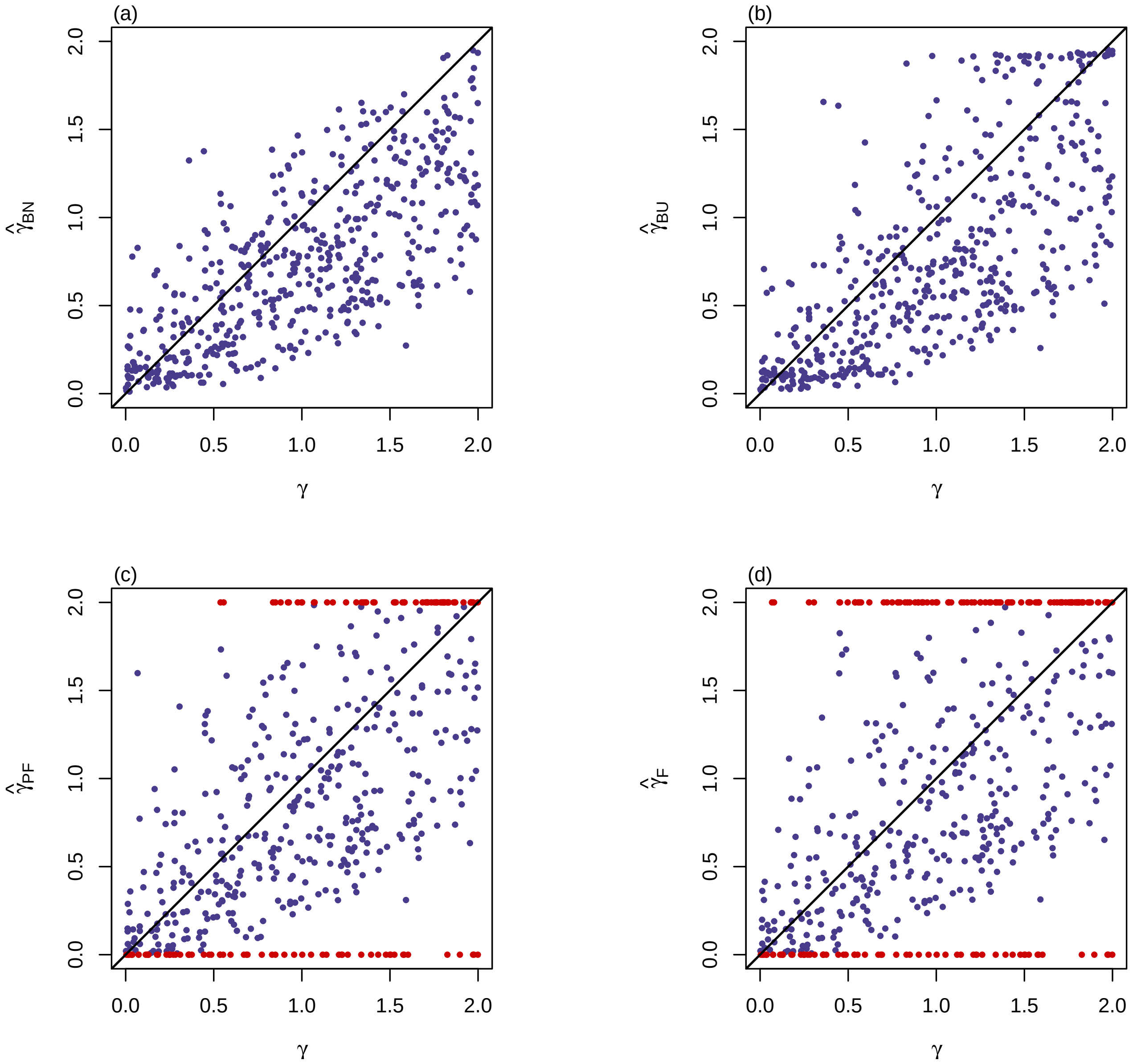

Supplementary Figure S10 Scatter plots of point estimates of γ against true value of γ for qualitative trait with $n = 2000$, MAF $= 0.1$ and $\rho = -0.05$. The red points represent the extreme values (0 or 2). **(a)** $\hat{\gamma}_{BN}$; **(b)** $\hat{\gamma}_{BU}$; **(c)** $\hat{\gamma}_{PF}$; **(d)** $\hat{\gamma}_F$

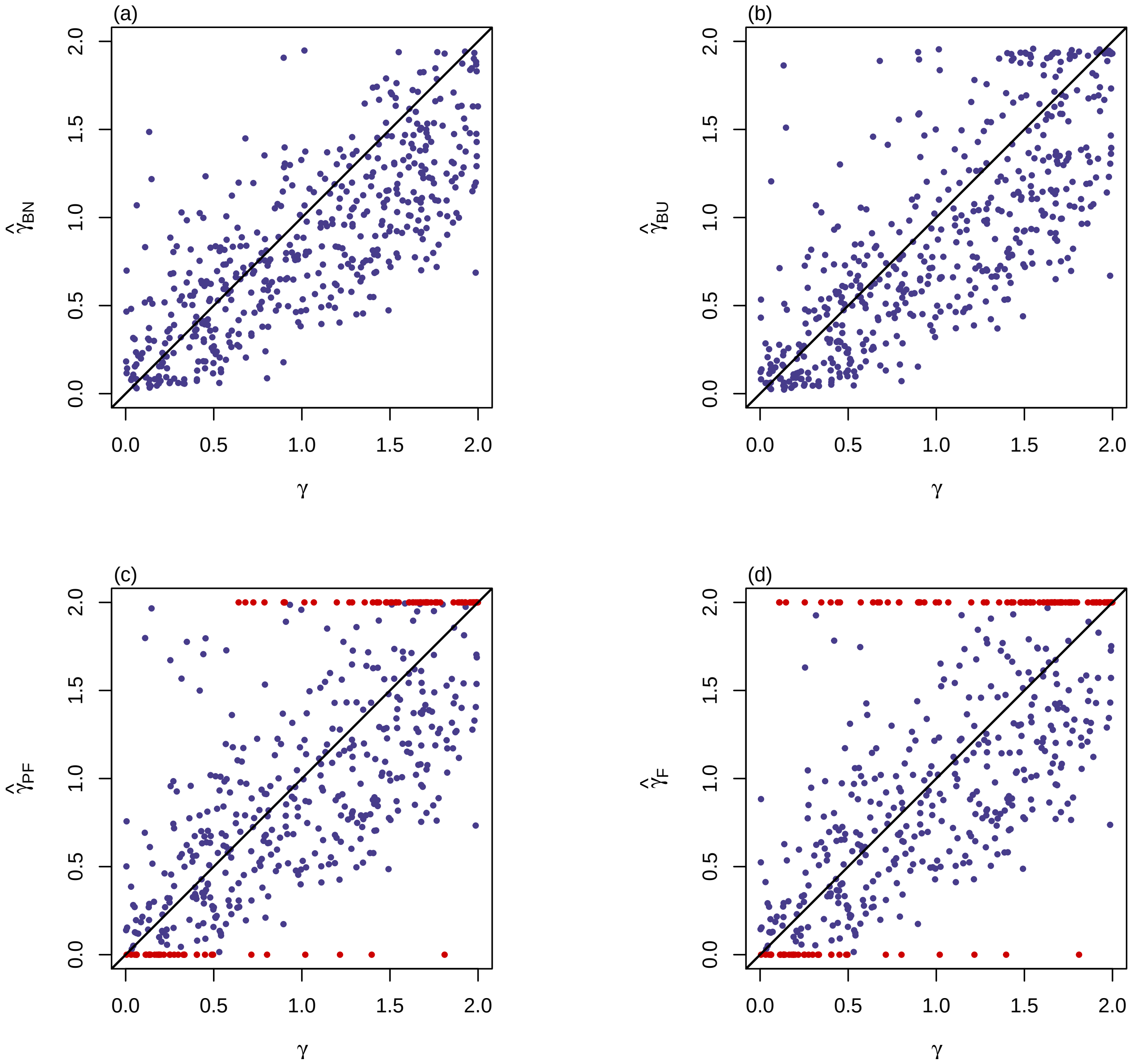

Supplementary Figure S11 Scatter plots of point estimates of γ against true value of γ for qualitative trait with $n = 2000$, MAF $= 0.1$ and $\rho = 0.05$. The red points represent the extreme values (0 or 2). **(a)** $\hat{\gamma}_{BN}$; **(b)** $\hat{\gamma}_{BU}$; **(c)** $\hat{\gamma}_{PF}$; **(d)** $\hat{\gamma}_{F}$

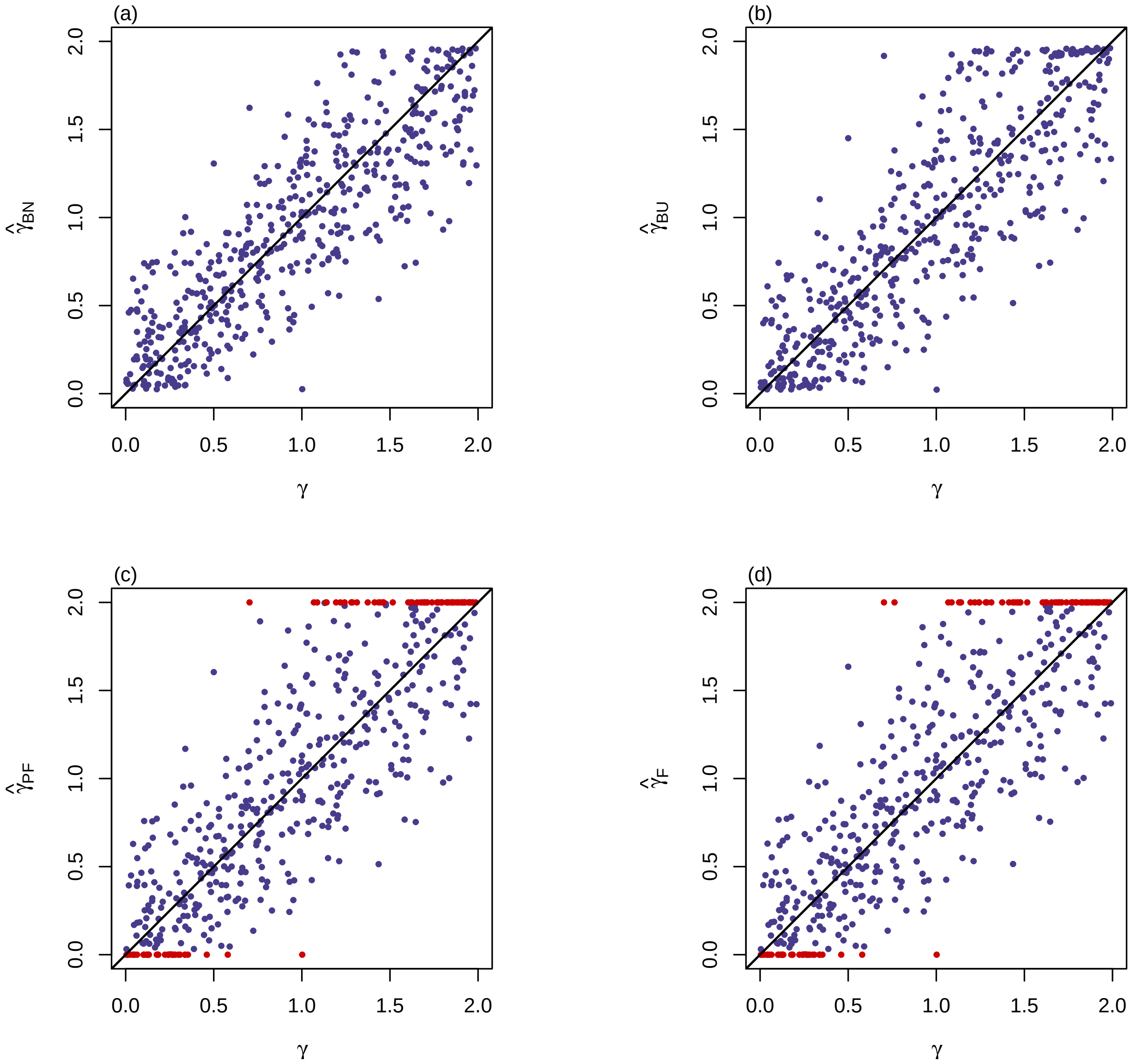

Supplementary Figure S12 Scatter plots of point estimates of γ against true value of γ for quantitative trait when $(\sigma_0^2,\ \sigma_1^2,\ \sigma_2^2) = (1,\ 1.2,\ 1)$ with $n = 500$, MAF $= 0.3$ and $\rho = -0.05$. The red points represent the extreme values (0 or 2). **(a)** $\hat{\gamma}_{BN}$; **(b)** $\hat{\gamma}_{BU}$; **(c)** $\hat{\gamma}_{PF}$; **(d)** $\hat{\gamma}_{F}$

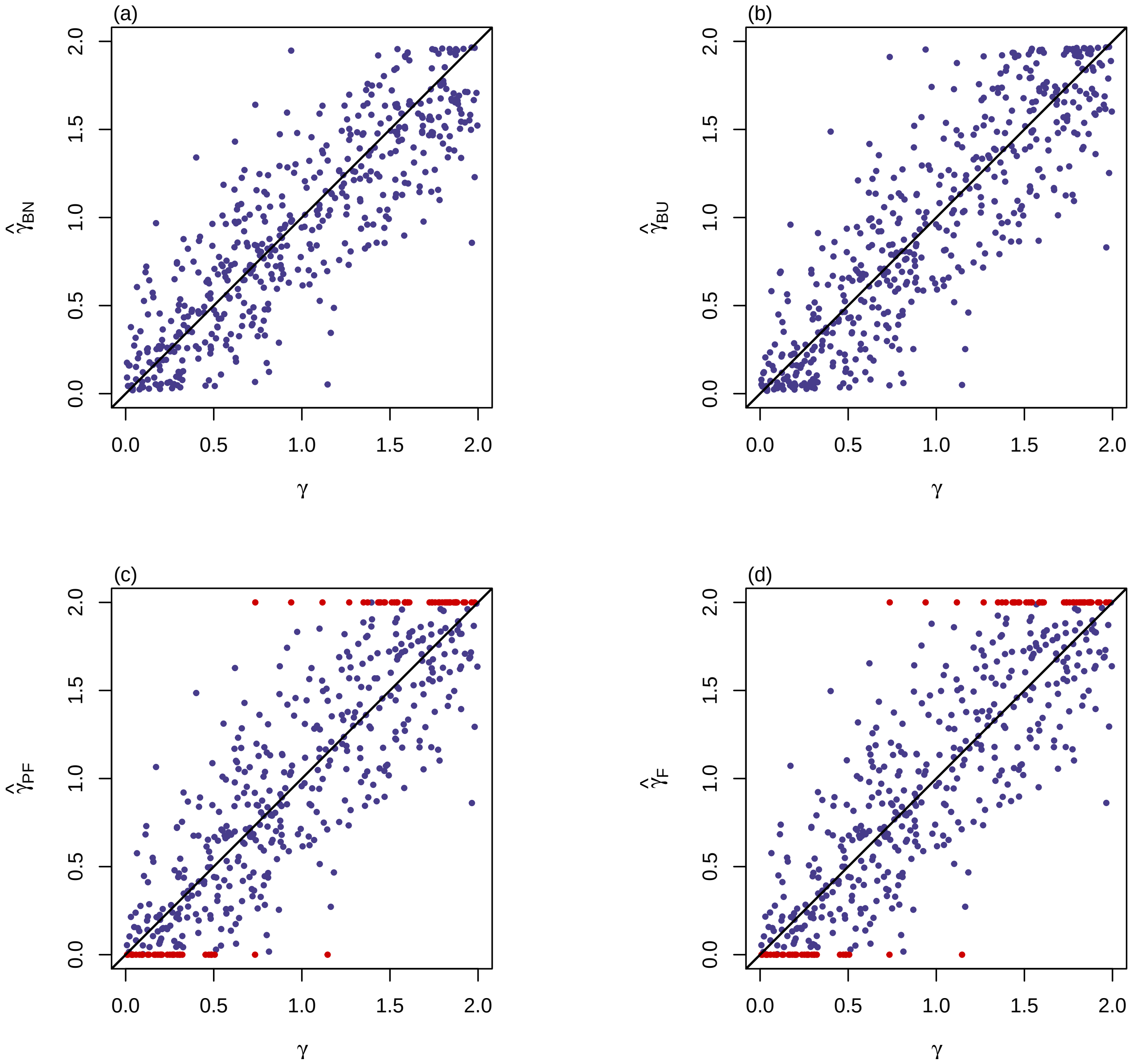

Supplementary Figure S13 Scatter plots of point estimates of γ against true value of γ for quantitative trait when $(\sigma_0^2,\ \sigma_1^2,\ \sigma_2^2) = (1,\ 1.2,\ 1)$ with $n = 500$, MAF $= 0.3$ and $\rho = 0.05$. The red points represent the extreme values (0 or 2). **(a)** $\hat{\gamma}_{BN}$; **(b)** $\hat{\gamma}_{BU}$; **(c)** $\hat{\gamma}_{PF}$; **(d)** $\hat{\gamma}_F$

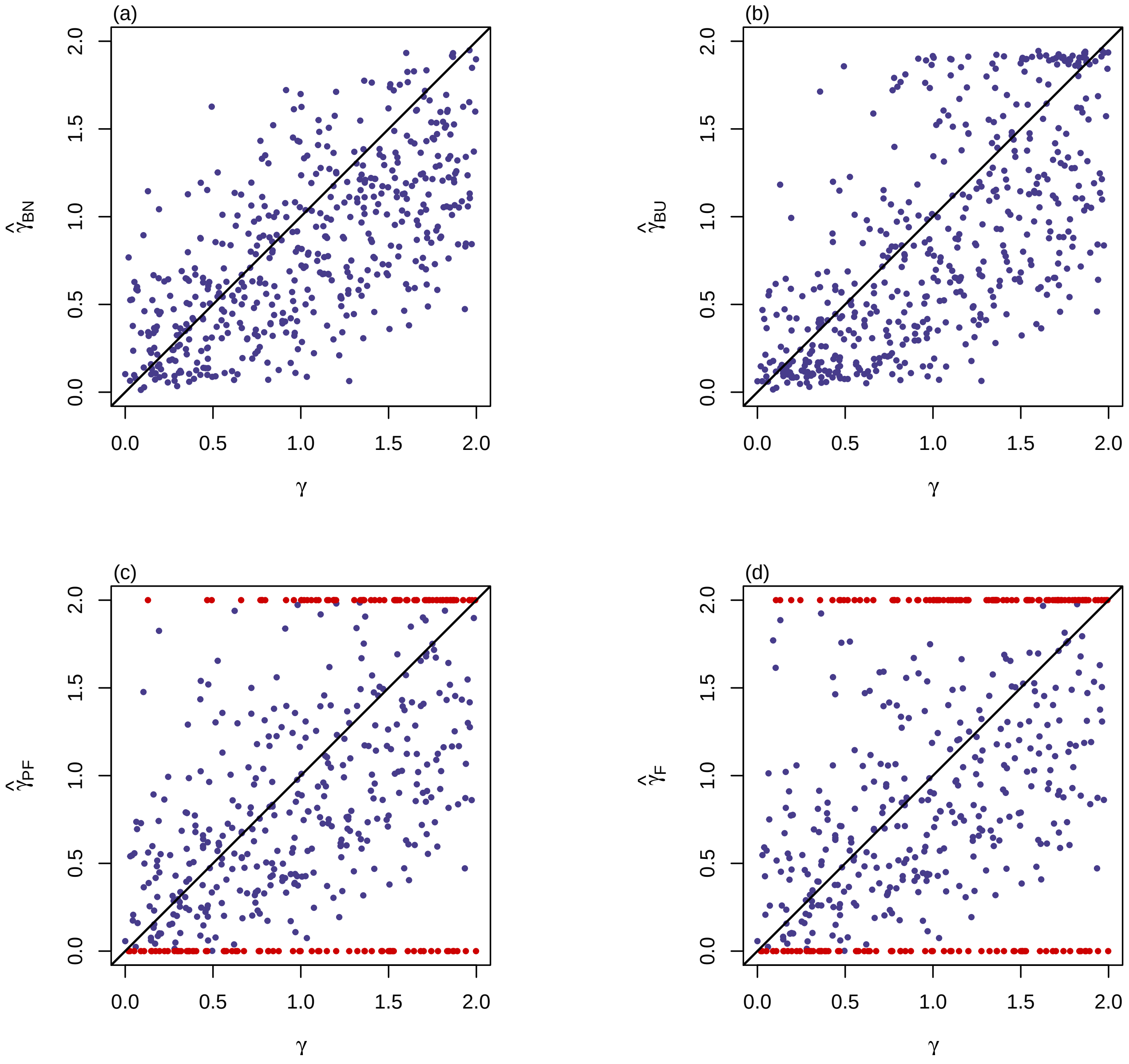

Supplementary Figure S14 Scatter plots of point estimates of γ against true value of γ for quantitative trait when $(\sigma_0^2,\ \sigma_1^2,\ \sigma_2^2) = (1,\ 1.2,\ 1)$ with $n = 500$, MAF $= 0.1$ and $\rho = 0$. The red points represent the extreme values (0 or 2). **(a)** $\hat{\gamma}_{BN}$; **(b)** $\hat{\gamma}_{BU}$; **(c)** $\hat{\gamma}_{PF}$; **(d)** $\hat{\gamma}_F$

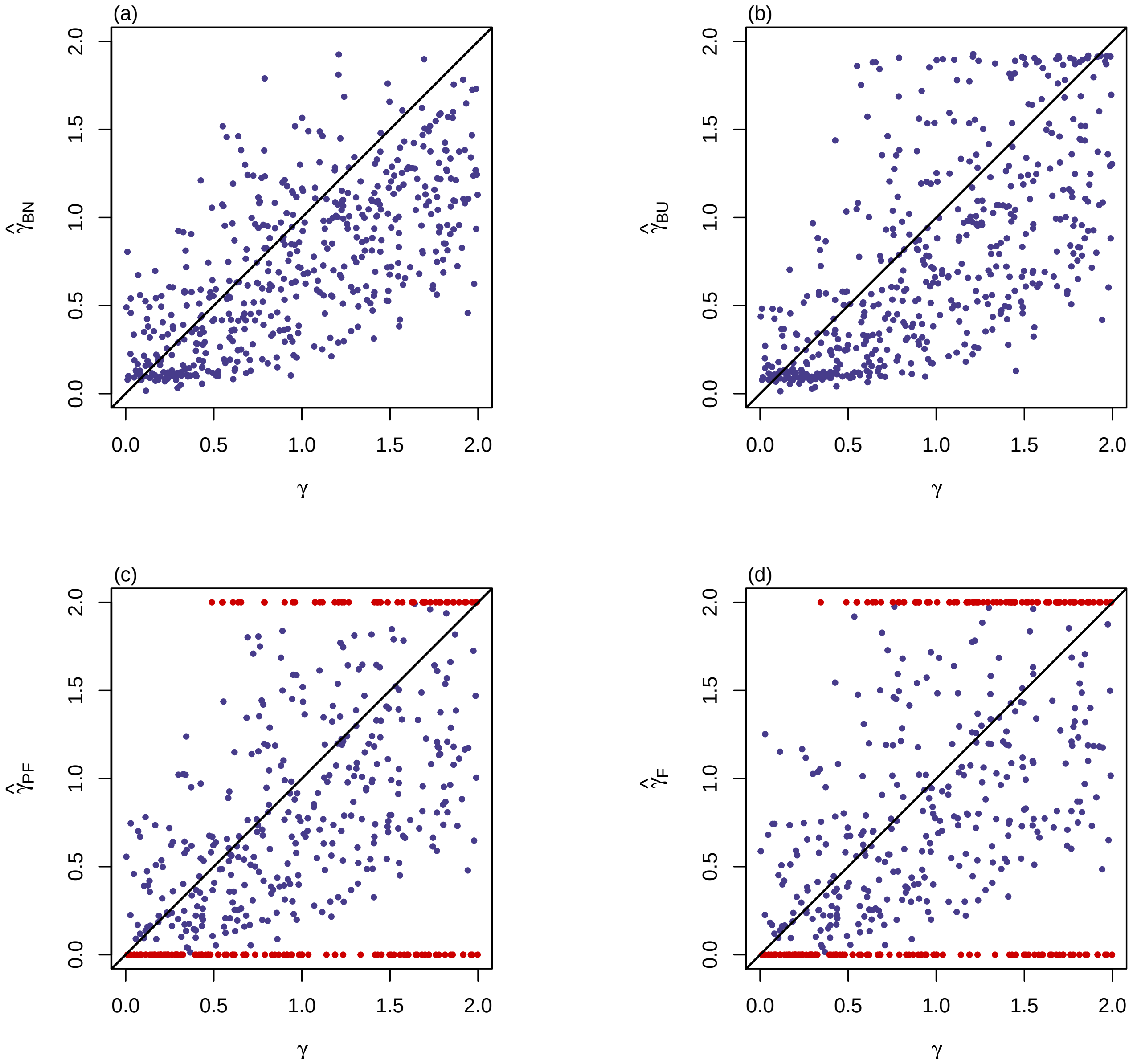

Supplementary Figure S15 Scatter plots of point estimates of γ against true value of γ for quantitative trait when $(\sigma_0^2,\ \sigma_1^2,\ \sigma_2^2) = (1,\ 1.2,\ 1)$ with $n = 500$, MAF $= 0.1$ and $\rho = -0.05$. The red points represent the extreme values (0 or 2). **(a)** $\hat{\gamma}_{BN}$; **(b)** $\hat{\gamma}_{BU}$; **(c)** $\hat{\gamma}_{PF}$; **(d)** $\hat{\gamma}_F$

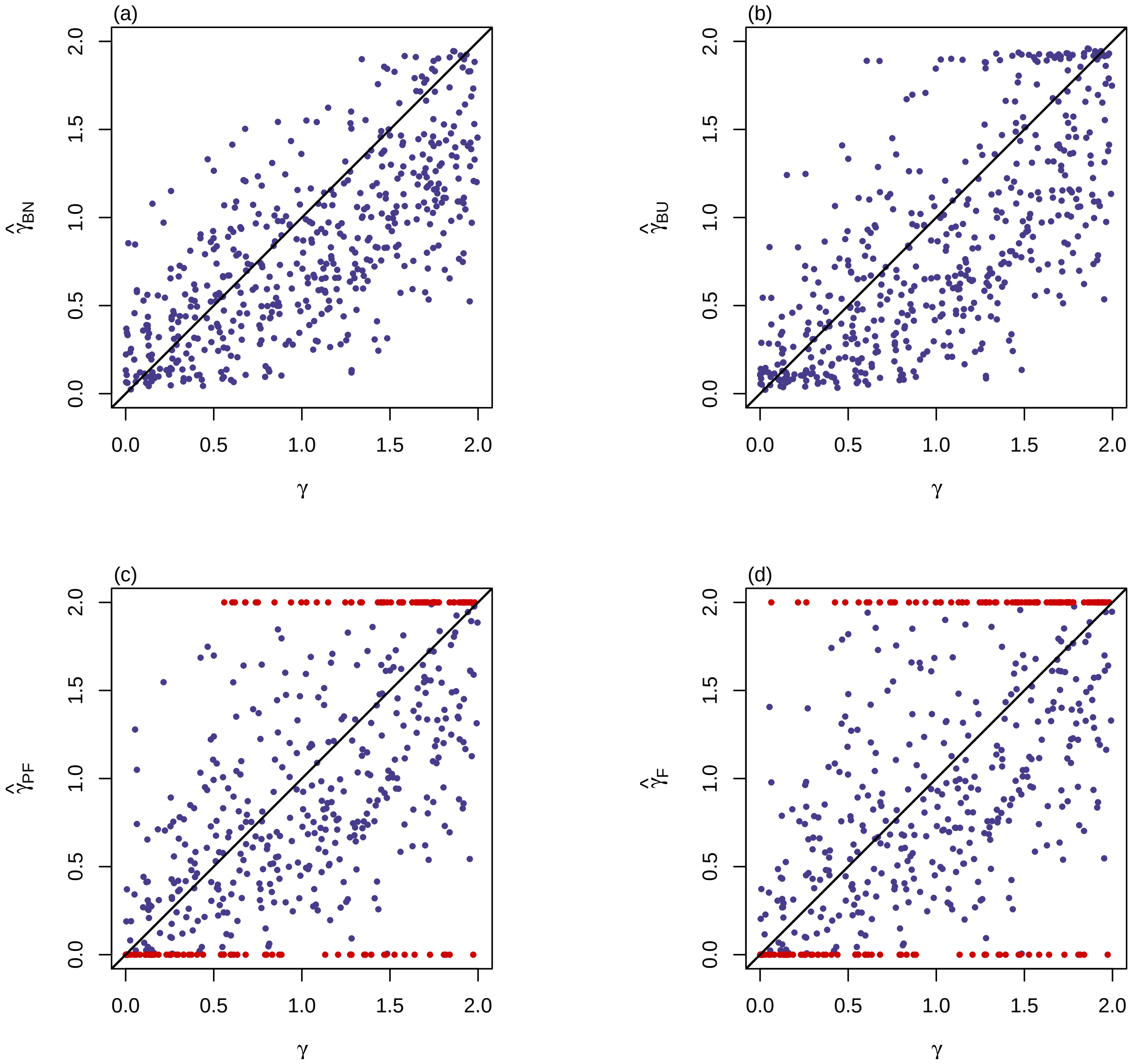

Supplementary Figure S16 Scatter plots of point estimates of γ against true value of γ for quantitative trait when $(\sigma_0^2,\ \sigma_1^2,\ \sigma_2^2) = (1,\ 1.2,\ 1)$ with $n = 500$, MAF $= 0.1$ and $\rho = 0.05$. The red points represent the extreme values (0 or 2). **(a)** $\hat{\gamma}_{BN}$; **(b)** $\hat{\gamma}_{BU}$; **(c)** $\hat{\gamma}_{PF}$; **(d)** $\hat{\gamma}_{F}$

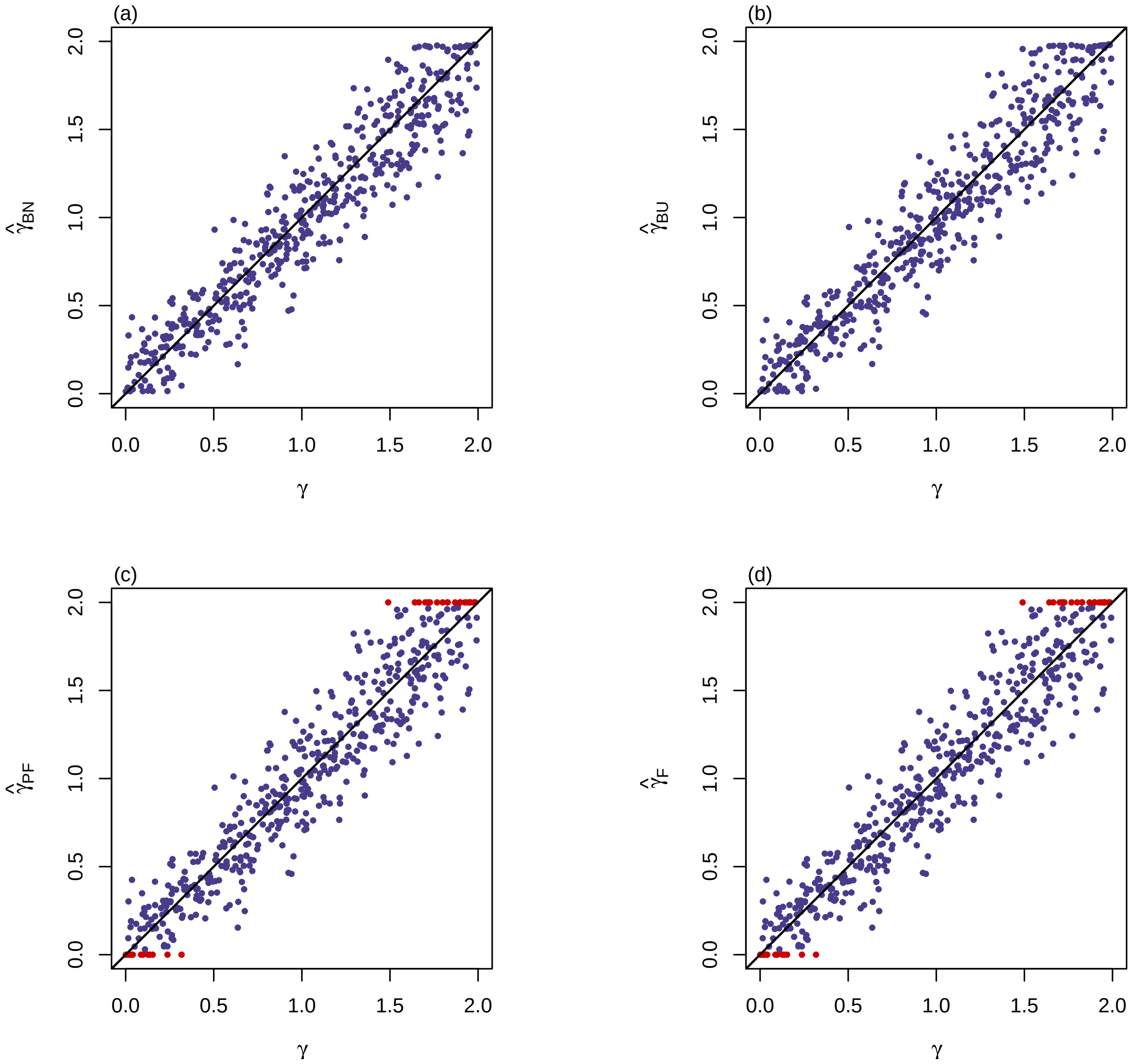

Supplementary Figure S17 Scatter plots of point estimates of γ against true value of γ for quantitative trait when $(\sigma_0^2,\ \sigma_1^2,\ \sigma_2^2) = (1,\ 1.2,\ 1)$ with $n = 2000$, MAF $= 0.3$ and $\rho = 0$. The red points represent the extreme values (0 or 2). **(a)** $\hat{\gamma}_{BN}$; **(b)** $\hat{\gamma}_{BU}$; **(c)** $\hat{\gamma}_{PF}$; **(d)** $\hat{\gamma}_F$

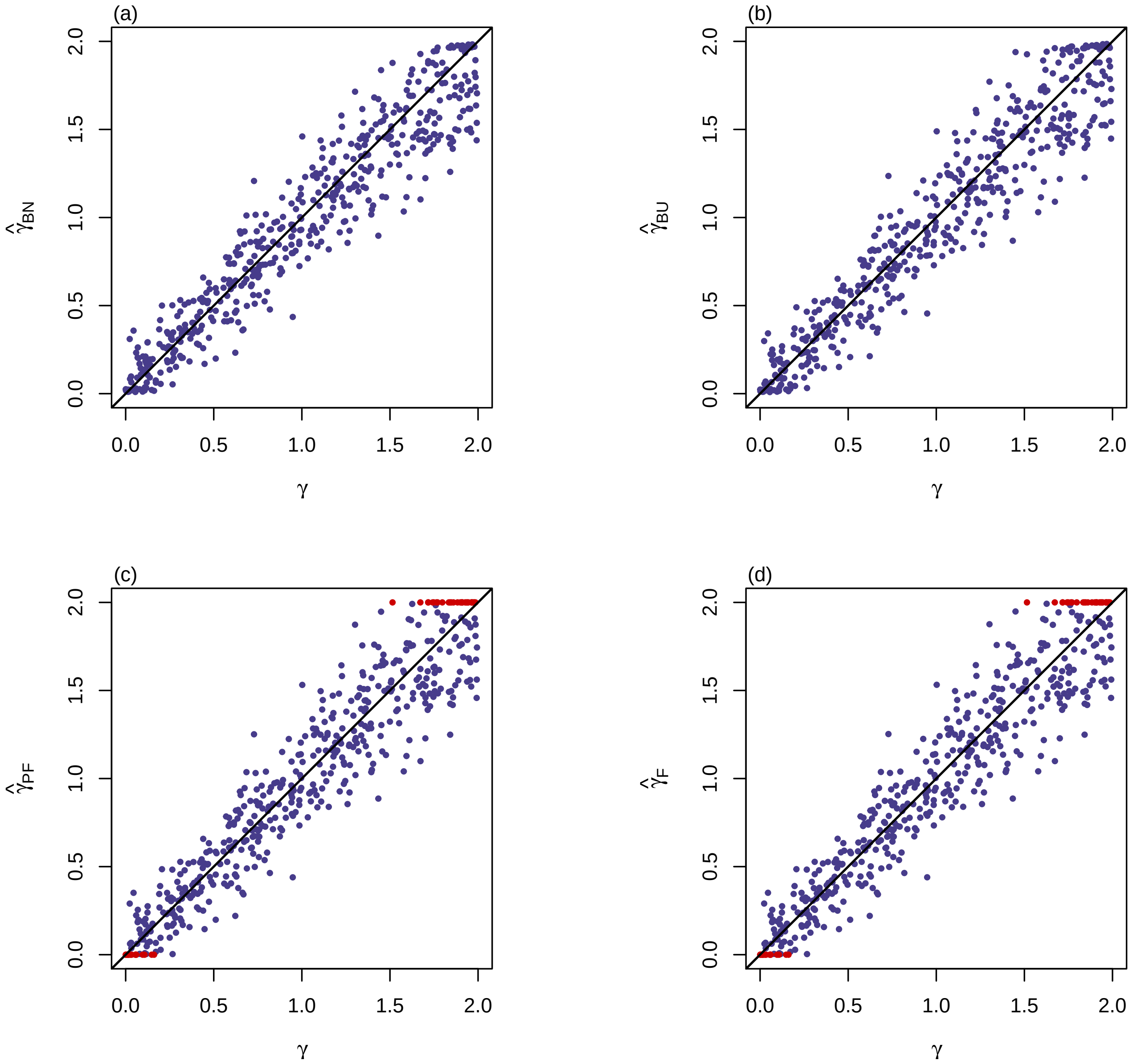

Supplementary Figure S18 Scatter plots of point estimates of γ against true value of γ for quantitative trait when $(\sigma_0^2,\ \sigma_1^2,\ \sigma_2^2) = (1,\ 1.2,\ 1)$ with $n = 2000$, MAF $= 0.3$ and $\rho = -0.05$. The red points represent the extreme values (0 or 2). **(a)** $\hat{\gamma}_{BN}$; **(b)** $\hat{\gamma}_{BU}$; **(c)** $\hat{\gamma}_{PF}$; **(d)** $\hat{\gamma}_F$

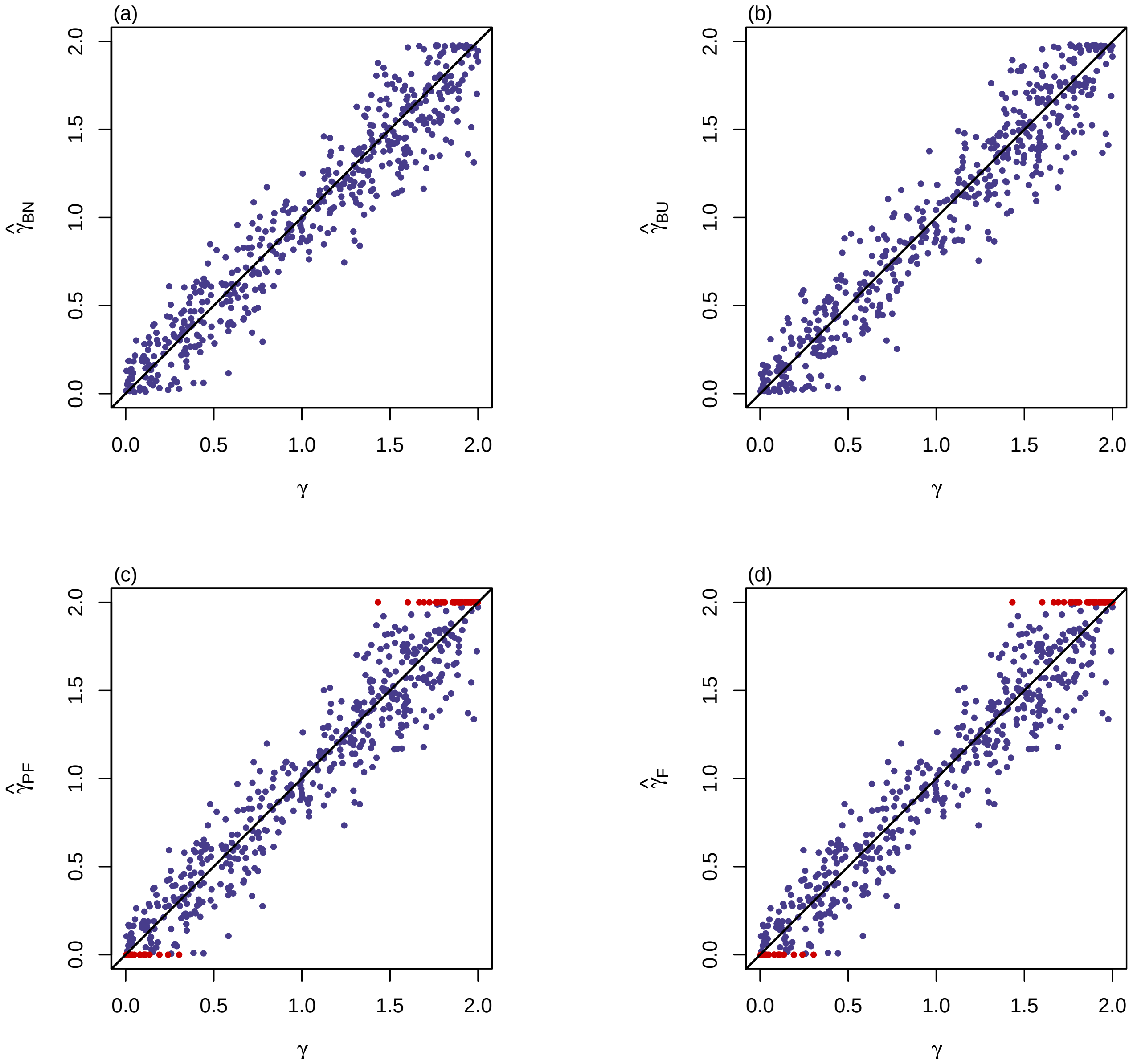

Supplementary Figure S19 Scatter plots of point estimates of γ against true value of γ for quantitative trait when $(\sigma_0^2,\ \sigma_1^2,\ \sigma_2^2) = (1,\ 1.2,\ 1)$ with $n = 2000$, MAF $= 0.3$ and $\rho = 0.05$. The red points represent the extreme values (0 or 2). **(a)** $\hat{\gamma}_{BN}$; **(b)** $\hat{\gamma}_{BU}$; **(c)** $\hat{\gamma}_{PF}$; **(d)** $\hat{\gamma}_F$

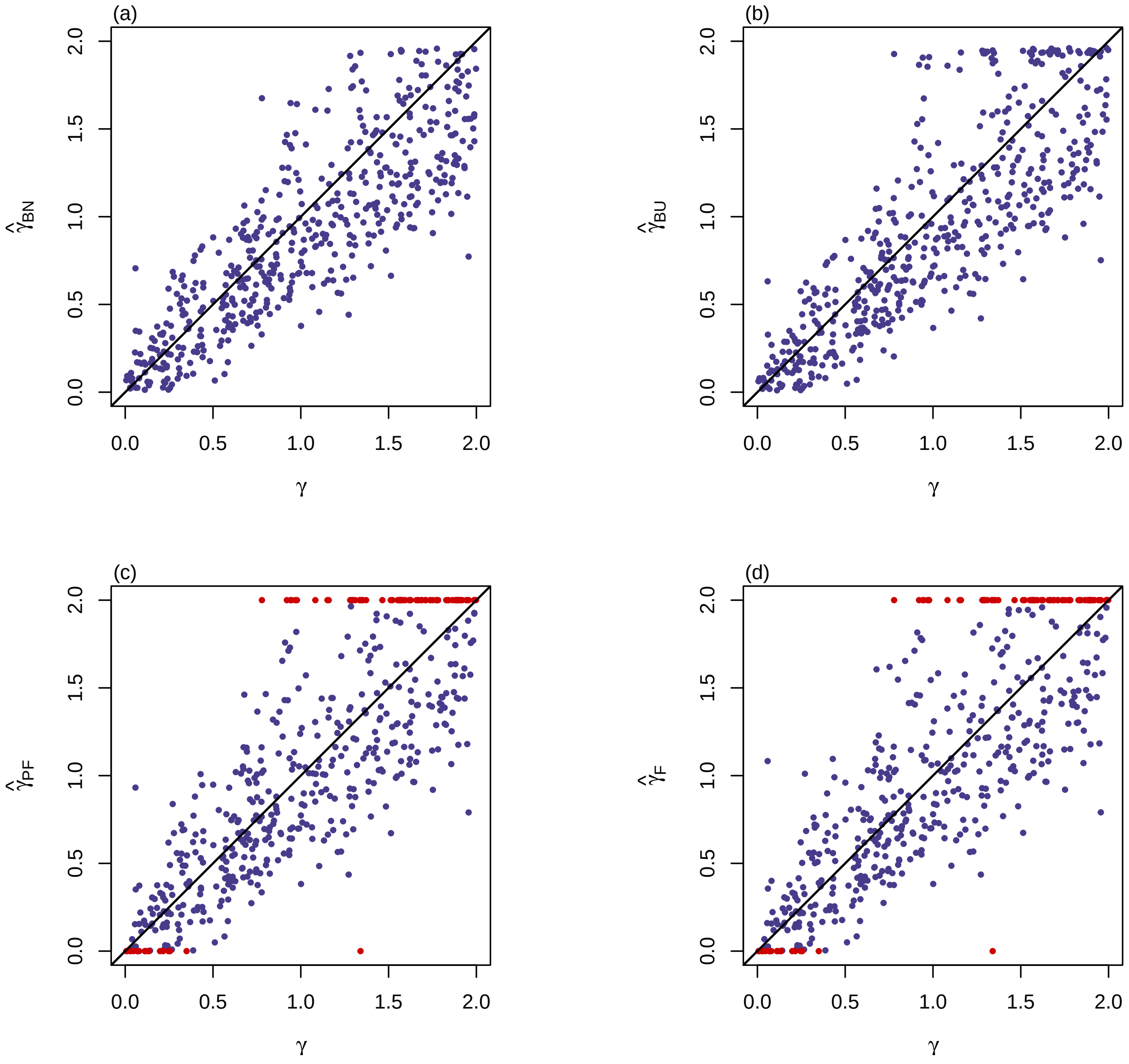

Supplementary Figure S20 Scatter plots of point estimates of γ against true value of γ for quantitative trait when $(\sigma_0^2,\ \sigma_1^2,\ \sigma_2^2) = (1,\ 1.2,\ 1)$ with $n = 2000$, MAF $= 0.1$ and $\rho = 0$. The red points represent the extreme values (0 or 2). **(a)** $\hat{\gamma}_{BN}$; **(b)** $\hat{\gamma}_{BU}$; **(c)** $\hat{\gamma}_{PF}$; **(d)** $\hat{\gamma}_F$

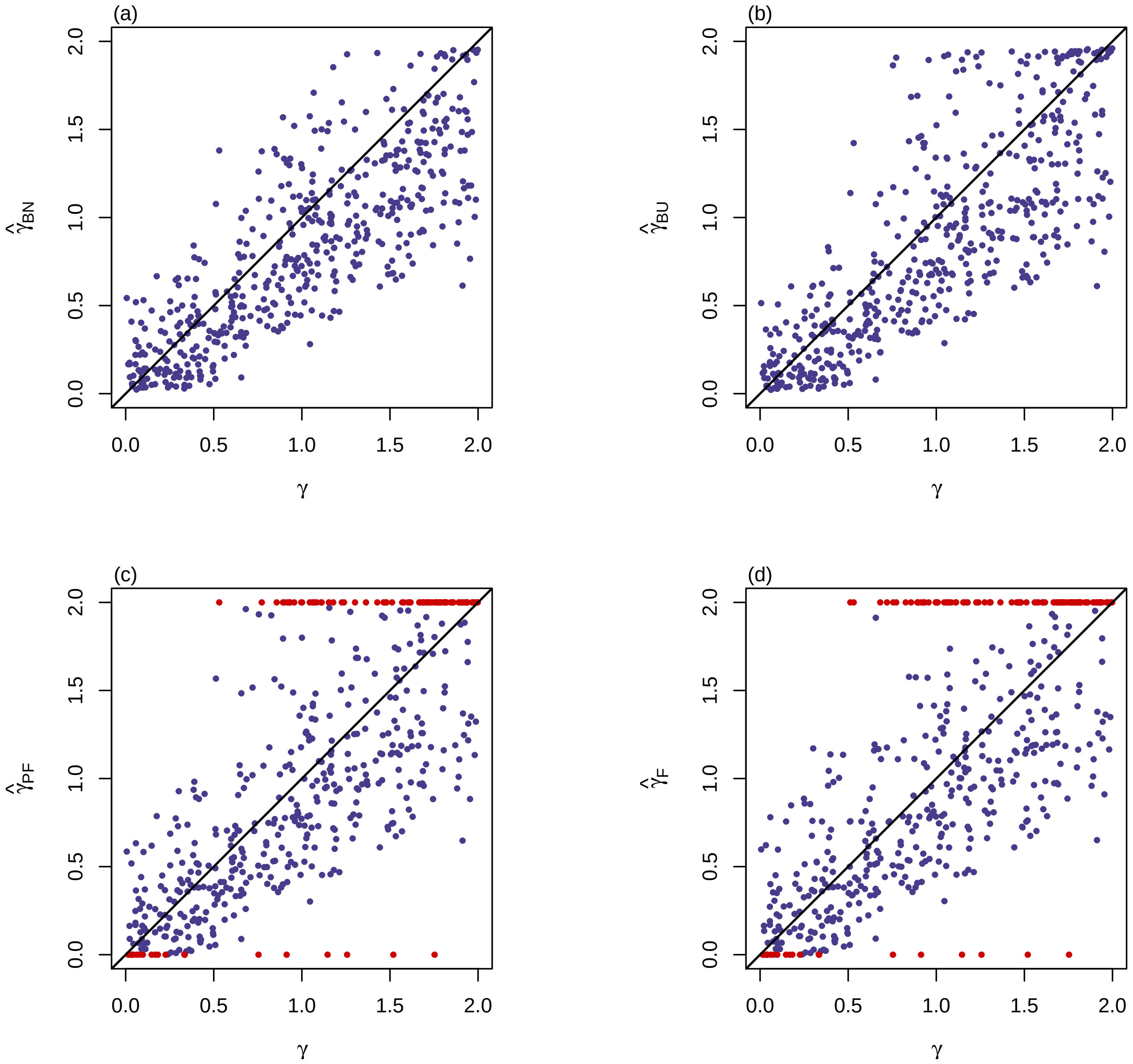

Supplementary Figure S21 Scatter plots of point estimates of γ against true value of γ for quantitative trait when $(\sigma_0^2,\ \sigma_1^2,\ \sigma_2^2) = (1,\ 1.2,\ 1)$ with $n = 2000$, MAF $= 0.1$ and $\rho = -0.05$. The red points represent the extreme values (0 or 2). **(a)** $\hat{\gamma}_{BN}$; **(b)** $\hat{\gamma}_{BU}$; **(c)** $\hat{\gamma}_{PF}$; **(d)** $\hat{\gamma}_F$

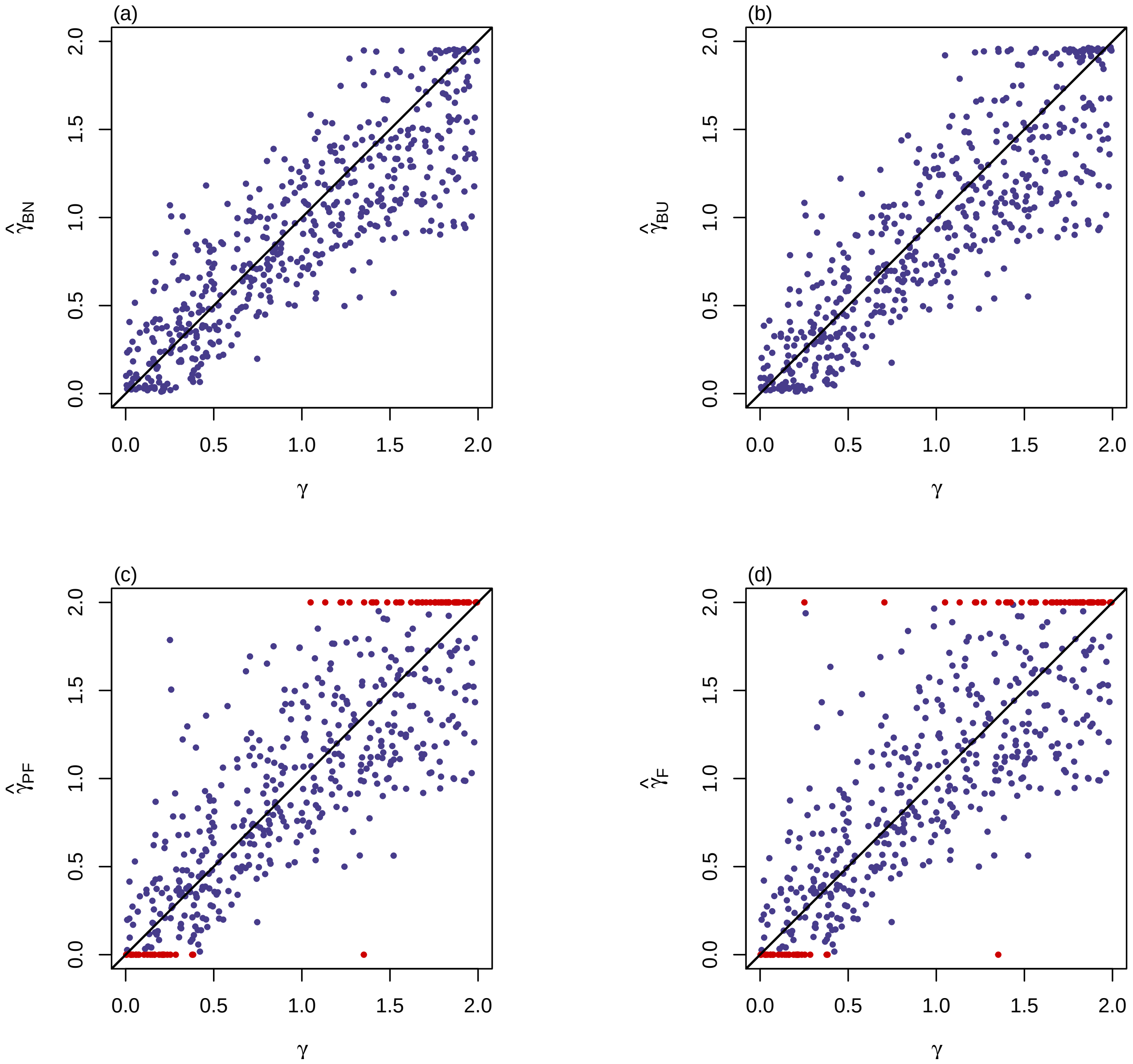

Supplementary Figure S22 Scatter plots of point estimates of γ against true value of γ for quantitative trait when $(\sigma_0^2,\ \sigma_1^2,\ \sigma_2^2) = (1,\ 1.2,\ 1)$ with $n = 2000$, MAF $= 0.1$ and $\rho = 0.05$. The red points represent the extreme values (0 or 2). **(a)** $\hat{\gamma}_{BN}$; **(b)** $\hat{\gamma}_{BU}$; **(c)** $\hat{\gamma}_{PF}$; **(d)** $\hat{\gamma}_F$
