# Supplementary material for: BEXCIS: Bayesian methods for estimating the degree of the skewness of X chromosome inactivation
Source: BMC Bioinformatics. 2022 May 24;23:193. doi: 10.1186/s12859-022-04721-y (PMC9128296; doi:10.1186/s12859-022-04721-y)
Supplement: Supplementary file 2 — Additional file 2. Figs. S23-S44. Widths of HPDIs or CIs against true value of γ for qualitative trait and quantitative trait when (σ02, σ12, σ22)=(1, 1.2, 1) with n=500 and 2000, MAF=0.3 and 0.1, and ρ=0, -0.05 and 0.05, respectively. [file 12859_2022_4721_MOESM2_ESM.pdf]

Additional file 2: Supplementary Figures S23–S44

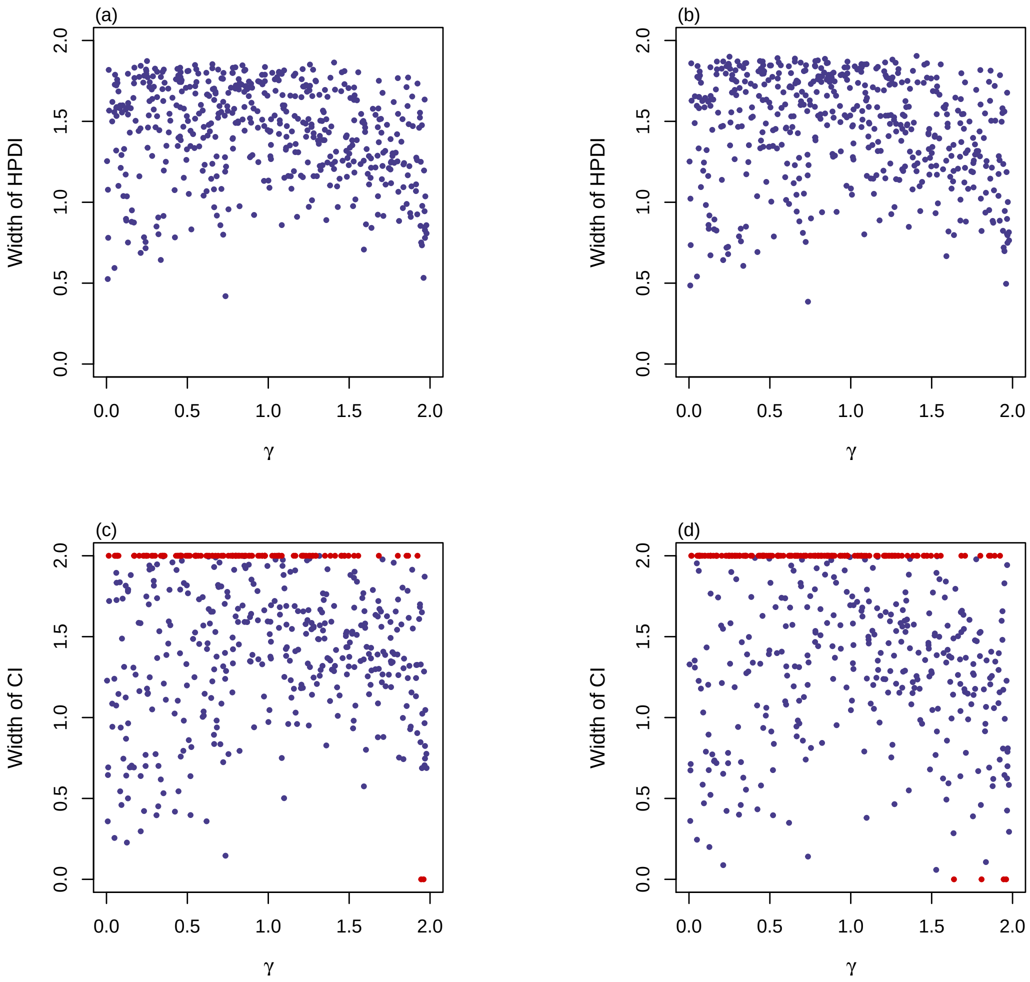

Supplementary Figure S23 Widths of HPDIs or CIs against true value of γ for qualitative trait with $n = 500$, MAF $= 0.3$ and $\rho = -0.05$. The red points represent the widths of the noninformative intervals or the empty sets. **(a)** BN method; **(b)** BU method; **(c)** PF method; **(d)** Fieller's method

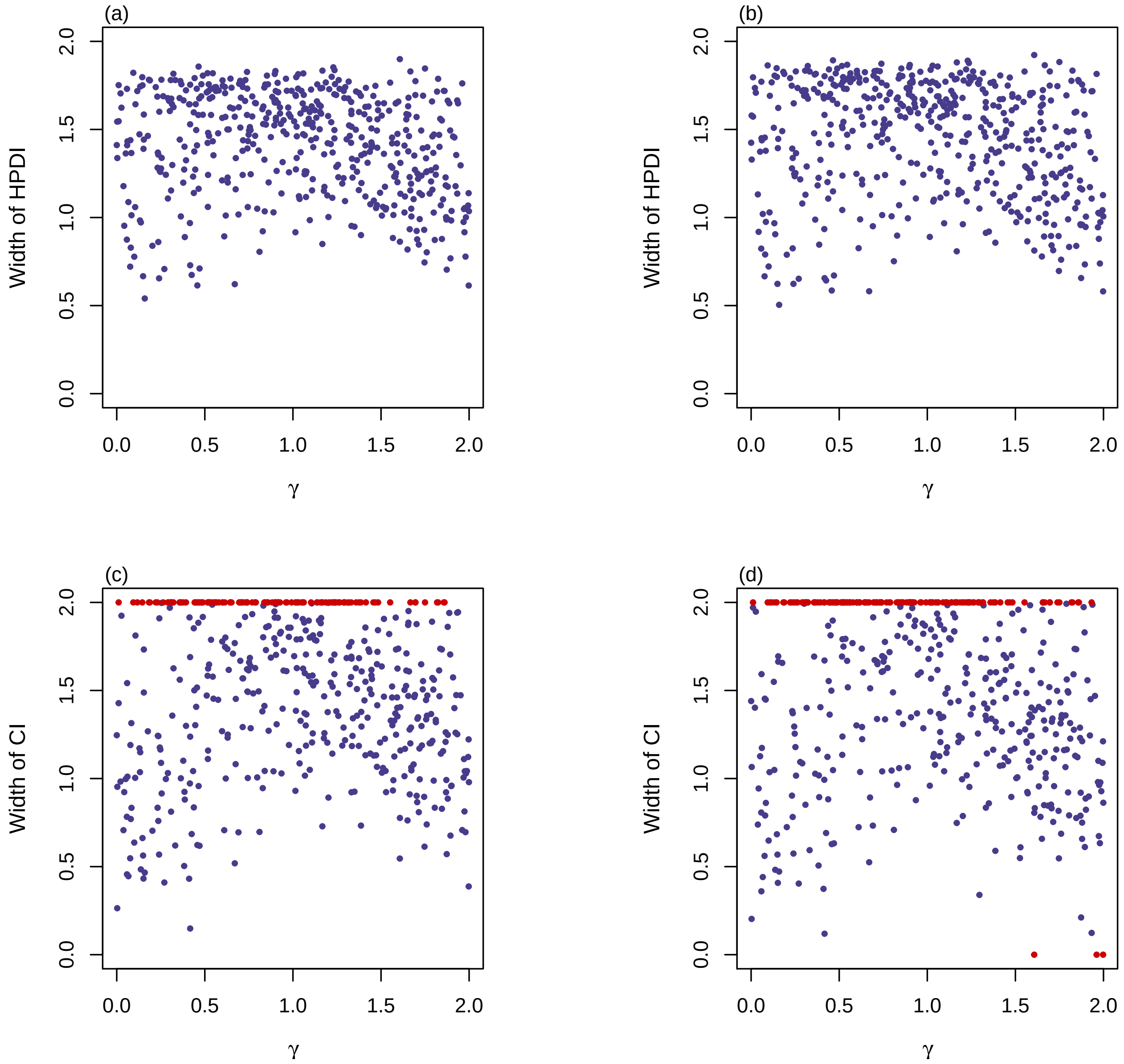

Supplementary Figure S24 Widths of HPDIs or CIs against true value of γ for qualitative trait with $n = 500$, MAF $= 0.3$ and $\rho = 0.05$. The red points represent the widths of the noninformative intervals or the empty sets. **(a)** BN method; **(b)** BU method; **(c)** PF method; **(d)** Fieller's method

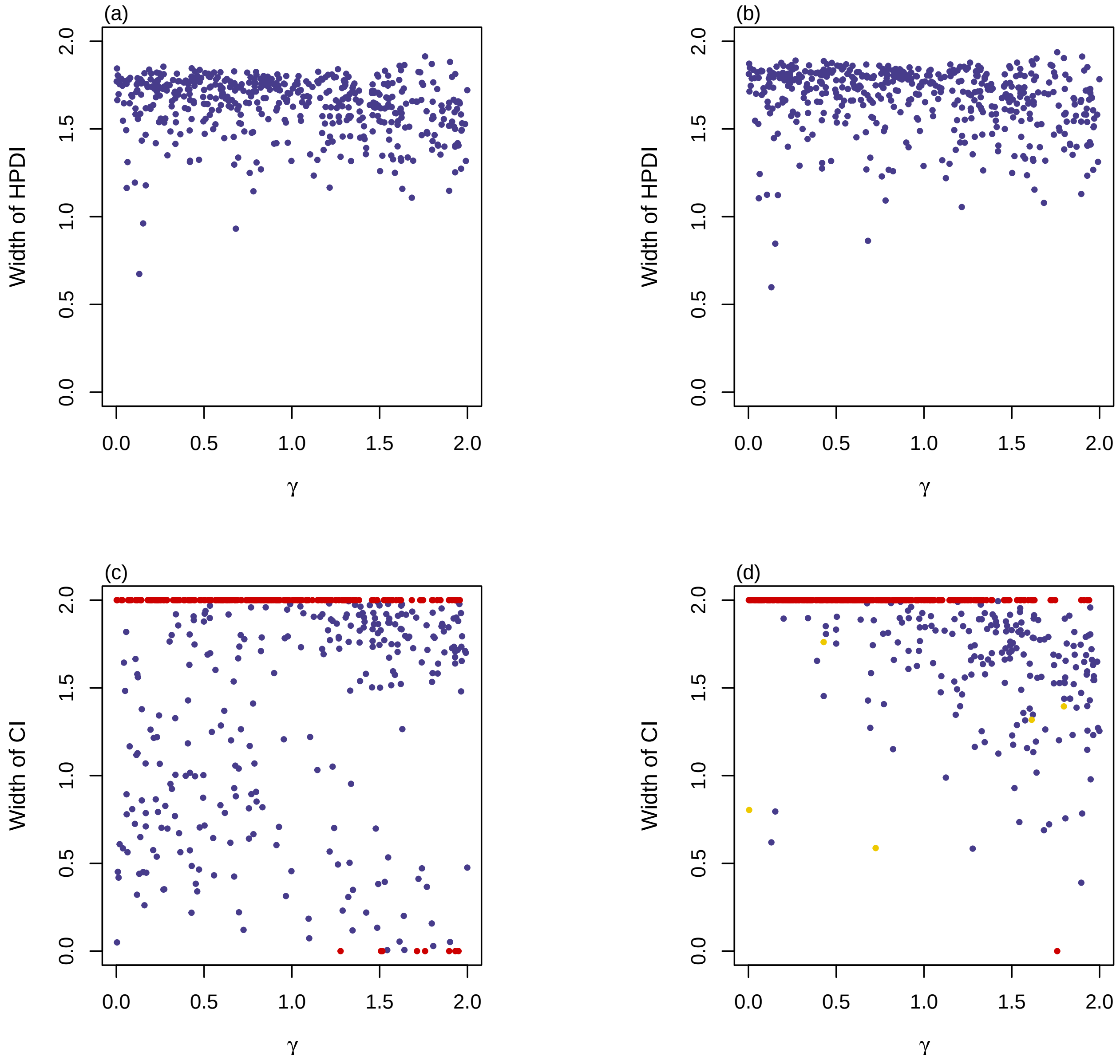

Supplementary Figure S25 Widths of HPDIs or CIs against true value of γ for qualitative trait with $n = 500$, MAF $= 0.1$ and $\rho = -0.05$. The red points represent the widths of the noninformative intervals or the empty sets, and the yellow points represent the widths of the discontinuous intervals. **(a)** BN method; **(b)** BU method; **(c)** PF method; **(d)** Fieller's method

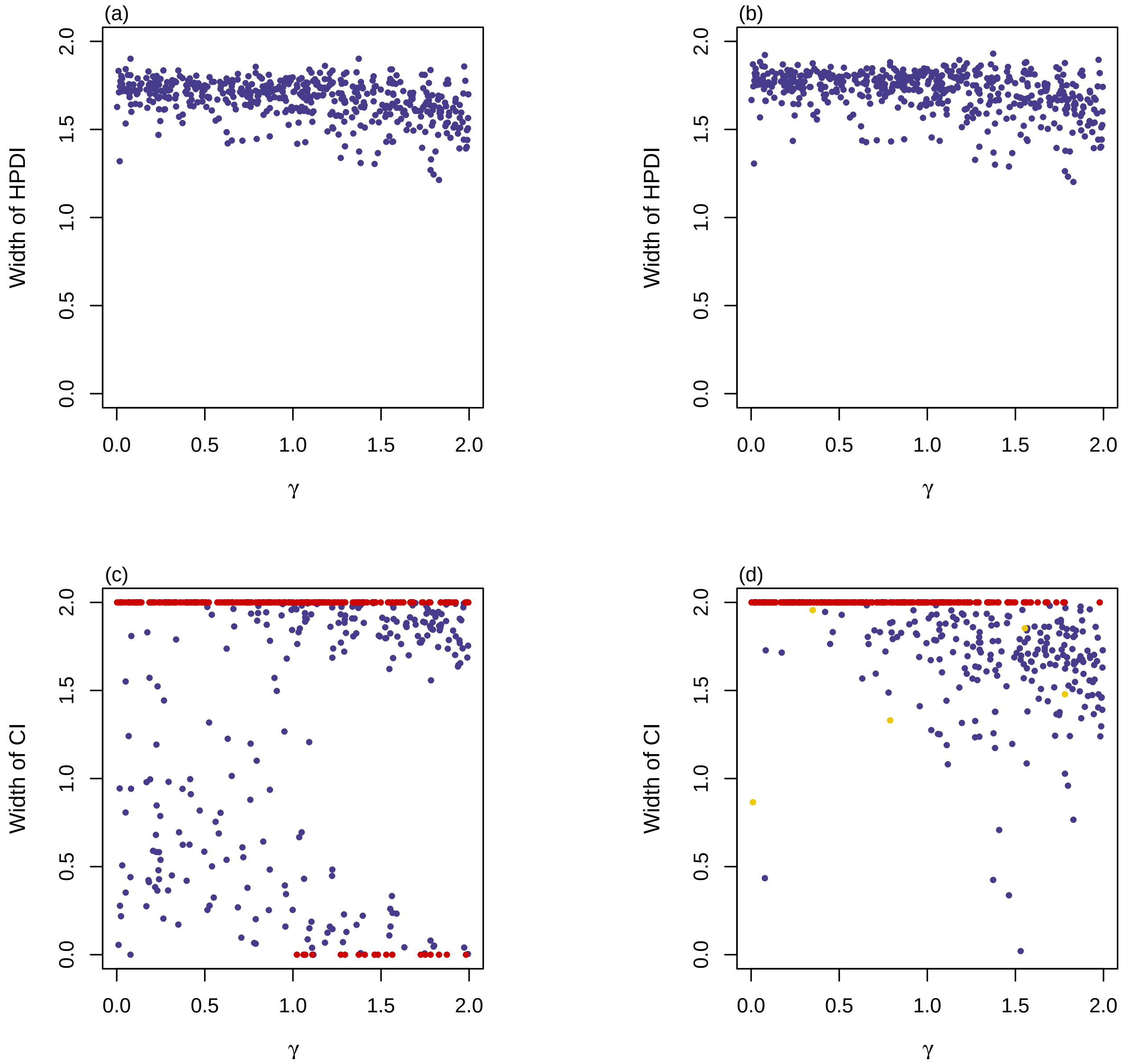

Supplementary Figure S26 Widths of HPDIs or CIs against true value of γ for qualitative trait with $n = 500$, MAF $= 0.1$ and $\rho = -0.05$. The red points represent the widths of the noninformative intervals or the empty sets, and the yellow points represent the widths of the discontinuous intervals. **(a)** BN method; **(b)** BU method; **(c)** PF method; **(d)** Fieller's method

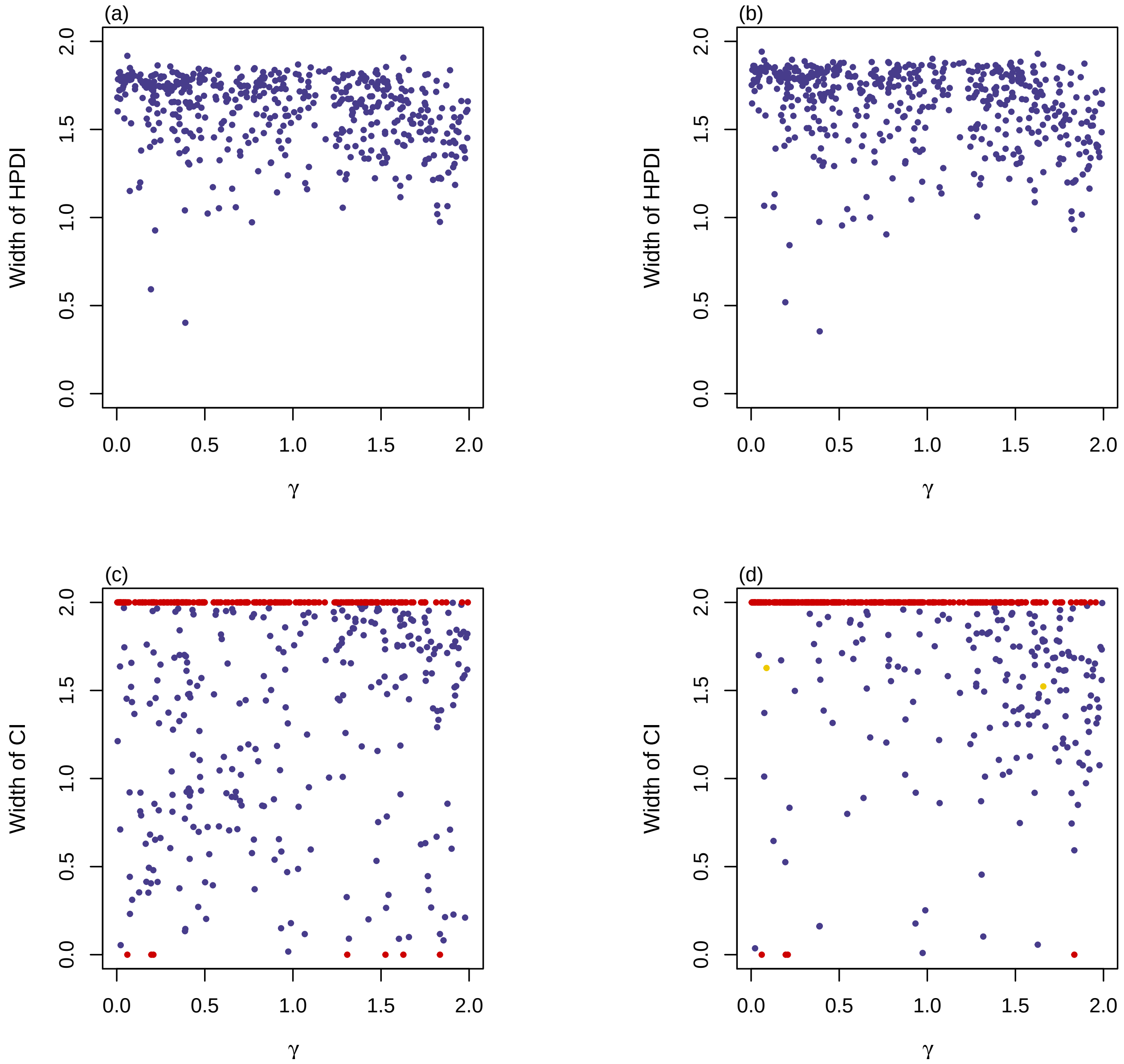

Supplementary Figure S27 Widths of HPDIs or CIs against true value of γ for qualitative trait with $n = 500$, MAF $= 0.1$ and $\rho = 0.05$. The red points represent the widths of the noninformative intervals or the empty sets, and the yellow points represent the widths of the discontinuous intervals. **(a)** BN method; **(b)** BU method; **(c)** PF method; **(d)** Fieller's method

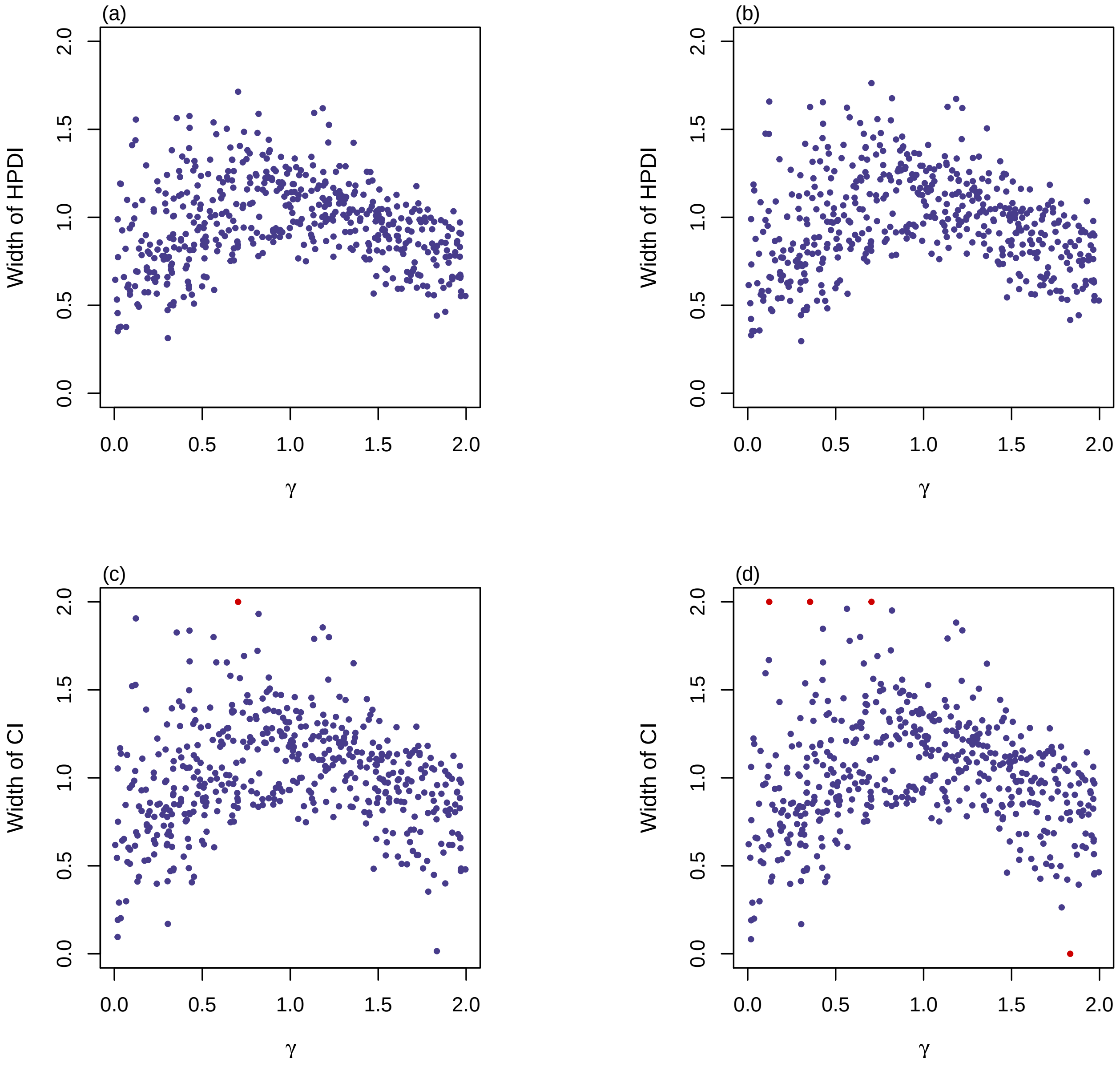

Supplementary Figure S28 Widths of HPDIs or CIs against true value of γ for qualitative trait with $n = 2000$, MAF $= 0.3$ and $\rho = 0$. The red points represent the widths of the noninformative intervals or the empty sets. **(a)** BN method; **(b)** BU method; **(c)** PF method; **(d)** Fieller's method

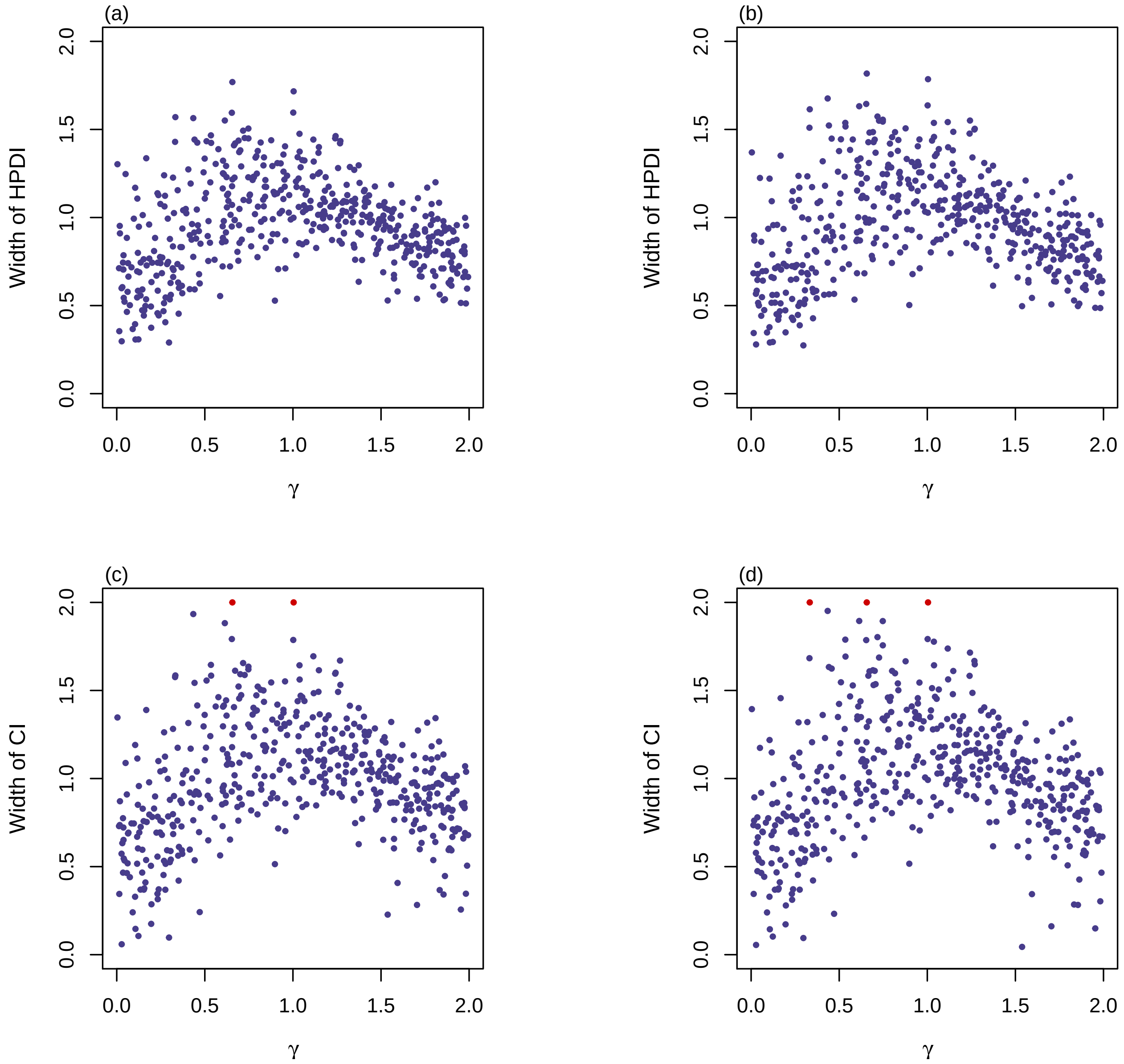

Supplementary Figure S29 Widths of HPDIs or CIs against true value of γ for qualitative trait with $n = 2000$, MAF $= 0.3$ and $\rho = -0.05$. The red points represent the widths of the noninformative intervals or the empty sets. **(a)** BN method; **(b)** BU method; **(c)** PF method; **(d)** Fieller's method

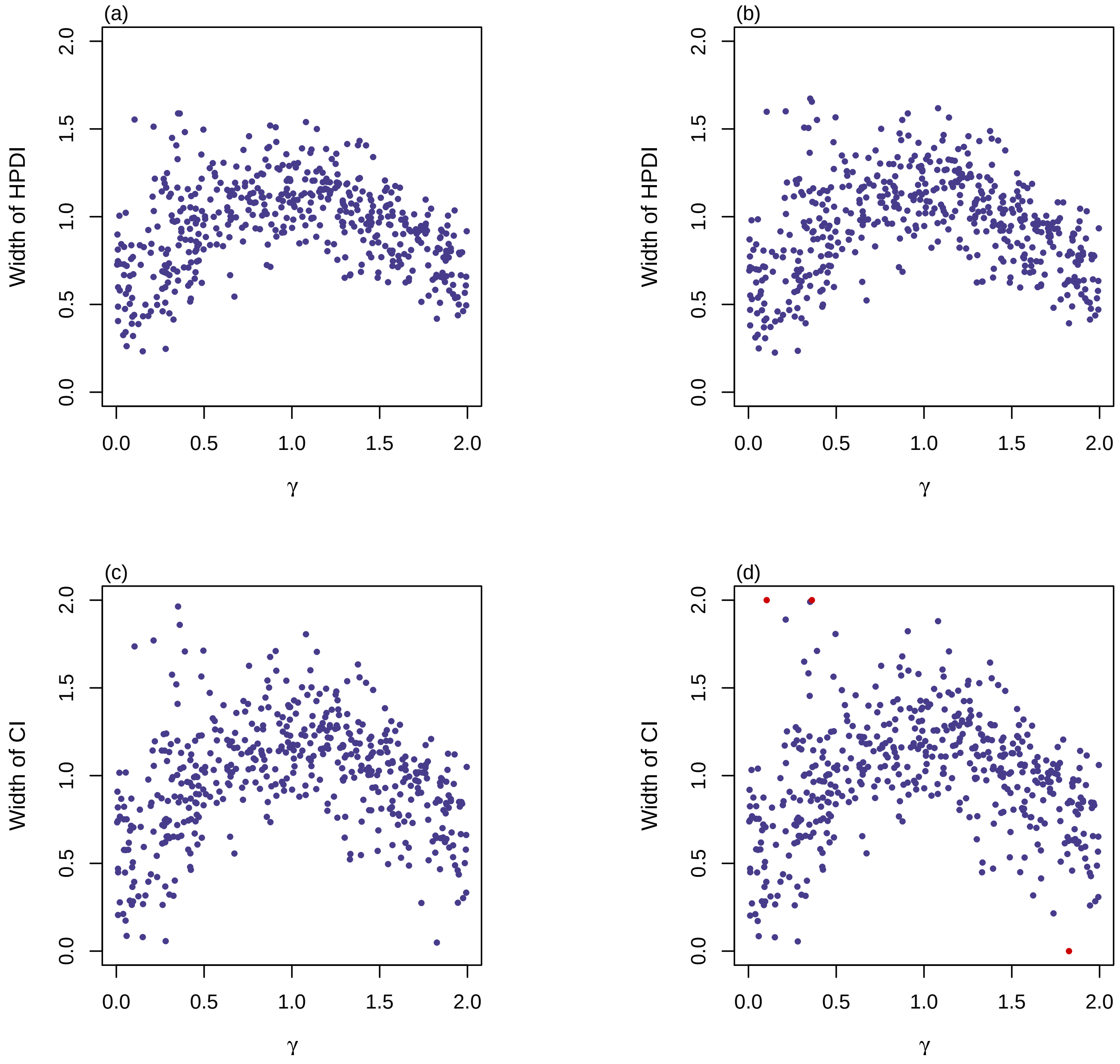

Supplementary Figure S30 Widths of HPDIs or CIs against true value of γ for qualitative trait with $n = 2000$, MAF $= 0.3$ and $\rho = 0.05$. The red points represent the widths of the noninformative intervals or the empty sets. **(a)** BN method; **(b)** BU method; **(c)** PF method; **(d)** Fieller's method

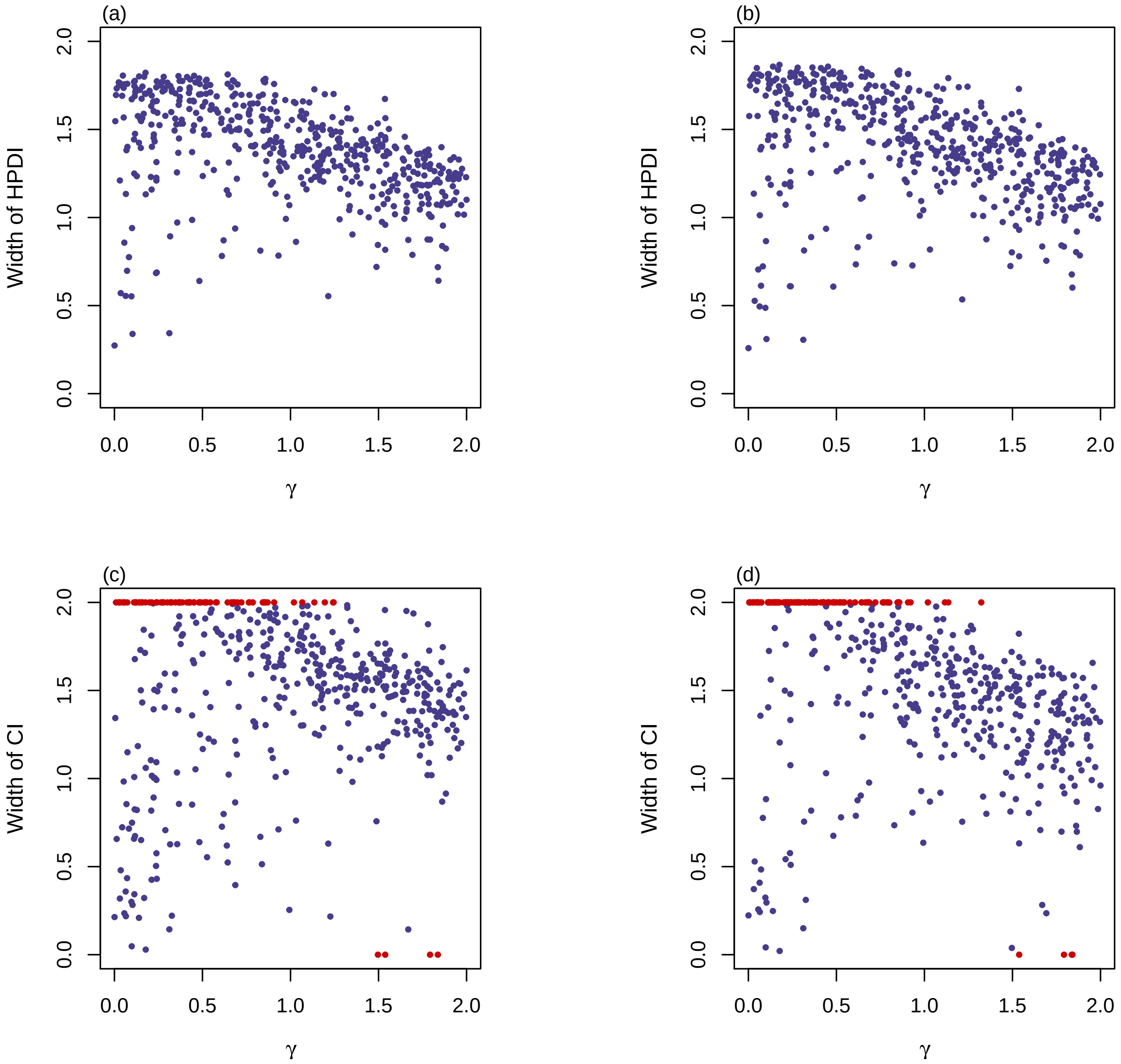

Supplementary Figure S31 Widths of HPDIs or CIs against true value of γ for qualitative trait with $n = 2000$, MAF $= 0.1$ and $\rho = 0$. The red points represent the widths of the noninformative intervals or the empty sets. **(a)** BN method; **(b)** BU method; **(c)** PF method; **(d)** Fieller's method

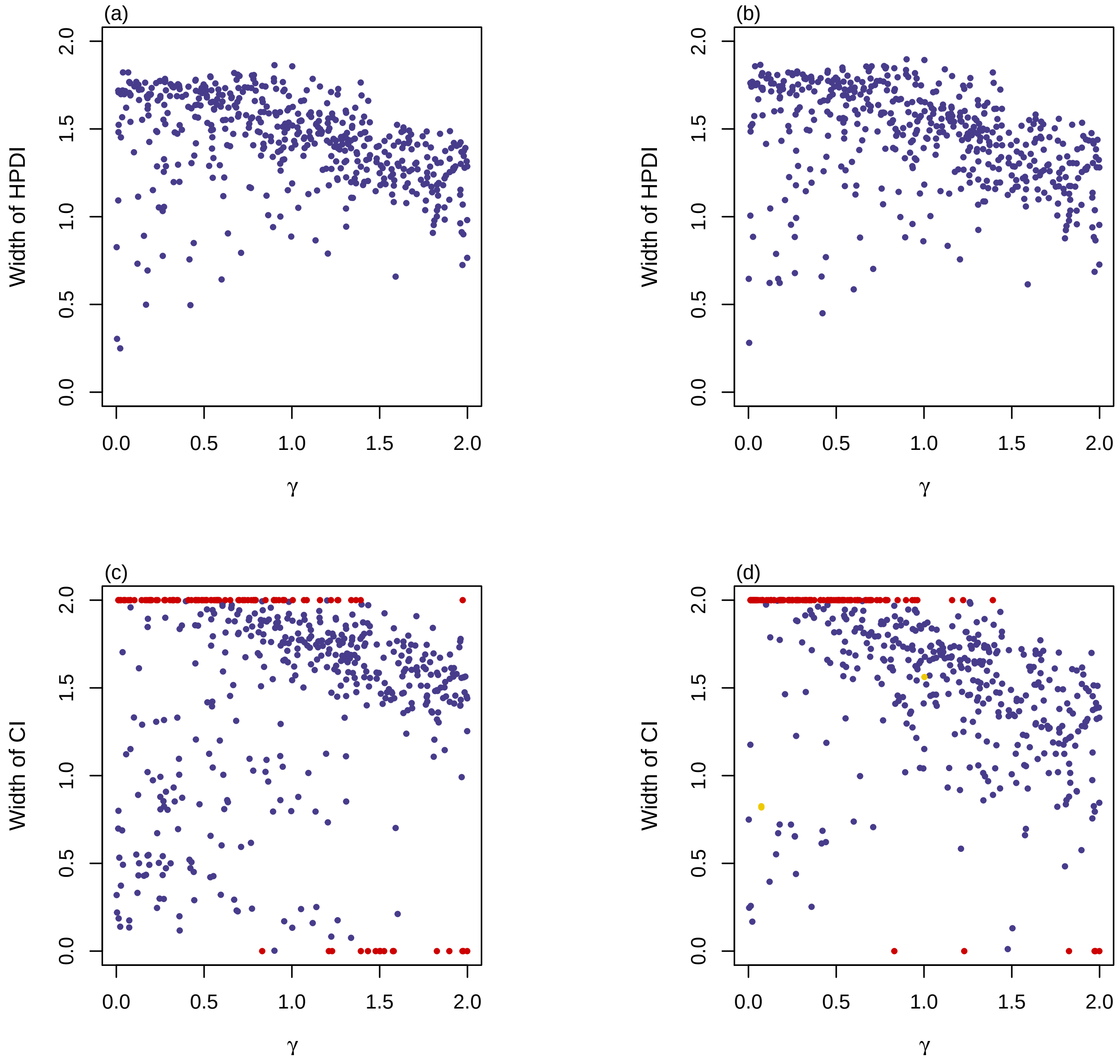

Supplementary Figure S32 Widths of HPDIs or CIs against true value of γ for qualitative trait with $n = 2000$, MAF $= 0.1$ and $\rho = -0.05$. The red points represent the widths of the noninformative intervals or the empty sets, and the yellow points represent the widths of the discontinuous intervals. **(a)** BN method; **(b)** BU method; **(c)** PF method; **(d)** Fieller's method

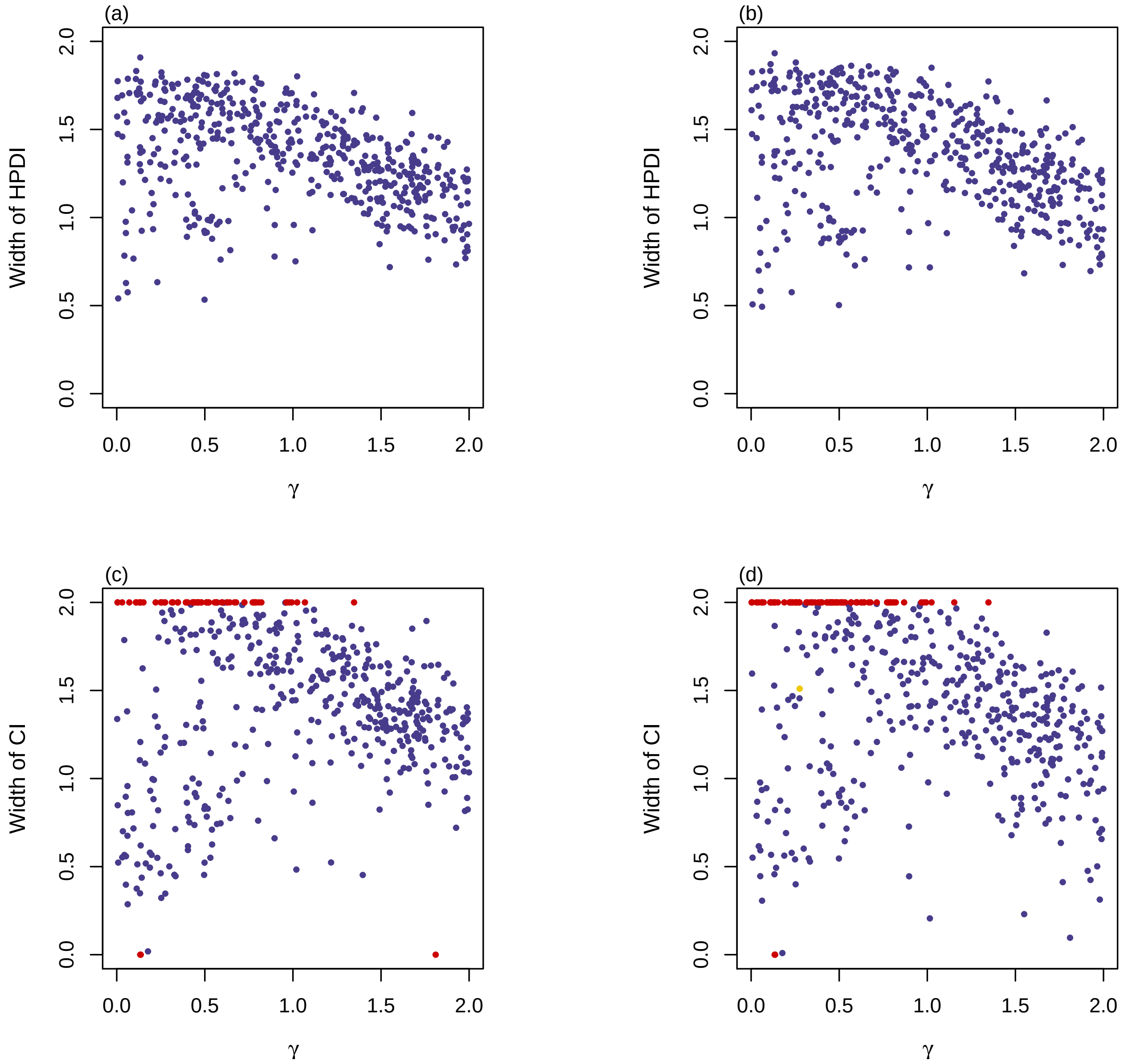

Supplementary Figure S33 Widths of HPDIs or CIs against true value of γ for qualitative trait with $n = 2000$, MAF $= 0.1$ and $\rho = 0.05$. The red points represent the widths of the noninformative intervals or the empty sets, and the yellow point represents the width of the discontinuous interval. **(a)** BN method; **(b)** BU method; **(c)** PF method; **(d)** Fieller's method

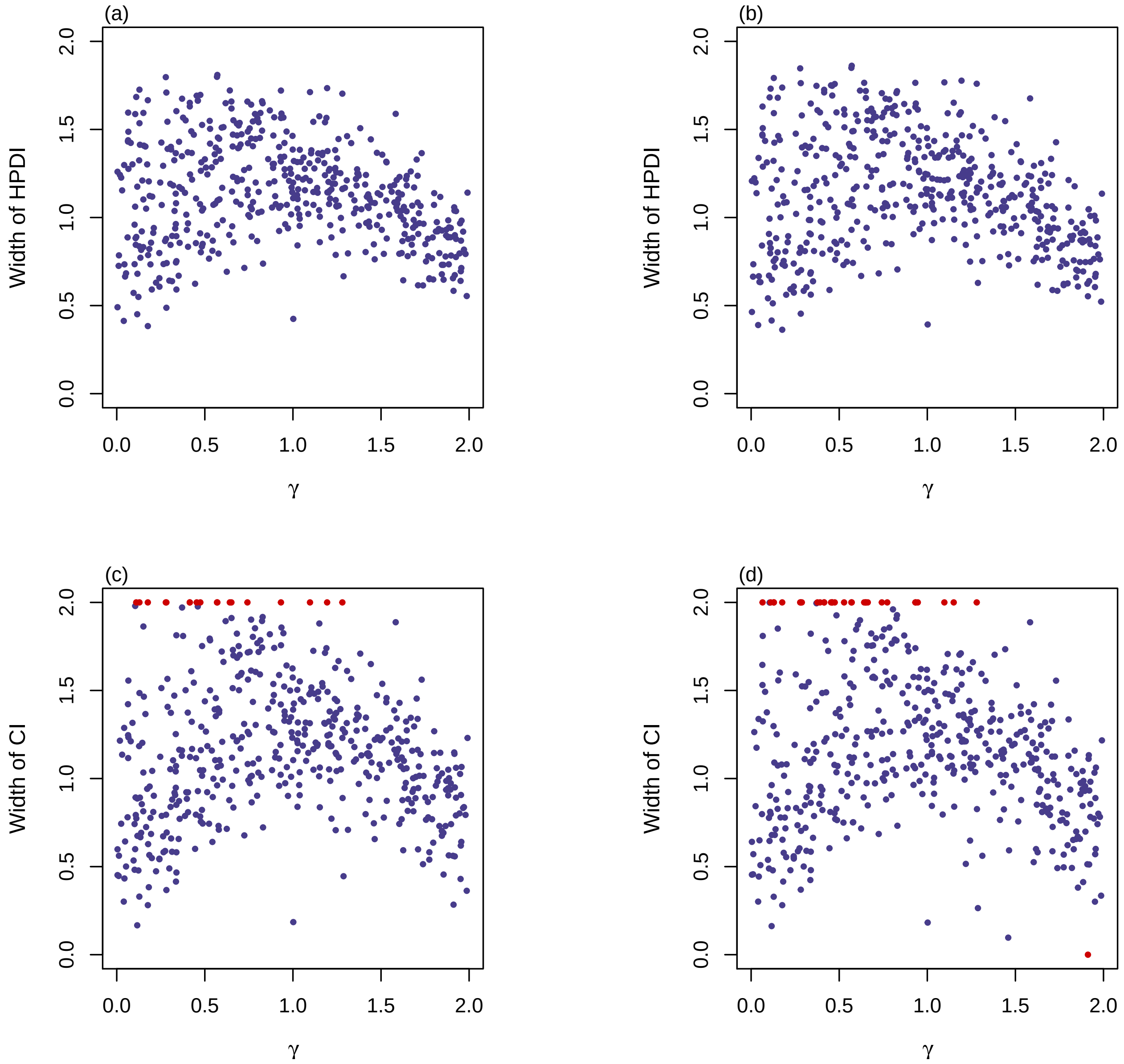

Supplementary Figure S34 Widths of HPDIs or CIs against true value of γ for quantitative trait when $(\sigma_0^2,\ \sigma_1^2,\ \sigma_2^2) = (1,\ 1.2,\ 1)$ with $n = 500$, MAF $= 0.3$ and $\rho = -0.05$. The red points represent the widths of the noninformative intervals or the empty sets. **(a)** BN method; **(b)** BU method; **(c)** PF method; **(d)** Fieller's method

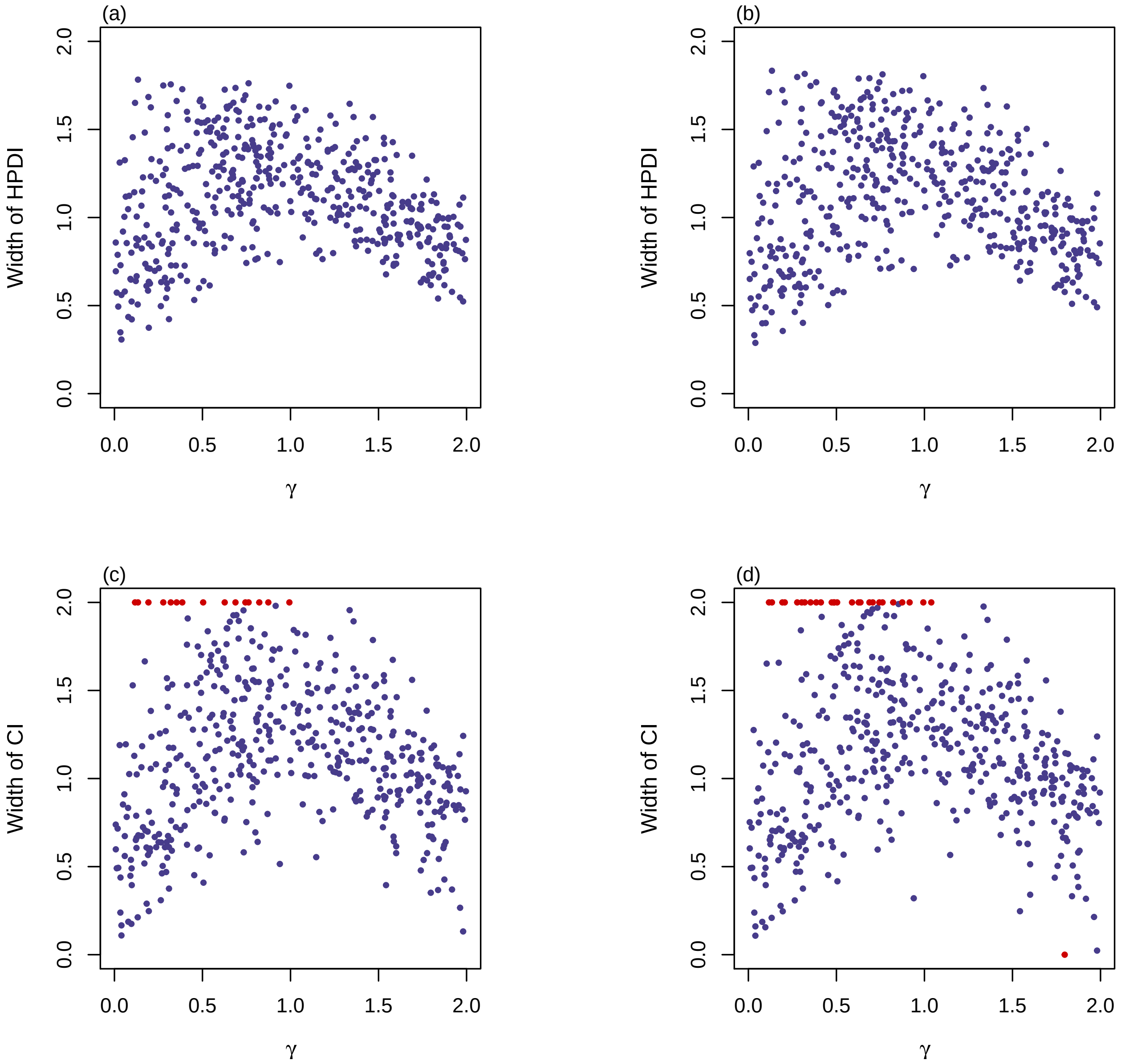

Supplementary Figure S35 Widths of HPDIs or CIs against true value of γ for quantitative trait when $(\sigma_0^2,\ \sigma_1^2,\ \sigma_2^2) = (1,\ 1.2,\ 1)$ with $n = 500$, MAF $= 0.3$ and $\rho = 0.05$. The red points represent the widths of the noninformative intervals or the empty sets. **(a)** BN method; **(b)** BU method; **(c)** PF method; **(d)** Fieller's method

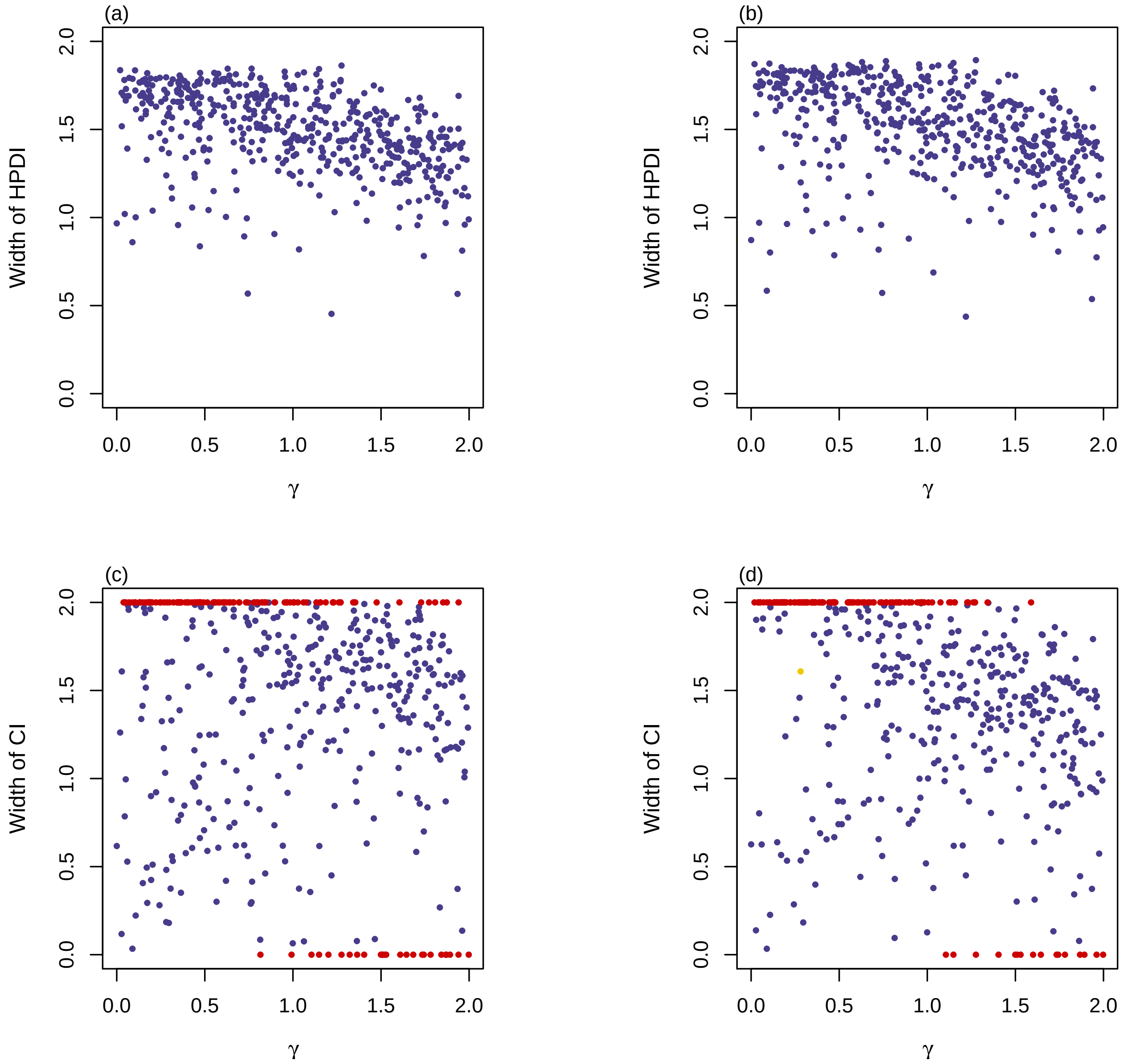

Supplementary Figure S36 Widths of HPDIs or CIs against true value of γ for quantitative trait when $(\sigma_0^2,\ \sigma_1^2,\ \sigma_2^2) = (1,\ 1.2,\ 1)$ with $n = 500$, MAF $= 0.1$ and $\rho = 0$. The red points represent the widths of the noninformative intervals or the empty sets, and the yellow point represents the width of the discontinuous interval. **(a)** BN method; **(b)** BU method; **(c)** PF method; **(d)** Fieller's method

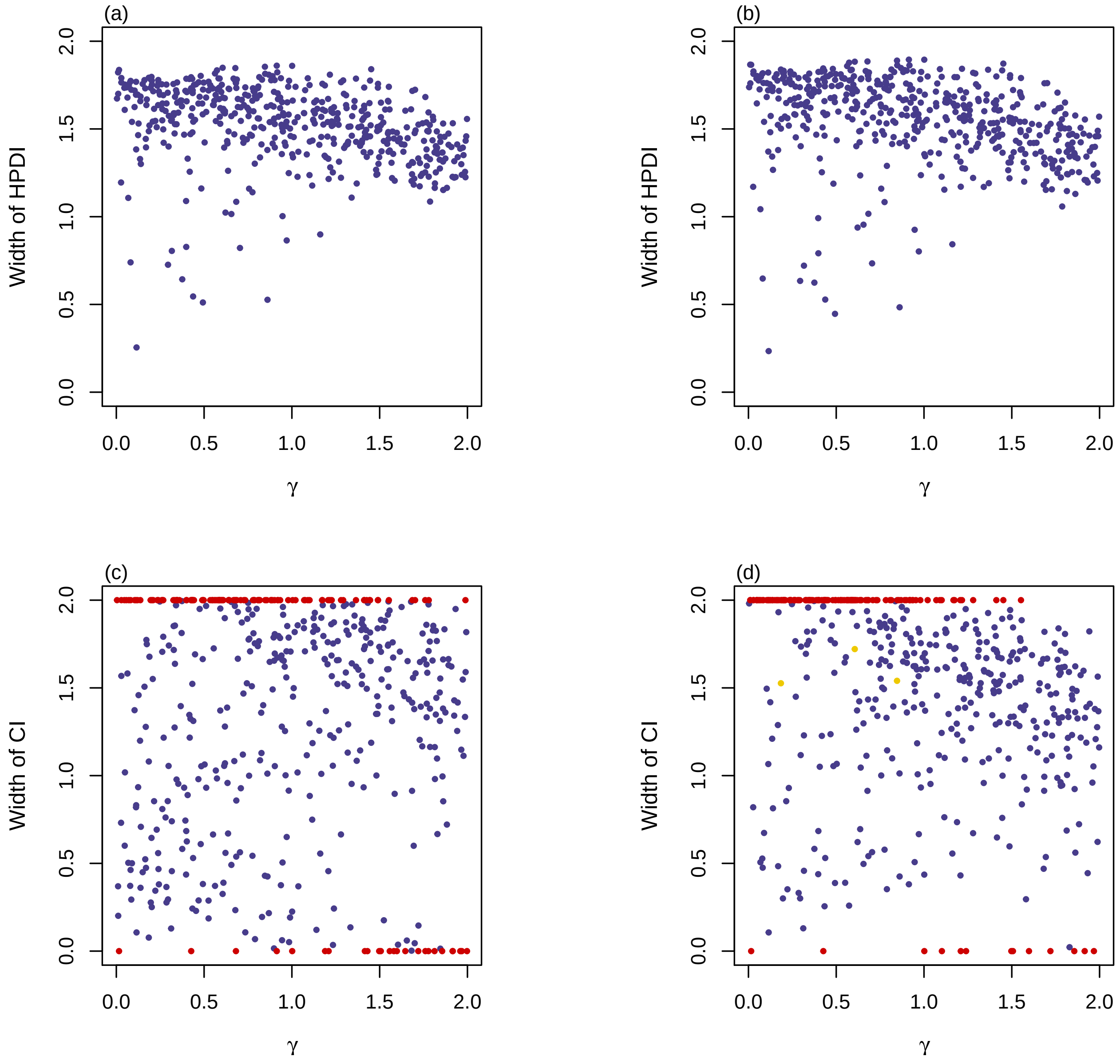

Supplementary Figure S37 Widths of HPDIs or CIs against true value of γ for quantitative trait when $(\sigma_0^2,\ \sigma_1^2,\ \sigma_2^2) = (1,\ 1.2,\ 1)$ with $n = 500$, MAF $= 0.1$ and $\rho = -0.05$. The red points represent the widths of the noninformative intervals or the empty sets, and the yellow points represent the widths of the discontinuous intervals. **(a)** BN method; **(b)** BU method; **(c)** PF method; **(d)** Fieller's method

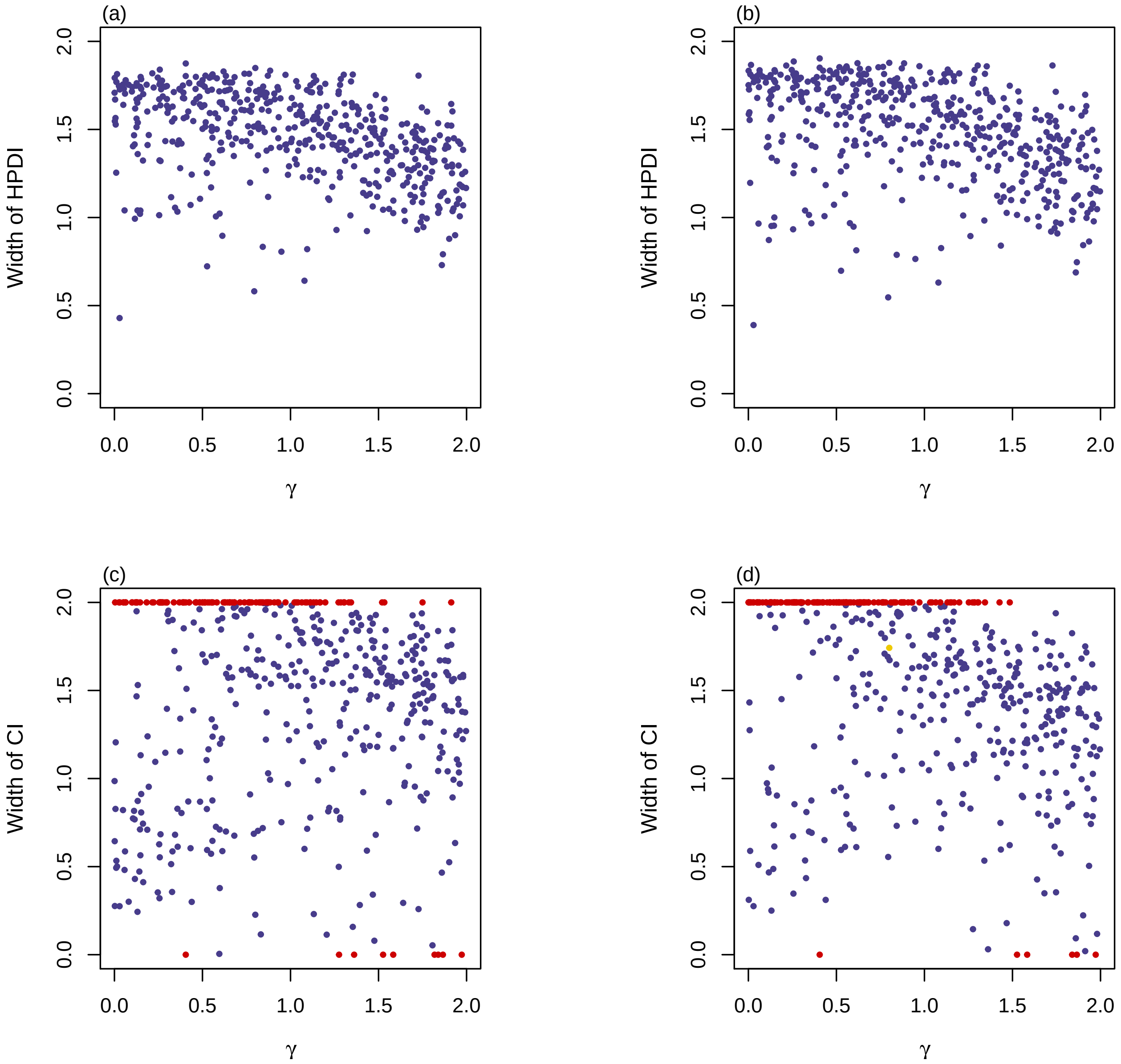

Supplementary Figure S38 Widths of HPDIs or CIs against true value of γ for quantitative trait when $(\sigma_0^2,\ \sigma_1^2,\ \sigma_2^2) = (1,\ 1.2,\ 1)$ with $n = 500$, MAF $= 0.1$ and $\rho = 0.05$. The red points represent the widths of the noninformative intervals or the empty sets, and the yellow point represents the width of the discontinuous interval. **(a)** BN method; **(b)** BU method; **(c)** PF method; **(d)** Fieller's method

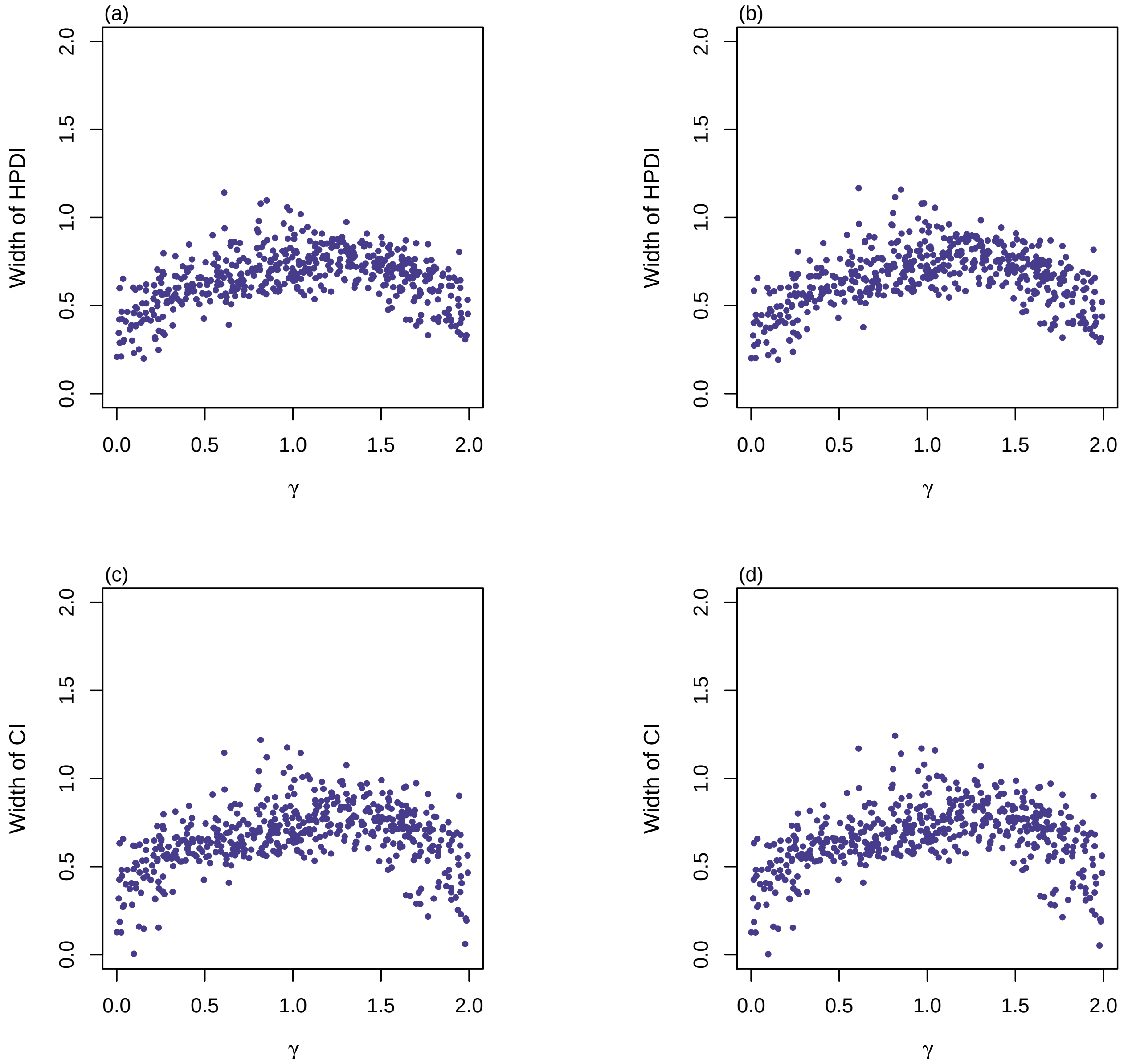

Supplementary Figure S39 Widths of HPDIs or CIs against true value of γ for quantitative trait when $(\sigma_0^2,\ \sigma_1^2,\ \sigma_2^2) = (1,\ 1.2,\ 1)$ with $n = 2000$, MAF $= 0.3$ and $\rho = 0$. **(a)** BN method; **(b)** BU method; **(c)** PF method; **(d)** Fieller's method

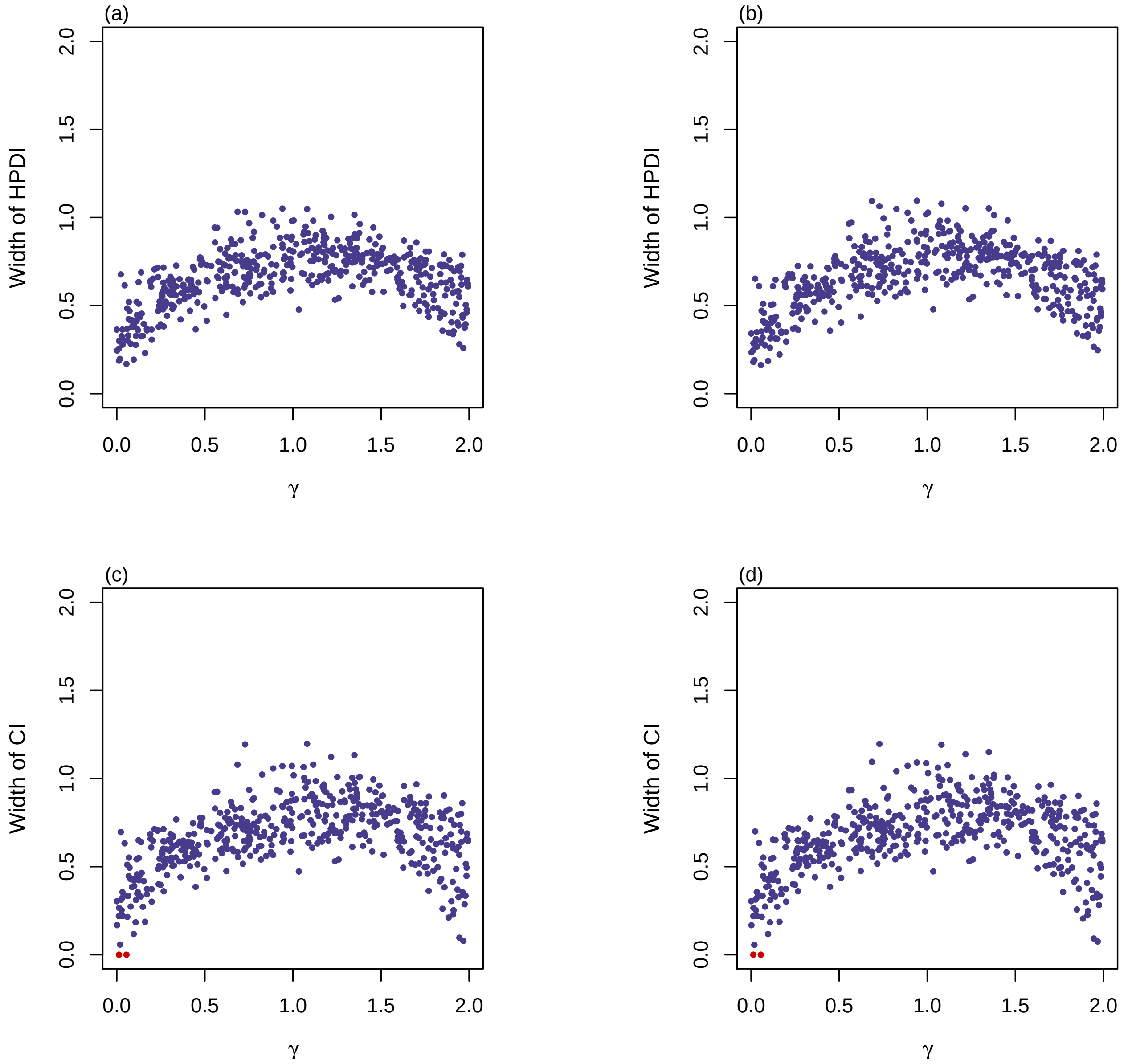

Supplementary Figure S40 Widths of HPDIs or CIs against true value of γ for quantitative trait when $(\sigma_0^2,\ \sigma_1^2,\ \sigma_2^2) = (1,\ 1.2,\ 1)$ with $n = 2000$, MAF $= 0.3$ and $\rho = -0.05$. The red points represent the widths of the noninformative intervals or the empty sets. **(a)** BN method; **(b)** BU method; **(c)** PF method; **(d)** Fieller's method

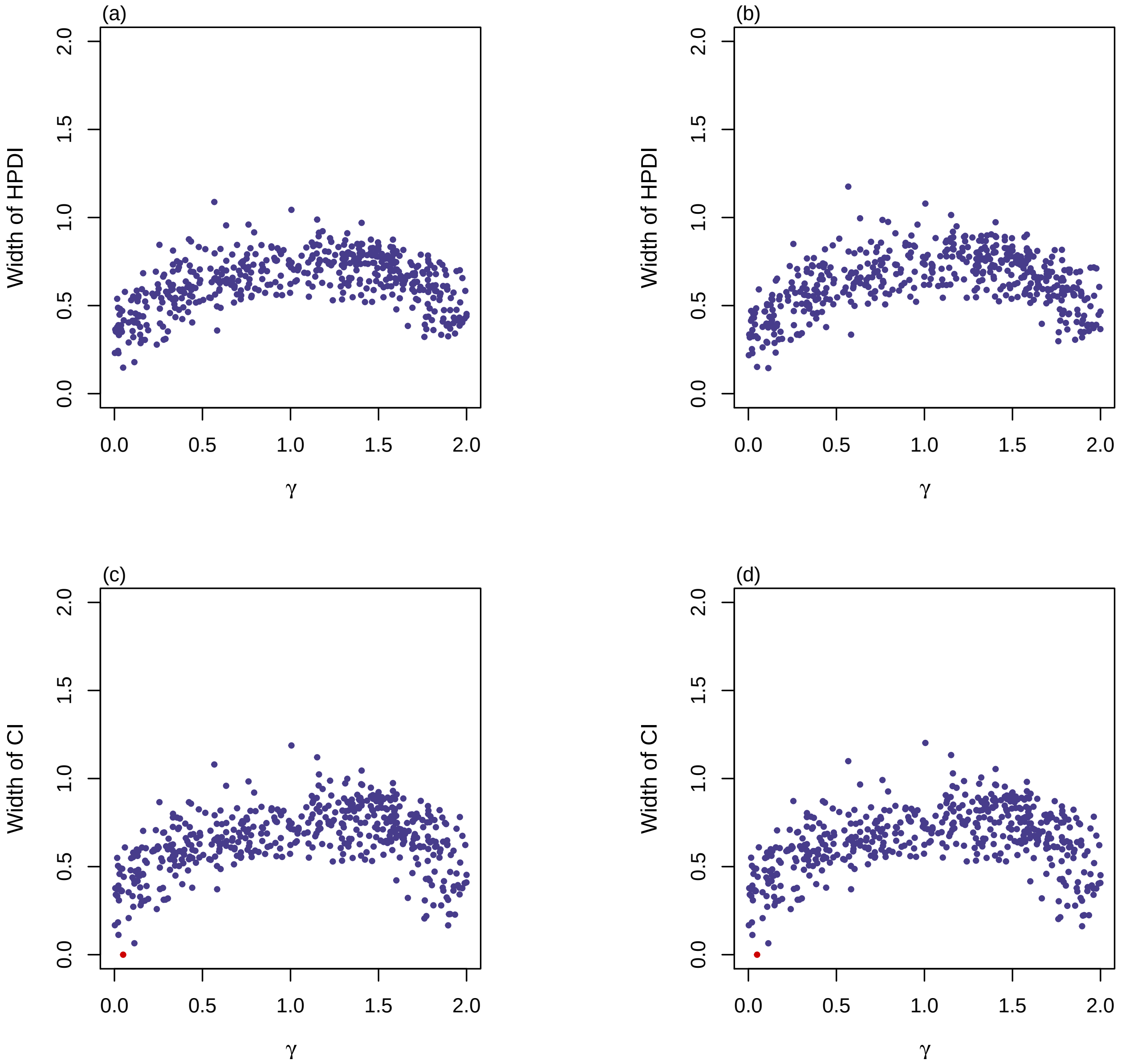

Supplementary Figure S41 Widths of HPDIs or CIs against true value of γ for quantitative trait when $(\sigma_0^2,\ \sigma_1^2,\ \sigma_2^2) = (1,\ 1.2,\ 1)$ with $n = 2000$, MAF $= 0.3$ and $\rho = 0.05$. The red points represent the widths of the noninformative intervals or the empty sets. **(a)** BN method; **(b)** BU method; **(c)** PF method; **(d)** Fieller's method

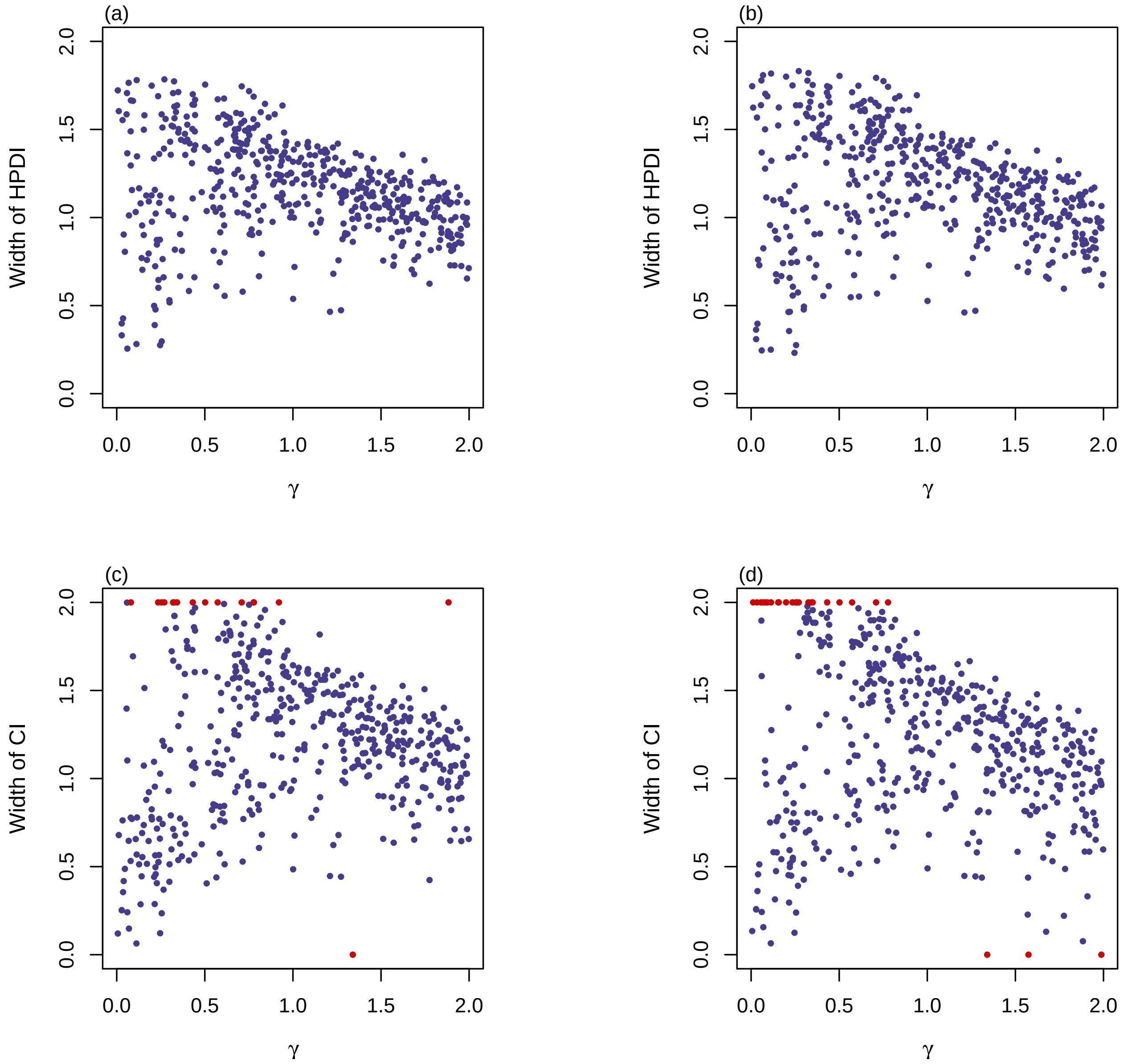

Supplementary Figure S42 Widths of HPDIs or CIs against true value of γ for quantitative trait when $(\sigma_0^2,\ \sigma_1^2,\ \sigma_2^2) = (1,\ 1.2,\ 1)$ with $n = 2000$, MAF $= 0.1$ and $\rho = 0$. The red points represent the widths of the noninformative intervals or the empty sets. **(a)** BN method; **(b)** BU method; **(c)** PF method; **(d)** Fieller's method

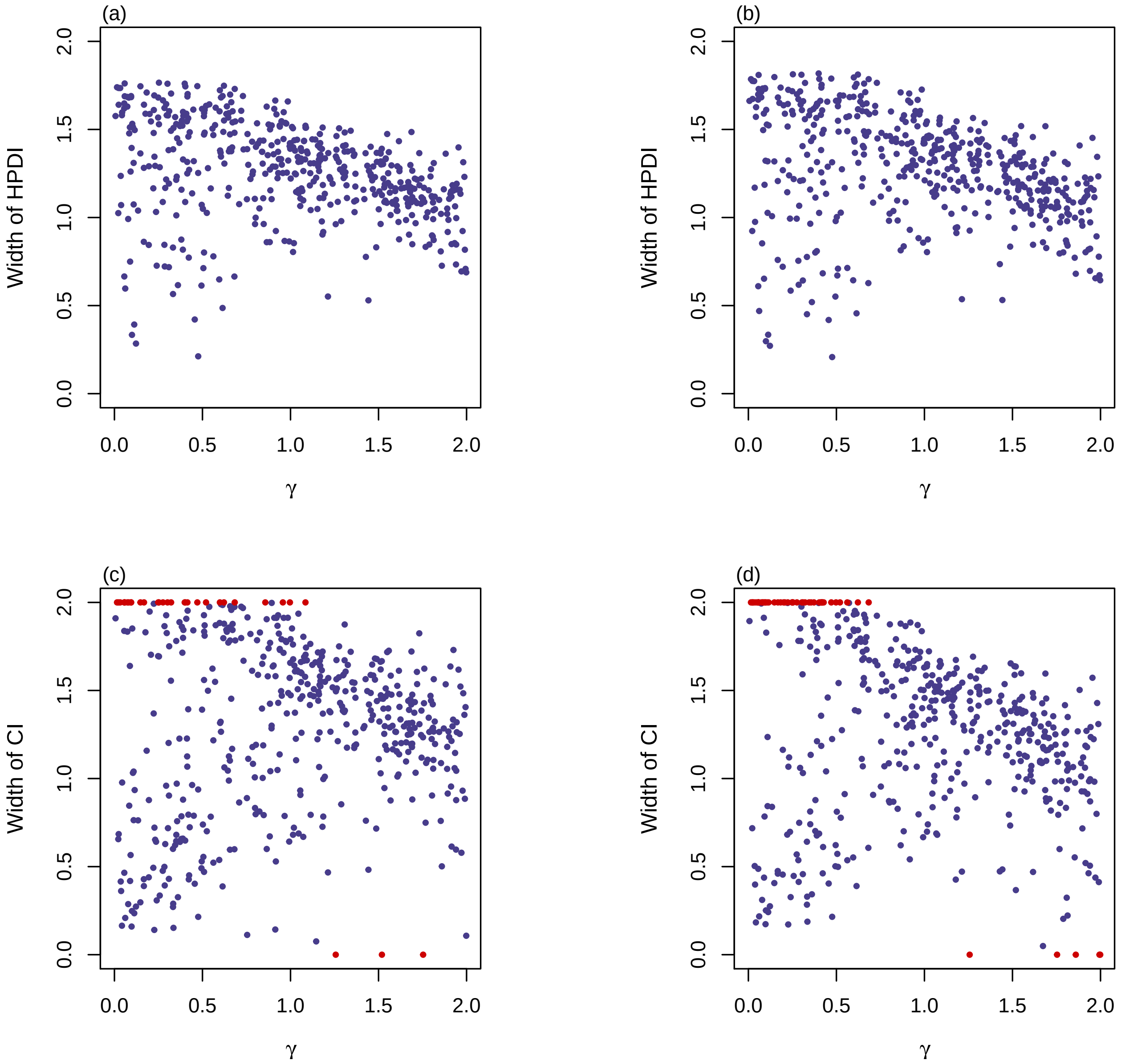

Supplementary Figure S43 Widths of HPDIs or CIs against true value of γ for quantitative trait when $(\sigma_0^2,\ \sigma_1^2,\ \sigma_2^2) = (1,\ 1.2,\ 1)$ with $n = 2000$, MAF $= 0.1$ and $\rho = -0.05$. The red points represent the widths of the noninformative intervals or the empty sets. **(a)** BN method; **(b)** BU method; **(c)** PF method; **(d)** Fieller's method

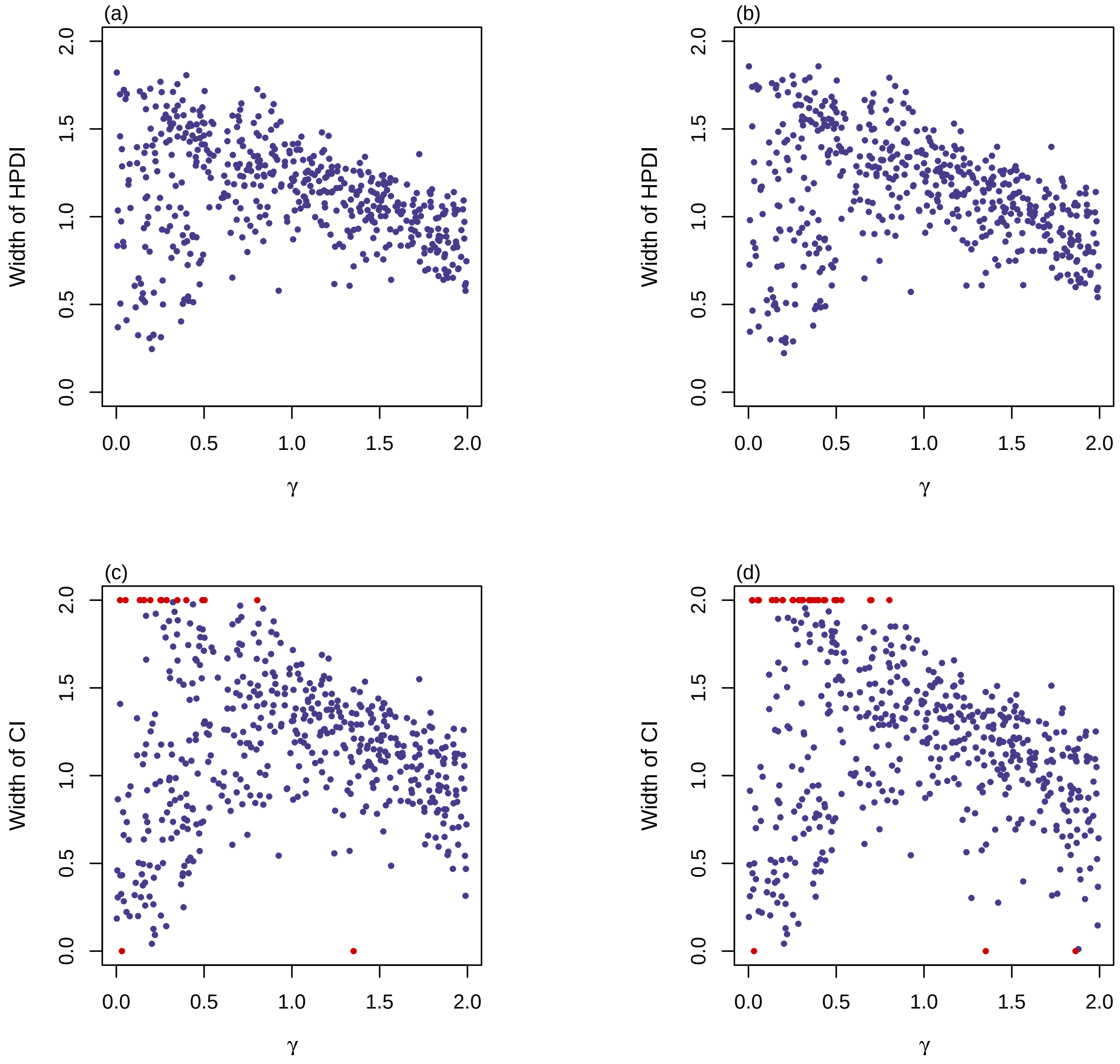

Supplementary Figure S44 Widths of HPDIs or CIs against true value of γ for quantitative trait when $(\sigma_0^2,\ \sigma_1^2,\ \sigma_2^2) = (1,\ 1.2,\ 1)$ with $n = 2000$, MAF $= 0.1$ and $\rho = 0.05$. The red points represent the widths of the noninformative intervals or the empty sets. **(a)** BN method; **(b)** BU method; **(c)** PF method; **(d)** Fieller's method
