# Supplementary material for: BEXCIS: Bayesian methods for estimating the degree of the skewness of X chromosome inactivation
Source: BMC Bioinformatics. 2022 May 24;23:193. doi: 10.1186/s12859-022-04721-y (PMC9128296; doi:10.1186/s12859-022-04721-y)
Supplement: Supplementary file 3 — Additional file 3. Table S1. WSD's and WIQR's of BN, BU, PF and Fieller's methods for qualitative trait and quantitative trait with (σ02, σ12, σ22)=(1, 1.2, 1). Table S2. Proportions of extreme values of ${\hat{\gamma }}_{PF}$γ^PF and ${\hat{\gamma }}_{F}$γ^F for quantitative trait when (σ02, σ12, σ22)=(4, 4.8, 4). Table S3. MSEs of ${\hat{\gamma }}_{BN}$γ^BN, ${\hat{\gamma }}_{BU}$γ^BU, ${\hat{\gamma }}_{PF}$γ^PF and ${\hat{\gamma }}_{F}$γ^F for quantitative trait when (σ02, σ12, σ22)=(4, 4.8, 4). Table S4. NPs, EPs and DPs for P [file 12859_2022_4721_MOESM3_ESM.pdf]

Additional file 3: Supplementary Tables

Table S1. Standard deviation and interquartile range of the widths of the highest posterior density intervals or confidence intervals (respectively denoted as W_{SD} and W_{IQR}) of Bayesian method with normal prior (BN), Bayesian method with uniform prior (BU), penalized Fieller's (PF) and Fieller's methods among 500 replicates for qualitative trait and quantitative trait with $(\sigma_0^2,\ \sigma_1^2,\ \sigma_2^2) = (1,\ 1.2,\ 1)$

				W_{SD}				W_{IQR}			
Trait	n	MAF	ρ	BN	BU	PF	Fieller	BN	BU	PF	Fieller
Qualitative	500	0.3	0	0.2881	0.3194	0.4391	0.4773	0.4301	0.4732	0.7140	0.7595
			-0.05	0.3022	0.3345	0.4577	0.5183	0.4575	0.5139	0.7543	0.8160
			0.05	0.2972	0.3303	0.4461	0.4907	0.4628	0.5221	0.7723	0.8743
		0.1	0	0.1642	0.1836	0.6031	0.2981	0.2004	0.2083	0.7195	0.2372
			-0.05	0.1113	0.1204	0.7025	0.2680	0.1363	0.1387	0.6949	0.2719
			0.05	0.2062	0.2296	0.5921	0.4096	0.2753	0.2945	0.7329	0.3140
	2000	0.3	0	0.2447	0.2703	0.3056	0.3187	0.3192	0.3651	0.3826	0.3980
			-0.05	0.2614	0.2894	0.3284	0.3489	0.3354	0.3871	0.4056	0.4264
			0.05	0.2558	0.2804	0.3224	0.3357	0.3531	0.3954	0.4027	0.4258
		0.1	0	0.2770	0.3047	0.4607	0.4555	0.3706	0.4079	0.5256	0.7051
			-0.05	0.2611	0.2862	0.5753	0.4379	0.3580	0.4151	0.6017	0.5898
			0.05	0.2718	0.3047	0.4432	0.4531	0.4035	0.4669	0.5953	0.6837
Quantitative	500	0.3	0	0.2987	0.3302	0.4054	0.4347	0.4252	0.4890	0.5387	0.5668
			-0.05	0.2892	0.3214	0.3933	0.4265	0.4127	0.4765	0.5067	0.5830
			0.05	0.3047	0.3377	0.4085	0.4360	0.4480	0.5333	0.5497	0.5833
		0.1	0	0.2343	0.2612	0.6025	0.5423	0.3239	0.3600	0.8201	0.7886
			-0.05	0.2238	0.2453	0.6571	0.5269	0.2724	0.3029	1.0253	0.7639
			0.05	0.2517	0.2803	0.5456	0.5076	0.3697	0.4053	0.7974	0.7951
	2000	0.3	0	0.1539	0.1657	0.1803	0.1820	0.1779	0.1975	0.1962	0.1991
			-0.05	0.1659	0.1783	0.1953	0.1970	0.1995	0.2195	0.2250	0.2282
			0.05	0.1522	0.1641	0.1790	0.1803	0.2002	0.2156	0.2235	0.2222
		0.1	0	0.2875	0.3174	0.4169	0.4521	0.3661	0.4312	0.5491	0.6162
			-0.05	0.2759	0.3051	0.4902	0.4897	0.3608	0.3891	0.6682	0.6535
			0.05	0.3012	0.3287	0.4203	0.4577	0.3963	0.4475	0.5226	0.5791

Table S2. Proportions (in %) of extreme values (0 or 2) of $\hat{\gamma}_{PF}$ and $\hat{\gamma}_F$ among 500 replicates for quantitative trait when $(\sigma_0^2,\ \sigma_1^2,\ \sigma_2^2) = (4,\ 4.8,\ 4)$

			$\hat{\gamma}_{PF}$			$\hat{\gamma}_F$		
n	MAF	ρ	0	2	Total	0	2	Total
500	0.3	0	14.6	15.0	29.6	14.6	19.2	33.8
		-0.05	15.4	16.6	32.0	15.4	20.6	36.0
		0.05	15.8	14.0	29.8	15.8	20.6	36.4
	0.1	0	33.0	6.0	39.0	33.0	11.8	44.8
		-0.05	34.8	4.6	39.4	34.8	13.8	48.6
		0.05	27.8	7.6	35.4	27.8	15.8	43.6
2000	0.3	0	5.6	10.8	16.4	5.6	11.8	17.4
		-0.05	6.8	9.2	16.0	6.8	9.8	16.6
		0.05	6.6	10.4	17.0	6.6	10.6	17.2
	0.1	0	13.8	10.8	24.6	13.8	17.0	30.8
		-0.05	24.4	6.2	30.6	24.4	15.6	40.0
		0.05	14.0	12.8	26.8	14.0	18.8	32.8

Table S3. Mean squared errors (MSEs) of point estimates $\hat{\gamma}_{BN}$, $\hat{\gamma}_{BU}$, $\hat{\gamma}_{PF}$ and $\hat{\gamma}_{F}$ among 500 replicates for quantitative trait when $(\sigma_0^2,\ \sigma_1^2,\ \sigma_2^2) = (4,\ 4.8,\ 4)$

n	MAF	ρ	$\hat{\gamma}_{BN}$	$\hat{\gamma}_{BU}$	$\hat{\gamma}_{PF}$	$\hat{\gamma}_{F}$
500	0.3	0	0.2270	0.2750	0.3457	0.3807
		-0.05	0.2106	0.2603	0.3366	0.3790
		0.05	0.2028	0.2342	0.3203	0.3605
	0.1	0	0.4612	0.5764	0.7257	0.7812
		-0.05	0.4847	0.5820	0.7138	0.7630
		0.05	0.4446	0.5382	0.6334	0.7068
2000	0.3	0	0.1052	0.1193	0.1182	0.1195
		-0.05	0.0933	0.1036	0.1193	0.1212
		0.05	0.0959	0.1003	0.1041	0.1054
	0.1	0	0.2171	0.2378	0.3659	0.4251
		-0.05	0.2870	0.3186	0.4922	0.5701
		0.05	0.2059	0.2338	0.3709	0.4303

Table S4. Proportions (in %) of the noninformative intervals (NP), empty sets (EP) and discontinuous intervals (DP) of the 95% confidence intervals for the penalized Fieller's (PF) and Fieller's methods based on 500 replicates for quantitative trait when $(\sigma_0^2,\ \sigma_1^2,\ \sigma_2^2) = (4,\ 4.8,\ 4)$

			PF			Fieller		
n	MAF	ρ	NP	EP	DP	NP	EP	DP
500	0.3	0	37.2	0.2	0.0	46.2	0.8	0.6
		-0.05	35.6	0.2	0.0	45.6	0.8	0.2
		0.05	36.0	0.0	0.0	44.6	0.8	0.4
	0.1	0	43.0	4.4	0.0	55.4	3.6	0.6
		-0.05	40.2	5.0	0.0	56.2	3.4	1.6
		0.05	47.6	2.2	0.0	57.6	1.6	1.0
2000	0.3	0	2.2	0.2	0.0	3.0	0.2	0.0
		-0.05	3.4	0.4	0.0	4.8	0.4	0.0
		0.05	1.8	0.2	0.0	3.4	0.2	0.0
	0.1	0	25.8	3.4	0.0	30.6	1.2	0.2
		-0.05	24.0	5.2	0.0	31.4	1.6	0.8
		0.05	27.8	1.8	0.0	33.4	1.6	0.2

Table S5. Coverage probabilities (CPs, in %) of Bayesian method with normal prior (BN), Bayeisan method with uniform prior (BU), penalized Fieller's (PF) and Fieller's methods among 500 replicates for quantitative trait when $(\sigma_0^2,\ \sigma_1^2,\ \sigma_2^2) = (4,\ 4.8,\ 4)$[a]

n	MAF	ρ	BN	BU	PF	Fieller
500	0.3	0	93.6	95.0	95.8	95.8
		-0.05	96.0	96.4	95.8	95.4
		0.05	96.0	95.8	95.6	95.4
	0.1	0	89.8	91.0	74.6	87.6
		-0.05	90.0	90.8	72.4	88.6
		0.05	91.4	90.4	82.4	92.8
2000	0.3	0	95.2	95.2	94.8	93.6
		-0.05	95.8	96.4	95.4	96.2
		0.05	95.0	95.2	94.8	94.2
	0.1	0	95.8	96.0	89.8	93.4
		-0.05	94.2	96.0	78.4	90.0
		0.05	94.8	95.8	92.2	93.6

[a]The empirical CP should be between 93.05% and 96.95% ($0.95 \pm 2 \times \sqrt{\frac{0.95\times0.05}{500}}$) with 95% probability.

Table S6. Mean, median, standard deviation and interquartile range of the widths of the highest posterior density intervals or confidence intervals (respectively denoted as W_{mean}, W_{median}, W_{SD} and W_{IQR}) of Bayesian method with normal prior (BN), Bayesian method with uniform prior (BU), penalized Fieller's (PF) and Fieller's methods among 500 replicates for quantitative trait when $(\sigma_0^2,\ \sigma_1^2,\ \sigma_2^2) = (4,\ 4.8,\ 4)$

			W_{mean}				W_{median}				W_{SD}				W_{IQR}			
n	MAF	ρ	BN	BU	PF	Fieller	BN	BU	PF	Fieller	BN	BU	PF	Fieller	BN	BU	PF	Fieller
500	0.3	0	1.5487	1.5801	1.6045	1.6220	1.6307	1.6754	1.7496	1.8686	0.2577	0.2835	0.4488	0.4820	0.3303	0.3587	0.6838	0.6515
		-0.05	1.5606	1.5920	1.6120	1.6455	1.6377	1.6920	1.7584	1.9083	0.2469	0.2731	0.4348	0.4619	0.3389	0.3619	0.6688	0.6436
		0.05	1.5267	1.5541	1.5785	1.5952	1.5892	1.6352	1.7245	1.8691	0.2568	0.2835	0.4669	0.5036	0.3869	0.4278	0.7326	0.7266
	0.1	0	1.6286	1.6643	1.4188	1.6680	1.7050	1.7559	1.8836	2.0000	0.2282	0.2508	0.7149	0.5577	0.2130	0.2181	1.2395	0.4566
		-0.05	1.6324	1.6664	1.3431	1.6776	1.7088	1.7589	1.7973	2.0000	0.2238	0.2485	0.7469	0.5442	0.2032	0.2094	1.3719	0.4237
		0.05	1.6586	1.6772	1.5402	1.7247	1.7320	1.7651	1.9620	2.0000	0.1947	0.2344	0.6395	0.4791	0.1890	0.2325	0.9492	0.3603
2000	0.3	0	1.1235	1.1324	1.1426	1.1524	1.1366	1.1445	1.1383	1.1372	0.2877	0.3188	0.3775	0.3997	0.3839	0.4515	0.4820	0.5226
		-0.05	1.1284	1.1353	1.1591	1.1736	1.1308	1.1357	1.1709	1.1650	0.3047	0.3362	0.3987	0.4219	0.4316	0.4964	0.5127	0.5295
		0.05	1.0863	1.1034	1.1079	1.1175	1.0812	1.1142	1.1053	1.1128	0.2847	0.3202	0.3762	0.4011	0.3856	0.4379	0.4862	0.5064
	0.1	0	1.4946	1.5232	1.5189	1.5753	1.5460	1.5911	1.7188	1.6996	0.2473	0.2743	0.5683	0.4777	0.3519	0.3832	0.6458	0.6344
		-0.05	1.5145	1.5420	1.3606	1.5882	1.5579	1.6016	1.6848	1.7286	0.2509	0.2781	0.6987	0.4850	0.3234	0.3491	1.2325	0.5917
		0.05	1.4653	1.4896	1.5391	1.5620	1.5288	1.5754	1.6701	1.6618	0.2819	0.3122	0.5136	0.4876	0.4172	0.4607	0.6730	0.6801

Table S7. Proportions (in %) of extreme values (0 or 2) of $\hat{\gamma}_{PF}$ and $\hat{\gamma}_F$ among 500 replicates for qualitative trait and quantitative trait when $(\sigma_0^2,\ \sigma_1^2,\ \sigma_2^2) = (1,\ 1.2,\ 1)$ with a covariate and $\rho = 0$

			$\hat{\gamma}_{PF}$			$\hat{\gamma}_F$		
Trait	n	MAF	0	2	Total	0	2	Total
Qualitative	500	0.3	15.2	16.0	31.2	15.2	18.6	33.8
		0.1	33.4	4.6	38.0	33.4	11.0	44.4
	2000	0.3	5.2	9.4	14.6	5.2	9.6	14.8
		0.1	16.8	10.4	27.2	16.8	16.0	32.8
Quantitative	500	0.3	8.4	11.2	19.6	8.4	11.4	19.8
		0.1	18.6	12.2	30.8	18.6	18.4	37.0
	2000	0.3	2.6	6.2	8.8	2.6	6.2	8.8
		0.1	4.6	10.4	15.0	4.6	11.8	16.4

Table S8. Mean squared errors (MSEs) of point estimates $\hat{\gamma}_{BN}$, $\hat{\gamma}_{BU}$, $\hat{\gamma}_{PF}$ and $\hat{\gamma}_{F}$ among 500 replicates for qualitative trait and quantitative trait when $(\sigma_0^2,\ \sigma_1^2,\ \sigma_2^2) = (1,\ 1.2,\ 1)$ with a covariate and $\rho = 0$

Trait	n	MAF	$\hat{\gamma}_{BN}$	$\hat{\gamma}_{BU}$	$\hat{\gamma}_{PF}$	$\hat{\gamma}_{F}$
Qualitative	500	0.3	0.2398	0.2920	0.3738	0.4008
		0.1	0.5163	0.5991	0.6940	0.7463
	2000	0.3	0.0993	0.1101	0.1318	0.1351
		0.1	0.2636	0.2840	0.3560	0.4113
Quantitative	500	0.3	0.0970	0.1046	0.1148	0.1168
		0.1	0.2276	0.2572	0.3883	0.4591
	2000	0.3	0.0321	0.0335	0.0348	0.0349
		0.1	0.0937	0.0991	0.1272	0.1520

Table S9. Proportions (in %) of the noninformative intervals (NP), empty sets (EP) and discontinuous intervals (DP) of the 95% confidence intervals for the penalized Fieller's (PF) and Fieller's methods based on 500 replicates for qualitative trait and quantitative trait when $(\sigma_0^2,\ \sigma_1^2,\ \sigma_2^2) = (1,\ 1.2,\ 1)$ with a covariate and $\rho = 0$

			PF			Fieller		
Trait	n	MAF	NP	EP	DP	NP	EP	DP
Qualitative	500	0.3	30.4	0.8	0.0	40.2	2.6	0.6
		0.1	47.8	2.2	0.0	66.2	0.0	0.8
	2000	0.3	2.2	0.6	0.0	3.2	1.0	0.0
		0.1	22.2	3.4	0.0	31.2	0.8	0.4
Quantitative	500	0.3	4.2	0.0	0.0	7.6	0.2	0.2
		0.1	31.6	3.2	0.0	35.0	2.6	0.8
	2000	0.3	0.0	0.0	0.0	0.0	0.0	0.0
		0.1	5.6	0.0	0.0	10.2	0.2	0.0

Table S10. Coverage probabilities (CPs, in %) and the mean and median of the widths of the highest posterior density intervals or confidence intervals (respectively denoted as W_{mean} and W_{median}) of Bayesian method with normal prior (BN), Bayeisan method with uniform prior (BU), penalized Fieller's (PF) and Fieller's methods among 500 replicates for qualitative trait and quantitative trait when $(\sigma_0^2,\ \sigma_1^2,\ \sigma_2^2) = (1,\ 1.2,\ 1)$ with a covariate and $\rho = 0$ [a]

			CP				W_{mean}				W_{median}			
Trait	n	MAF	BN	BU	PF	Fieller	BN	BU	PF	Fieller	BN	BU	PF	Fieller
Qualitative	500	0.3	94.8	95.8	94.2	92.8	1.5325	1.5618	1.5484	1.5657	1.5990	1.6472	1.6573	1.7683
		0.1	89.6	91.2	84.8	97.6	1.6336	1.6845	1.5217	1.8637	1.7250	1.7772	1.9712	2.0000
	2000	0.3	94.4	94.4	93.8	93.0	1.1272	1.1370	1.1463	1.1616	1.1259	1.1269	1.1285	1.1385
		0.1	93.8	94.8	88.8	95.2	1.5065	1.5328	1.4680	1.6200	1.5427	1.5807	1.6835	1.7194
Quantitative	500	0.3	96.4	96.2	97.6	97.2	1.1177	1.1234	1.2226	1.2451	1.1250	1.1301	1.2320	1.2507
		0.1	94.4	96.0	87.8	92.0	1.5020	1.5295	1.4999	1.5558	1.5334	1.5822	1.7555	1.7548
	2000	0.3	94.2	94.8	97.0	97.0	0.6562	0.6589	0.7344	0.7363	0.6676	0.6689	0.7470	0.7495
		0.1	96.6	97.2	97.6	96.4	1.1853	1.1965	1.2969	1.3128	1.2032	1.2279	1.3362	1.3371

[a] The empirical CP should be between 93.05% and 96.95% ($0.95 \pm 2 \times \sqrt{\frac{0.95\times 0.05}{500}}$) with 95% probability.

Table S11. Standard deviation and interquartile range of the widths of the highest posterior density intervals or confidence intervals (respectively denoted as W_{SD} and W_{IQR}) of Bayesian method with normal prior (BN), Bayesian method with uniform prior (BU), penalized Fieller's (PF) and Fieller's methods among 500 replicates for qualitative trait and quantitative trait when $(\sigma_0^2,\ \sigma_1^2,\ \sigma_2^2) = (1,\ 1.2,\ 1)$ with a covariate and $\rho = 0$

			W_{SD}				W_{IQR}			
Trait	n	MAF	BN	BU	PF	Fieller	BN	BU	PF	Fieller
Qualitative	500	0.3	0.2631	0.2896	0.4764	0.5243	0.3589	0.3933	0.7472	0.7382
		0.1	0.2547	0.2576	0.6829	0.3062	0.1682	0.1627	1.0447	0.0971
	2000	0.3	0.2937	0.3259	0.3956	0.4209	0.4170	0.4882	0.5103	0.5371
		0.1	0.2391	0.2678	0.5993	0.4352	0.3073	0.3397	0.7207	0.5674
Quantitative	500	0.3	0.3024	0.3341	0.4239	0.4562	0.4280	0.5049	0.5614	0.6334
		0.1	0.2430	0.2700	0.6009	0.5414	0.3448	0.3834	0.8271	0.6648
	2000	0.3	0.1616	0.1742	0.2029	0.2064	0.1980	0.2200	0.2365	0.2407
		0.1	0.2840	0.3117	0.4284	0.4451	0.3717	0.4335	0.5814	0.6226
