# Supplementary material for: BEXCIS: Bayesian methods for estimating the degree of the skewness of X chromosome inactivation
Source: BMC Bioinformatics. 2022 May 24;23:193. doi: 10.1186/s12859-022-04721-y (PMC9128296; doi:10.1186/s12859-022-04721-y)
Supplement: Supplementary file 4 — Additional file 4. Text--Simulation results of WSD and WIQR, simulation settings with a covariate, and simulation results with a covariate. [file 12859_2022_4721_MOESM4_ESM.pdf]

Additional file 4: Supplementary Text

Simulation results of $\mathbf{W_{SD}}$ and $\mathbf{W_{IQR}}$

The $\mathrm{W_{SD}}$'s and $\mathrm{W_{IQR}}$'s of the BN, BU, PF and Fieller's methods among the 500 replicates for qualitative trait and quantitative trait with $(\sigma_0^2,\ \sigma_1^2,\ \sigma_2^2) = (1,\ 1.2,\ 1)$ are displayed in Additional file 3: Supplementary Table S1. From Additional file 3: Supplementary Table S1, we see that the $\mathrm{W_{SD}}$'s and $\mathrm{W_{IQR}}$'s of the four methods both show different trends when the sample size, the MAF or the type of the trait changes. Specifically, for the qualitative trait, the $\mathrm{W_{SD}}$'s and $\mathrm{W_{IQR}}$'s of the BN, BU and Fieller's methods increase while those of the PF method decrease as MAF gets bigger with $n = 500$ or n becomes larger with MAF $= 0.1$. For the quantitative trait, the $\mathrm{W_{SD}}$'s and $\mathrm{W_{IQR}}$'s of the BN and BU methods still turn to be larger while those of the PF and Fieller's methods become smaller for higher MAF with $n = 500$ or greater n with MAF $= 0.1$. Fixing MAF $= 0.1$, the $\mathrm{W_{SD}}$'s of the BN, BU and Fieller's methods increase while those of the PF method decline when the trait turns from qualitative into quantitative. When $n = 500$ and MAF $= 0.1$, if the trait turns from qualitative into quantitative, the $\mathrm{W_{IQR}}$'s of these four methods all increase. When $n = 2{,}000$ and MAF $= 0.1$, the $\mathrm{W_{IQR}}$'s of these four methods for the qualitative trait are similar to those for the quantitative trait. Except for the situations we discussed above, the $\mathrm{W_{SD}}$'s and $\mathrm{W_{IQR}}$'s of these four interval estimation methods generally become less with larger n, bigger MAF or the trait changing from qualitative to quantitative. The reason for these results can be listed as follows. The BN, BU and Fieller's methods generally obtain wide intervals while the PF method has more chance to get shorter CIs with many noninformative CIs when $n = 500$ and MAF $= 0.1$, especially for the qualitative trait (Additional file 2: Supplementary Figures S25-S27 and S36-S38). So, the BN, BU and Fieller's methods have smaller $\mathrm{W_{SD}}$'s and $\mathrm{W_{IQR}}$'s compared to the PF method when $n = 500$ and MAF $= 0.1$. The four methods all have more chance to get shorter intervals as n becomes larger, MAF gets higher or the trait turns from qualitative into quantitative, which tend to enlarge the variation of the BN, BU

and Fieller's methods but reduce that of the PF method. For the situations with larger n, higher MAF or the trait turns from qualitative into quantitative, all the four methods are likely to obtain shorter intervals, and increasing n, MAF or changing the trait from qualitative to quantitative may lead to smaller $\mathrm{W_{SD}}$'s and $\mathrm{W_{IQR}}$'s of the four methods.

Simulation settings with a covariate

Assume that the frequencies of the normal allele d and the deleterious allele D are q and p ($p+q=1$), respectively. Since the simulation results without any covariate in the Results section all indicate that ρ has little impact on the performances of the proposed methods, we only consider the situations where the inbreeding coefficient $\rho=0$ for these additional simulations with a covariate. As such, we have the frequencies of genotypes dd, Dd and DD are $(g_0,\ g_1,\ g_2)=(q^2,\ 2pq,\ p^2)$. Then, we can simulate the samples of genotypes dd, Dd and DD from a trinomial distribution with probabilities $(g_0,\ g_1,\ g_2)$. MAF (i.e., p) is set to 0.3 and 0.1, and the sample size n is fixed at 500 and 2,000. For the qualitative trait, Y_i is generated by $Y_i \sim Bernoulli(p_i)$ with $p_i=\frac{1}{1+\exp[-(\beta_0+\beta\gamma X_{1i}+\beta(2-\gamma)X_{2i}+bZ_i)]}$, where Z_i is the covariate of female i which is simulated from the standard normal distribution and b is the corresponding regression coefficient. To fix the case-control ratio at $1:1$, we keep sampling from the trinomial distribution until the number of the cases and that of the controls are both greater than $n/2$. Then, we randomly extract $n/2$ samples from the case group and the control group to create the final study sample of size n, respectively. We can accordingly get X_{1i} and X_{2i} for all the females. On the other hand, for the quantitative trait, we directly simulate the numbers n_0, n_1 and n_2 with $n_0+n_1+n_2=n$ for genotypes dd, Dd and DD from the trinomial distribution with probabilities $(g_0,\ g_1,\ g_2)$ and Y_i is generated by $Y_i \sim N\left(\mu_i, \sigma_0^2 I_{\{G_i=dd\}}+\sigma_1^2 I_{\{G_i=Dd\}}+\sigma_2^2 I_{\{G_i=DD\}}\right)$ with $\mu_i=\beta_0+\beta\gamma X_{1i}+\beta(2-\gamma)X_{2i}+bZ_i$. For both the qualitative trait and the quantitative trait, β_0, β and b are set to be 0, 0.3 and 0.5, respectively, and γ is randomly sampled from $U(0,\ 2)$. For the quantitative trait, $(\sigma_0^2,\ \sigma_1^2,\ \sigma_2^2)=(1,\ 1.2,\ 1)$. For each simulation setting, we conduct 500 replicates (i.e., 500 SNPs) and the confidence level $(1-\alpha)$ is fixed at 95% for the frequentist methods.

To make the HPDIs comparable to the CIs, we calculate the 95% HPDIs for the Bayesian methods. The prior distributions of γ, β_0, β, b and σ_j (j=0, 1, 2) in the Bayesian methods are selected as follows: $\gamma \sim U(0,\ 2)$ and $\gamma \sim N(1,\ 1) \in [0,\ 2]$, $\beta_0 \sim N(0,\ 10^2)$, $\beta \sim N(0,\ 10^2)$, $b \sim N(0,\ 10^2)$ and $\sigma_j \sim \exp(1)$. We set 8 chains to extract

the samples parallelly and simultaneously. We extract 20,000 samples in each chain, among which the first 10,000 samples are only used for warming up and are discarded when the sampling is finished. So eventually, we get 80,000 samples in total. The target acceptance rate is set to be 0.99 to ensure the convergence. The convergence diagnostic $\hat{R}$ for Markov chains in the Bayesian method is done, and the calculated $\hat{R}$'s in our Bayesian models are all less than 1.05 which indicates good convergence (data not shown). The simulation study is implemented by the R software (version 4.0.0).

Simulation results with a covariate

The proportions of the extreme values of $\hat{\gamma}_{PF}$ and $\hat{\gamma}_F$ among the 500 replicates for the qualitative trait and the quantitative trait when $(\sigma_0^2,\ \sigma_1^2,\ \sigma_2^2) = (1,\ 1.2,\ 1)$ with a covariate and $\rho = 0$ are presented in Additional file 3: Supplementary Table S7, the MSEs of $\hat{\gamma}_{BN}$, $\hat{\gamma}_{BU}$, $\hat{\gamma}_{PF}$ and $\hat{\gamma}_F$ for the qualitative trait and the quantitative trait when $(\sigma_0^2,\ \sigma_1^2,\ \sigma_2^2) = (1,\ 1.2,\ 1)$ with a covariate and $\rho = 0$ are shown in Additional file 3: Supplementary Table S8, and the scatter plots of these four point estimates against the true values of γ under these settings are respectively displayed in Additional file 5: Supplementary Figures S69-S76. The NPs, EPs and DPs of the PF and Fieller's methods among the 500 replicates are displayed in Additional file 3: Supplementary Table S9, the CPs, $\mathrm{W_{mean}}$'s and $\mathrm{W_{median}}$'s of the BN, BU, PF and Fieller's methods are listed in Additional file 3: Supplementary Table S10, the $\mathrm{W_{SD}}$'s and $\mathrm{W_{IQR}}$'s of the BN, BU, PF and Fieller's methods among the 500 replicates for the qualitative trait and the quantitative trait with $(\sigma_0^2,\ \sigma_1^2,\ \sigma_2^2) = (1,\ 1.2,\ 1)$ are displayed in Additional file 3: Supplementary Table S11, and the widths of the 95% HPDIs or CIs for these four interval estimation methods against the true values of γ under these settings are respectively presented in Additional file 5: Supplementary Figures S77-S84. From these results, we see that although the performances of all the proposed methods under the scenarios with a covariate are worse than those without any covariate, e.g., all the proposed methods may have larger MSEs and wider intervals, the PF and Fieller's methods may have greater proportions of the extreme point estimates and higher NPs and EPs, and the Fieller's method may have bigger DPs, the trends are similar to those in the Results section without any covariate. More importantly, the Bayesian methods also show their own advantages over the PF and Fieller's methods in both the point estimation and the interval estimation.
