# Supplementary material for: BEXCIS: Bayesian methods for estimating the degree of the skewness of X chromosome inactivation
Source: BMC Bioinformatics. 2022 May 24;23:193. doi: 10.1186/s12859-022-04721-y (PMC9128296; doi:10.1186/s12859-022-04721-y)
Supplement: Supplementary file 5 — Additional file 5. Figs. S45-S56. Scatter plots of point estimates of γ against true value of γ for quantitative trait when (σ02, σ12, σ22)=(4, 4.8, 4) with n=500 and 2000, MAF=0.3 and 0.1, and ρ=0, -0.05 and 0.05, respectively. Figs. S57-S68. Widths of HPDIs or CIs against true value of γ for quantitative trait when (σ02, σ12, σ22)=(4, 4.8, 4) with n=500 and 2000, MAF=0.3 and 0.1, and ρ=0, -0.05 and 0.05, respectively. Figs. S69-S76. Scatter plots of point estimates of γ against true value of γ for qualitative trait and quantitative trait when (σ02, σ12, σ22)=(1, 1.2, 1) with a covariate when n=500 and 2000, MAF=0.3 and 0.1, and ρ=0, respectively. Figs. S77-S84. Widths of HPDIs or CIs against true value of γ for qualitative trait and quantitative trait when (σ02, σ12, σ22)=(1, 1.2, 1) with a covariate when n=500 and 2000, MAF=0.3 and 0.1, and ρ=0, respectively. [file 12859_2022_4721_MOESM5_ESM.pdf]

Additional file 5: Supplementary Figures S45–S84

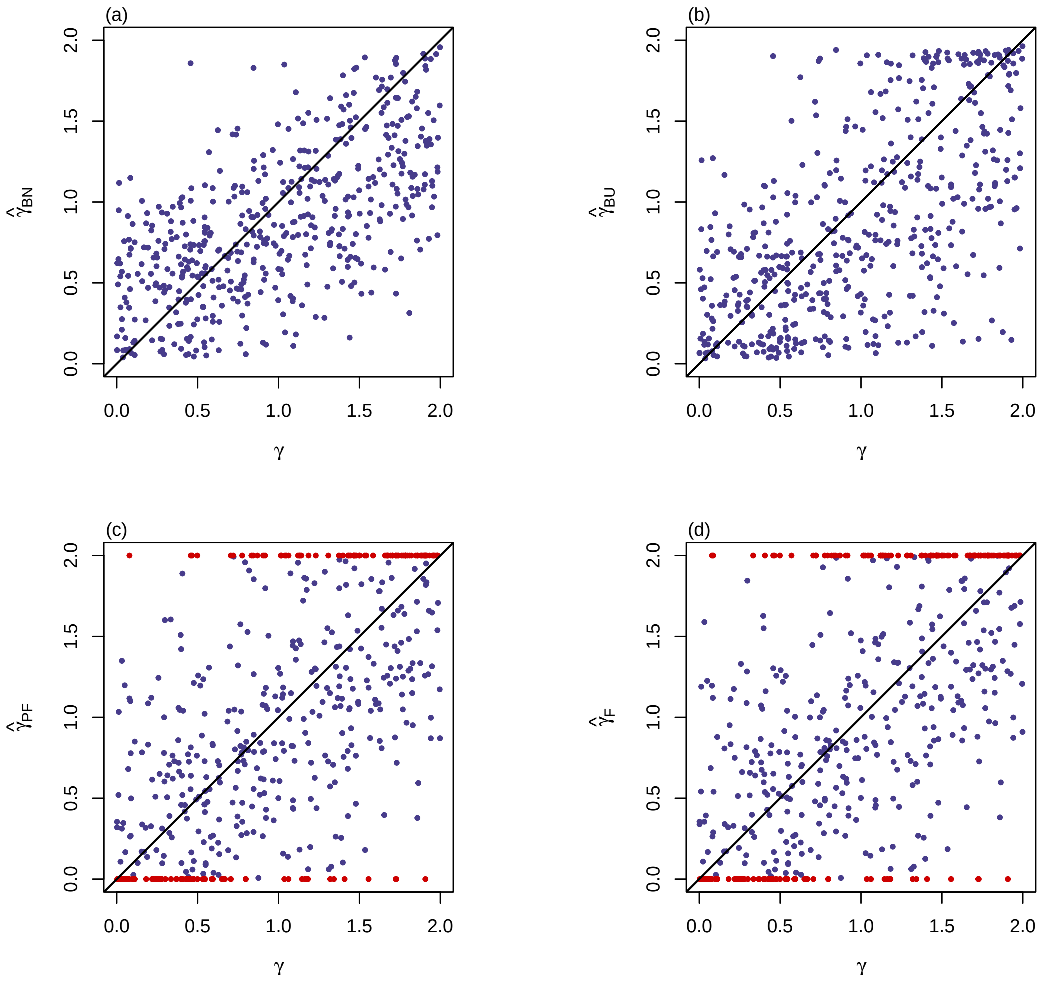

Supplementary Figure S45 Scatter plots of point estimates of γ against true value of γ for quantitative trait when $(\sigma_0^2,\ \sigma_1^2,\ \sigma_2^2) = (4,\ 4.8,\ 4)$ with $n = 500$, MAF $= 0.3$ and $\rho = 0$. The red points represent the extreme values (0 or 2). **(a)** $\hat{\gamma}_{BN}$; **(b)** $\hat{\gamma}_{BU}$; **(c)** $\hat{\gamma}_{PF}$; **(d)** $\hat{\gamma}_F$

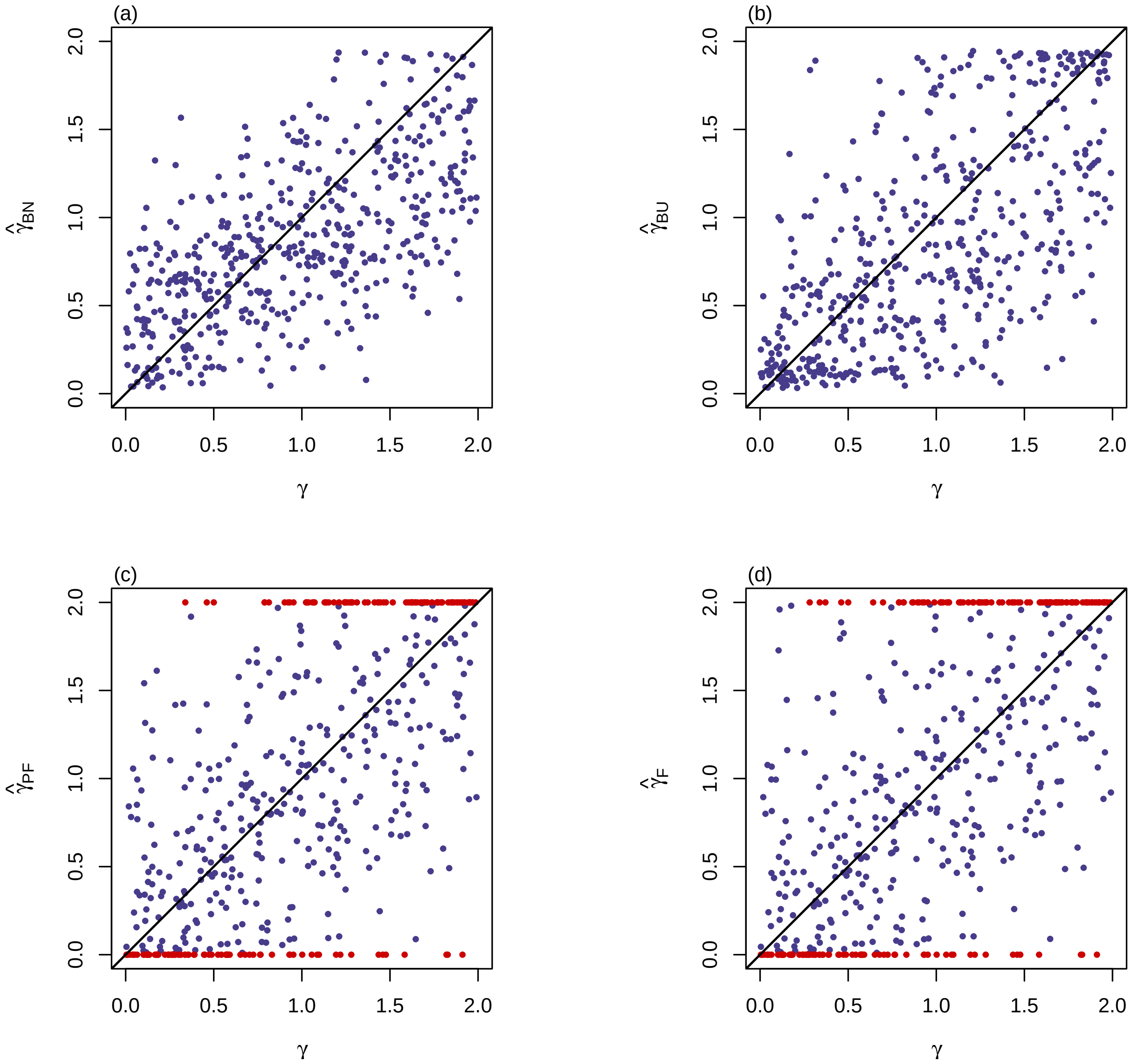

Supplementary Figure S46 Scatter plots of point estimates of γ against true value of γ for quantitative trait when $(\sigma_0^2,\ \sigma_1^2,\ \sigma_2^2) = (4,\ 4.8,\ 4)$ with $n = 500$, MAF $= 0.3$ and $\rho = -0.05$. The red points represent the extreme values (0 or 2). **(a)** $\hat{\gamma}_{BN}$; **(b)** $\hat{\gamma}_{BU}$; **(c)** $\hat{\gamma}_{PF}$; **(d)** $\hat{\gamma}_F$

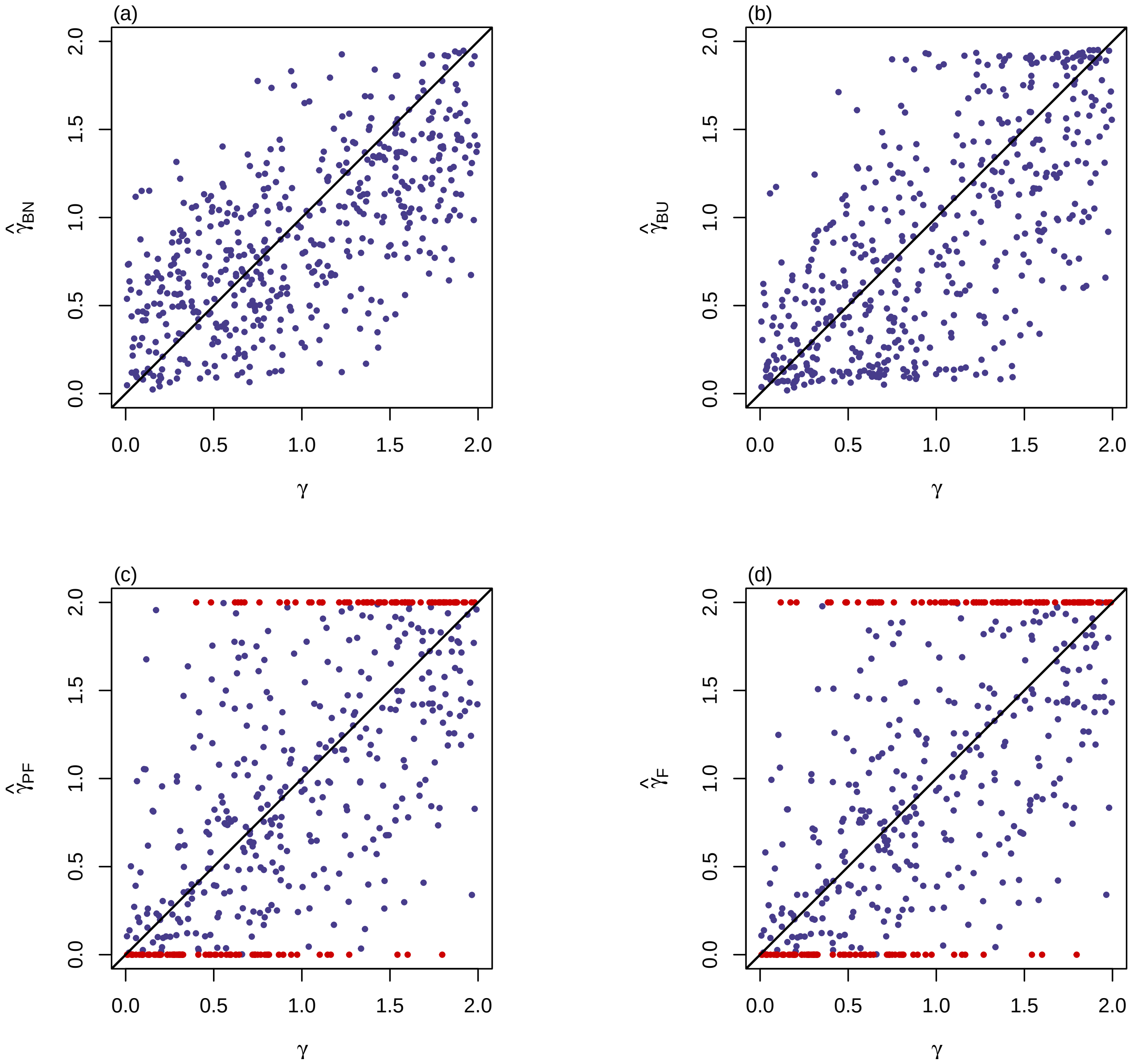

Supplementary Figure S47 Scatter plots of point estimates of γ against true value of γ for quantitative trait when $(\sigma_0^2,\ \sigma_1^2,\ \sigma_2^2) = (4,\ 4.8,\ 4)$ with $n = 500$, MAF $= 0.3$ and $\rho = 0.05$. The red points represent the extreme values (0 or 2). **(a)** $\hat{\gamma}_{BN}$; **(b)** $\hat{\gamma}_{BU}$; **(c)** $\hat{\gamma}_{PF}$; **(d)** $\hat{\gamma}_{F}$

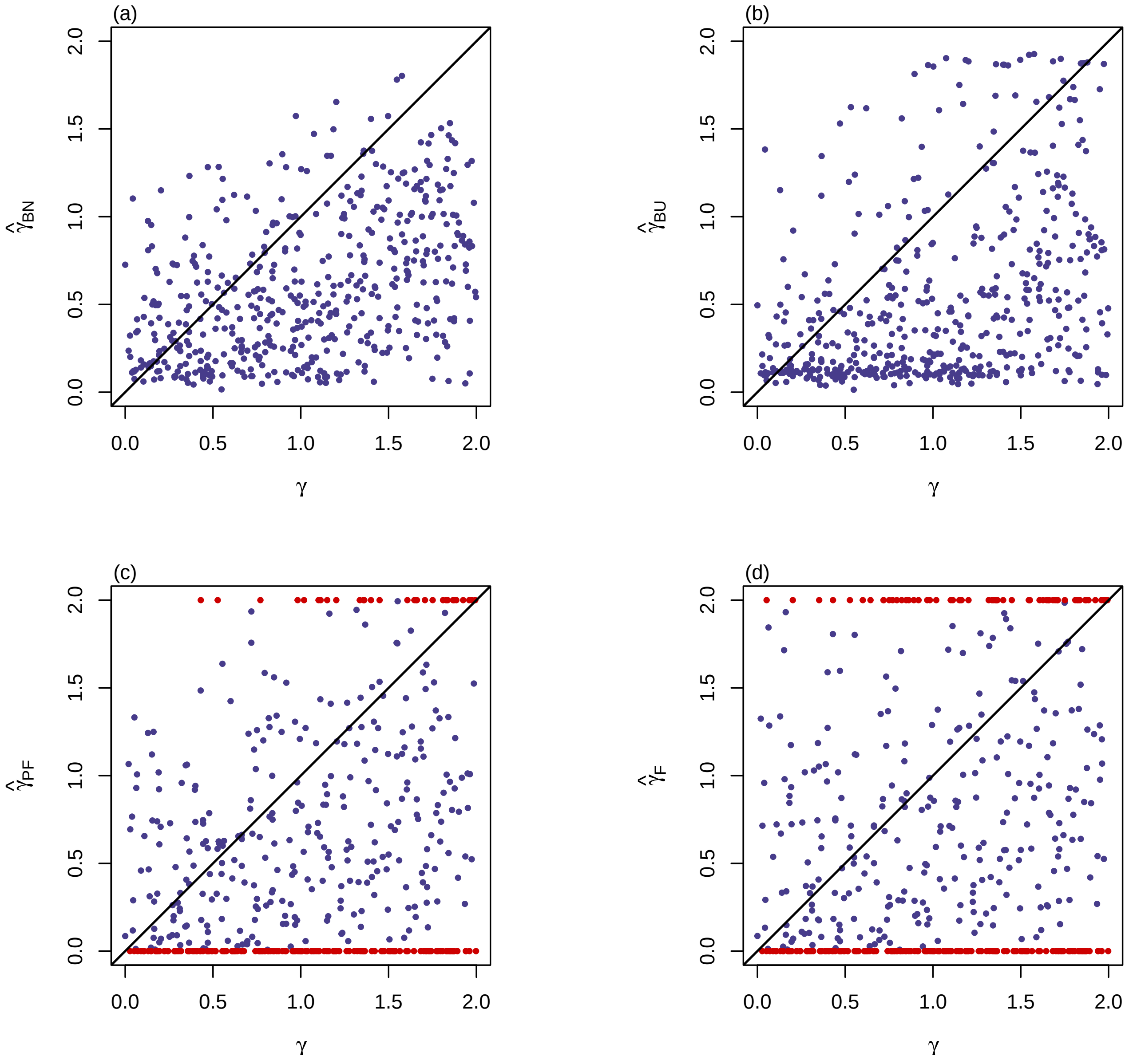

Supplementary Figure S48 Scatter plots of point estimates of γ against true value of γ for quantitative trait when $(\sigma_0^2,\ \sigma_1^2,\ \sigma_2^2) = (4,\ 4.8,\ 4)$ with $n = 500$, MAF $= 0.1$ and $\rho = 0$. The red points represent the extreme values (0 or 2). **(a)** $\hat{\gamma}_{BN}$; **(b)** $\hat{\gamma}_{BU}$; **(c)** $\hat{\gamma}_{PF}$; **(d)** $\hat{\gamma}_F$

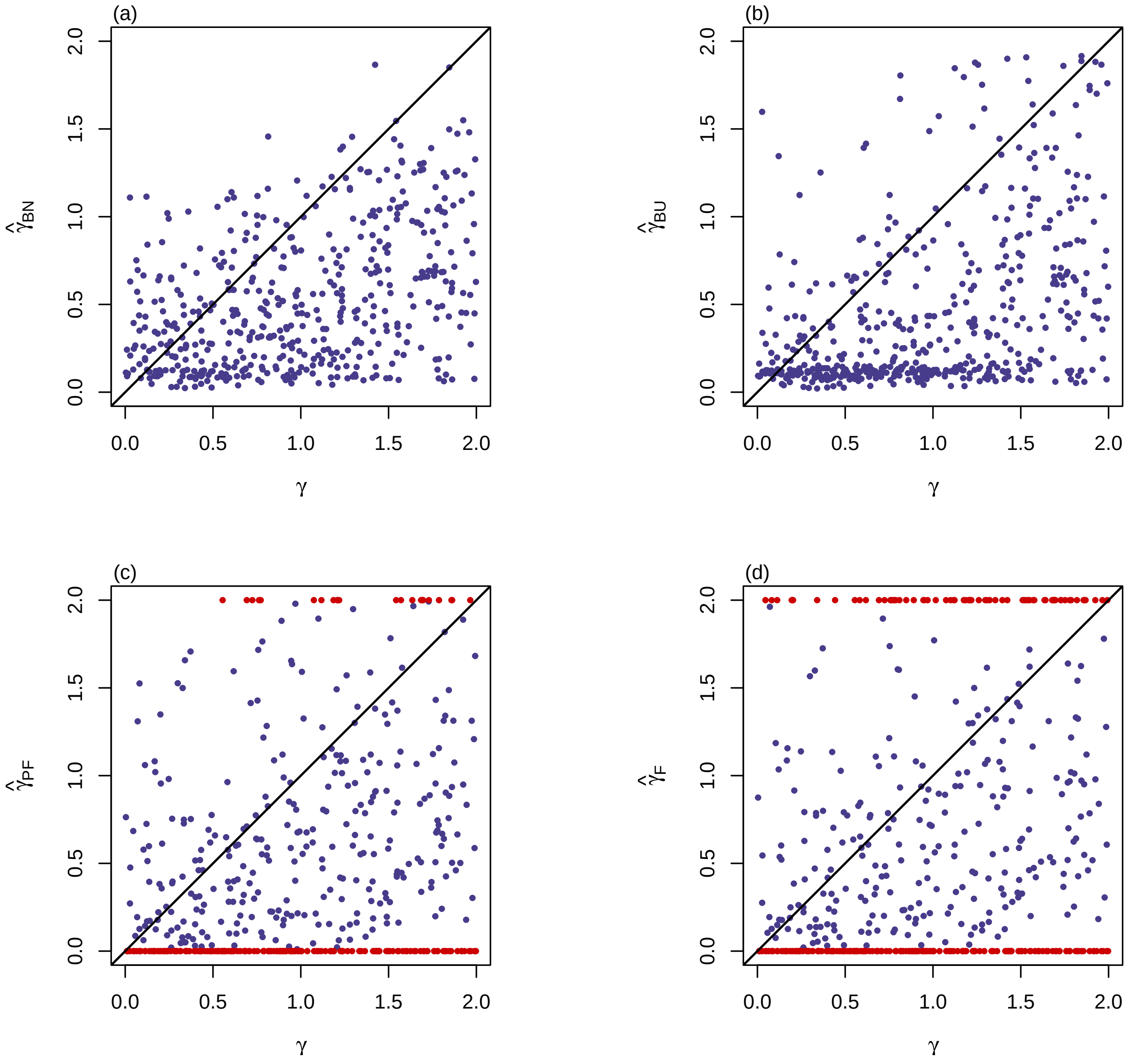

Supplementary Figure S49 Scatter plots of point estimates of γ against true value of γ for quantitative trait when $(\sigma_0^2,\ \sigma_1^2,\ \sigma_2^2) = (4,\ 4.8,\ 4)$ with $n = 500$, MAF $= 0.1$ and $\rho = -0.05$. The red points represent the extreme values (0 or 2). **(a)** $\hat{\gamma}_{BN}$; **(b)** $\hat{\gamma}_{BU}$; **(c)** $\hat{\gamma}_{PF}$; **(d)** $\hat{\gamma}_F$

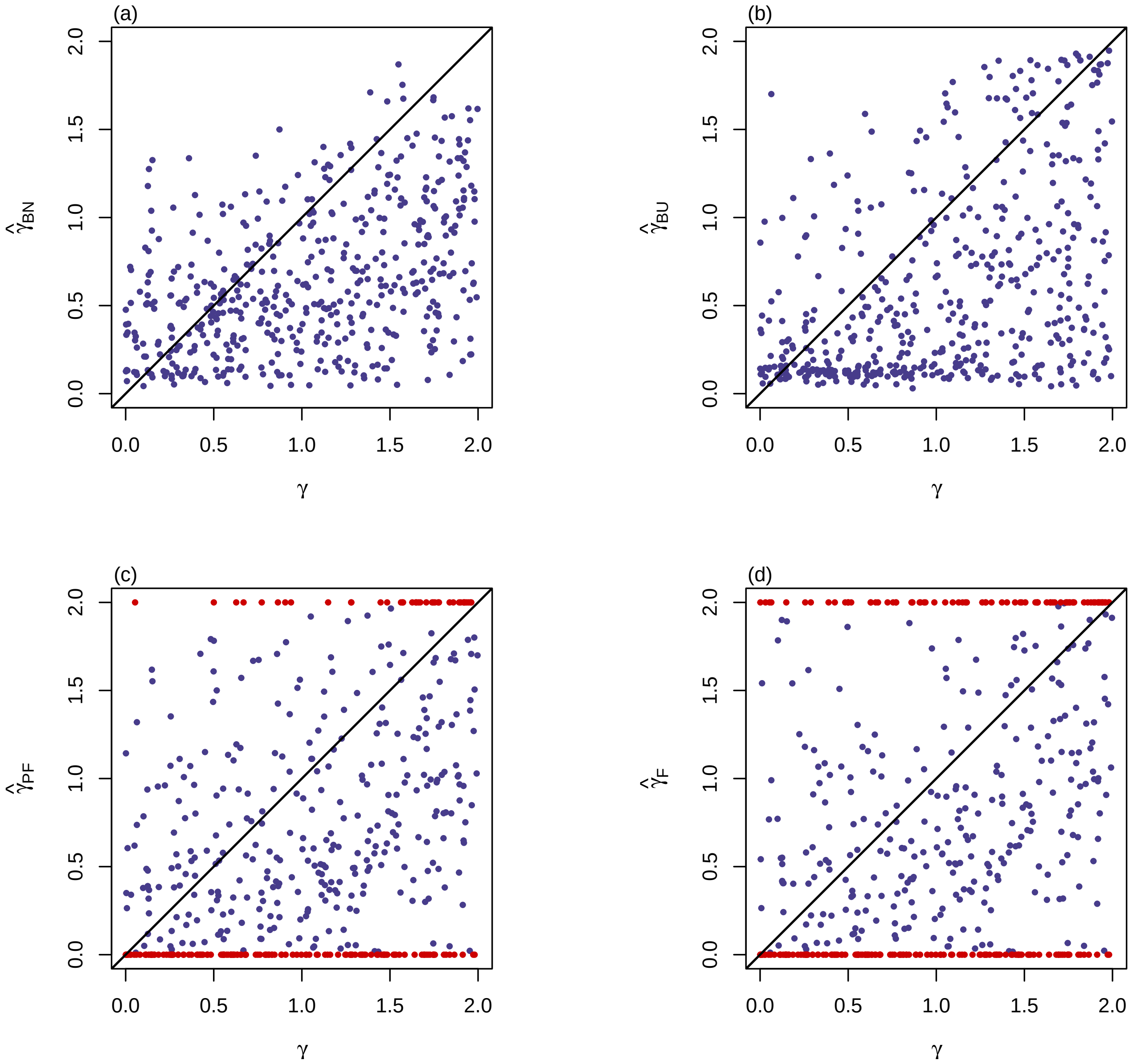

Supplementary Figure S50 Scatter plots of point estimates of γ against true value of γ for quantitative trait when $(\sigma_0^2,\ \sigma_1^2,\ \sigma_2^2) = (4,\ 4.8,\ 4)$ with $n = 500$, MAF $= 0.1$ and $\rho = 0.05$. The red points represent the extreme values (0 or 2). **(a)** $\hat{\gamma}_{BN}$; **(b)** $\hat{\gamma}_{BU}$; **(c)** $\hat{\gamma}_{PF}$; **(d)** $\hat{\gamma}_F$

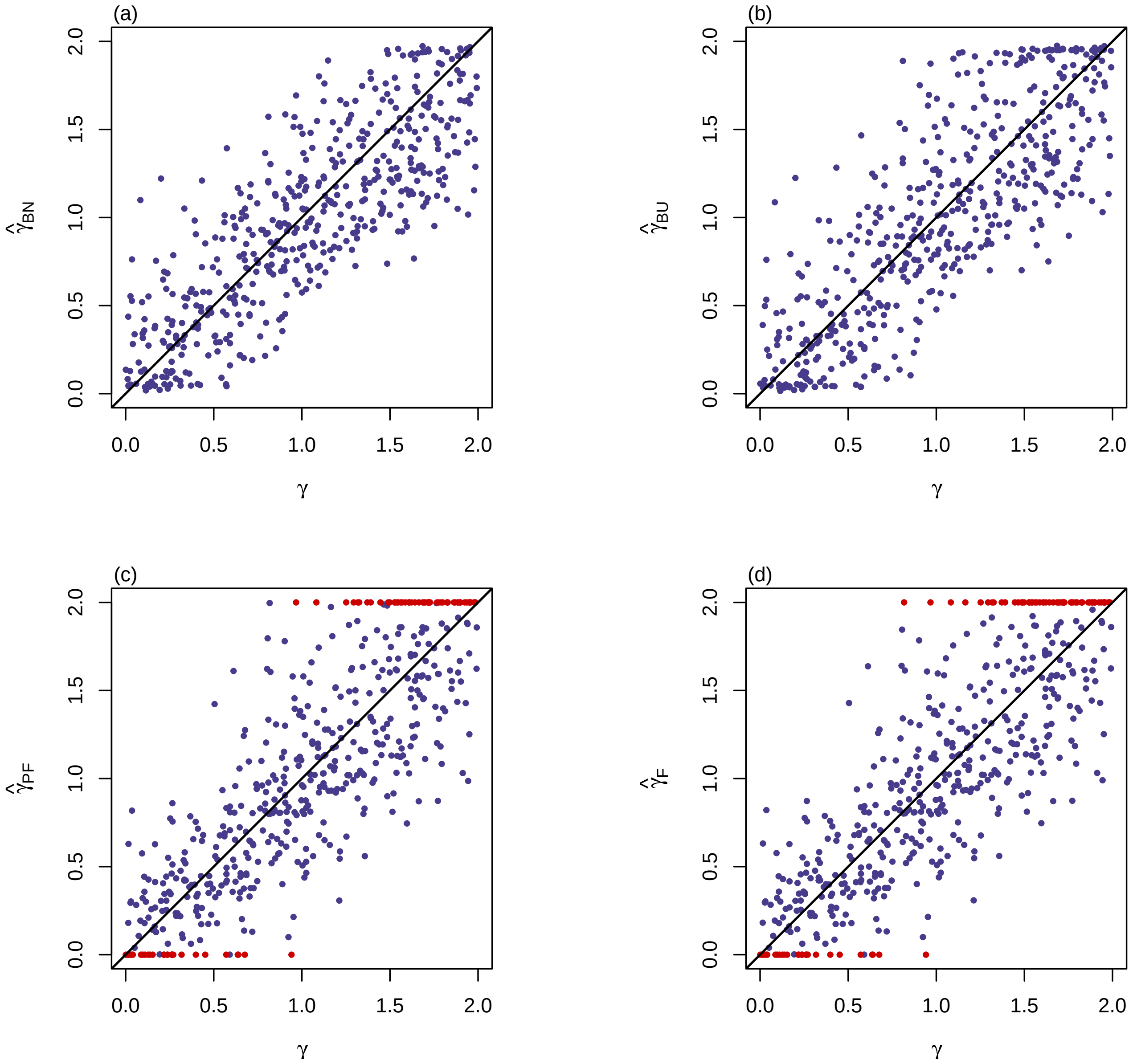

Supplementary Figure S51 Scatter plots of point estimates of γ against true value of γ for quantitative trait when $(\sigma_0^2,\ \sigma_1^2,\ \sigma_2^2) = (4,\ 4.8,\ 4)$ with $n = 2000$, MAF $= 0.3$ and $\rho = 0$. The red points represent the extreme values (0 or 2). **(a)** $\hat{\gamma}_{BN}$; **(b)** $\hat{\gamma}_{BU}$; **(c)** $\hat{\gamma}_{PF}$; **(d)** $\hat{\gamma}_F$

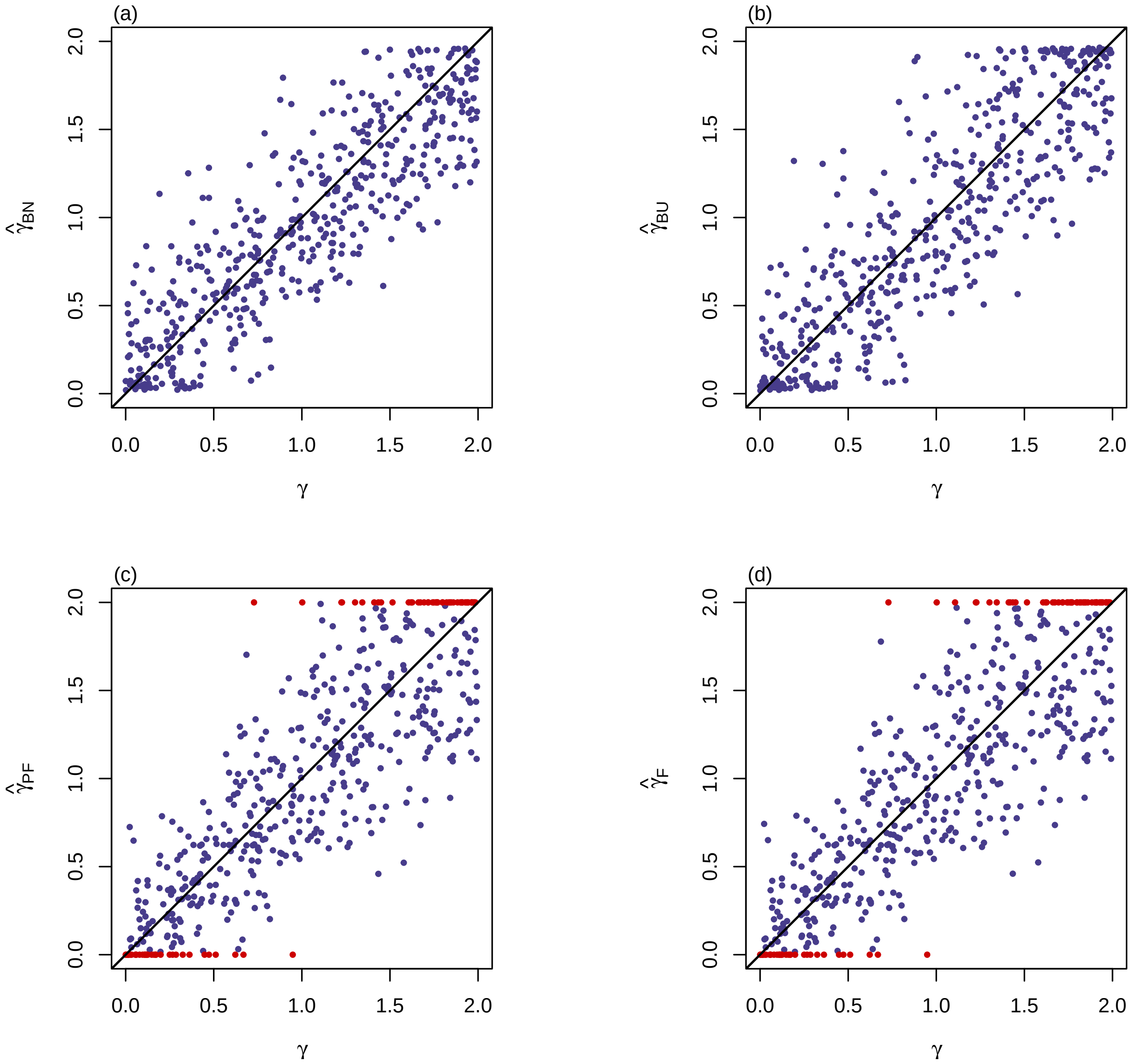

Supplementary Figure S52 Scatter plots of point estimates of γ against true value of γ for quantitative trait when $(\sigma_0^2,\ \sigma_1^2,\ \sigma_2^2) = (4,\ 4.8,\ 4)$ with $n = 2000$, MAF $= 0.3$ and $\rho = -0.05$. The red points represent the extreme values (0 or 2). **(a)** $\hat{\gamma}_{BN}$; **(b)** $\hat{\gamma}_{BU}$; **(c)** $\hat{\gamma}_{PF}$; **(d)** $\hat{\gamma}_{F}$

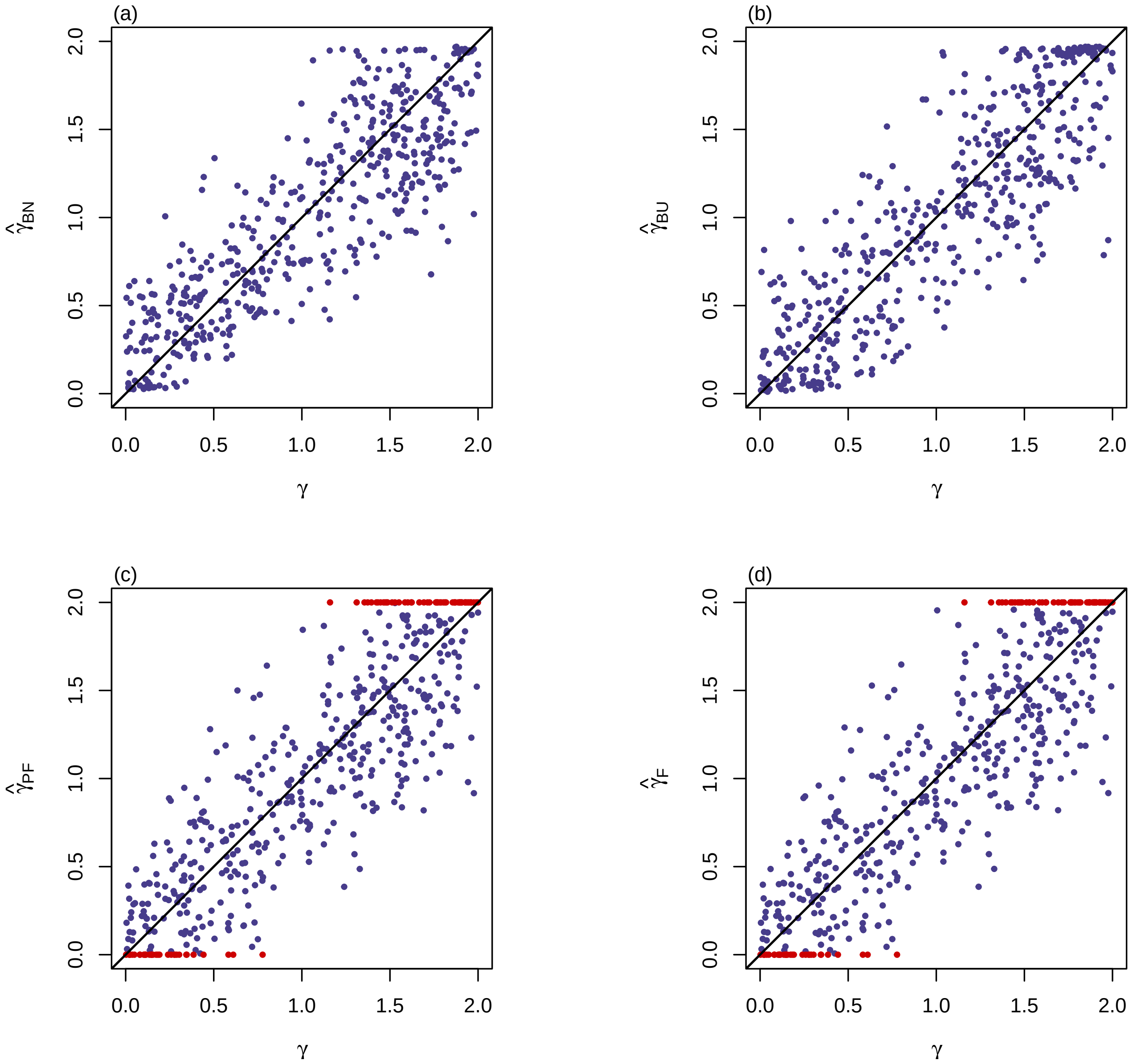

Supplementary Figure S53 Scatter plots of point estimates of γ against true value of γ for quantitative trait when $(\sigma_0^2,\ \sigma_1^2,\ \sigma_2^2) = (4,\ 4.8,\ 4)$ with $n = 2000$, MAF $= 0.3$ and $\rho = 0.05$. The red points represent the extreme values (0 or 2). **(a)** $\hat{\gamma}_{BN}$; **(b)** $\hat{\gamma}_{BU}$; **(c)** $\hat{\gamma}_{PF}$; **(d)** $\hat{\gamma}_F$

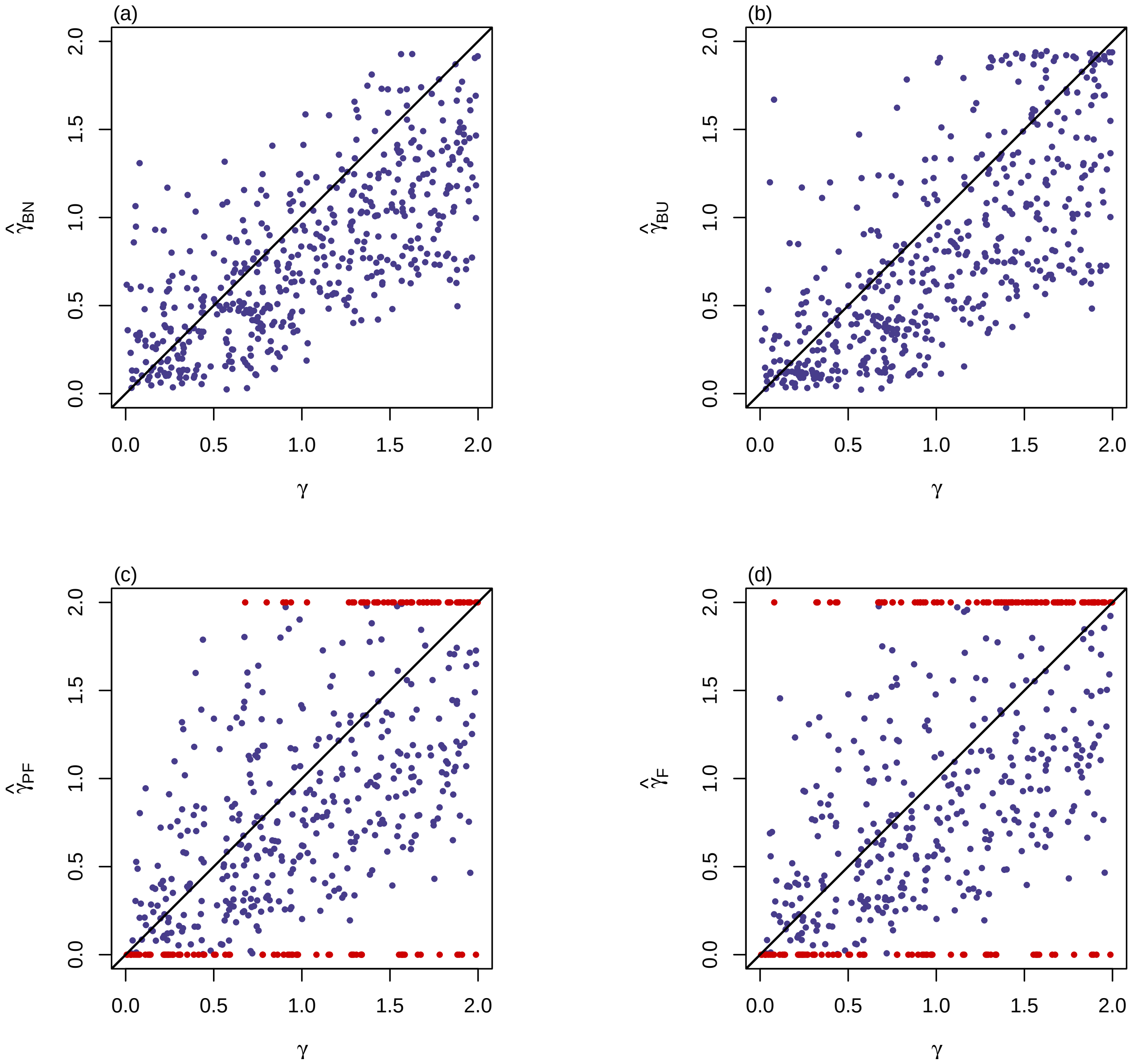

Supplementary Figure S54 Scatter plots of point estimates of γ against true value of γ for quantitative trait when $(\sigma_0^2,\ \sigma_1^2,\ \sigma_2^2) = (4,\ 4.8,\ 4)$ with $n = 2000$, MAF $= 0.1$ and $\rho = 0$. The red points represent the extreme values (0 or 2). **(a)** $\hat{\gamma}_{BN}$; **(b)** $\hat{\gamma}_{BU}$; **(c)** $\hat{\gamma}_{PF}$; **(d)** $\hat{\gamma}_F$

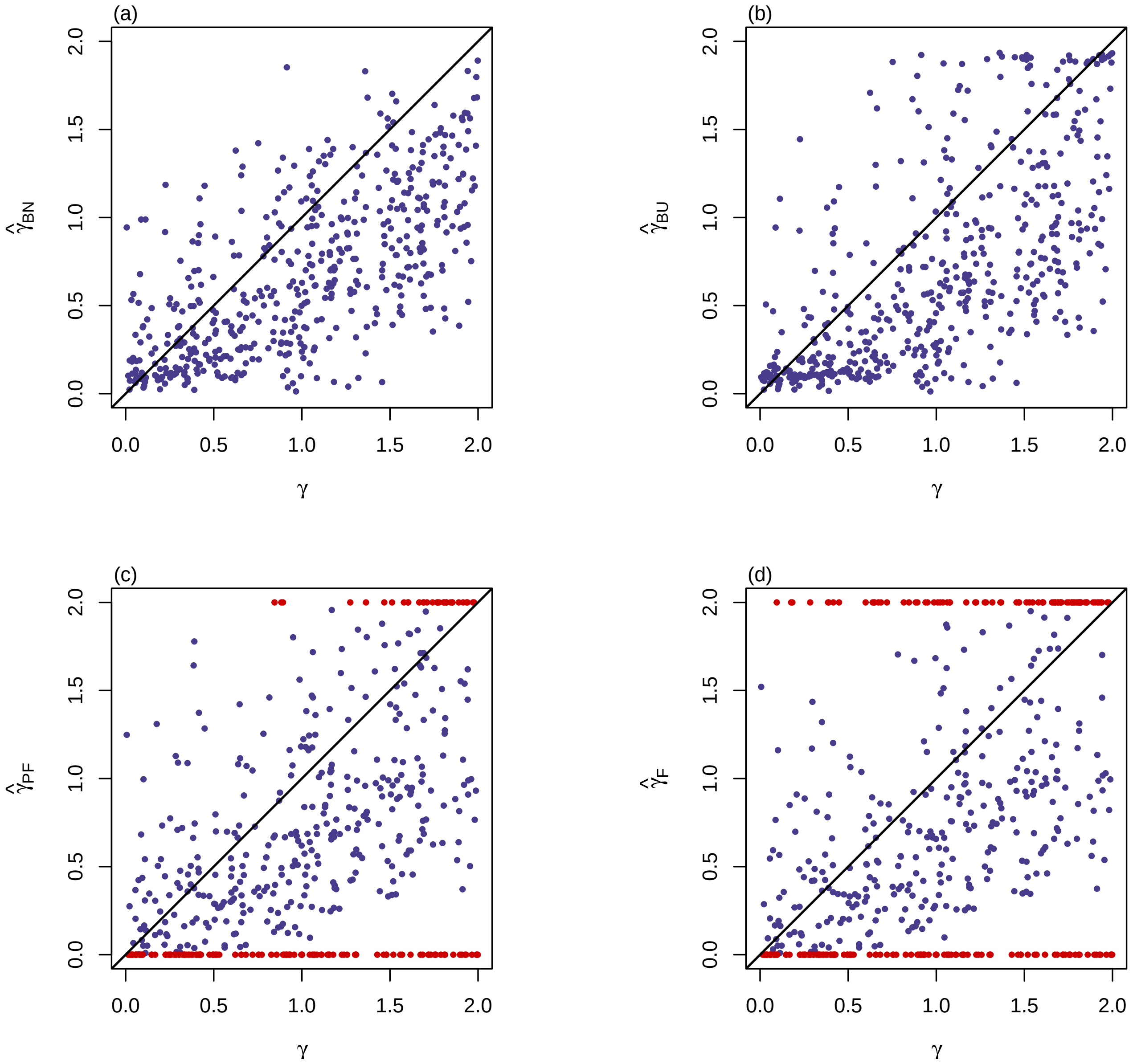

Supplementary Figure S55 Scatter plots of point estimates of γ against true value of γ for quantitative trait when $(\sigma_0^2,\ \sigma_1^2,\ \sigma_2^2) = (4,\ 4.8,\ 4)$ with $n = 2000$, MAF $= 0.1$ and $\rho = -0.05$. The red points represent the extreme values (0 or 2). **(a)** $\hat{\gamma}_{BN}$; **(b)** $\hat{\gamma}_{BU}$; **(c)** $\hat{\gamma}_{PF}$; **(d)** $\hat{\gamma}_F$

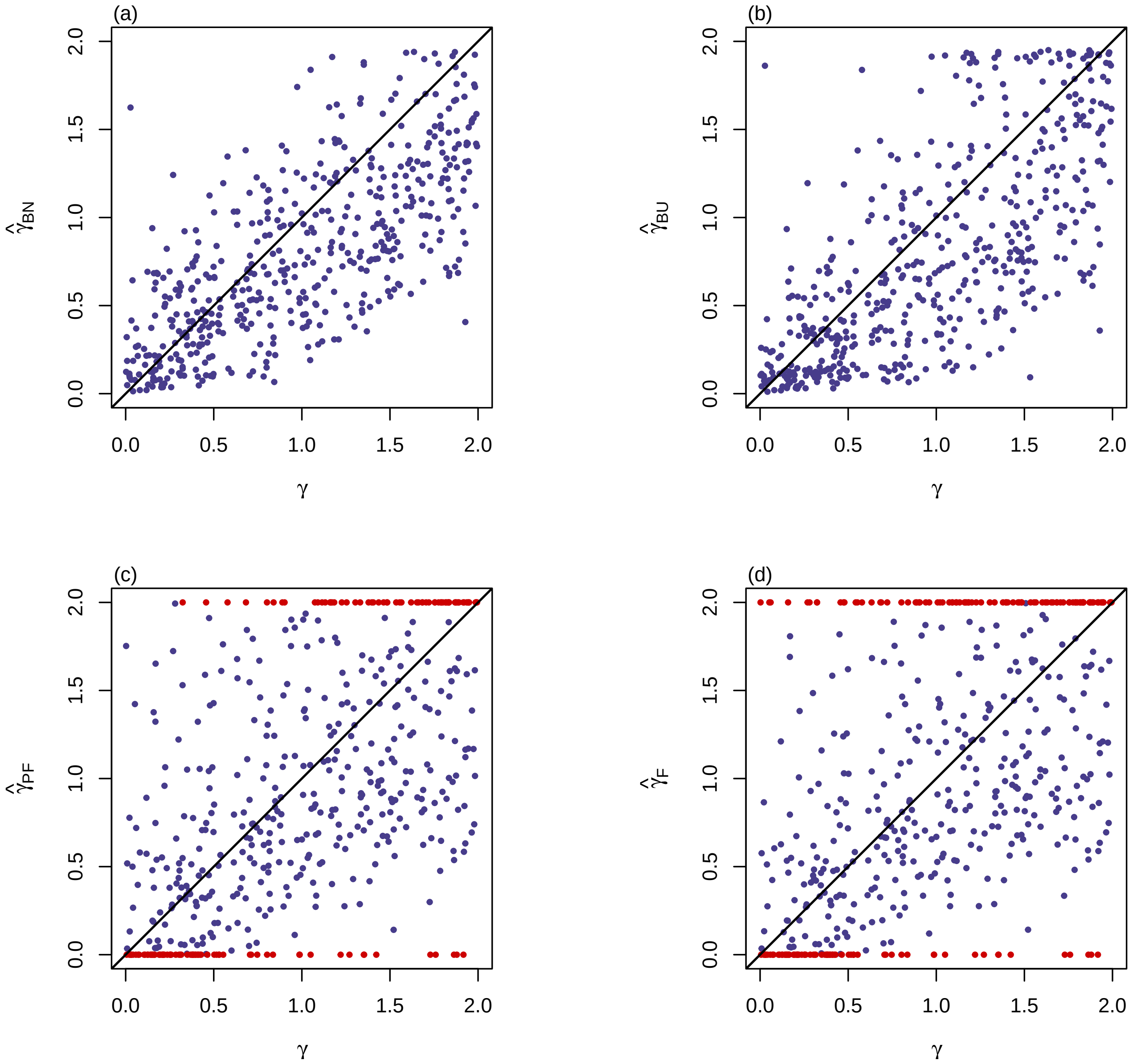

Supplementary Figure S56 Scatter plots of point estimates of γ against true value of γ for quantitative trait when $(\sigma_0^2,\ \sigma_1^2,\ \sigma_2^2) = (4,\ 4.8,\ 4)$ with $n = 2000$, MAF $= 0.1$ and $\rho = 0.05$. The red points represent the extreme values (0 or 2). **(a)** $\hat{\gamma}_{BN}$; **(b)** $\hat{\gamma}_{BU}$; **(c)** $\hat{\gamma}_{PF}$; **(d)** $\hat{\gamma}_F$

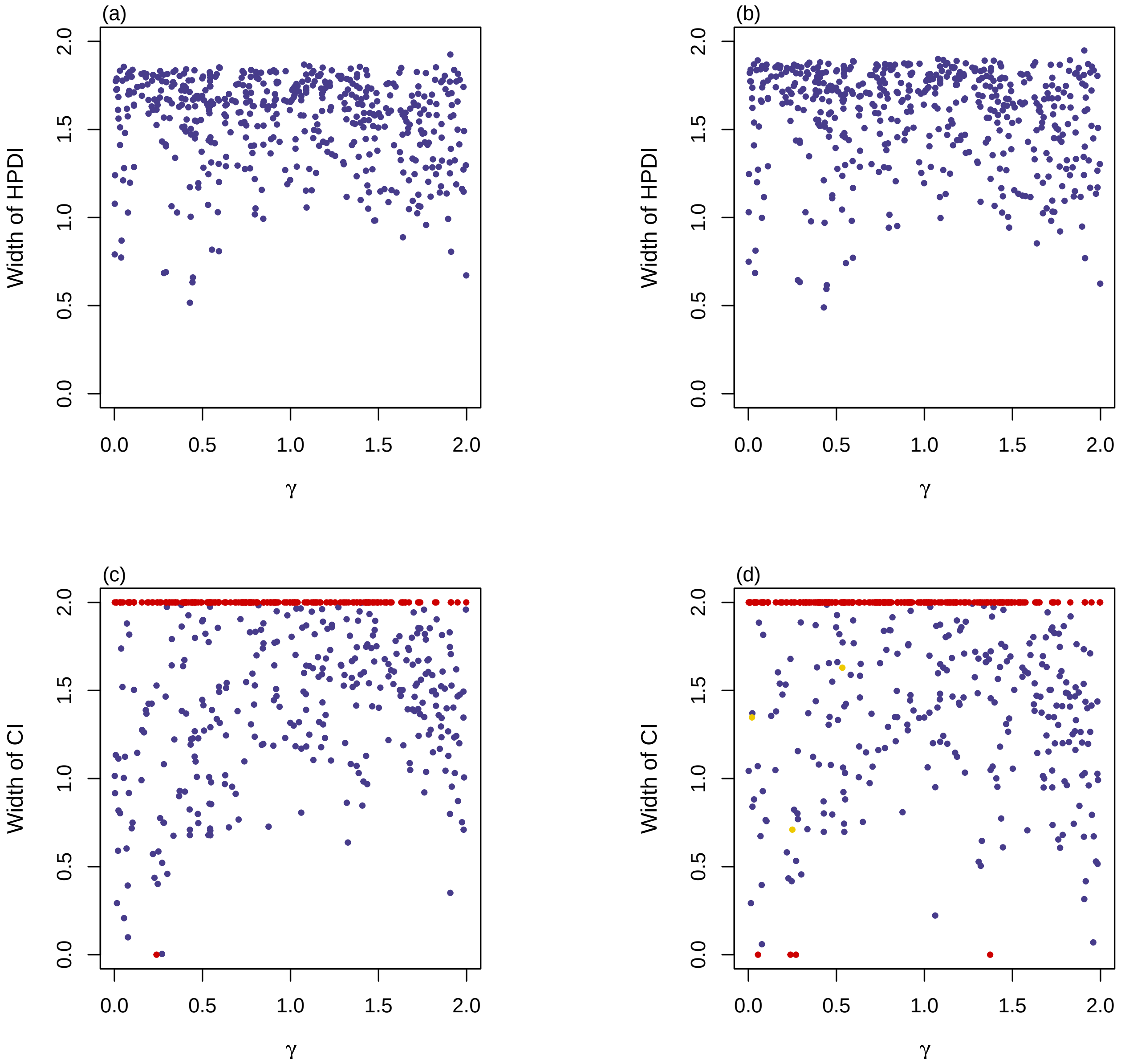

Supplementary Figure S57 Widths of HPDIs or CIs against true value of γ for quantitative trait when $(\sigma_0^2,\ \sigma_1^2,\ \sigma_2^2) = (4,\ 4.8,\ 4)$ with $n = 500$, MAF $= 0.3$ and $\rho = 0$. The red points represent the widths of the noninformative intervals or the empty sets, and the yellow points represent the widths of the discontinuous intervals. **(a)** BN method; **(b)** BU method; **(c)** PF method; **(d)** Fieller's method

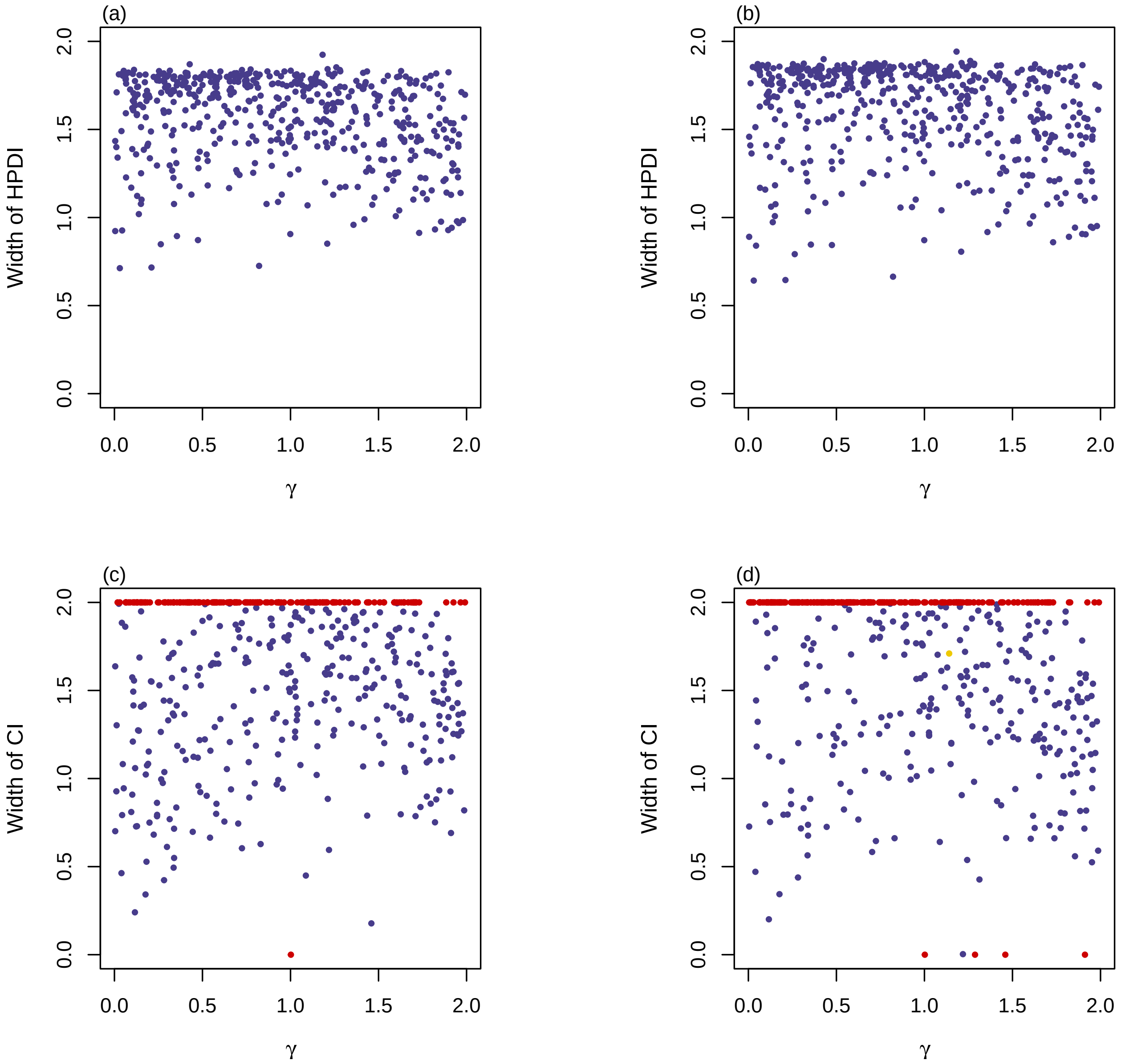

Supplementary Figure S58 Widths of HPDIs or CIs against true value of γ for quantitative trait when $(\sigma_0^2,\ \sigma_1^2,\ \sigma_2^2) = (4,\ 4.8,\ 4)$ with $n = 500$, MAF $= 0.3$ and $\rho = -0.05$. The red points represent the widths of the noninformative intervals or the empty sets, and the yellow point represents the width of the discontinuous interval. **(a)** BN method; **(b)** BU method; **(c)** PF method; **(d)** Fieller's method

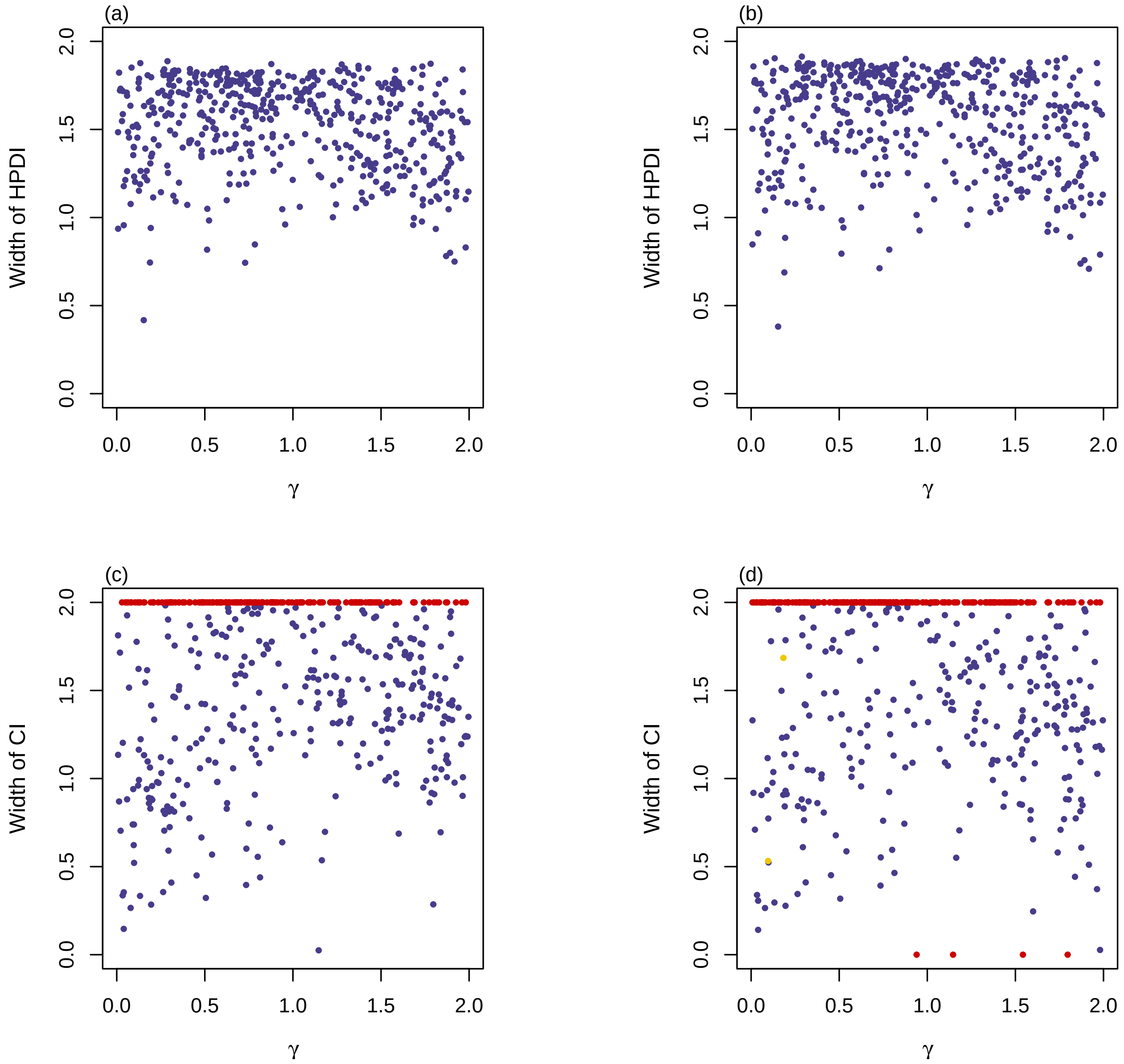

Supplementary Figure S59 Widths of HPDIs or CIs against true value of γ for quantitative trait when $(\sigma_0^2,\ \sigma_1^2,\ \sigma_2^2) = (4,\ 4.8,\ 4)$ with $n = 500$, MAF $= 0.3$ and $\rho = 0.05$. The red points represent the widths of the noninformative intervals or the empty sets, and the yellow points represent the widths of the discontinuous intervals. **(a)** BN method; **(b)** BU method; **(c)** PF method; **(d)** Fieller's method

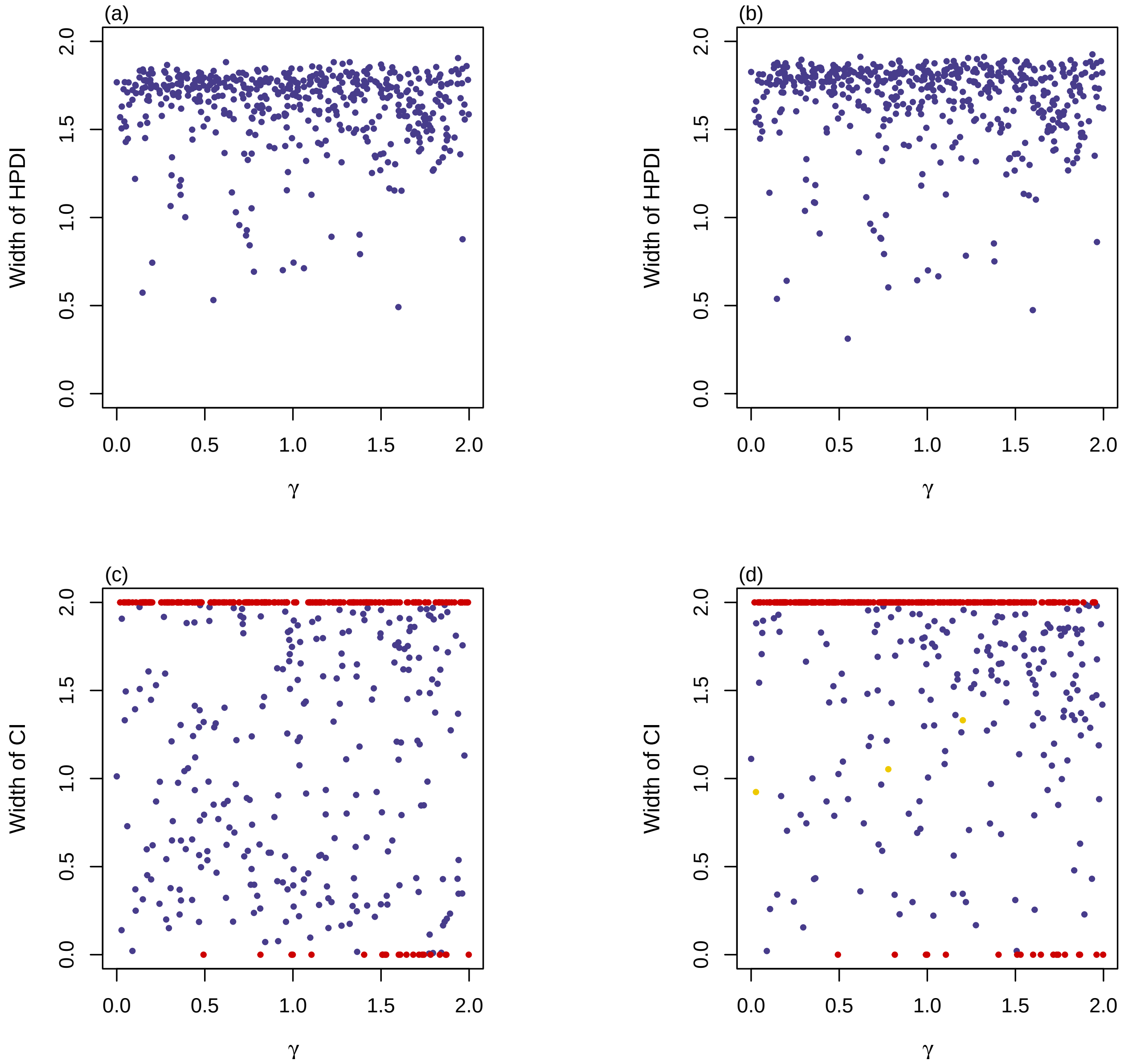

Supplementary Figure S60 Widths of HPDIs or CIs against true value of γ for quantitative trait when $(\sigma_0^2,\ \sigma_1^2,\ \sigma_2^2) = (4,\ 4.8,\ 4)$ with $n = 500$, MAF $= 0.1$ and $\rho = 0$. The red points represent the widths of the noninformative intervals or the empty sets, and the yellow points represent the widths of the discontinuous intervals. **(a)** BN method; **(b)** BU method; **(c)** PF method; **(d)** Fieller's method

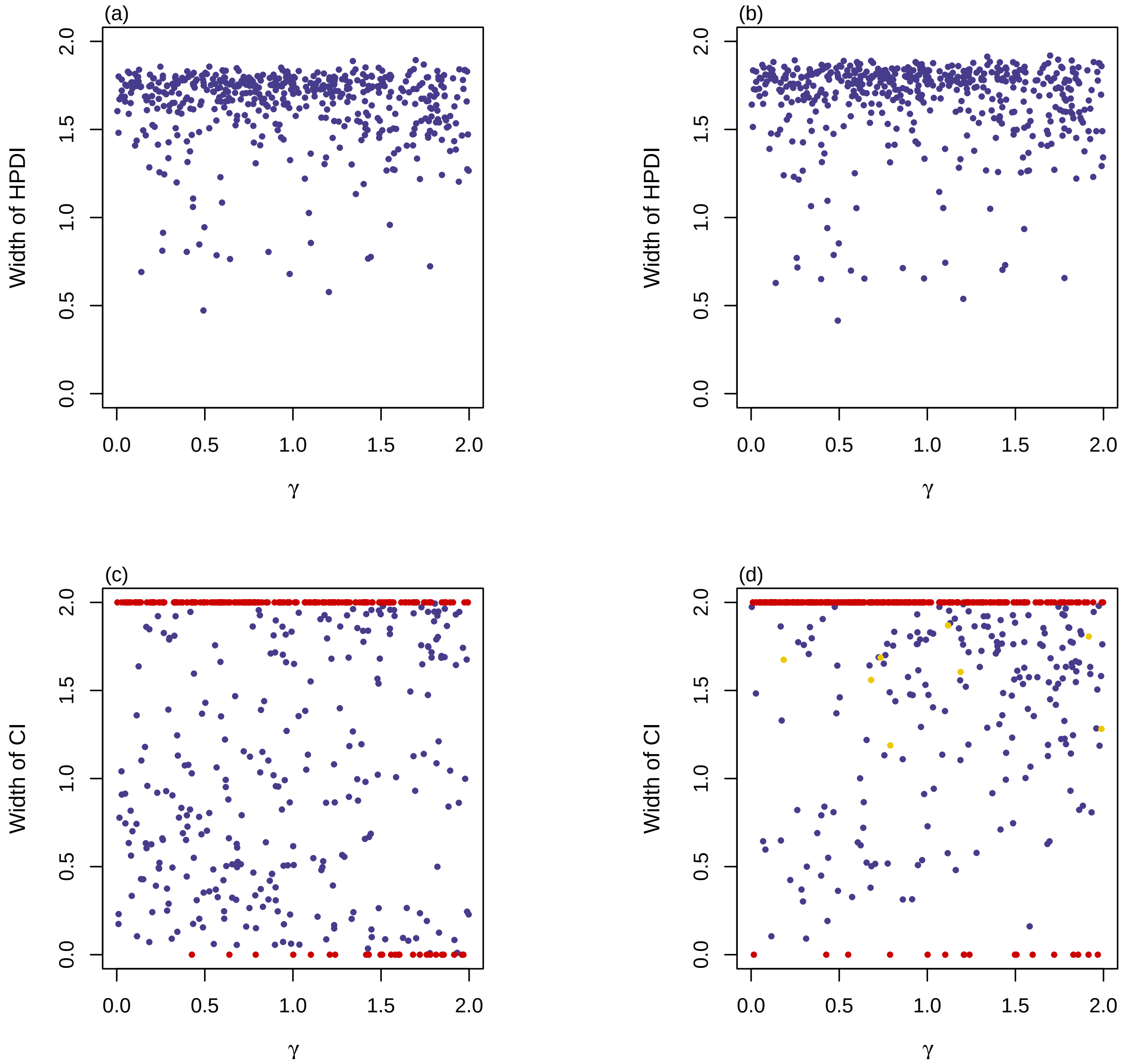

Supplementary Figure S61 Widths of HPDIs or CIs against true value of γ for quantitative trait when $(\sigma_0^2,\ \sigma_1^2,\ \sigma_2^2) = (4,\ 4.8,\ 4)$ with $n = 500$, MAF $= 0.1$ and $\rho = -0.05$. The red points represent the widths of the noninformative intervals or the empty sets, and the yellow points represent the widths of the discontinuous intervals. **(a)** BN method; **(b)** BU method; **(c)** PF method; **(d)** Fieller's method

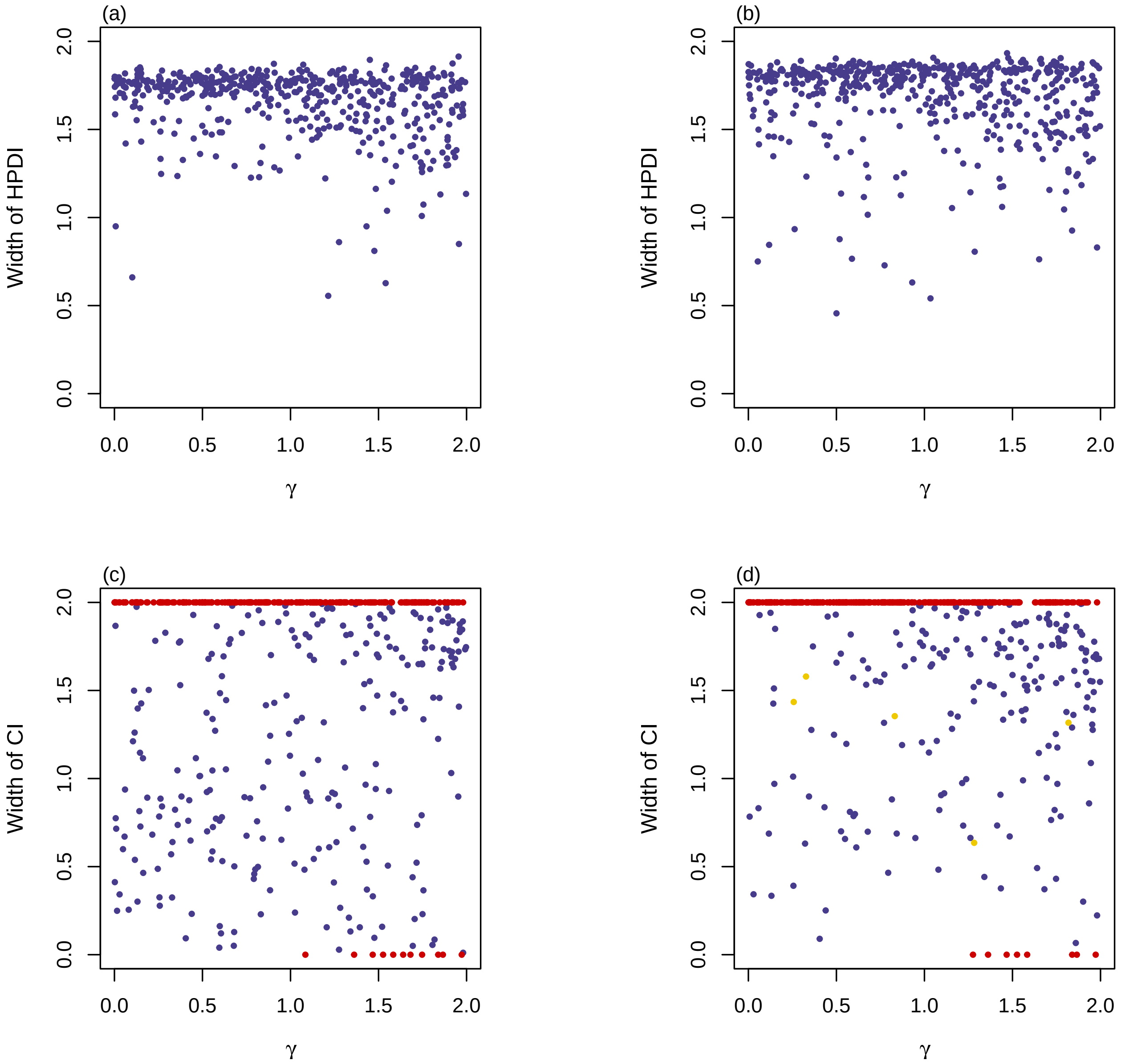

Supplementary Figure S62 Widths of HPDIs or CIs against true value of γ for quantitative trait when $(\sigma_0^2,\ \sigma_1^2,\ \sigma_2^2) = (4,\ 4.8,\ 4)$ with $n = 500$, MAF $= 0.1$ and $\rho = 0.05$. The red points represent the widths of the noninformative intervals or the empty sets, and the yellow points represent the widths of the discontinuous intervals. **(a)** BN method; **(b)** BU method; **(c)** PF method; **(d)** Fieller's method

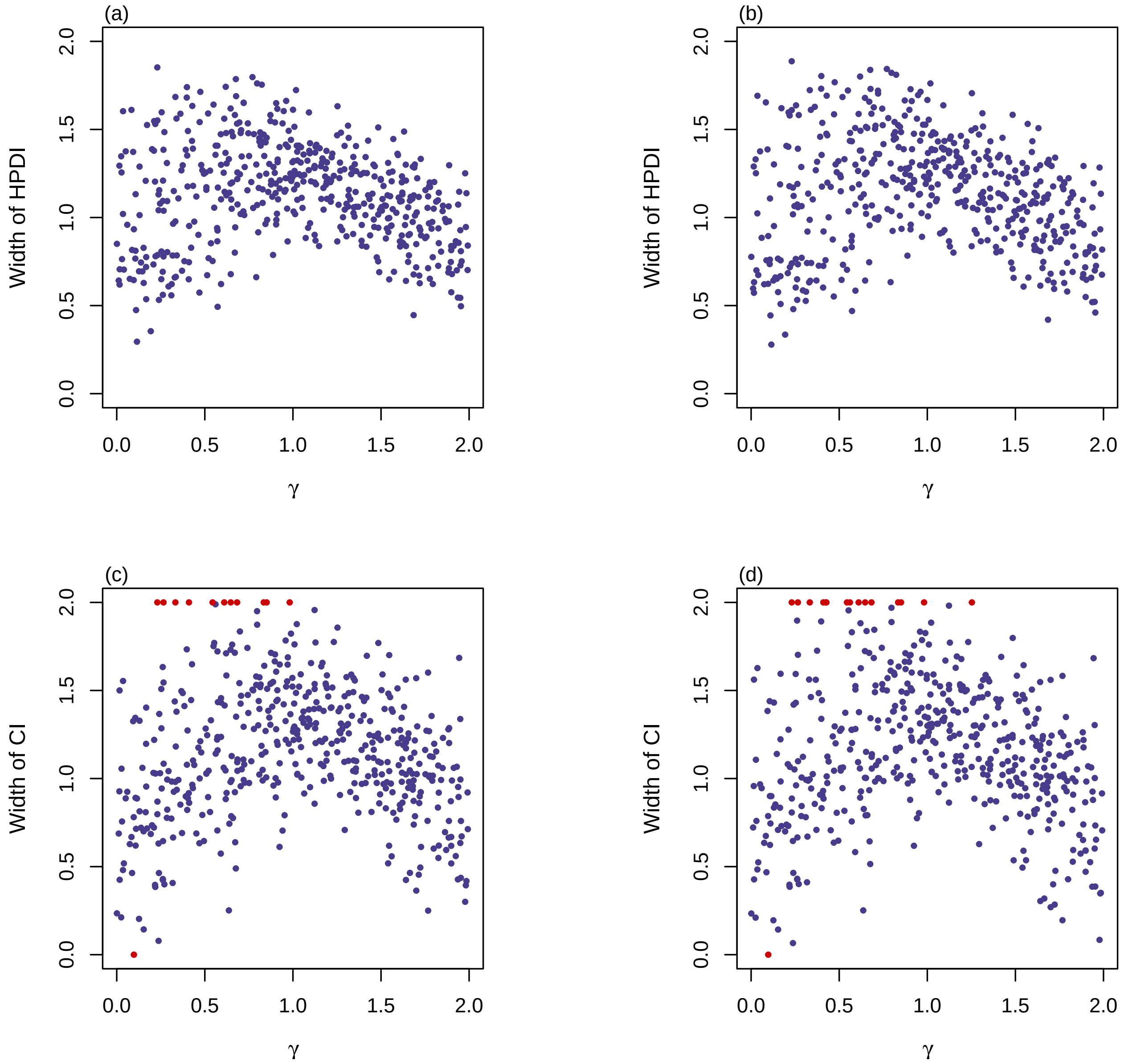

Supplementary Figure S63 Widths of HPDIs or CIs against true value of γ for quantitative trait when $(\sigma_0^2,\ \sigma_1^2,\ \sigma_2^2) = (4,\ 4.8,\ 4)$ with $n = 2000$, MAF $= 0.3$ and $\rho = 0$. The red points represent the widths of the noninformative intervals or the empty sets. **(a)** BN method; **(b)** BU method; **(c)** PF method; **(d)** Fieller's method

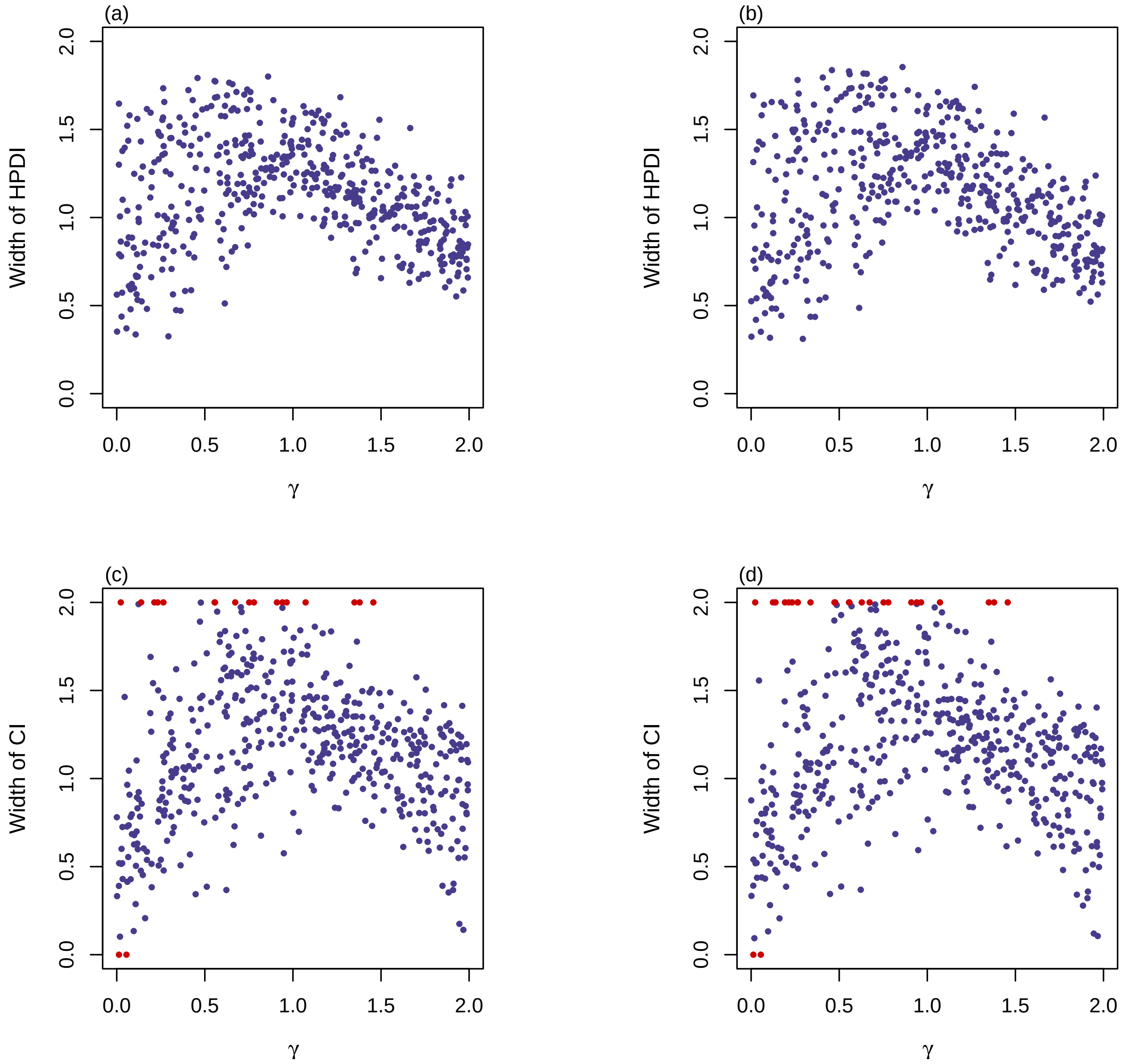

Supplementary Figure S64 Widths of HPDIs or CIs against true value of γ for quantitative trait when $(\sigma_0^2,\ \sigma_1^2,\ \sigma_2^2) = (4,\ 4.8,\ 4)$ with $n = 2000$, MAF $= 0.3$ and $\rho = -0.05$. The red points represent the widths of the noninformative intervals or the empty sets. **(a)** BN method; **(b)** BU method; **(c)** PF method; **(d)** Fieller's method

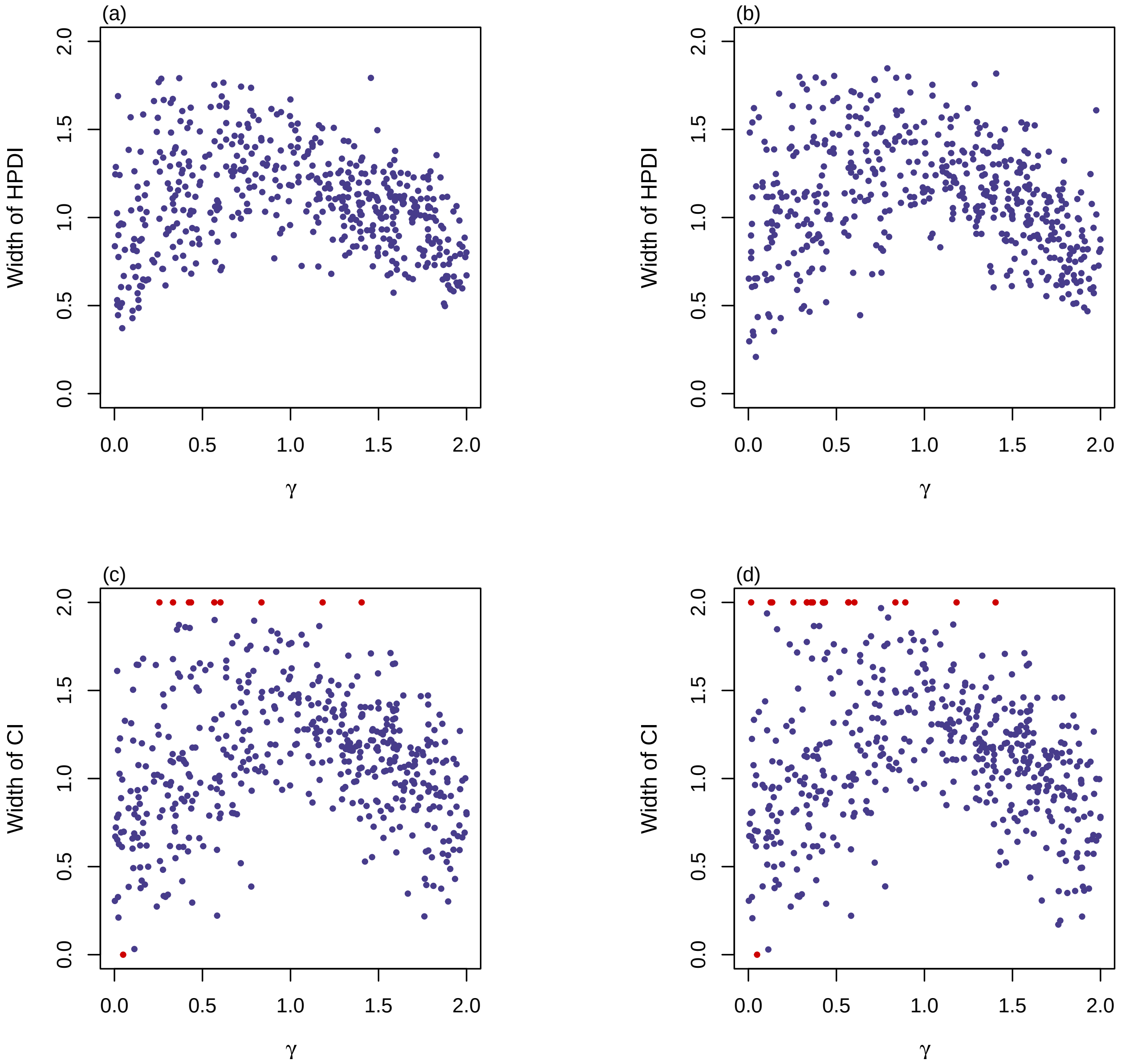

Supplementary Figure S65 Widths of HPDIs or CIs against true value of γ for quantitative trait when $(\sigma_0^2,\ \sigma_1^2,\ \sigma_2^2) = (4,\ 4.8,\ 4)$ with $n = 2000$, MAF $= 0.3$ and $\rho = 0.05$. The red points represent the widths of the noninformative intervals or the empty sets. **(a)** BN method; **(b)** BU method; **(c)** PF method; **(d)** Fieller's method

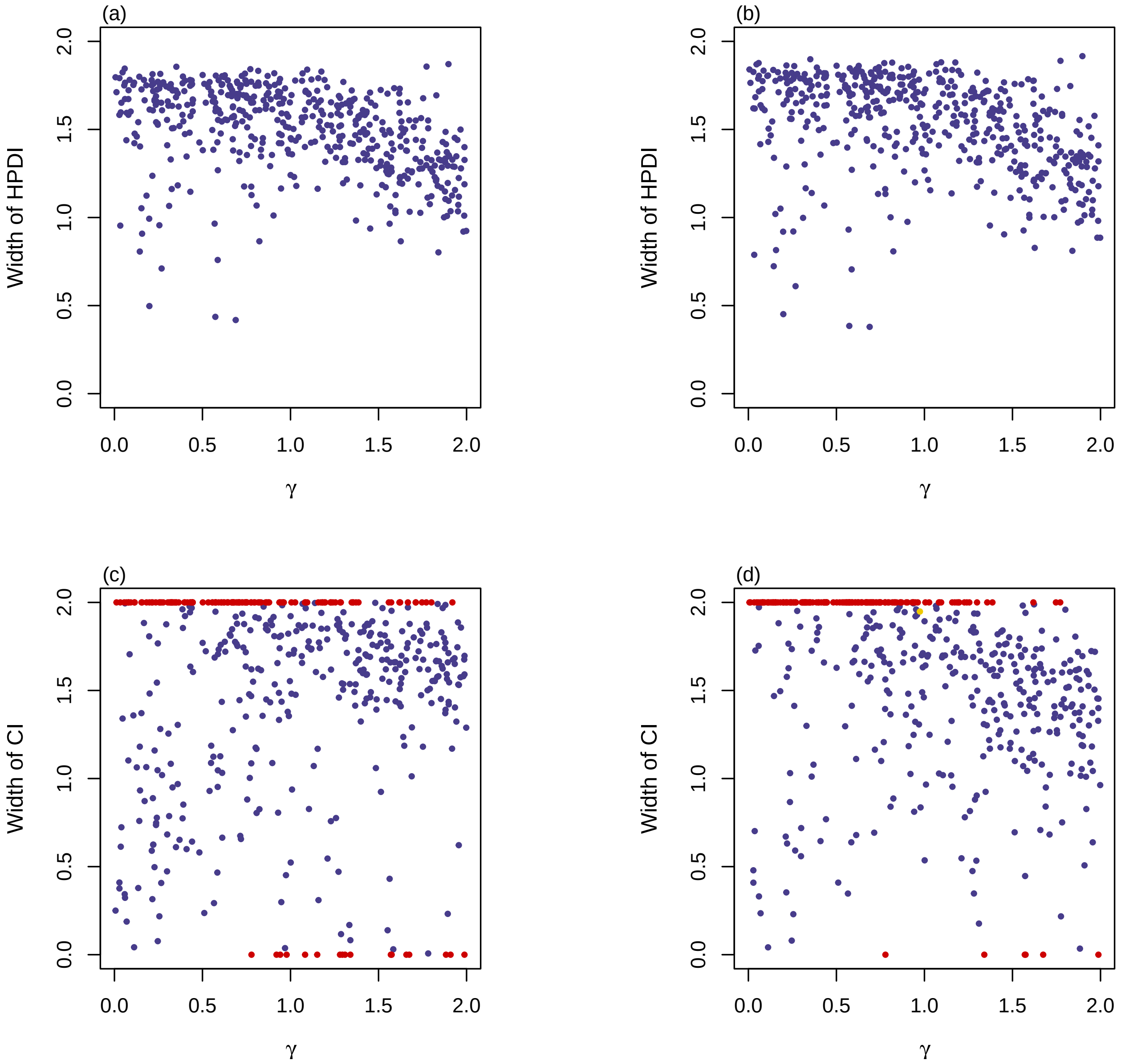

Supplementary Figure S66 Widths of HPDIs or CIs against true value of γ for quantitative trait when $(\sigma_0^2,\ \sigma_1^2,\ \sigma_2^2) = (4,\ 4.8,\ 4)$ with $n = 2000$, MAF $= 0.1$ and $\rho = 0$. The red points represent the widths of the noninformative intervals or the empty sets, and the yellow point represents the width of the discontinuous interval. **(a)** BN method; **(b)** BU method; **(c)** PF method; **(d)** Fieller's method

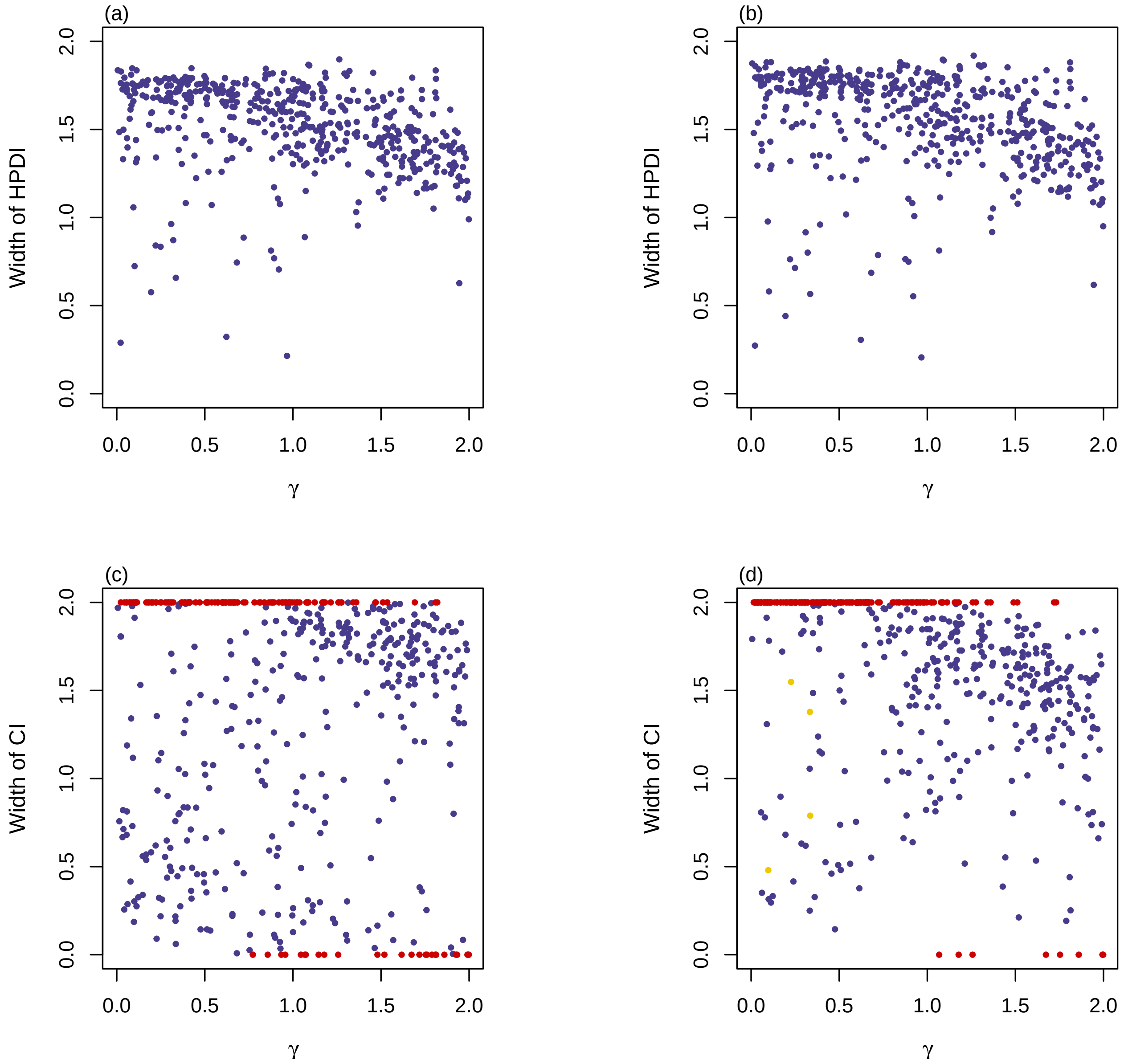

Supplementary Figure S67 Widths of HPDIs or CIs against true value of γ for quantitative trait when $(\sigma_0^2,\ \sigma_1^2,\ \sigma_2^2) = (4,\ 4.8,\ 4)$ with $n = 2000$, MAF $= 0.1$ and $\rho = -0.05$. The red points represent the widths of the noninformative intervals or the empty sets, and the yellow points represent the widths of the discontinuous intervals. **(a)** BN method; **(b)** BU method; **(c)** PF method; **(d)** Fieller's method

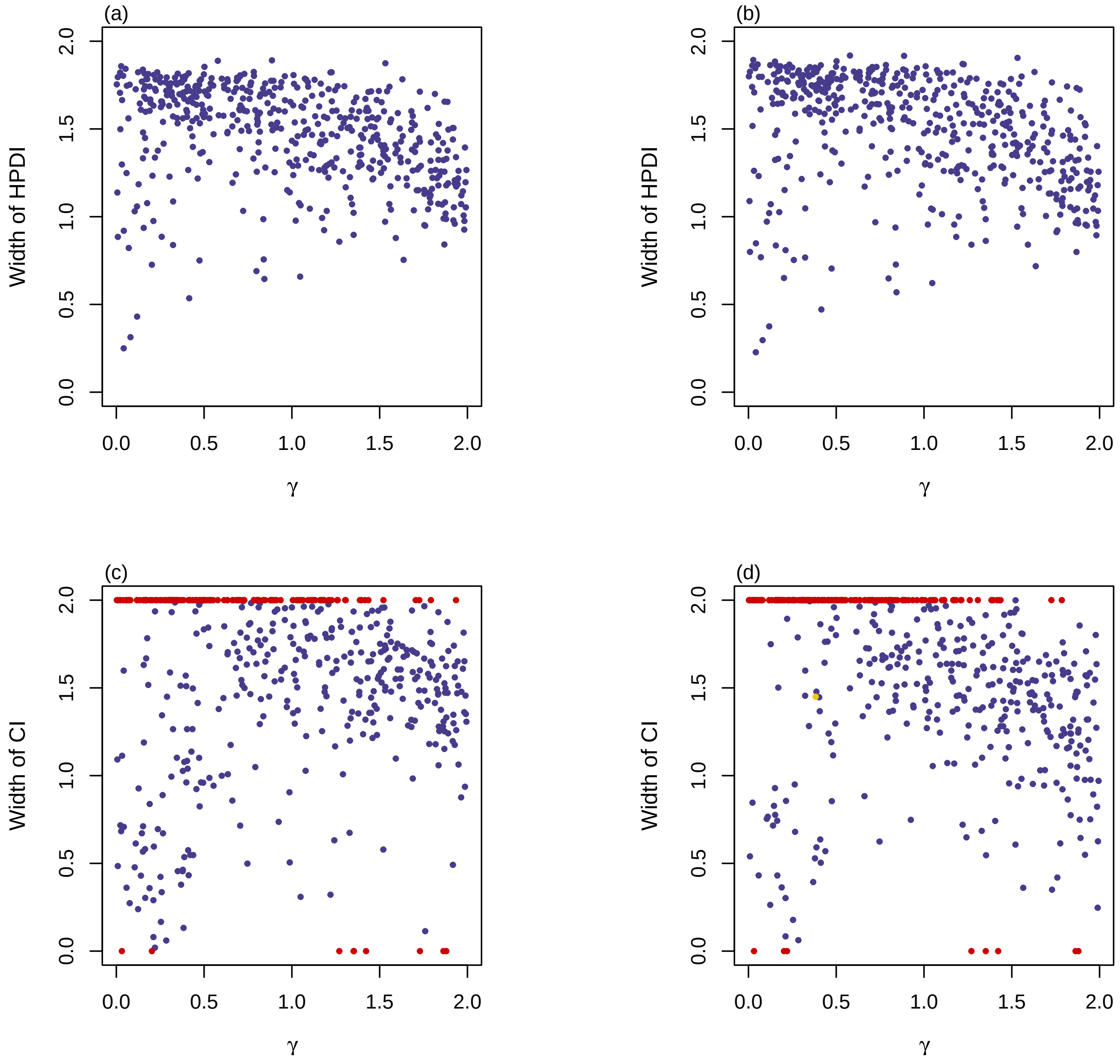

Supplementary Figure S68 Widths of HPDIs or CIs against true value of γ for quantitative trait when $(\sigma_0^2,\ \sigma_1^2,\ \sigma_2^2) = (4,\ 4.8,\ 4)$ with $n = 2000$, MAF $= 0.1$ and $\rho = 0.05$. The red points represent the widths of the noninformative intervals or the empty sets, and the yellow point represents the width of the discontinuous interval. **(a)** BN method; **(b)** BU method; **(c)** PF method; **(d)** Fieller's method

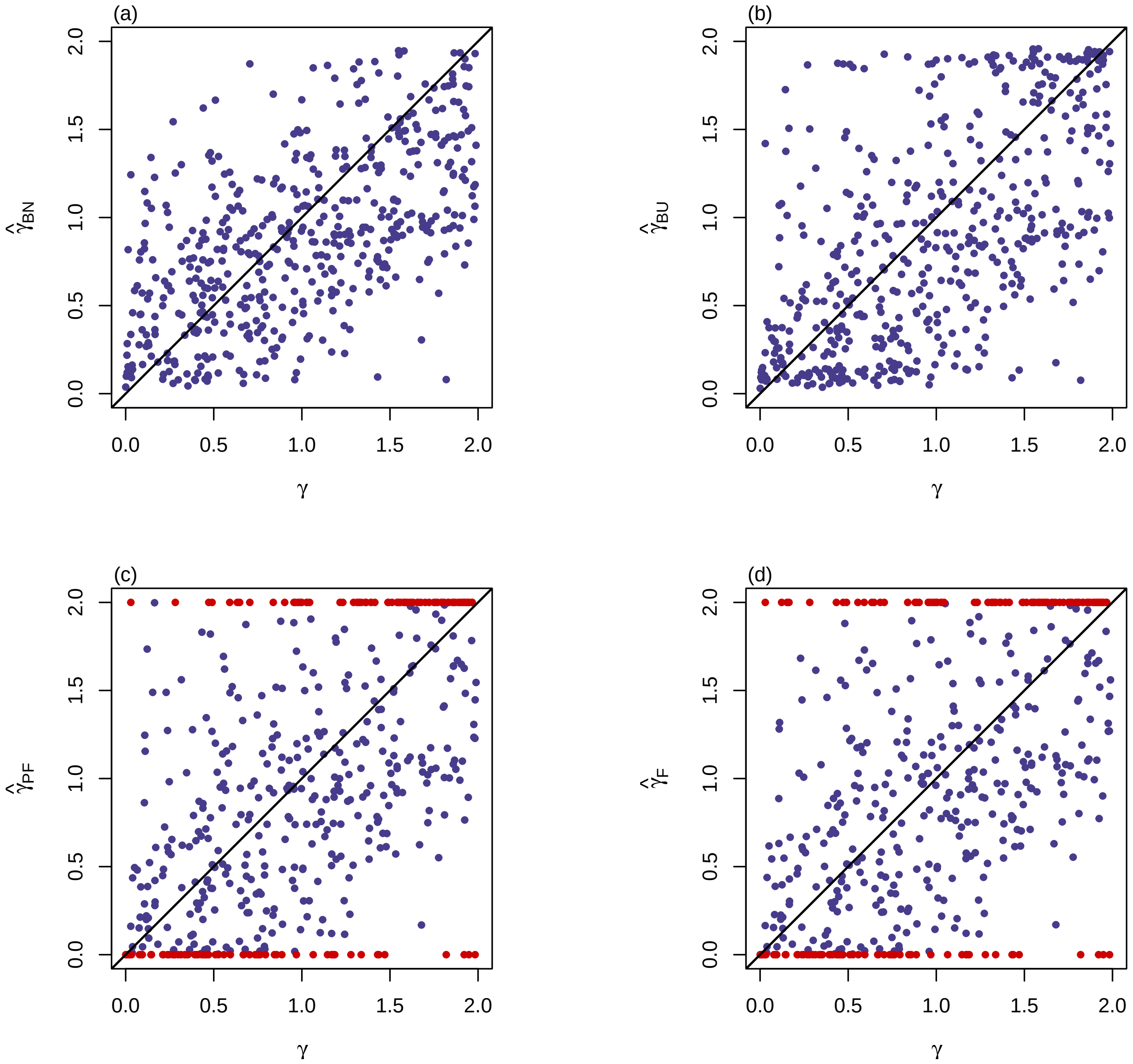

Supplementary Figure S69 Scatter plots of point estimates of γ for qualitative trait with a covariate when $n = 500$, MAF $= 0.3$ and $\rho = 0$. The results are against true value of γ. The red points represent the extreme values (0 or 2). **(a)** $\hat{\gamma}_{BN}$; **(b)** $\hat{\gamma}_{BU}$; **(c)** $\hat{\gamma}_{PF}$; **(d)** $\hat{\gamma}_{F}$

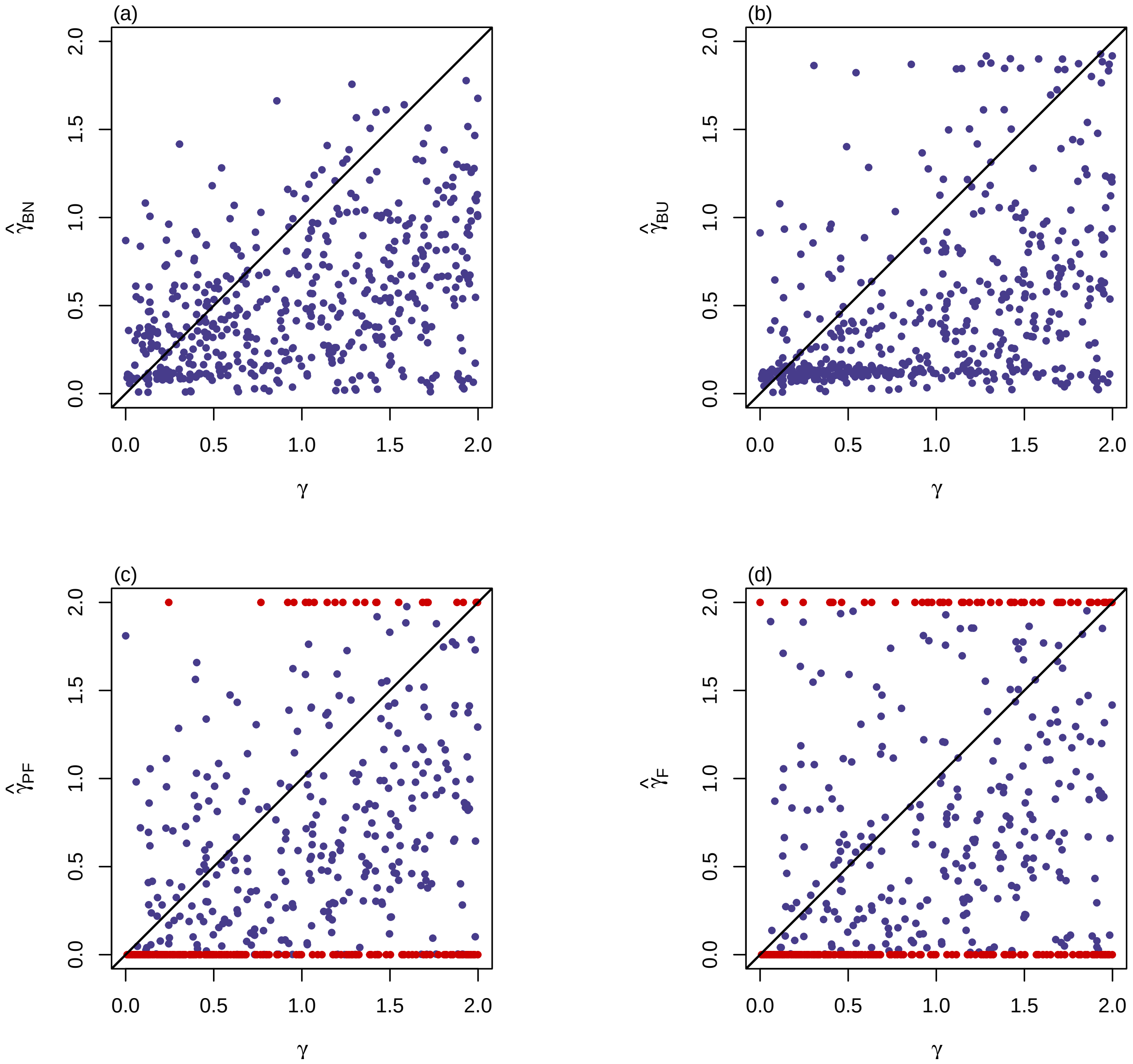

Supplementary Figure S70 Scatter plots of point estimates of γ for qualitative trait with a covariate when $n = 500$, MAF $= 0.1$ and $\rho = 0$. The results are against true value of γ. The red points represent the extreme values (0 or 2). **(a)** $\hat{\gamma}_{BN}$; **(b)** $\hat{\gamma}_{BU}$; **(c)** $\hat{\gamma}_{PF}$; **(d)** $\hat{\gamma}_{F}$

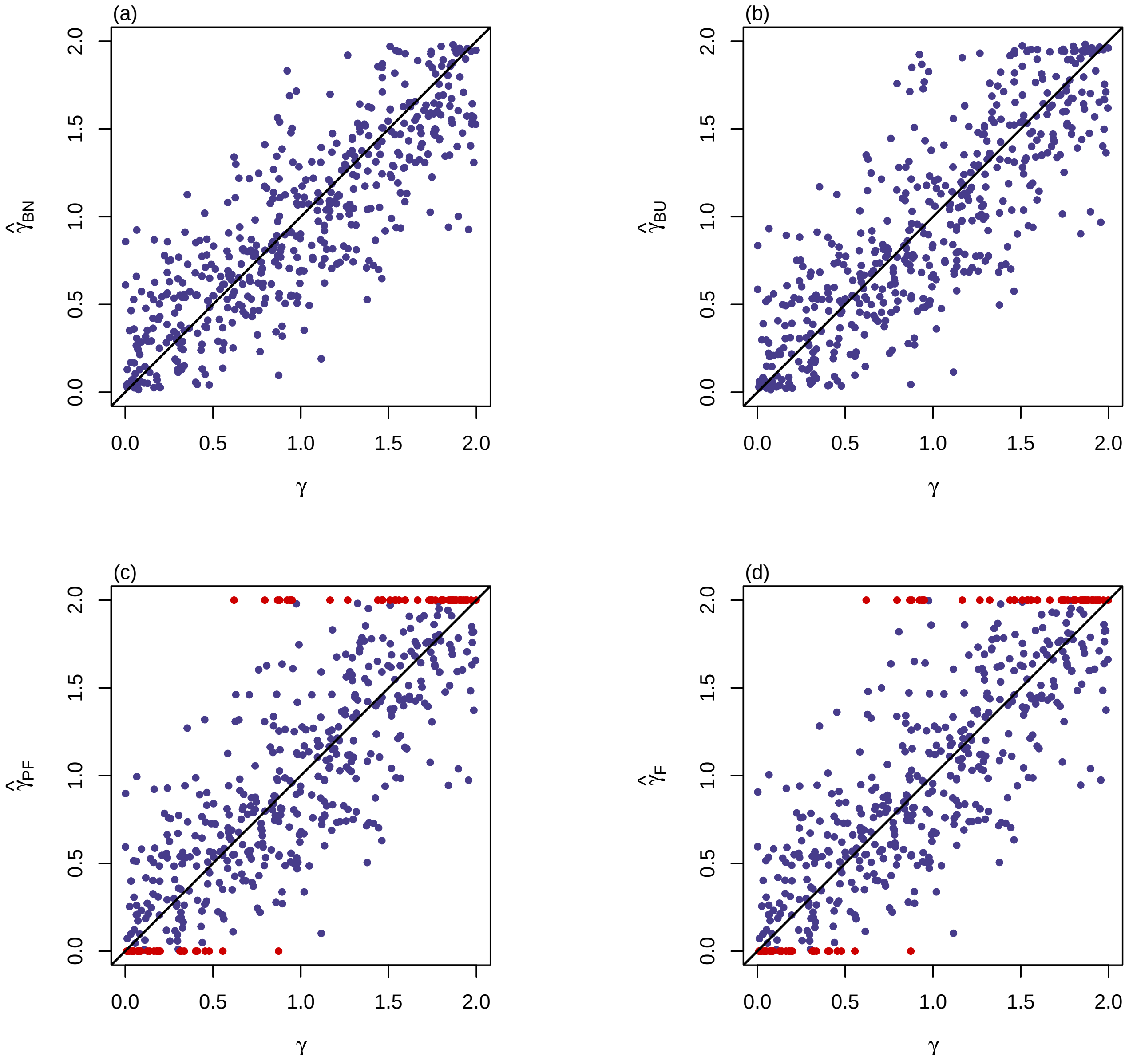

Supplementary Figure S71 Scatter plots of point estimates of γ for qualitative trait with a covariate when $n = 2000$, MAF $= 0.3$ and $\rho = 0$. The results are against true value of γ. The red points represent the extreme values (0 or 2). **(a)** $\hat{\gamma}_{BN}$; **(b)** $\hat{\gamma}_{BU}$; **(c)** $\hat{\gamma}_{PF}$; **(d)** $\hat{\gamma}_{F}$

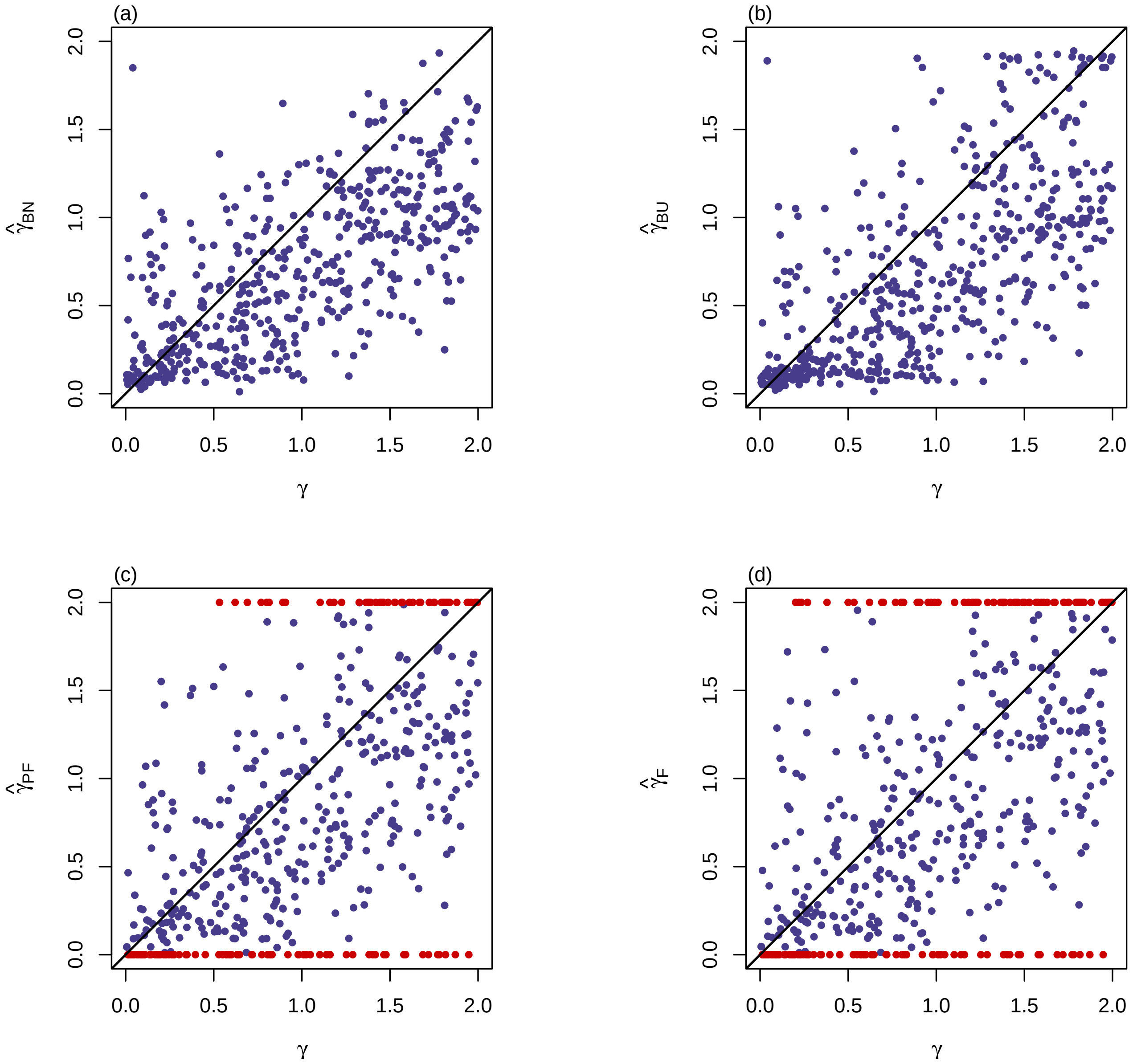

Supplementary Figure S72 Scatter plots of point estimates of γ for qualitative trait with a covariate when $n = 2000$, MAF $= 0.1$ and $\rho = 0$. The results are against true value of γ. The red points represent the extreme values (0 or 2). **(a)** $\hat{\gamma}_{BN}$; **(b)** $\hat{\gamma}_{BU}$; **(c)** $\hat{\gamma}_{PF}$; **(d)** $\hat{\gamma}_{F}$

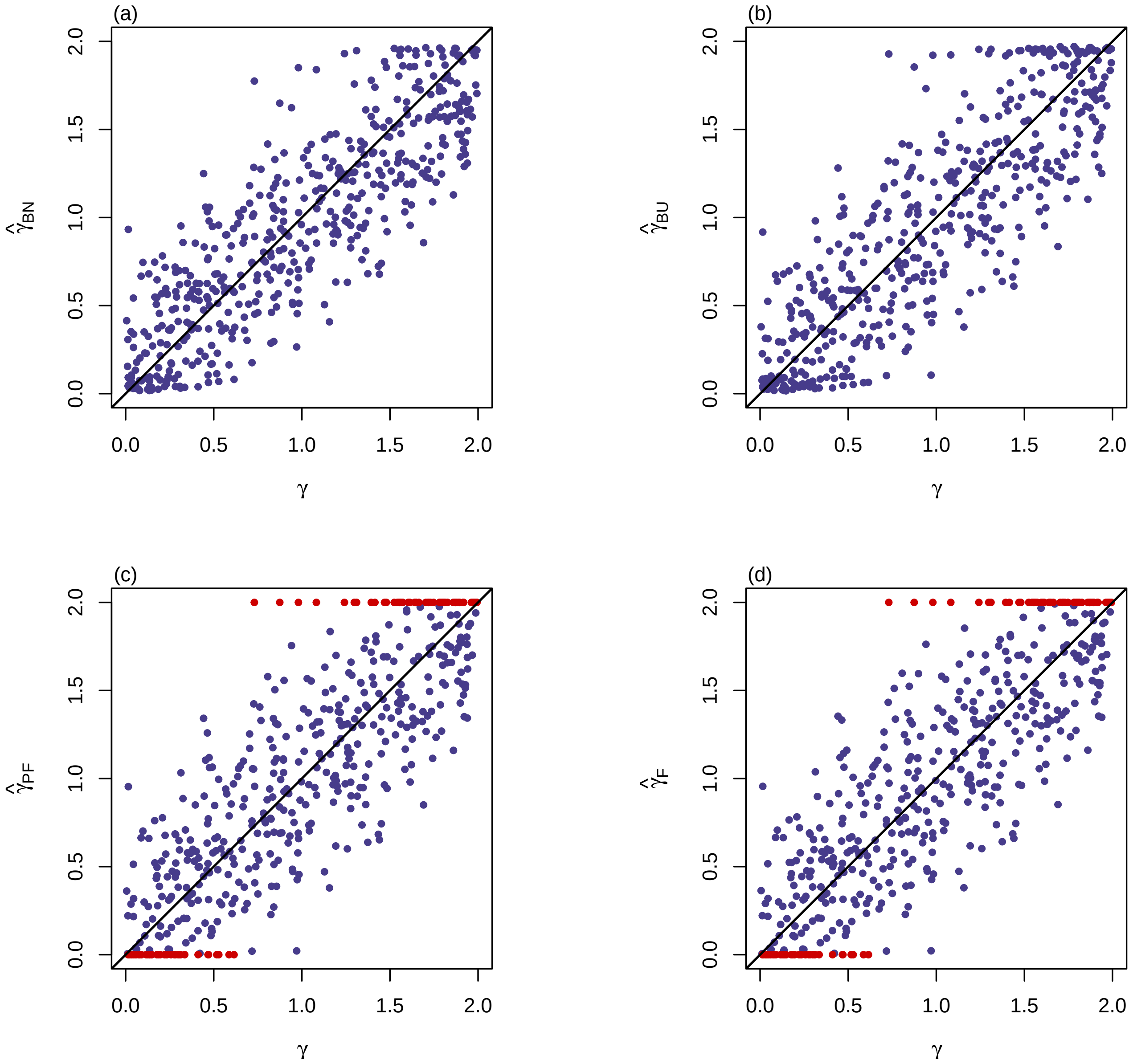

Supplementary Figure S73 Scatter plots of point estimates of γ for quantitative trait with a covariate when $n = 500$, MAF $= 0.3$ and $\rho = 0$. The results are against true value of γ with $(\sigma_0^2,\ \sigma_1^2,\ \sigma_2^2) = (1,\ 1.2,\ 1)$. The red points represent the extreme values (0 or 2). **(a)** $\hat{\gamma}_{BN}$; **(b)** $\hat{\gamma}_{BU}$; **(c)** $\hat{\gamma}_{PF}$; **(d)** $\hat{\gamma}_{F}$

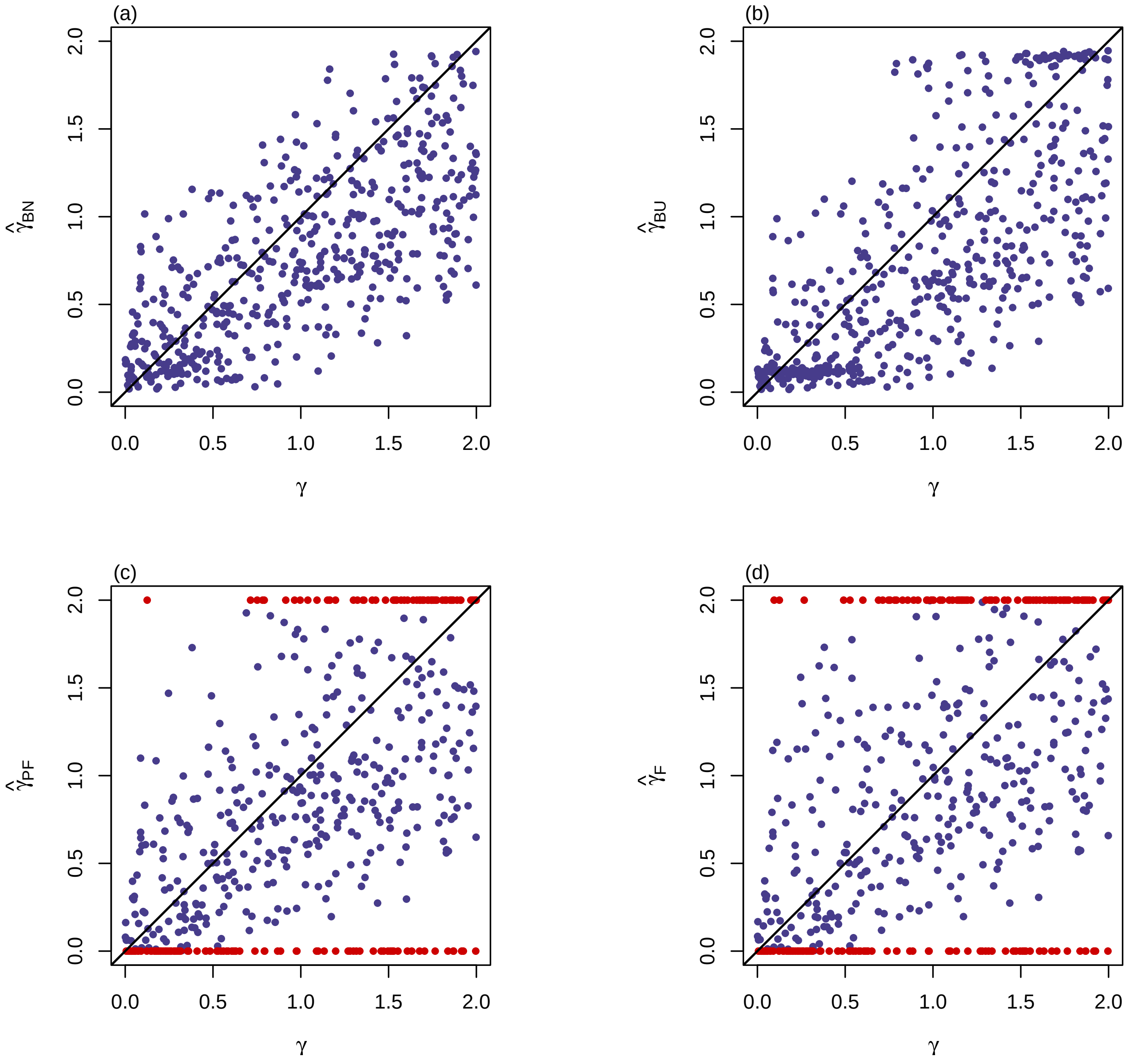

Supplementary Figure S74 Scatter plots of point estimates of γ for quantitative trait with a covariate when $n = 500$, MAF $= 0.1$ and $\rho = 0$. The results are against true value of γ with $(\sigma_0^2,\ \sigma_1^2,\ \sigma_2^2) = (1,\ 1.2,\ 1)$. The red points represent the extreme values (0 or 2). **(a)** $\hat{\gamma}_{BN}$; **(b)** $\hat{\gamma}_{BU}$; **(c)** $\hat{\gamma}_{PF}$; **(d)** $\hat{\gamma}_F$

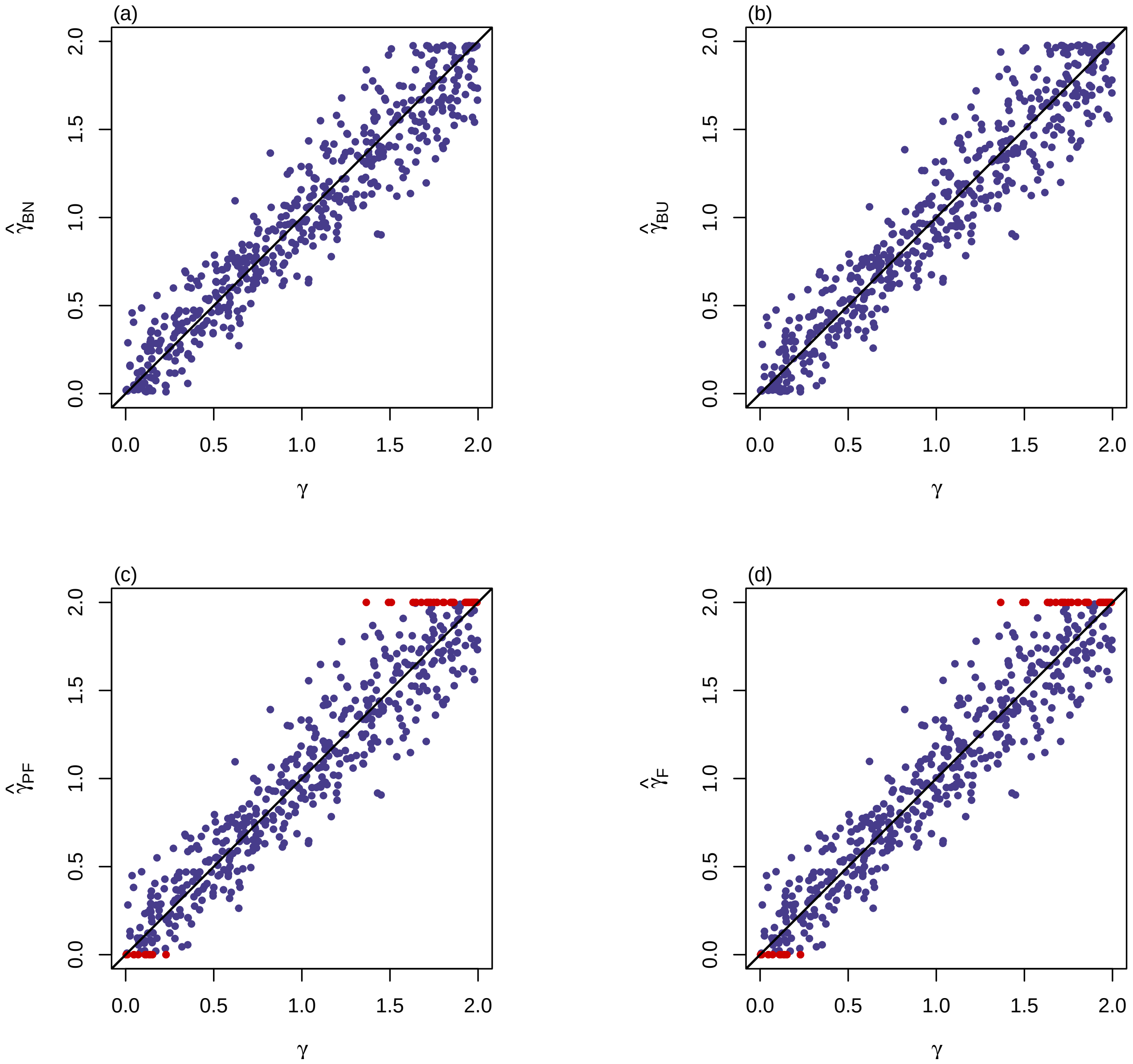

Supplementary Figure S75 Scatter plots of point estimates of γ for quantitative trait with a covariate when $n = 2000$, MAF $= 0.3$ and $\rho = 0$. The results are against true value of γ with $(\sigma_0^2,\ \sigma_1^2,\ \sigma_2^2) = (1,\ 1.2,\ 1)$. The red points represent the extreme values (0 or 2). **(a)** $\hat{\gamma}_{BN}$; **(b)** $\hat{\gamma}_{BU}$; **(c)** $\hat{\gamma}_{PF}$; **(d)** $\hat{\gamma}_{F}$

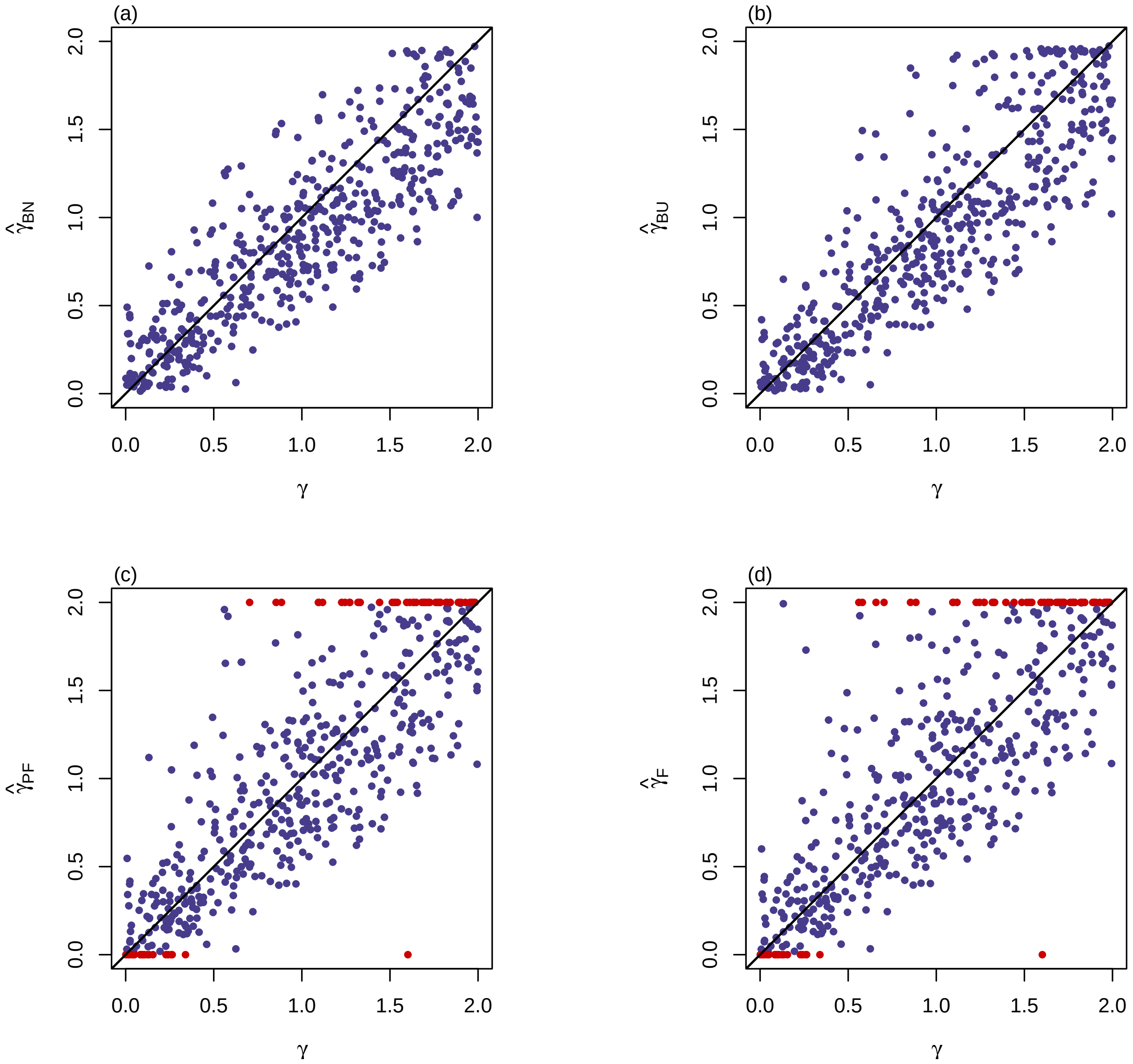

Supplementary Figure S76 Scatter plots of point estimates of γ for quantitative trait with a covariate when $n = 2000$, MAF $= 0.1$ and $\rho = 0$. The results are against true value of γ with $(\sigma_0^2,\ \sigma_1^2,\ \sigma_2^2) = (1,\ 1.2,\ 1)$. The red points represent the extreme values (0 or 2). **(a)** $\hat{\gamma}_{BN}$; **(b)** $\hat{\gamma}_{BU}$; **(c)** $\hat{\gamma}_{PF}$; **(d)** $\hat{\gamma}_F$

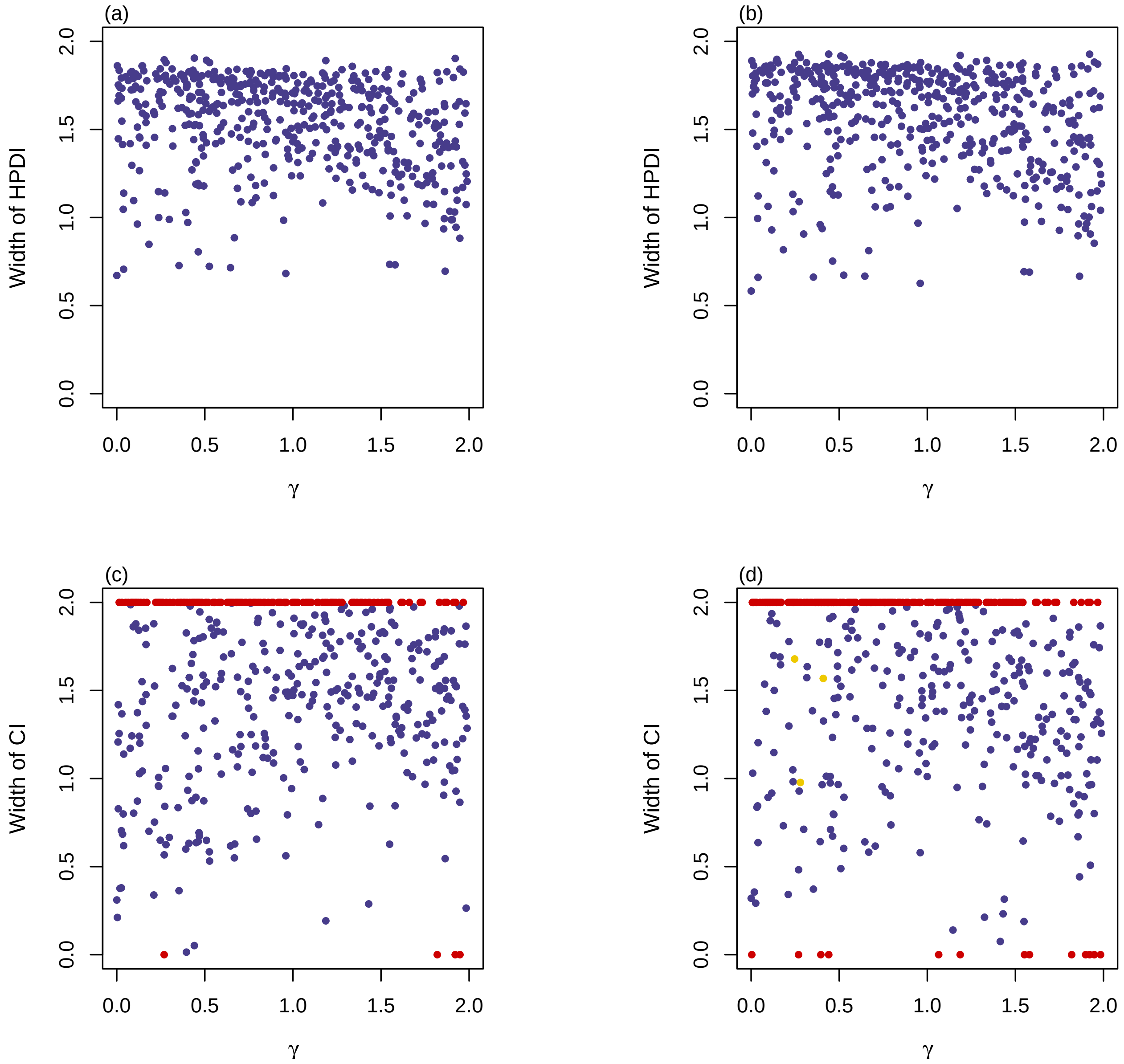

Supplementary Figure S77 Widths of HPDIs or CIs for qualitative trait with a covariate when $n = 500$, MAF $= 0.3$ and $\rho = 0$. The results are against true value of γ. The red points represent the widths of the noninformative intervals or the empty sets, and the yellow points represent the widths of the discontinuous intervals. **(a)** BN method; **(b)** BU method; **(c)** PF method; **(d)** Fieller's method

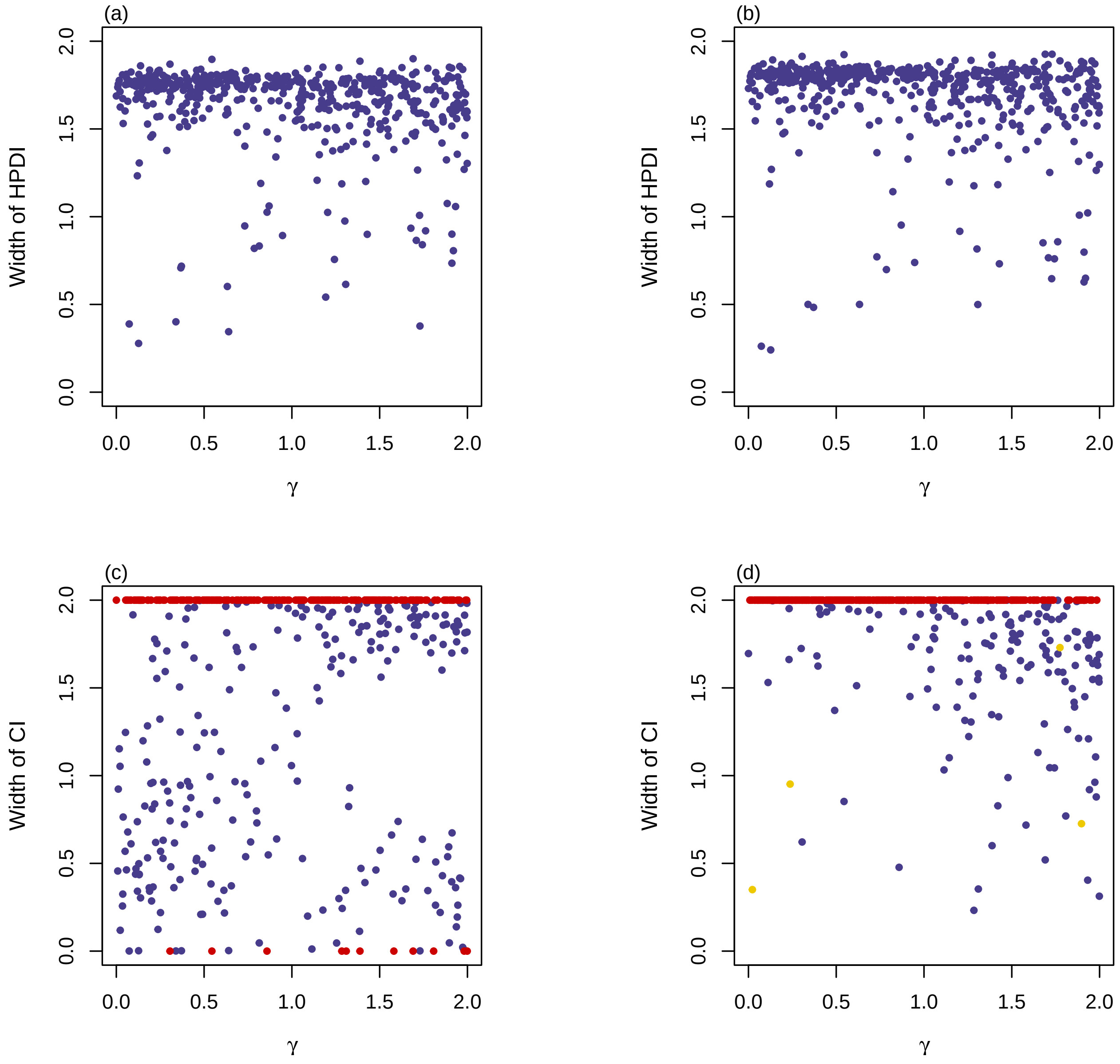

Supplementary Figure S78 Widths of HPDIs or CIs for qualitative trait with a covariate when $n = 500$, MAF $= 0.1$ and $\rho = 0$. The results are against true value of γ. The red points represent the widths of the noninformative intervals or the empty sets, and the yellow points represent the widths of the discontinuous intervals. **(a)** BN method; **(b)** BU method; **(c)** PF method; **(d)** Fieller's method

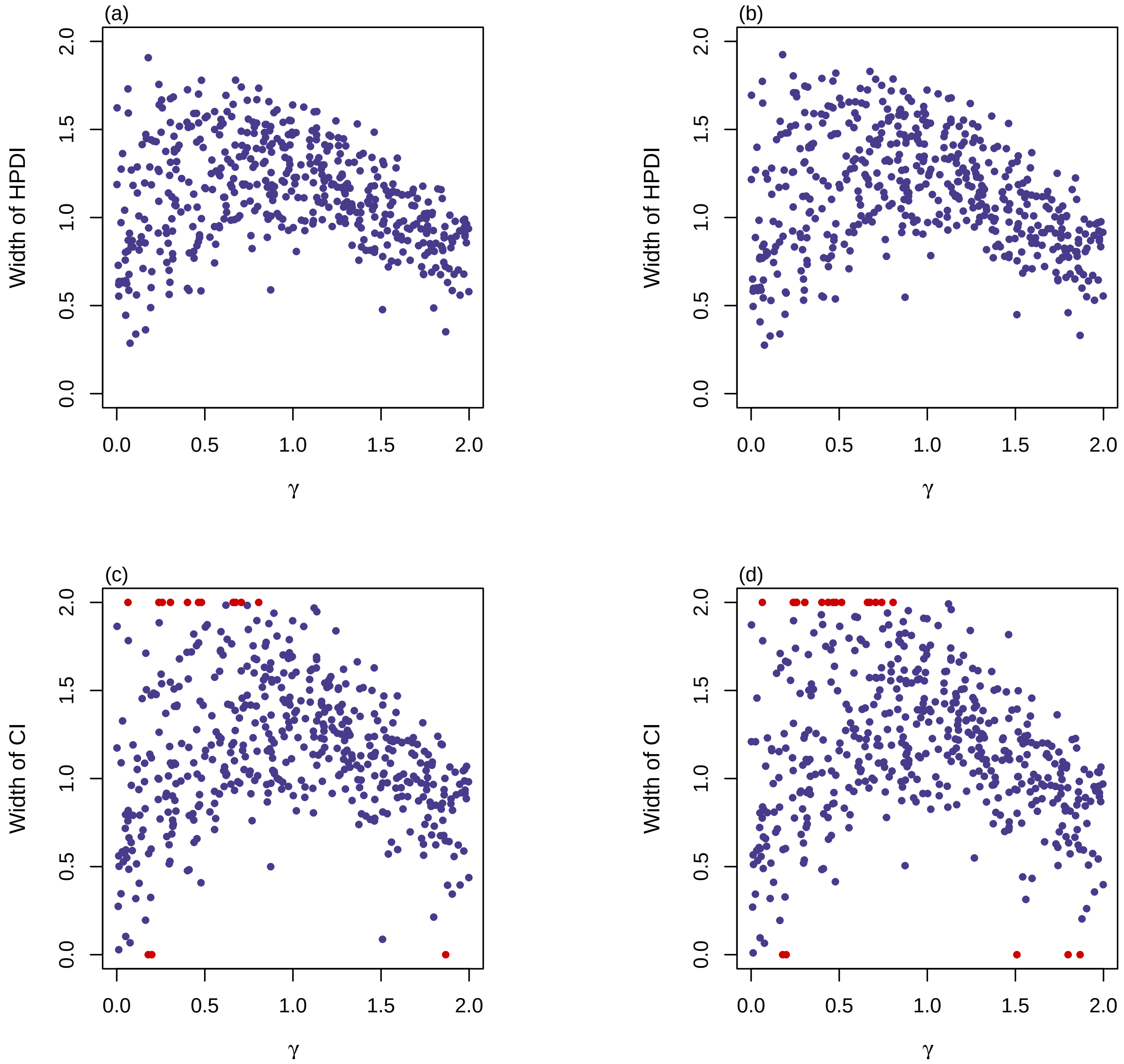

Supplementary Figure S79 Widths of HPDIs or CIs for qualitative trait with a covariate when $n = 2000$, MAF $= 0.3$ and $\rho = 0$. The results are against true value of γ. The red points represent the widths of the noninformative intervals or the empty sets. **(a)** BN method; **(b)** BU method; **(c)** PF method; **(d)** Fieller's method

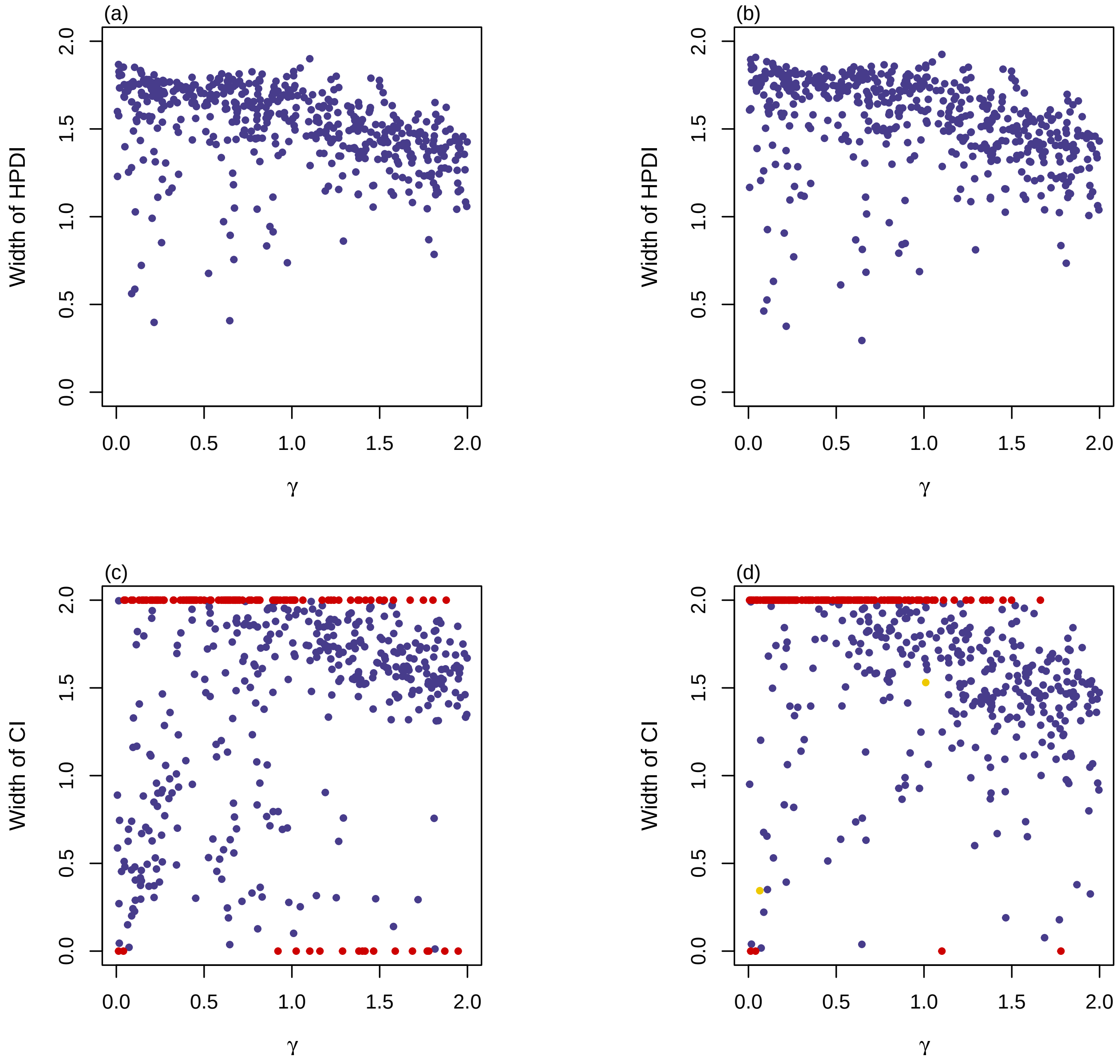

Supplementary Figure S80 Widths of HPDIs or CIs for qualitative trait with a covariate when $n = 2000$, MAF $= 0.1$ and $\rho = 0$. The results are against true value of γ. The red points represent the widths of the noninformative intervals or the empty sets, and the yellow points represent the widths of the discontinuous intervals. **(a)** BN method; **(b)** BU method; **(c)** PF method; **(d)** Fieller's method

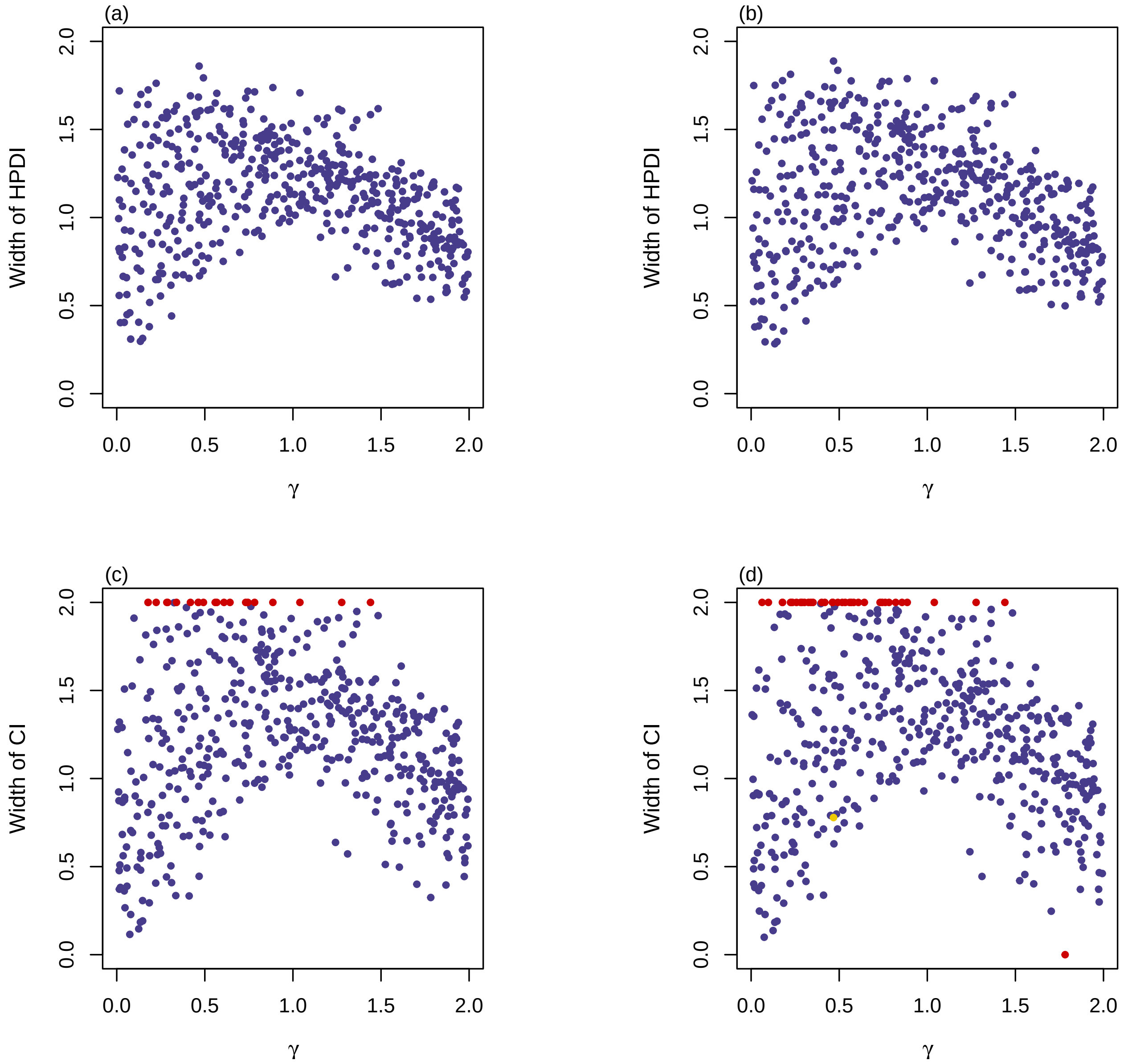

Supplementary Figure S81 Widths of HPDIs or CIs for quantitative trait with a covariate when $n = 500$, MAF $= 0.3$ and $\rho = 0$. The results are against true value of γ with $(\sigma_0^2,\ \sigma_1^2,\ \sigma_2^2) = (1,\ 1.2,\ 1)$. The red points represent the widths of the noninformative intervals or the empty sets, and the yellow point represents the width of the discontinuous interval. **(a)** BN method; **(b)** BU method; **(c)** PF method; **(d)** Fieller's method

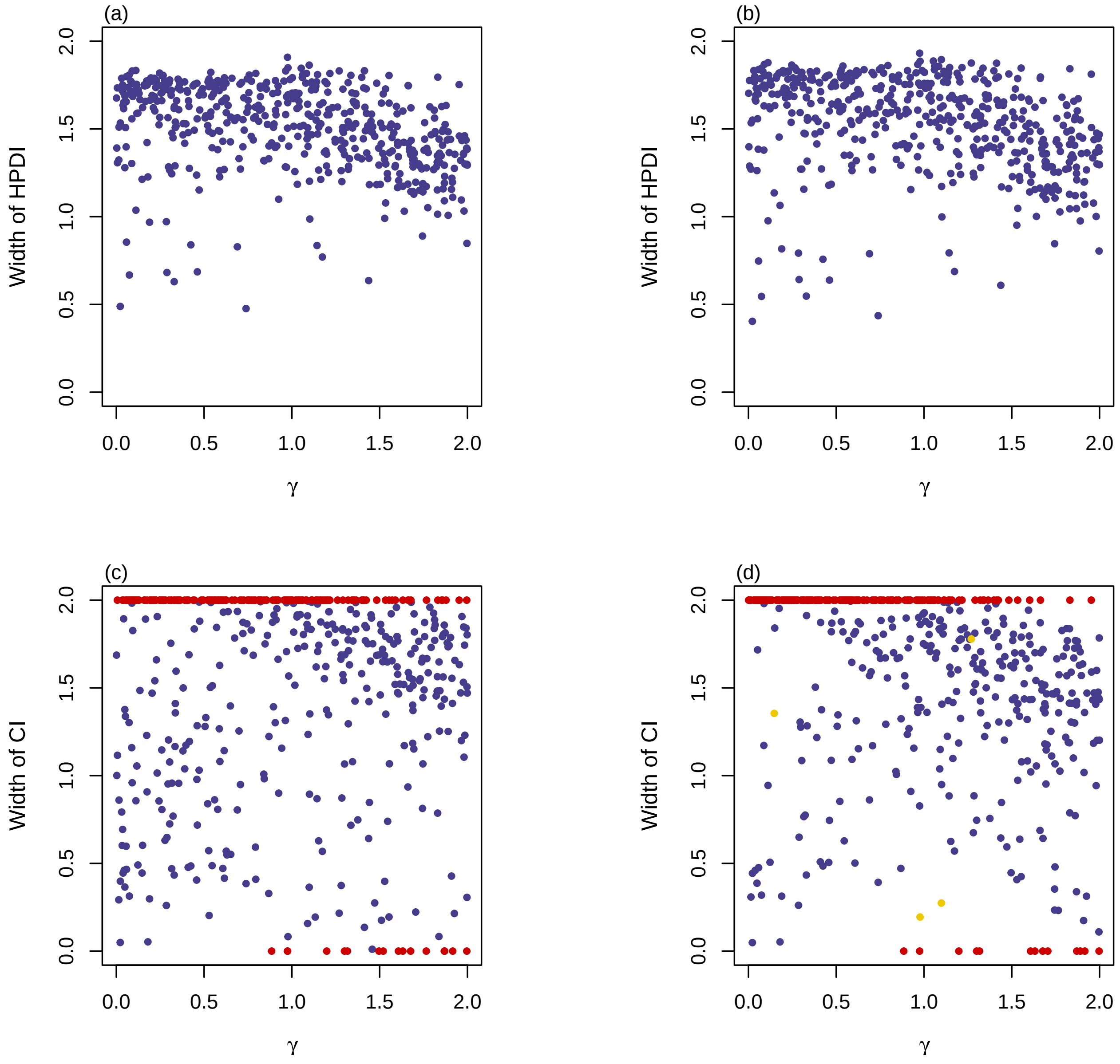

Supplementary Figure S82 Widths of HPDIs or CIs for quantitative trait with a covariate when $n = 500$, MAF $= 0.1$ and $\rho = 0$. The results are against true value of γ with $(\sigma_0^2,\ \sigma_1^2,\ \sigma_2^2) = (1,\ 1.2,\ 1)$. The red points represent the widths of the noninformative intervals or the empty sets, and the yellow points represent the widths of the discontinuous intervals. **(a)** BN method; **(b)** BU method; **(c)** PF method; **(d)** Fieller's method

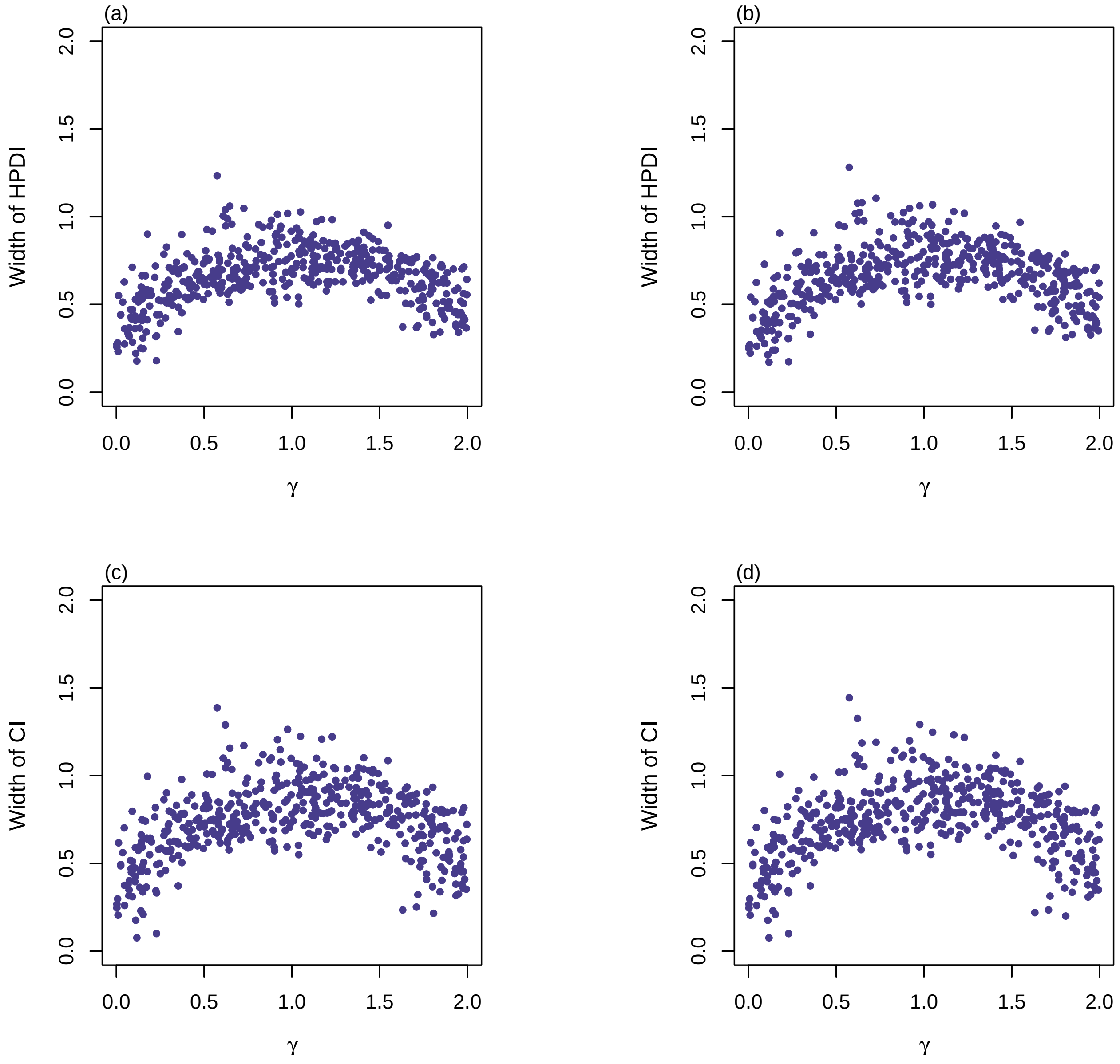

Supplementary Figure S83 Widths of HPDIs or CIs for quantitative trait with a covariate when $n = 2000$, MAF $= 0.3$ and $\rho = 0$. The results are against true value of γ with $(\sigma_0^2,\ \sigma_1^2,\ \sigma_2^2) = (1,\ 1.2,\ 1)$. **(a)** BN method; **(b)** BU method; **(c)** PF method; **(d)** Fieller's method

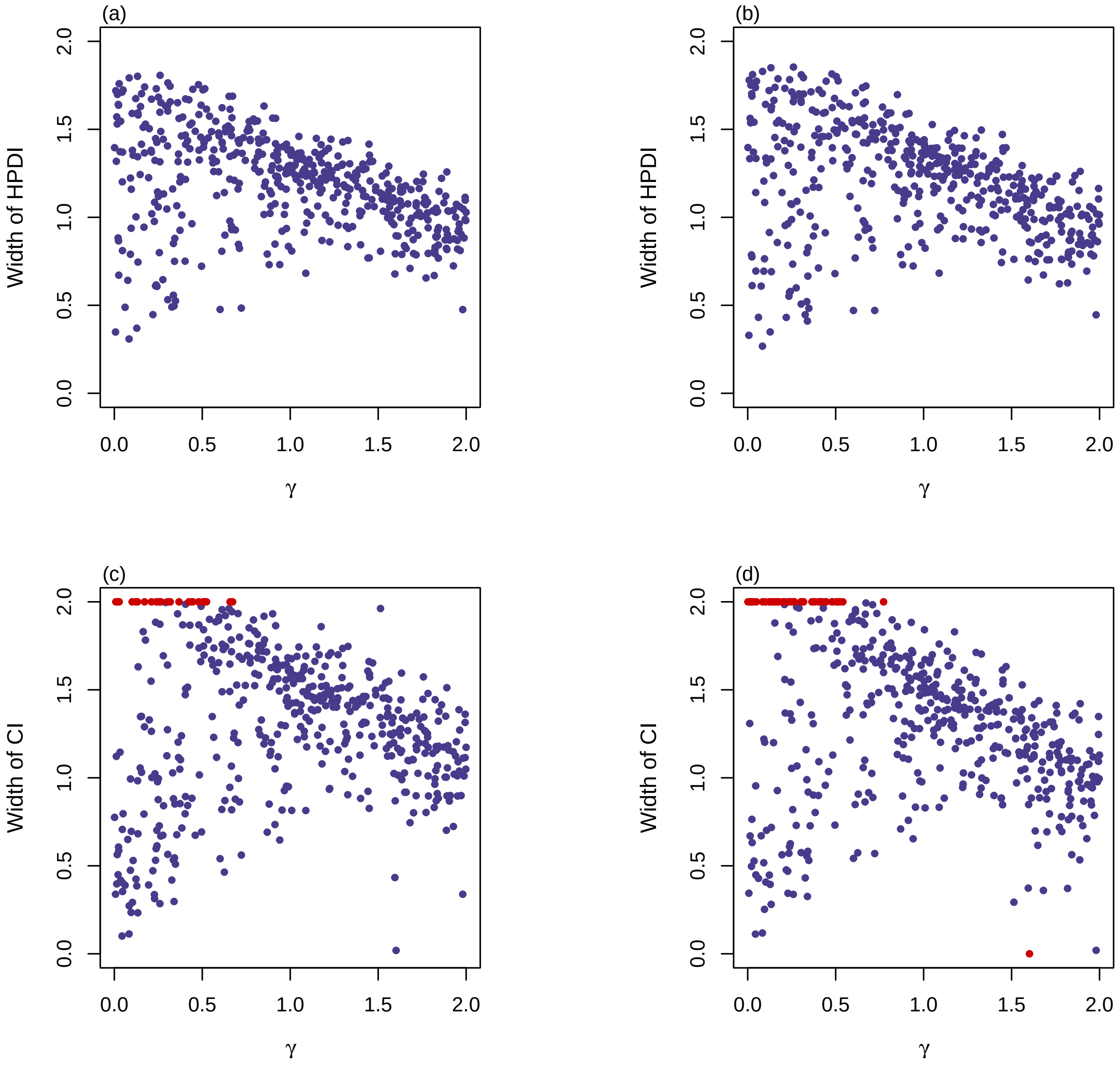

Supplementary Figure S84 Widths of HPDIs or CIs for quantitative trait with a covariate when $n = 2000$, MAF $= 0.1$ and $\rho = 0$. The results are against true value of γ with $(\sigma_0^2,\ \sigma_1^2,\ \sigma_2^2) = (1,\ 1.2,\ 1)$. The red points represent the widths of the noninformative intervals or the empty sets. **(a)** BN method; **(b)** BU method; **(c)** PF method; **(d)** Fieller's method
